# Synthesis of Gibberellic Acid Derivatives and Their Effects on Plant Growth

**DOI:** 10.3390/molecules22050694

**Published:** 2017-04-26

**Authors:** Hao Tian, Yiren Xu, Shaojin Liu, Dingsha Jin, Jianjun Zhang, Liusheng Duan, Weiming Tan

**Affiliations:** 1 Engineering Research Centre of Plant Growth Regulators, Ministry of Education, College of Agronomy and Biotechnology, China Agricultural University, Beijing 100193, China; tianhao2013@cau.edu.cn (H.T.); liusj9112@126.com (S.L.); jindingsha@163.com (D.J.); duanlsh@cau.edu.cn (L.D.); 2 Department of Applied Chemistry, College of Science, China Agricultural University, Beijing 100193, China; ryandota@sina.com (Y.X.); zhangjianjun@cau.edu.cn (J.Z.)

**Keywords:** gibberellin derivatives, natural products, Huisgen reaction, amide, plant growth regulators

## Abstract

A series of novel C-3-OH substituted gibberellin derivatives bearing an amide group were designed and synthesized from the natural product gibberellic acid (GA_3_). Their activities on the plant growth regulation of rice and *Arabidopsis* were evaluated in vivo. Among these compounds, **10d** and **10f** exhibited appreciable inhibitory activities on rice (48.6% at 100 μmol/L) and *Arabidopsis* (41.4% at 100 μmol/L), respectively. These results provide new insights into the design and synthesis of potential plant growth regulators.

## 1. Introduction

**Figure 1:**
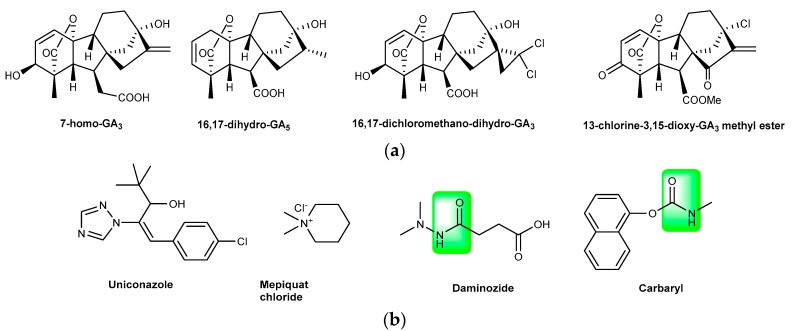
Gibberellic acid (GA_3_) derivatives (**a**) and commercially available synthetic plant growth regulators (PGRs) (**b**).

Plant growth regulators (PGRs) are organic compounds that can promote, inhibit or modify physiological processes in plants [1]. Various synthetic PGRs, such as uniconazole, mepiquat chloride, and daminozide (Figure 1) have been widely used to regulate plant growth and ensure ultimate harvesting. Although these compounds are effective, there have been increasing concerns from consumers’ point of view regarding the potential harmful side effects of these chemical agents. This, in turn, has led people to pay more attention to products of natural origin, potentially presenting less likely adverse side effects.

**Figure 2:**
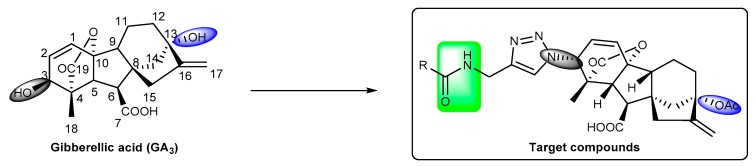
The molecular design of target compounds.

Gibberellins (GAs) are a large family of natural products that regulate many developmental processes in plants, including seed germination, stem elongation, and induction of flowering [2]. Over the years, more than a hundred GAs have been identified from organisms, amongst which only few compounds are available in large quantities such as the relatively cheap natural derived phytohormone named gibberellic acid (GA_3_) (Figure 2). The latter can be obtained by fermentation of the fungus *Gibberella fujikuroi* [3]. Moreover, GA_3_ presents an ideal molecule platform for chemical modifications, as it is a tetracyclic diterpenoid compound containing two hydroxyl groups, a lactone ring, and a carboxyl group. Besides, GA_3_ derivatives featuring diverse structures could exhibit different biological functions from parent molecule GA_3_ [4,5]. For example, 7-homo-GA_3_ and 16,17-dihydro-GA_5_ inhibited stem elongation, meanwhile 16,17-dichloromethano-dihydro-GA_3_ had an inhibitory effect on both stem elongation and flowering in *Lolium temulentum* [6,7]. In addition, some gibberellin derivatives characterized by an α,β-unsaturated ketone moiety even exhibited anti-tumor bioactivities [8,9]. However, other chemical modifications could be undertaken on the GA_3_ that could lead to new relevant biological effects.

Many PGRs regulate the growth of plants and contain an amide group, such as daminozide and carbaryl. Taking into consideration the chemical group, we believed that modified GA_3_ derivatives characterized by amide groups could provide interesting bioactivities to the target compounds. As part of our research in the field of PGRs [10,11,12,13], we report herein the synthesis of a series of novel C3-OH substituted GA_3_ derivatives bearing an amide moiety. To reach this aim, we applied a Huisgen ‘click’ cycloaddition [14] because it proceeds via the formation of a highly proteolytic and metabolic stable intermediate presenting a triazole moiety [15]. Besides, Mander and co-workers reported that the addition of an acetate group at the C13 position of 16,17-dihydro-GA_5_ increased the inhibition efficacy of the molecule towards graminaceous species [16]. Following this interesting result, we also acetylated the hydroxyl group at the C13 position of our target compounds. Fifteen new gibberellin derivatives were synthesized, and their inhibitory activities towards rice (*Oryza sativa* L.) and *Arabidopsis* were evaluated.

## 2. Results and Discussion

### 2.1. Synthesis

**Scheme 1:**
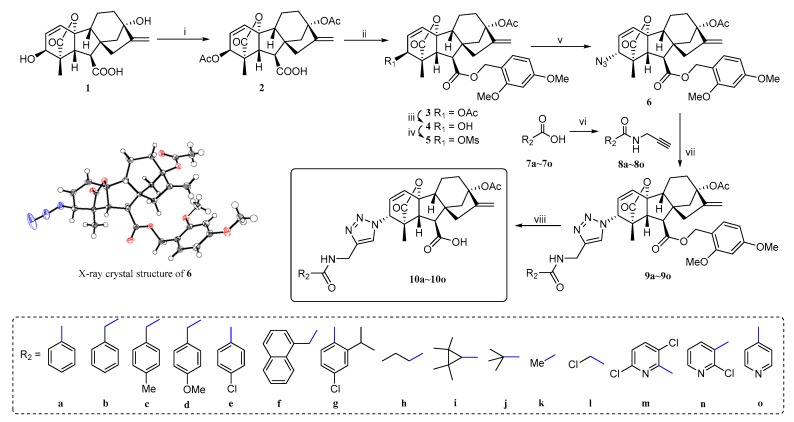
Total synthesis of the target compounds. *Reagents and conditions*: (i) Ac_2_O, DMAP, Py, r.t., 24 h, yield 98%; (ii) 2,4-dimethoxybenzyl alcohol, EDCI, DMAP, CH_2_Cl_2_, 0 °C, r.t., 3 h, yield 85%; (iii) K_2_CO_3_, MeOH:H_2_O at a ratio of 4:1 vt, pH 9~10, 0.5 h, yield 83%; (iv) MsCl, Py, 0 °C, r.t., 3 h, yield 95%; (v) NaN_3_, Dimethylformamide (DMF), 60 °C, 3 h, yield 89%; (vi) a. oxalyl chloride, DMF, CH_2_Cl_2_, 3 h; b. propargylamine, triethylamine (Et_3_N), CH_2_Cl_2_, 3 h, yield 82–99%; (vii) NaAsc, CuSO_4_·5H_2_O, MeOH/CH_2_Cl_2_/H_2_O = 1/8/1, 24 h, yield 72–88%; (viii) ZnCl_2_, CH_2_Cl_2_, 0.5 h, yield 71–90%.

Firstly, all the targeted compounds were prepared starting by the synthesis of the precursor, 3α-azido-13-acetate-7-(2,4-dimethoxybenzyl) ester-GA_3_ **6** (Scheme 1). GA_3_ (**1**) was peracetylated [17] in the presence of dimethylaminopyridine (DMAP), acetic anhydride in pyridine afforded 3,13-diacetylated GA_3_ (**2**). Then, compound **2** was treated with 2,4-dimethoxybenzyl alcohol in dichloromethane at 0 °C in the presence of 1-Ethyl-3-(3-dimethylaminopropyl)-carbodiimide hydrochloride (EDCI) and DMAP, producing the 3,13-diacetates-GA_3_ ester **3** in high yield [18]. Subsequently, the selective deacetylation [19,20] of **3** with potassium carbonate in methanol afforded the 3-hydroxyl-13-acetate-GA_3_ ester **4**. At this point of the synthesis, the structure of compound **4** was confirmed by comparing the ^1^H-NMR spectra of compounds **3** with **4**. We observed an up-field shift of the signal assigned to H-3 (δ 5.36 ppm in **3** vs. δ 4.16 ppm in **4**), confirming the conversion of compound **3** into **4**. Methyl sulfonylation of **4** with mesyl chloride in pyridine provided the 3-mesyl-13-acetate-GA_3_ ester **5**, which was subjected to a nucleophilic attack with sodium azide in *N*,*N*-Dimethylformamide (DMF) to result in the expected SN_2_ product **6**. The comparison of the ^1^H-NMR spectra of compounds **5** and **6** indicated that the characteristic signal of H-3 shifted to the up-field region after nucleophilic substitution (δ 5.07 ppm in **5** vs. δ 4.05 ppm in **6**). The obtention of a single crystal of **6** (Scheme 1) permitted the elucidation of the full configuration by X-ray diffraction.

Secondly, fifteen different carboxylic acids containing phenylic, aliphatic, and pyridyl groups (**7a**–**7o**) were selected as the initial materials to perform acyl chlorination [21] with oxalyl chloride and DMF in dichloromethane (Scheme 1). The acyl chloride substrates were subjected to amidation with mono-propargylamine and triethylamine in dichloromethane to afford the alkynyl carboxamides (**8a**–**8o**). Then, the expected target compounds presenting the protection groups (**9a**–**9o**) were formed by the fusion of a specific azide intermediate **6** with the terminal alkyne group of the corresponding alkynyl carboxamide (**8a**–**8o**) via a Huisgen ‘click’ cycloaddition [14,22]. Finally, the protecting groups were selectively removed following typical procedures from literature [23,24]. Shortly, the 2,4-dimethoxybenzyl esters could be readily oxidized by 2,3-dichloro-5,6-dicyano benzoquinone (DDQ) to generate the corresponding carboxylic acids. Unexpectedly, we did not obtain the target compounds (**10a**–**10o**) according to this method. Indeed, this specific carboxylic acid is prone to a different acidic character when compared to a simple carboxylic acid. Moreover, the funtional groups in the ring A of GA_3_ were quite sensitive under many reaction conditions [4,7,25]. In order to overcome the difficulty described above, a series of thorough experiments were carried out. Fortunately, we found that zinc dichloride used as a catalyst could cleave the 2,4-dimethoxylbenzyl esters (**9a**–**9o**) to successfully obtain the corresponding carboxylic acids (**10a**–**10o**) without affecting other sensitive functional groups such as lactone, acetyl, amide, or triazole group. This method offered a practical protective strategy for complex structures. The molecular structure of the intermediates and target compounds was confirmed by ^1^H- and ^13^C-NMR spectroscopy and High-resolution mass spectra (HRMS).

### 2.2. Biological Activities towards Arabidopsis and Rice

To examine whether the fifteen prepared synthetic GA_3_ derivatives (**10a**–**10o**) could trigger a dwarf or elongated phenotype in *Arabidopsis*, the hypocotyl length of *Arabidopsis* seedlings and the second leaf sheath length of rice were used as the activity indicators [26,27], respectively.

The in vivo activities of the synthesized GA_3_ derivatives (**10a**–**10o**) obtained from the measurements of the hypocotyl lengths and the second leaf sheath are summarized in Table 1. As expected, compound **10f** exhibited the highest inhibitory activities (41.4%) on *Arabidopsis*. In contrast, the rice bioassays resulted in a higher number of compounds presenting more remarkable inhibitory activity than for *Arabidopsis*. Compound **10d** had the best results with an inhibition rate of 48.6% on rice, different from the results obtained for *Arabidopsis* (**10f**). Therefore, we observed a discrepancy in the inhibition activity of the GA_3_ derivatives towards these two plants. The discrepancy in the inhibition activity of the GA_3_ derivatives towards the two different plants could come from the fact that the plants are complex organisms, in which the enzymes involved in the growth of *Arabidopsis* and rice are still not yet fully identified. On the whole, the molecular size of the substituent group could play an important role in the inhibition activities towards *Arabidopsis*. In rice, the benzene ring presenting more electron donating groups and less aliphatic hydrocarbons should present more inhibition activities. In addition, the moderate activities suggested a new direction for the development of these compounds to be used as new plant-type regulators that could specifically target crops and ornamental plants in the agricultural industry.

## 3. Materials and Methods

### 3.1. General Information

Solvents were purified according to standard procedure. All commercially available reagents were used without further purification. All the solvents were purchased at Sinopharm, Shanghai, China. The following compounds GA_3_; dimathylaminopyridine (DMAP); acetic anhydride; anhydrous pyridine; hydrochloric acid; dichloromethane; anhydrous sodium sulfate; ethyl acetate; light petroleum; 1-Ethyl-3-(3-dimethylaminopropyl)carbodiimide (EDCI); 2,4-dimethoxybenzyl alcohol; methanol; methylsulfonyl chloride; oxalyl chloride; propargylamine; sodium sulfate; sodium ascorbate; chalcanthite; and celite were purchased at Coupling technology co., LTD, Beijing, China. The seeds of *Arabidopsis* and rice were provided by the department of breeding and seed engineering of the China Agriculture University, Beijing.

All reactions were monitored by TLC or by iodine fuming detection. Column chromatography was conducted on a silica gel plug (200–300 mesh) using a mixture of ethyl actetate (EtOAc) and petroleum ether (b.p. 60–90 °C) as the eluent. The ^1^H- and ^13^C-NMR spectra were recorded on Bruker DPX300 (300 MHz and 75 MHz) and Bruker AVANCE 600 spectrometers with samples dissolved in deuterated chloroform (CDCl_3_) or deuterated dimethyl sulfoxide (DMSO-*d*_6_). The internal reference was tetramethyl silane TMS (δ = 0.000 ppm for ^1^H and ^13^C). High-resolution mass spectra (HRMS) were performed at the Peking University acquired with an Agilent 6520 Accurate-Mass-Q-TOF LC/MS system equipped with Electrospray ionization (ESI) source in the positive ionization mode. Solutions were concentrated at a temperature <60 °C under diminished pressure.

### 3.2. Chemical Synthesis

*Synthesis of 3,13-Acetoxy-ent-10β-hydroxy-20-norgibberella-1,16-diene-7,19-dioic acid-19,10-lactone* (**2**). A mixture of compound **1** [4] (50 g, 144.4 mmol), acetic anhydride (150 mL), and DMAP (0.18 g, 1.4 mmol) in anhydrous pyridine (200 mL) was stirred at room temperature overnight. The solution was diluted with water (50 mL) under an ice bath then acidified until reaching a pH value of 2 with diluted hydrochloric acid and finally extracted with dichloromethane (3 × 150 mL). The organic layers were pooled and dried over anhydrous sodium sulfate. The solvent was evaporated under reduced pressure and the residue was recrystallized in a 50% vt solution of ethyl acetate and light petroleum to give GA_3_ 3,13-diacetate (**2**). The analytical results of compound **2** were identical to the one described in the literature [28]. White solid, yield 98%, ^1^H-NMR (300 MHz, CDCl_3_): δ 6.39 (d, *J* = 9.3 Hz, 1 H), 5.90 (dd, *J* = 9.3, 3.8 Hz, 1 H), 5.36 (d, *J* = 3.8 Hz, 1 H), 5.20 (d, *J* = 1.7 Hz, 1 H), 5.04 (s, 1 H), 3.31 (d, *J* = 11.0 Hz, 1 H), 2.84 (d, *J* = 11.0 Hz, 1 H), 2.51–2.20 (m, 6 H), 2.15 (s, 3 H), 2.05 (s, 3 H), 2.01–1.81 (m, 3 H), 1.22 (s, 3 H).

*Synthesis of 3,13-Acetoxy-ent-10β-hydroxy-20-norgibberella-1,16-diene-7,19-dioic acid-7-(2,4-dimethoxybenzyl) ester-19,10-lactone* (**3**). To a solution of **2** (50 g, 116.2 mmol) dissolved in dichloromethane (300 mL), EDCI (33.4 g, 174.3 mmol) was slowly added followed by the addition of DMAP (0.14 g, 1.7 mmol) at 0 °C. After stirring at r.t. for 15 min, a solution of 2,4-dimethoxybenzyl alcohol (19.54 g, 116.2 mmol) dissolved in 80 mL of dichloromethane was added dropwise at 0 °C. After stirring the mixture at r.t. for 3 h, the reaction mixture was washed with diluted hydrochloric acid (1 M, 100 mL) and extracted with dichloromethane (3 × 150 mL). The combined organic layers were pooled and washed with a solution of saturated brine, then dried over anhydrous sodium sulfate followed by solvent evaporation under reduced pressure. The product was recrystallized in methanol to give **3**. White solid, yield 85%, m.p. 156–157 °C. ^1^H-NMR (300 MHz, CDCl_3_): δ 7.23 (d, *J* = 8.5 Hz, 1 H), 6.47–6.43 (m, 2 H), 6.35 (dd, *J* = 9.3, 0.6 Hz, 1 H), 5.85 (dd, *J* = 9.3, 3.8 Hz, 1 H), 5.31 (d, *J* = 3.8 Hz, 1 H), 5.19–5.18 (m, 1 H), 5.16–5.07 (m, 2 H), 4.94 (s, 1 H), 3.80 (s, 3 H), 3.77 (s, 3 H), 3.31 (d, *J* = 11.0 Hz, 1 H), 2.77 (d, *J* = 11.0 Hz, 1 H), 2.44–2.11 (m, 5 H), 2.09 (s, 3 H), 2.01 (s, 3 H), 1.96–1.62 (m, 4 H), 1.13 (s, 3 H). ^13^C-NMR(75 MHz, CDCl_3_): δ 176.68, 171.36, 169.63, 169.26, 161.26, 158.71, 153.46, 133.96, 131.47, 128.72, 115.59, 107.77, 103.63, 98.11, 89.61, 83.78, 69.87, 62.53, 55.03, 54.97, 52.96, 51.77, 50.71, 50.09, 41.86, 39.72, 35.63, 21.60, 20.39, 16.54, 13.88. HRMS for C_32_H_40_NO_10_ (M + NH_4_)^+^ 598.2647. Found: 598.2648.

*Synthesis of 13-Acetoxy-ent-3α,10β-dihydroxy-20-norgibb-erella-1,16-diene-7,19-dioic acid-7-(2,4-dimethoxybenzyl) ester-19,10-lactone* (**4**). To a solution of compound **3** (40 g, 68.9 mmol) dissolved in methanol (300 mL) was slowly added a saturated potassium carbonate solution dropwise until the pH value of the mixture reached 9–10. Then, the solution was stirred at r.t. for 40 min. The reaction mixture was acidified until reaching a pH value of 7 with a hydrochloric acid solution (1 M). The reaction mixture was washed with water (100 mL) and then with a solution of saturated brine (150 mL) after extraction with dichoromethane (3 × 100 mL) to yield **4** without further purification. White solid, yield 83%, m.p. 125–126 °C. ^1^H-NMR (300 MHz, CDCl_3_): δ 7.27 (d, *J* = 8.2 Hz, 1 H), 6.50–6.47 (m, 2 H), 6.33 (d, *J* = 9.4 Hz, 1 H), 5.93 (dd, *J* = 9.3, 3.7 Hz, 1 H), 5.21 (s, 1 H), 5.19–5.10 (m, 2 H), 4.98 (s, 1 H), 3.84 (s, 3 H), 3.81 (s, 3 H), 3.23 (d, *J* = 10.9 Hz, 1 H), 2.82 (d, *J* = 10.9 Hz, 1 H), 2.47–2.09 (m, 6 H), 2.04 (s, 3 H), 1.98–1.74 (m, 3 H), 1.25 (s, 3 H). ^13^C-NMR (75 MHz, CDCl_3_): δ 178.24, 171.80, 169.42, 161.23, 158.71, 153.50, 132.30, 131.48, 115.61, 107.69, 103.63, 98.12, 90.03, 83.92, 69.35, 62.61, 55.05, 54.98, 53.16, 52.35, 50.85, 50.77, 41.89, 39.80, 35.68, 21.63, 16.58, 14.00. HRMS for C_30_H_38_NO_9_ (M + NH_4_)^+^ 556.2541. Found: 556.2546.

*Synthesis of 3α-Methylsulfonyl-13-acetoxy-ent-10β-hydroxy-20-norgibberella-1,16-diene-7,19-dioic acid-7-(2,4-dimethoxybenzyl) ester-19,10-lactone* (**5**). To a solution of compound **4** (30 g, 55.7 mmol) dissolved in anhydrous pyridine (150 mL) was slowly added a solution of methylsulfonyl chloride (6.38 g, 61.27 mmol) dissolved in dichloromethane dropwise at 0 °C. After stirring the reaction mixture at r.t. for 3 h, the mixture was washed with a solution of diluted hydrochloric acid (2 M, 500 mL) followed by extractions with dichoromethane (3 × 150 mL) to yield **5** without further purification. White solid, yield 95%, m.p. 80–82 °C. ^1^H-NMR (300 MHz, CDCl_3_): δ 7.26 (d, *J* = 8.2 Hz, 1 H), 6.50 (s, 1 H), 6.47 (s, 2 H), 6.02 (dd, *J* = 9.3, 3.8 Hz, 1 H), 5.22 (d, *J* = 1.4 Hz, 1 H), 5.19–5.10 (m, 2 H), 5.07 (d, *J* = 3.8 Hz, 1 H), 4.98 (s, 1 H), 3.84 (s, 3 H), 3.81 (s, 3 H), 3.32 (d, *J* = 11.0 Hz, 1 H), 3.08 (s, 3 H), 2.80 (d, *J* = 11.0 Hz, 1 H), 2.47–2.09 (m, 5 H), 2.04 (s, 3 H), 2.01–1.72 (m, 4 H), 1.28 (s, 3 H). ^13^C-NMR (75 MHz, CDCl_3_) δ 175.66, 171.01, 169.32, 161.29, 158.75, 153.25, 135.40, 131.55, 128.16, 115.52, 107.93, 103.65, 98.12, 89.47, 83.68, 62.62, 55.05, 55.00, 52.75, 52.28, 50.62, 50.54, 50.03, 41.76, 39.76, 38.37, 35.52, 21.60, 16.51, 14.44. HRMS for C_31_H_37_O_11_S (M + H)^+^ 617.2051. Found: 617.2052.

*Synthesis of 3α-Azido-13-acetoxy-ent-10β-hydroxy-20-norgibberella-1,16-diene-7,19-dioic acid-7-(2,4-dimethoxybenzyl) ester-19,10-lactone* (**6**). A solution containing compound **5** (30 g, 48.6 mmol) and NaN_3_ (9.49 g, 145.94 mmol) dissolved in anhydrous dimethyl formamide (150 mL) was stirred at r.t. for 3 h. The reaction mixture was poured into iced water (150 g) and then extracted with ethyl acetate (3 × 150 mL) to give a crude product **6**. The combined organic layers were concentrated under reduced pressure and followed by purification by silica gel chromatography to provide the intermediate **6**. White solid, yield 89%, m.p. 126–128 °C. ^1^H-NMR (300 MHz, CDCl_3_): δ 7.24 (d, *J* = 8.3 Hz, 1 H), 6.50–6.46 (m, 2 H), 6.41 (dd, *J* = 9.3, 2.0 Hz, 1 H), 5.92 (dd, *J* = 9.3, 2.6 Hz, 1 H), 5.20 (s, 1 H), 5.18–5.12 (m, 2 H), 4.97 (s, 1 H), 4.05 (t, *J* = 2.3 Hz, 1 H), 3.84 (s, 3 H), 3.80 (s, 3 H), 2.99 (d, *J* = 10.7 Hz, 1 H), 2.77 (d, *J* = 10.8 Hz, 1 H), 2.03 (s, 3 H), 1.29 (s, 3 H). ^13^C-NMR (75 MHz, CDCl_3_) δ: 174.64, 171.22, 169.23, 161.30, 158.72, 153.20, 132.90, 131.56, 127.98, 115.42, 107.78, 103.67, 98.07, 88.17, 83.69, 63.84, 62.68, 57.27, 55.02, 54.97, 52.75, 50.62, 50.47, 50.43, 41.79, 39.80, 35.58, 21.58, 16.56, 14.51. HRMS for C_30_H_37_N_4_O_8_ (M + NH_4_)^+^ 581.2606. Found: 581.2605.

X-ray crystal structure of compound **6**: CCDC 1541695 contains the supplementary crystallographic data for this paper. These data can be obtained free of charge via http://www.ccdc.cam.ac.uk/conts/retrieving.html (or from the CCDC, 12 Union Road, Cambridge CB2 1EZ, UK; Fax: +44 1223 336033; E-mail: deposit@ccdc.cam.ac.uk).

#### 3.2.1. Synthesis of *N*-Prop-2-ynylamide (**8a**–**8o**)

The compounds **8a**–**8o** were all synthesized by the following general procedure. To a solution containing **7a** (5 g, 40.94 mmol) and DMF (0.05 mL, 0.65 mmol) dissolved in dichloromethane (100 mL) was added a solution of oxalyl chloride (3.8 mL, 45.03 mmol) dissolved in dichloromethane (10 mL) dropwise at 0 °C. After stirring the reaction mixture at r.t. for 3 h, the solution was concentrated under reduced pressure to give the corresponding acyl chloride. To a solution composed of propargylamine (3.1 mL, 45.03 mmol) and triethylamine (8.5 mL, 61.41 mmol) dissolved in dichloromethane (100 mL) was added dropwise at 0 °C a solution of the corresponding acyl chloride previously dissolved in dichloromethane (30 mL). After stirring at r.t. for 3 h, the mixture was washed with a solution of diluted hydrochloric acid (1 M, 100 mL) followed by an extraction with dichloromethane (3 × 100 mL). The combined organic layers were then dried over anhydrous sodium sulfate and then concentrated under reduced pressure to afford the crude product **8a**. The residue was then crystallized with a mixture of ethyl acetate/petroleum ether to afford the pure product **8a**. The spectral data for the compounds **8a**–**8o** are given in the Supporting-Information.

#### 3.2.2. Synthesis of 3α-(4-Amido-methyl-1*H*-1,2,3-trizol-1-yl)-13-acetoxy-ent-10β-hydroxy-20-norgibberella-1,16-diene-7,19-dioic acid-7-(2,4-dimethoxybenzyl) ester-19,10-lactone (**9a**–**9o**)

The compounds **9a**–**9o** were all synthesized by the following general procedure. To a solution containing a mixture of the corresponding compound **8a** (0.47 g, 2.93 mmol) and **6** (1.5 g, 2.66 mmol) in dichloromethane (16 mL) was added slowly an aqueous solution (2 ml) of sodium ascorbate (5 mg) and chalcanthite (10 mg). Methanol (ca. 2 mL) was added to the reaction mixture under stirring until obtaining a homogeneous phase. Then, the solution was stirred at r.t. for 12 h. The crude mixture was filtrated through a short plug of celite and the solvents were evaporated under reduced pressure. The residue was purified by silica gel chromatography to provide the corresponding **9a**. The spectral data for the compounds **9a**–**9o** are given in the Supporting-Information.

#### 3.2.3. Synthesis of 3α-(4-Amido-methyl-1*H*-1,2,3-trizol-1-yl)-13-acetoxy-ent-10β-hydroxy-20-norgibberella-1,16-diene-7,19-dioic acid-19,10-lactone-7-carboxyl (**10a**–**10o**)

The compounds **10a**–**10o** were synthesized by the following general procedure. A solution containing the corresponding compound **9a** (1.5 g, 2.08 mmol) and zinc chloride (ZnCl_2_, 0.85 g, 6.24 mmol) dissolved in dichloromethane (20 mL) was stirred at r.t. for 1 h. The mixture was then filtered through a celite plug followed by solvent evaporation under reduced pressure. The residue was purified by silica gel chromatography to provide the corresponding target compound **10a**. The spectral data for the compounds **10a**–**10o** are given in the Supporting-Information.

### 3.3. Biological Activity Assay

The inhibitory activity of all the synthesized compounds was determined in vivo by evaluating the hypocotyl length of *Arabidopsis* (Columbia-0, Col-0) plants according to the following experimental procedures. The ½ Murashige–Skoog (MS) culture medium, a plant growth medium used in the laboratories for cultivation of plant cell culture, was prepared as follows: to 1 L of distilled water, MS (2.215 g), sucrose (10 g), and agar (8 g) were added, and the pH was adjusted to 5.9 by adding sodium hydroxide (5 M). The media were sterilized in a high-pressure steam sterilization pot. Seeds of *Arabidopsis* were sterilized in 70% ethanol for 1 min, with 1% sodium hypochlorite solution (*w*/*v*) for 15 min and then cleaned five times with distilled water. Seeds of *Arabidopsis* were grown on the ½ MS medium containing 100 μM of the respective synthetic GA_3_ derivate. Each medium was sowed with 15 seeds and then maintained at 4 °C for two consecutive days. The seedswere then incubated at 22 °C in a growth chamber containing 60% relative humidity. All the plants were maintained in the dark during the growth period. After 6 days, the length of each hypocotyl was measured using a ImageJ software (https://imagej.nih.gov/ij/index.html).

The inhibitory activities of all the synthesized compounds were determined in vivo by evaluating the length of the second leaf sheath of rice according to the following experimental procedure. Seeds of rice (*Oryza sativa* L.) cultivar *Nipponbare* were sterilized with 2% sodium hypochlorite solution (*w*/*v*) for 40 min and then soaked in distilled water for 24 h in darkness at 30 °C. The seeds were then washed with distilled water and incubated in distilled water at 30 °C until germination. When the coleoptiles were of ca. 2.0 mm in length after 2 days incubation, the 25 germinated seeds were planted in a 120 mm× 120 mm × 60 mm plastic box that was filled with 80 mL of 0.9% (*w*/*v*) agar and then placed in a growth chamber with a 16/8 h photoperiod, 30/24 °C day/night temperatures, 60% relative humidity, and 400 μmol^−2^ s^−1^ of photon flux density. Two days later, a 1 μL-drop of a solution containing 100 μM of the respective synthetic GA_3_ derivate was deposited at the gap between the coleoptile and the leaf sheath of each rice seedling. After three days, the length of the second leaf sheath of the seedlings was measured using a ruler.

The following equation was used to determine the inhibitory activity of all the synthesized compounds towards *Arabidopsis* and rice:

(1) $$I=\frac{{\bar{L}}_{0}-\bar{L}}{{\bar{L}}_{0}}\times 100\%$$

where *I* was the inhibition rate, ${\bar{L}}_{0}$ and $\bar{L}$ were the average lengths of the hypocotyl of *Arabidopsis* (or the second leaf sheath of rice) in the blank test and in the presence of the target compound, respectively.

## 4. Conclusions

In summary, a series of novel GA_3_ derivatives characterized by the amide group were synthesized by utilizing a Huisgen reaction to fuse the amide group moiety to the main chemical skeleton of the natural product GA_3_. Moreover, we established a novel protection method of the carboxyl groups despite the challenging presence of other highly functionalized chemical groups. The results of an in vivo assay suggested that some of these new compounds exhibited appreciable plant growth inhibitory activities. In particular, compounds **10d** and **10f** were the most effective towards rice and *Arabidopsis*, respectively. The discrepancy in the inhibitory activities towards the two plants could come from the inherently different biological mechanism of the two different plants. In the future, we will strive to study the detailed action of mechanism of these inhibitors.

## Figures and Tables

**Table 1:**
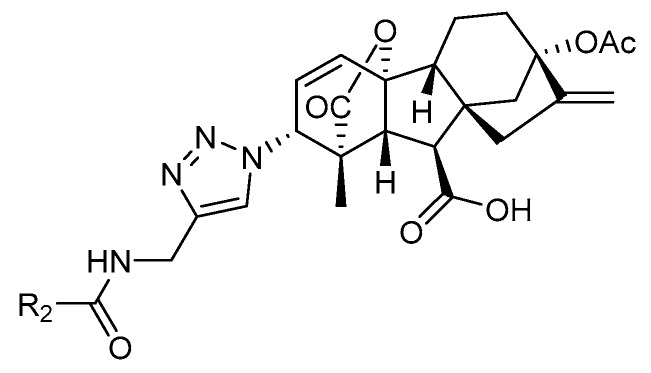
In vivo activities of the synthetic GA_3_ derivatives (100 µM).

Compound No.	Length (cm) ^a^	
	*Arabidopsis*	Rice
**10a**	1.48 ± 0.27	1.89 ± 0.36
**10b**	1.40 ± 0.14	2.16 ± 0.38
**10c**	1.54 ± 0.20	1.83 ± 0.27 *
**10d**	1.55 ± 0.20	1.47 ± 0.20 *
**10e**	1.39 ± 0.17	1.98 ± 0.35
**10f**	0.88 ± 0.15 *	2.13 ± 0.32
**10g**	1.06 ± 0.15	2.01 ± 0.30
**10h**	1.54 ± 0.25	2.43 ± 0.39
**10i**	1.43 ± 0.20	2.72 ± 0.28
**10j**	1.55 ± 0.23	2.26 ± 0.28
**10k**	1.53 ± 0.25	2.61 ± 0.21
**10l**	1.37 ± 0.20	2.22 ± 0.24
**10m**	1.43 ± 0.20	2.12 ± 0.51
**10n**	1.05 ± 0.18	2.04 ± 0.41
**10o**	1.43 ± 0.14	2.49 ± 0.29
**Blank control**	1.50 ± 0.21	2.86 ± 0.36
**Uniconazole**	0.12 ± 0.03 **	1.07 ± 0.11 **
**GA_3_**	1.77 ± 0.22 *	5.40 ± 0.48 **

* *p* < 0.05, ** *p* < 0.01, ^a^ Data are mean values ± the standard deviation obtained from three independent experiments.

## Supplementary Materials

Supplementary materials are available online.

## References

[1] Tan W.M., Hou N., Pang S., Zhu X.F., Li Z.H., Wen L.X., Duan L.S. (2012). Improved biological effects of uniconazole using porous hollow silica nanoparticles as carriers. Pest Manag. Sci..

[2] Shani E., Weinstain R., Zhang Y., Castillejo C., Kaiserli E., Chory J., Tsien R.Y., Estelle M. (2013). Gibberellins accumulate in the elongating endodermal cells of arabidopsis root. Proc. Natl. Acad. Sci. USA.

[3] Phuoc L.T., Mander L.N., Koshioka M., Oyama-Okubo N., Nakayama M., Ito A. (2008). Confirmation of structure and synthesis of three new 11β-OH C_20_ gibberellins from loquat fruit. Tetrahedron.

[4] Mander L.N. (2003). Twenty years of gibberellin researchelectronic supplementary information (ESI) available: Functionality patterns for naturally occurring gibberellins (gan). See http://www.Rsc.Org/suppdata/np/b0/b007744p. Nat. Prod. Rep..

[5] Murase K., Hirano Y., Sun T.P., Hakoshima T. (2008). Gibberellin-induced della recognition by the gibberellin receptor gid1. Nature.

[6] Bergner C., Lischewski M., Adam G., Sembdner G. (1982). Biological activity of gibberellin analogues. Planta.

[7] Mander L.N., Sherburn M., Camp D., King R.W., Evans L.T., Pharis R.P. (1998). Effects of D-ring modified gibberellins on flowering and growth in *Lolium temulentum*. Phytochemistry.

[8] Chen J., Sun Z., Zhang Y., Zeng X., Qing C., Liu J., Li L., Zhang H. (2009). Synthesis of gibberellin derivatives with anti-tumor bioactivities. Bioorg. Med. Chem. Lett..

[9] Zhang Y., Zhang H., Chen J., Zhao H., Zeng X., Zhang H., Qing C. (2012). Antitumor and antiangiogenic effects of ga-13315, a gibberellin derivative. Investig. New Drugs.

[10] Gao F., Hu T., Tan W., Yu C., Li Z., Zhang L., Duan L. (2016). Photoprotectant improves photostability and bioactivity of abscisic acid under uv radiation. J. Photochem. Photobiol. B-Biol..

[11] Zong G., Liang X., Zhang J., Duan L., Tan W., Wang D. (2014). Synthesis and plant growth regulation activity of α-d-Man*p*NAc-(1→2)-[α-l-Rha*p*-(1→3)-]α-l-Rha*p*-(1→4)-β-d-Glu*p*NAc-(1→3)-α-l-Rha*p*, the repeating unit of *O*-antigen of *Rhizobium trifolii 4s*. Carbohydr. Res..

[12] Zhu X., Tan W., Zhou F., Li Z., Duan L. (2012). The effect of phosphate buffer solutions on uniconazole complexation with hydroxypropyl-β-cyclodextrin and methyl-β-cyclodextrin. J. Incl. Phenom. Macrocycl. Chem..

[13] Zhou F., Ran J., Tan W., Li Z., Duan L. (2013). Synthesis and biological activity of aryamido-cyclopropanecarboxylic acid. Chin. J. Pestic. Sci..

[14] Meldal M., Tornoe C.W. (2008). Cu-catalyzed azide-alkyne cycloaddition. Chem. Rev..

[15] Pedersen D.S., Abell A. (2011). 1,2,3-Triazoles in peptidomimetic chemistry. Eur. J. Organ. Chem..

[16] Beck E.J., Twitchin B., Mander L.N. (2004). Radio labeling of the gibberellin plant growth inhibitor 16,17-dihydro-GA5. Can. J. Chem..

[17] Huigens R.W., Morrison K.C., Hicklin R.W., Flood T.A., Richter M.F., Hergenrother P.J. (2013). A ring-distortion strategy to construct stereochemically complex and structurally diverse compounds from natural products. Nat. Chem..

[18] Ghosh P., Aube J. (2011). Resolution of carboxylic acids using copper(I)-promoted removal of propargylic esters under neutral conditions. J. Organ. Chem..

[19] Macmillan J., Willis C.L. (1986). Partial synthesis of [1β,2β-^2^H_2_]-, [2β-^2^H]-, and [2α-^2^H]-Gibberellin-A1. J. Chem. Soc.-Perkin Trans. 1.

[20] Dolan S.C., Macmillan J. (1985). Partial synthesis of the 15β-hydroxygibberellins A_67_ and A_68_ and of 15β-hydroxygibberellins A_1_ and A_3_. J. Chem. Soc.-Perkin Trans. 1.

[21] Chalmers B.A., Xing H., Houston S., Clark C., Ghassabian S., Kuo A., Cao B., Reitsma A., Murray C.-E.P., Stok J.E. (2016). Validating eaton’s hypothesis: Cubane as a benzene bioisostere. Angew. Chem..

[22] Brase S., Gil C., Knepper K., Zimmermann V. (2005). Organic azides: An exploding diversity of a unique class of compounds. Angew. Chem..

[23] Kim C.U., Misco P.F. (1985). New oxidatively removable carboxy protecting groups. Tetrahedron Lett..

[24] Yoo S.E., Kim H.R., Yi K.Y. (1990). Oxidative debenzylation of 4-methoxy-α-methylbenzyl esters. Tetrahedron Lett..

[25] Kirkwood P.S., Macmillan J., Sinnott M.L. (1980). Rearrangement of the lactone ring of gibberellin-A_3_ in aqueous alkali—Participation of the ionized 3-hydroxy-group in an *anti S*_N_2′ reaction. J. Chem. Soc.-Perkin Trans. 1.

[26] Oh K., Yamada K., Asami T., Yoshizawa Y. (2012). Synthesis of novel brassinosteroid biosynthesis inhibitors based on the ketoconazole scaffold. Bioorg. Med. Chem. Lett..

[27] Nishijima T., Katsura N. (1989). A modified micro-drop bioassay using dwarf rice for detection of femtomol quantities of gibberellins. Plant Cell Physiol..

[28] Serebryakov E.P., Agnistikova V.N., Suslova L.M. (1984). Structure-activity study of gibberellins. Part 1. Growth-promoting activity of some selectively modified gibberellins. Phytochemistry.

