# Supplementary material for: Synthesis of Gibberellic Acid Derivatives and Their Effects on Plant Growth
Source: Molecules. 2017 Apr 26;22(5):694. doi: 10.3390/molecules22050694 (PMC6153925; doi:10.3390/molecules22050694)

Supporting Information:

# Synthesis of gibberellic acid derivatives and their effects on plant growth

**Hao Tian [1, 2], Yiren Xu [2], Shaojin Liu [1], Dingsha Jin [1], Jianjun Zhang [2], Liusheng Duan [1], and Weiming Tan [1,*]**

[1] Engineering Research Centre of Plant Growth Regulators, Ministry of Education, College of Agronomy and Biotechnology, China Agricultural University, Beijing 100193, PR China; tianhao2013@cau.edu.cn (H.T.); liusj9112@126.com (S.L.); jindingsha@163.com (D.J.); duanlsh@cau.edu.cn (L.D.)

[2] Department of Applied Chemistry, College of Science, China Agricultural University, Beijing 100193, PR China; ryandota@sina.com (Y.X.); 06001h@cau.edu.cn (J.Z.);

* Correspondence: tanwm@cau.edu.cn (W.T.); Tel.: +86 (0)10 62732039

---

## Table of Contents:

| Supporting Information | |
|---|---|
| **Contents** | Page |
| Spectral Data of **2~6** | S5 |
| Spectral Data of **8a~8o** | S6 |
| Spectral Data of **9a~9o** | S7 |
| Spectral Data of **10a~10o** | S10 |
| References and Notes | S13 |
| $^{1}$H-NMR spectrum of compound **2.** | S14 |
| $^{1}$H-NMR spectrum of compound **3.** | S14 |
| $^{13}$C-NMR spectrum of compound **3.** | S15 |
| HRMS of compound **3.** | S15 |
| $^{1}$H-NMR spectrum of compound **4.** | S16 |
| $^{13}$C-NMR spectrum of compound **4.** | S16 |
| HRMS of compound **4.** | S17 |
| $^{1}$H-NMR spectrum of compound **5.** | S17 |
| $^{13}$C-NMR spectrum of compound **5.** | S18 |
| HRMS of compound **5.** | S18 |
| $^{1}$H-NMR spectrum of compound **6.** | S19 |
| $^{13}$C-NMR spectrum of compound **6.** | S19 |
| HRMS of compound **6.** | S20 |
| $^{1}$H-NMR spectrum of compound **8c.** | S20 |
| $^{13}$C-NMR spectrum of compound **8c.** | S21 |
| HRMS of compound **8c.** | S21 |
| $^{1}$H-NMR spectrum of compound **8d.** | S22 |
| $^{13}$C-NMR spectrum of compound **8d.** | S22 |

| | |
|---|---|
| HRMS of compound **8d.** | S23 |
| $^{1}$H-NMR spectrum of compound **8f.** | S23 |
| $^{13}$C-NMR spectrum of compound **8f.** | S24 |
| HRMS of compound **8f.** | S24 |
| $^{1}$H-NMR spectrum of compound **8g.** | S25 |
| $^{13}$C-NMR spectrum of compound **8g.** | S25 |
| HRMS of compound **8g.** | S26 |
| $^{1}$H-NMR spectrum of compound **8i.** | S26 |
| $^{13}$C-NMR spectrum of compound **8i.** | S27 |
| HRMS of compound **8i.** | S27 |
| $^{1}$H-NMR spectrum of compound **8n.** | S28 |
| $^{13}$C-NMR spectrum of compound **8n.** | S28 |
| HRMS of compound **8n.** | S29 |
| $^{1}$H-NMR spectrum of compound **9a.** | S29 |
| $^{13}$C-NMR spectrum of compound **9a.** | S30 |
| HRMS of compound **9a.** | S30 |
| $^{1}$H-NMR spectrum of compound **9b.** | S31 |
| $^{13}$C-NMR spectrum of compound **9b.** | S31 |
| HRMS of compound **9b.** | S32 |
| $^{1}$H-NMR spectrum of compound **9c.** | S32 |
| $^{13}$C-NMR spectrum of compound **9c.** | S33 |
| HRMS of compound **9c.** | S33 |
| $^{1}$H-NMR spectrum of compound **9d.** | S34 |
| $^{13}$C-NMR spectrum of compound **9d.** | S34 |
| HRMS of compound **9d.** | S35 |
| $^{1}$H-NMR spectrum of compound **9e.** | S35 |
| $^{13}$C-NMR spectrum of compound **9e.** | S36 |
| HRMS of compound **9e.** | S36 |
| $^{1}$H-NMR spectrum of compound **9f.** | S37 |
| $^{13}$C-NMR spectrum of compound **9f.** | S37 |
| HRMS of compound **9f.** | S38 |
| $^{1}$H-NMR spectrum of compound **9g.** | S38 |
| $^{13}$C-NMR spectrum of compound **9g.** | S39 |
| HRMS of compound **9g.** | S39 |
| $^{1}$H-NMR spectrum of compound **9h.** | S40 |
| $^{13}$C-NMR spectrum of compound **9h.** | S40 |
| HRMS of compound **9h.** | S41 |
| $^{1}$H-NMR spectrum of compound **9i.** | S41 |
| $^{13}$C-NMR spectrum of compound **9i.** | S42 |
| HRMS of compound **9i.** | S42 |
| $^{1}$H-NMR spectrum of compound **9j.** | S43 |
| $^{13}$C-NMR spectrum of compound **9j.** | S43 |
| HRMS of compound **9j.** | S44 |

| | |
|---|---|
| $^{1}$H-NMR spectrum of compound **9k.** | S44 |
| $^{13}$C-NMR spectrum of compound **9k.** | S45 |
| HRMS of compound **9k.** | S45 |
| $^{1}$H-NMR spectrum of compound **9l.** | S46 |
| $^{13}$C-NMR spectrum of compound **9l.** | S46 |
| HRMS of compound **9l.** | S47 |
| $^{1}$H-NMR spectrum of compound **9m.** | S47 |
| $^{13}$C-NMR spectrum of compound **9m.** | S48 |
| HRMS of compound **9m.** | S48 |
| $^{1}$H-NMR spectrum of compound **9n.** | S49 |
| $^{13}$C-NMR spectrum of compound **9n.** | S49 |
| HRMS of compound **9n.** | S50 |
| $^{1}$H-NMR spectrum of compound **9o.** | S50 |
| $^{13}$C-NMR spectrum of compound **9o.** | S51 |
| HRMS of compound **9o.** | S51 |
| $^{1}$H-NMR spectrum of compound **10a.** | S52 |
| $^{13}$C-NMR spectrum of compound **10a.** | S52 |
| HRMS of compound **10a.** | S53 |
| $^{1}$H-NMR spectrum of compound **10b.** | S53 |
| $^{13}$C-NMR spectrum of compound **10b.** | S54 |
| HRMS of compound **10b.** | S54 |
| $^{1}$H-NMR spectrum of compound **10c.** | S55 |
| $^{13}$C-NMR spectrum of compound **10c.** | S55 |
| HRMS of compound **10c.** | S56 |
| $^{1}$H-NMR spectrum of compound **10d.** | S56 |
| $^{13}$C-NMR spectrum of compound **10d.** | S57 |
| HRMS of compound **10d.** | S57 |
| $^{1}$H-NMR spectrum of compound **10e.** | S58 |
| $^{13}$C-NMR spectrum of compound **10e.** | S58 |
| HRMS of compound **10e.** | S59 |
| $^{1}$H-NMR spectrum of compound **10f.** | S59 |
| $^{13}$C-NMR spectrum of compound **10f.** | S60 |
| HRMS of compound **10f.** | S60 |
| $^{1}$H-NMR spectrum of compound **10g.** | S61 |
| $^{13}$C-NMR spectrum of compound **10g.** | S61 |
| HRMS of compound **10g.** | S62 |
| $^{1}$H-NMR spectrum of compound **10h.** | S62 |
| $^{13}$C-NMR spectrum of compound **10h.** | S63 |
| HRMS of compound **10h.** | S63 |
| $^{1}$H-NMR spectrum of compound **10i.** | S64 |
| $^{13}$C-NMR spectrum of compound **10i.** | S64 |
| HRMS of compound **10i.** | S65 |
| $^{1}$H-NMR spectrum of compound **10j.** | S65 |

| | |
|---|---|
| $^{13}$C-NMR spectrum of compound **10j.** | S66 |
| HRMS of compound **10j.** | S66 |
| $^{1}$H-NMR spectrum of compound **10k.** | S67 |
| $^{13}$C-NMR spectrum of compound **10k.** | S67 |
| HRMS of compound **10k.** | S68 |
| $^{1}$H-NMR spectrum of compound **10l.** | S68 |
| $^{13}$C-NMR spectrum of compound **10l.** | S69 |
| HRMS of compound **10l.** | S69 |
| $^{1}$H-NMR spectrum of compound **10m.** | S70 |
| $^{13}$C-NMR spectrum of compound **10m.** | S70 |
| HRMS of compound **10m.** | S71 |
| $^{1}$H-NMR spectrum of compound **10n.** | S71 |
| $^{13}$C-NMR spectrum of compound **10n.** | S72 |
| HRMS of compound **10n.** | S72 |
| $^{1}$H-NMR spectrum of compound **10o.** | S73 |
| $^{13}$C-NMR spectrum of compound **10o.** | S73 |
| HRMS of compound **10o.** | S74 |

**Spectral Data of 2~6:**

***3,13-acetoxy-ent-10β-hydroxy-20-norgibberella-1,16-diene-7,19-dioic acid-19,10-lactone*** **(2):** White solid, yield 98%, $^{1}$H-NMR (300 MHz, $CDCl_3$): δ 6.39 (d, J = 9.3 Hz, 1 H), 5.90 (dd, J = 9.3, 3.8 Hz, 1 H), 5.36 (d, J = 3.8 Hz, 1 H), 5.20 (d, J = 1.7 Hz, 1 H), 5.04 (s, 1 H), 3.31 (d, J = 11.0 Hz, 1 H), 2.84 (d, J = 11.0 Hz, 1 H), 2.51 – 2.20 (m, 6 H), 2.15 (s, 3 H), 2.05 (s, 3 H), 2.01 - 1.81 (m, 3 H), 1.22 (s, 3 H). The analytical results of compound **2** was identical to the one described in the literature [1].

***3,13-acetoxy-ent-10β-hydroxy-20-norgibberella-1,16-diene-7,19-dioic acid-7-(2,4-dimethoxybenzyl) ester-19,10-lactone*** (**3**): White solid, yield 85%, m.p. 156-157 °C. $^{1}$H-NMR (300 MHz, $CDCl_3$): δ 7.23 (d, J = 8.5 Hz, 1 H), 6.47 - 6.43 (m, 2 H), 6.35 (dd, J = 9.3, 0.6 Hz, 1 H), 5.85 (dd, J = 9.3, 3.8 Hz, 1 H), 5.31 (d, J = 3.8 Hz, 1 H), 5.19 - 5.18 (m, 1 H), 5.16 – 5.07 (m, 2 H), 4.94 (s, 1 H), 3.80 (s, 3 H), 3.77 (s, 3 H), 3.31 (d, J = 11.0 Hz, 1 H), 2.77 (d, J = 11.0 Hz, 1 H), 2.44 - 2.11 (m, 5 H), 2.09 (s, 3 H), 2.01 (s, 3 H), 1.96 – 1.62 (m, 4 H), 1.13 (s, 3 H). $^{13}$C-NMR(75 MHz, $CDCl_3$): δ 176.68, 171.36, 169.63, 169.26, 161.26, 158.71, 153.46, 133.96, 131.47, 128.72, 115.59, 107.77, 103.63, 98.11, 89.61, 83.78, 69.87, 62.53, 55.03, 54.97, 52.96, 51.77, 50.71, 50.09, 41.86, 39.72, 35.63, 21.60, 20.39, 16.54, 13.88. HRMS for $C_{32}H_{40}NO_{10}$ $(M+NH_4)^+$ 598.2647. Found: 598.2648.

***13-acetoxy-ent-3α,10β-dihydroxy-20-norgibb- erella-1,16-diene-7,19-dioic acid-7-(2,4-dimethoxybenzyl) ester-19,10-lactone*** (**4**): White solid, yield 83%, m.p. 125-126 °C. $^{1}$H-NMR (300 MHz, $CDCl_3$): δ 7.27 (d, J = 8.2 Hz, 1 H), 6.50 – 6.47 (m, 2 H), 6.33 (d, J = 9.4 Hz, 1 H), 5.93 (dd, J = 9.3, 3.7 Hz, 1 H), 5.21 (s, 1 H), 5.19 – 5.10 (m, 2 H), 4.98 (s, 1 H), 3.84 (s, 3 H), 3.81 (s, 3 H), 3.23 (d, J = 10.9 Hz, 1 H), 2.82 (d, J = 10.9 Hz, 1 H), 2.47 - 2.09 (m, 6 H), 2.04 (s, 3 H), 1.98 - 1.74 (m, 3 H), 1.25 (s, 3 H). $^{13}$C-NMR (75 MHz, $CDCl_3$): δ 178.24, 171.80, 169.42, 161.23, 158.71, 153.50, 132.30, 131.48, 115.61, 107.69, 103.63, 98.12, 90.03, 83.92, 69.35, 62.61, 55.05, 54.98, 53.16, 52.35, 50.85, 50.77, 41.89, 39.80, 35.68, 21.63, 16.58, 14.00. HRMS for $C_{30}H_{38}NO_9$ $(M+NH_4)^+$ 556.2541. Found: 556.2546.

***3β-methylsulfonyl-13-acetoxy-ent-10β-hydroxy-20-norgibberella-1,16-diene-7,19-dioic acid-7-(2,4-dimethoxybenzyl) ester-19,10-lactone*** (**5**): White solid, yield 95%, m.p. 80-82 °C. $^{1}$H-NMR (300 MHz, $CDCl_3$): δ 7.26 (d, J = 8.2 Hz, 1 H), 6.50 (s, 1 H), 6.47 (s, 2 H), 6.02 (dd, J = 9.3, 3.8 Hz, 1 H), 5.22 (d, J = 1.4 Hz, 1 H), 5.19 - 5.10 (m, 2 H), 5.07 (d, J = 3.8 Hz, 1 H), 4.98 (s, 1 H), 3.84 (s, 3 H), 3.81 (s, 3 H), 3.32 (d, J = 11.0 Hz, 1 H), 3.08 (s, 3 H), 2.80 (d, J = 11.0 Hz, 1 H), 2.47 – 2.09 (m, 5 H), 2.04 (s, 3 H), 2.01 – 1.72 (m, 4 H), 1.28 (s, 3 H). $^{13}$C-NMR (75 MHz, $CDCl_3$) δ 175.66, 171.01, 169.32, 161.29, 158.75, 153.25, 135.40, 131.55, 128.16, 115.52, 107.93, 103.65, 98.12, 89.47, 83.68, 62.62, 55.05, 55.00, 52.75, 52.28, 50.62, 50.54, 50.03, 41.76, 39.76, 38.37, 35.52, 21.60, 16.51, 14.44. HRMS for $C_{31}H_{37}O_{11}S$ $(M+H)^+$ 617.2051. Found: 617.2052.

***3α-azido-13-acetoxy-ent-10β-hydroxy-20-norgibberella-1,16-diene-7,19-dioic acid-7-(2,4-dimethoxybenzyl) ester-19,10-lactone*** (**6**): White solid, yield 89%, m.p. 126-128 °C. $^{1}$H-NMR (300 MHz, $CDCl_3$): δ 7.24 (d, J = 8.3 Hz, 1 H), 6.50 - 6.46 (m, 2 H), 6.41 (dd, J = 9.3, 2.0 Hz, 1 H), 5.92 (dd, J = 9.3, 2.6 Hz, 1 H), 5.20 (s, 1 H), 5.18 – 5.12 (m, 2 H), 4.97 (s, 1 H), 4.05 (t, J = 2.3 Hz, 1 H), 3.84 (s, 3 H), 3.80 (s, 3 H), 2.99 (d, J = 10.7 Hz, 1 H), 2.77 (d, J = 10.8 Hz, 1 H), 2.03 (s, 3 H), 1.29 (s, 3 H). $^{13}$C-NMR (75 MHz, $CDCl_3$) δ： 174.64, 171.22, 169.23, 161.30, 158.72, 153.20, 132.90, 131.56, 127.98, 115.42, 107.78, 103.67, 98.07, 88.17, 83.69, 63.84, 62.68, 57.27, 55.02, 54.97, 52.75, 50.62, 50.47, 50.43, 41.79, 39.80, 35.58, 21.58, 16.56, 14.51. HRMS for $C_{30}H_{37}N_4O_8$ $(M+NH_4)^+$ 581.2606. Found: 581.2605.

**Spectral Data of 8a~8o:**

***N-Prop-2-ynylbenzamide*** (**8a**): White solid, yield 98%, $^{1}$H-NMR ($CDCl_3$): δ 7.79 (d, 2 H, J = 7.3 Hz), 7.56-7.40 (m, 3 H), 6.35 (br, 1 H), 4.26 (dd, 2 H, J = 5.1, 2.5 Hz), 2.29 (t, 1 H, J = 2.4 Hz). The analytical results of compound **8a** were identical with the ones described in the literature [2].

***N-Prop-2-ynyl phenylacetamide*** (**8b**): White solid, yield 90%, $^{1}$H-NMR ($CDCl_3$): δ 7.28-7.43 (m, 5 H), 5.79 (br, 1 H), 4.04 (dd, 2 H, J = 5.1, 2.4 Hz), 3.62 (s, 2 H), 2.22 (t,1 H, J = 2.6 Hz). The analytical results of compound **8b** were identical with the ones described in the literature [3].

***N-Prop-2-ynyl-p-methylphenylacetamide*** (**8c**): White solid, yield 85%, m.p. 125-126 °C. $^{1}$H-NMR ($CDCl_3$): δ 7.16 – 7.23 (m, 4 H), 5.61 (br, 1 H), 4.04 (dd, 5.3, 2.6 Hz), 3.59 (s, 2 H), 2.39 (s, 3 H), 2.21 (t, 1 H, J = 2.6 Hz). $^{13}$C NMR (75 MHz, $CDCl_3$): δ 170.62, 136.79, 129.39, 129.00, 79.15, 71.12, 42.68, 28.93, 20.72. HRMS for $C_{12}H_{14}NO$ (M+H)$^+$ 188.1070. Found: 188.1070.

***N-Prop-2-ynyl-p-methoxyphenylacetamide*** (**8d**): White solid, yield 95%, m.p. 99-100 °C. $^{1}$H-NMR ($CDCl_3$): δ 7.20 (d, 2 H, J = 8.7 Hz), 6.91 (d, 2 H, J = 8.7 Hz), 5.83 (br, 1 H), 4.02 (dd, 2 H, J = 5.4, 2.7 Hz), 3.83 (s, 3 H), 3.55 (s, 2 H), 2.21 (t, 1 H, J = 2.7 Hz). $^{13}$C NMR (75 MHz, $CDCl_3$): δ 170.93, 158.54, 130.16, 126.17, 114.06, 79.24, 71.11, 54.93, 42.08,28.91. HRMS for $C_{12}H_{14}NO_2$ (M+H)$^+$ 204.1019. Found: 204.1018.

***N-Prop-2-ynyl-p-chlorobenzamide*** (**8e**): White solid, yield 92%, $^{1}$H-NMR ($CDCl_3$): δ 7.64 (d, 2 H, J = 8.4 Hz), 7.33 (d, 2 H, J = 8.7 Hz), 6.41 (s, 1 H), 4.13 (dd, 2H, J = 5.4 Hz, J = 2.4 Hz), 2.24 (t, 1 H, J = 2.4 Hz). The analytical results of compound **8e** were identical with the ones described in the literature [4].

***N-Prop-2-ynyl-α-naphthoacetylamide*** (**8f**): White solid, yield 93 %, m.p. 141-142 °C. $^{1}$H-NMR ($CDCl_3$): δ 8.04 – 7.78 (m, 3 H), 7.62 – 7.36 (m, 4 H), 5.58 (s, 1 H), 4.04 (d, 2 H), 3.94 (dd, J = 5.4, 2.5 Hz, 2 H), 2.10 (t, J = 2.5 Hz, 1 H). $^{13}$C-NMR (75 MHz, $CDCl_3$): δ 170.22, 133.66, 131.69, 130.27, 128.48, 128.31, 128.09, 126.56, 125.92, 125.30, 123.40, 78.93, 71.06, 41.16, 28.91. HRMS for $C_{15}H_{14}NO$ (M+H)$^+$ 224.1070. Found: 224.1069.

***N-Prop-2-ynyl-o-isopropyl-p-chlorobenzamide*** (**8g**): White solid, yield 89 %, m.p. 132-133 °C. $^{1}$H-NMR (300 MHz, $CDCl_3$): δ 7.32 – 7.22 (m, 3 H), 6.20 (s, 1 H), 3.98 (dddd, J = 58.5, 17.6, 5.3, 2.6 Hz, 2 H), 2.87 (d, J = 10.3 Hz, 1 H), 2.18 (t, J = 2.6 Hz, 1 H), 1.04 (d, J = 6.5 Hz, 3 H), 0.69 (d, J = 6.7 Hz, 3 H). $^{13}$C-NMR (75 MHz, $CDCl_3$): δ 172.60, 137.13, 132.70, 129.60, 129.31, 128.28, 79.02, 71.30, 60.53, 31.48, 28.94, 21.11, 19.96. HRMS for $C_{13}H_{15}ClNO$ (M+H)$^+$ 236.0837. Found: 236.0838.

***N-Prop-2-ynyl-n-butylamide*** (**8h**): White solid, yield 82 %, $^{1}$H-NMR ($CDCl_3$): δ 5.72 (s, 1 H), 4.08 (dd, 2 H, J = 5.2, 2.6 Hz), 2.22 (m, 3 H), 1.72 (m, 2 H), 0.98 (t, 3 H, J = 7.4 Hz). The analytical results of compound **8h** was identical with the ones described in the literature [5].

***N-Prop-2-ynyl-(2,2,3,3-tetramethylcyclopropane-1-formoxyl)-amide*** (**8i**): White solid, yield 95%, m.p. 99-100 °C. $^{1}$H-NMR ($CDCl_3$): δ 5.87 (s, 1 H), 3.99 (dd, J = 5.2, 2.6 Hz, 2 H), 2.18 (t, J = 2.5 Hz, 1 H), 1.23 (s, 6 H), 1.12 (s, 6 H), 0.85 (s, 1 H). $^{13}$C-NMR (75 MHz, $CDCl_3$): δ 171.08, 79.89, 70.78, 36.99, 28.57, 28.27, 23.29, 16.42. HRMS for $C_{11}H_{18}NO$ (M+H)$^+$ 180.1383. Found: 180.1382.

***N-Prop-2-ynyl-pivalamide*** (**8j**): White solid, yield 89 %, $^{1}$H-NMR ($CDCl_3$): δ 5.96 (br, 1 H), 4.05 (dd, 2 H, J = 5.1, 2.4 Hz), 2.25 (t, 1 H, J = 2.4 Hz), 1.22 (s, 9 H). The analytical results of compound **8j** were identical with the ones described in the literature [6].

***N-Prop-2-ynyl-acetylamide*** (**8k**): White solid, yield 96%, $^{1}$H-NMR ($CDCl_3$): δ 5.99 (br, 1 H), 4.07 (dd, 2 H, J = 5.1, 2.4 Hz), 2.26 (t, 1 H, J = 2.4 Hz), 2.04 (s, 3 H). Compound **8k** is identical with the compound described in the literature [7].

***N-(Prop-2-ynyl)-2-chloro-acetylamide*** (**8l**): White solid, yield 99 %, $^{1}$H-NMR ($CDCl_3$): δ 6.83 (br, 1 H), 4.14 (dd, 2 H, J = 5.4, 2.7 Hz), 4.11 (s, 2 H), 2.32 (t, 1 H, J = 2.4 Hz). The analytical results of compound **8l** were identical with the ones described in the literature [7].

***N-(Prop-2-ynyl)-2-chloro-nicotinamide*** (**8m**): White solid, yield 91%, $^{1}$H-NMR ($CDCl_3$): δ 8.46 (dd, 1 H, J = 3.0,2.2 Hz), 8.10 (dd, 1 H, J = 6.2, 3.0 Hz), 7.36 (d, 1 H, 3.0 Hz), 6.94 (br, 1 H), 4.25 (m, 2 H), 2.32 (t, 1 H, 2.2 Hz). The analytical results of compound **8m** were identical with the ones described in the literature [8].

***N-(Prop-2-ynyl)-3,6-dichloro-picolinamide*** (**8n**): White solid, yield 96%, m.p. 113-114 °C. $^{1}$H-NMR ($CDCl_3$): δ 7.87 (br, 1 H), 7.82 (d, 1 H, J = 8.4 Hz), 7.44 (d, 1 H, J = 8.4 Hz), 4.27 (dd, 2 H, J = 5.6, 2.7 Hz), 2.32 (t, 1 H, J = 2.6 Hz). $^{13}$C NMR (75 MHz, $CDCl_3$): δ 161.33, 147.31, 145.09, 142.66, 130.63, 127.25, 78.72, 71.53, 28.93. HRMS for $C_9H_7Cl_2N_2O$ (M+H)$^+$ 228.9930. Found: 228.9926.

*N-Prop-2-ynyl-isonicotinamide* (**8o**): White solid, yield 97%, $^1$H-NMR ($CDCl_3$): δ 9.30 (t, 1 H, J = 5.3 Hz), 8.75 (m, 2 H), 7.79 (m, 2 H), 4.11 (dd, 2 H, J = 5.7, 2.6 Hz), 3.20 (t, 1 H, J = 2.5 Hz). The analytical results of compound **8o** were identical with the ones described in the literature [9].

**Spectral Data of 9a~9o:**

***3α-(4-benzamido-methyl-1H-1,2,3-trizol-1-yl)-13-acetoxy-ent-10β-hydroxy-20-norgibberella-1,16-diene-7,19-dioic acid-7-(2,4-dimethoxybenzyl) ester-19,10-lactone*** (**9a**): White solid, yield 78%, m.p. 90-91 °C. $^1$H-NMR (300 MHz, $CDCl_3$): δ 7.79 - 7.83 (m, 2 H), 7.30 – 7.48 (m, 5 H), 7.23 (d, 1 H, J = 8.4 Hz), 6.50 (dd, 1 H, J = 9.3, 2.4 Hz), 6.44 - 6.47 (m, 2 H), 5.83 (dd, 1 H, J = 9.3, 2.7 Hz), 5.49 (t, 1 H, 2.4 Hz), 5.21 (s, 1 H), 5.12 (m, 2 H), 4.98 (s, 1 H), 4.69 (dq, 2 H, J = 15.3, 5.4 Hz), 3.81 (s, 3 H), 3.77 (s, 3 H), 3.21 (d, 1 H, J = 10.8 Hz), 2.78 (s, 1 H, J = 10.8 Hz), 2.20 - 2.44 (m, 6 H), 2.02 (s, 3 H), 1.81 - 1.91 (m, 3 H), 1.09 (s, 3 H). $^{13}$C-NMR (75 MHz, $CDCl_3$): δ 174.58, 171.13, 169.39, 167.19, 161.44, 158.85, 153.18, 144.65, 133.78, 131.76, 131.26, 128.23, 127.55, 126.85, 121.52, 115.46, 108.04, 103.71, 98.22, 88.29, 83.74, 63.84, 62.93, 57.83, 55.12, 55.06, 53.79, 50.79, 50.66, 50.55, 41.94, 41.68, 39.91, 35.63, 35.18, 26.72, 24.70, 21.69, 16.66, 14.27. HRMS for $C_{40}H_{42}N_4O_9$ $(M+H)^+$ 723.3025. Found: 723.3028.

***3α-(4-phenylacetamido-methyl-1H-1,2,3-trizol-1-yl)-13-acetoxy-ent-10β-hydroxy-20-norgibberella-1,16-diene-7,19-dioic acid-7-(2,4-dimethoxybenzyl) ester-19,10-lactone*** (**9b**): White solid, yield 76%, m.p. 118-119 °C. $^1$H-NMR ($CDCl_3$): δ 7.31 – 7.36 (m, 4 H), 7.24 – 7.29 (m, 4 H), 6.53 (dd, 1 H, J = 9.3, 2.4 Hz), 6.46 - 6.50 (m, 2 H), 5.82 (dd, 1 H, J = 9.3, 2.4 Hz), 5.49 (t, 1 H, 2.6 Hz), 5.24 (s, 1 H), 5.09 – 5.20 (m, 2 H), 5.00 (s, 1 H), 4.48 (d, 2 H, J = 5.7 Hz), 3.84 (s, 3 H), 3.79 (s, 3 H), 3.60 (s, 2 H), 3.22 (d, 1 H, J = 10.5 Hz), 2.81 (d, 1 H, J = 10.8 Hz), 2.15 – 2.51 (m, 6 H), 2.05 (s, 3 H), 1.73 – 1.86 (m, 3 H), 1.09 (s, 3 H). $^{13}$C-NMR (75 MHz, $CDCl_3$): δ 174.43, 171.05, 170.74, 169.32, 161.36, 158.77, 153.09, 144.61, 134.39, 133.70, 131.68, 129.05, 128.59, 127.45, 126.89, 121.16, 115.38, 107.98, 103.61, 98.15, 88.17, 83.66, 63.72, 62.86, 57.75, 55.05, 54.98, 50.73, 50.57, 50.48, 43.17, 41.87, 39.85, 35.56, 34.77, 21.62, 16.60, 14.17. HRMS for C41H45N4O9 $(M+H)^+$ 737.3181. Found: 737.3190.

***3α-(4-p-methylphenylacetamido-methyl-1H-1,2,3-trizol-1-yl)-13-acetoxy-ent-10β-hydroxy-20-norgibberella-1,16-diene-7,19-dioic acid-7-(2,4-dimethoxybenzyl) ester-19,10-lactone*** (**9c**): White solid, yield 85%, m.p. 102-103 °C. $^1$H-NMR (300 MHz, $CDCl_3$): δ 7.32 (s, 1 H), 7.26 (d, 1 H, J = 7.8 Hz), 7.16 (m, 4 H), 6.53 (dd, 1 H, J = 9.3, 2.7 Hz), 6.47 - 6.50 (m, 2 H), 6.09 (t, 1 H, J= 5.6 Hz), 5.83 (dd, 1 H, J = 9.3, 2.4 Hz), 5.49 (t, 1 H, J = 2.4 Hz), 5.24 (s, 1 H), 5.10 – 5.21 (m, 2 H), 5.01 (s, 1 H), 4.42 – 4.54 (m, 2 H), 3.84 (s, 3 H), 3.80 (s, 3 H), 3.57 (s, 2 H), 3.23 (d, 1 H, J = 10.8 Hz), 2.81 (d, 1 H, J = 10.8 Hz), 2.45 – 2.51 (m, 2 H), 2.36 (s, 3 H), 2.10 – 2.30 (m, 4 H), 2.06 (s, 3 H), 1.73 – 1.86 (m, 3 H), 1.09 (s, 3 H). $^{13}$C-NMR (75 MHz, $CDCl_3$): δ 174.41, 171.05, 170.98, 169.31, 161.37, 158.77, 153.10, 144.68, 136.53, 133.68, 131.68, 131.25, 127.48, 121.11, 115.39, 107.97, 103.62, 98.15, 88.15, 83.66, 63.72, 62.85, 57.76, 55.05, 54.98, 53.68, 50.73, 50.58, 50.48, 42.78, 41.88, 39.85, 35.57, 34.76, 21.61, 20.71, 16.61, 14.16. HRMS for C42H47N4O9 $(M+H)^+$ 751.3338. Found: 751.3334.

***3α-(4-p-methoxyphenylacetamido-methyl-1H-1,2,3-trizol-1-yl)-13-acetoxy-ent-10β-hydroxy-20-norgibberella-1,16-diene-7,19-dioic acid-7-(2,4-dimethoxybenzyl) ester-19,10-lactone*** (**9d**): White solid, yield 88%, m.p. 98-99 °C. $^1$H-NMR (300 MHz, DMSO-$d_6$): δ 8.54 (t, J = 5.6 Hz, 1 H), 7.66 (s, 1H), 7.29 (d, J = 8.3 Hz, 1 H), 7.19 (d, J = 8.6 Hz, 2 H), 6.87 (d, J = 8.6 Hz, 2 H), 6.64 (dd, J = 9.3, 2.1 Hz, 1 H) 6.59 – 6.50 (m, 2 H), 5.91 (dd, J = 9.3, 2.4 Hz, 1 H), 5.77 (m, 1 H), 5.19 (s, 1 H), 5.13 – 5.03 (m, 2 H), 4.98 (s, 1 H), 4.32 (d, J = 5.4 Hz, 2 H), 3.78 (s, 3 H), 3.76 (s, 3 H), 3.74 (s, 3 H), 3.38 (s, 2 H), 3.20 (d, J = 10.7 Hz, 1 H), 2.66 (d, J = 10.7 Hz, 1 H), 2.39 - 2.04 (m, 6 H), 2.01 (m, 3 H), 1.82 – 1.70 (m, 3 H), 0.99 (s, 3 H). $^{13}$C-NMR (75 MHz, DMSO-$d_6$): δ 174.91, 169.41, 166.31, 153.91, 149.38, 144.78, 136.99, 134.28, 132.73, 131.37, 128.51, 128.37, 127.40, 123.26, 107.35, 88.91, 83.84, 79.35, 63.11, 57.73, 53.76, 50.19, 49.91, 42.00, 41.44, 35.97, 34.91, 26.56, 24.41, 21.85, 16.57, 14.56. HRMS for C42H47N4O10 $(M+H)^+$ 767.3287. Found: 767.3293.

***3α-(4-p-chlorobenzamido-methyl-1H-1,2,3-trizol-1-yl)-13-acetoxy-ent-10β-hydroxy-20-norgibberella-1,16-diene-7,19-dioic acid-7-(2,4-dimethoxybenzyl) ester-19,10-lactone*** (**9e**): White solid, yield 81%, m.p. 105-106 °C. $^1$H-NMR (300 MHz, DMSO-$d_6$): δ 9.14 (t, J = 5.7 Hz, 1 H), 7.91 (d, J = 8.7 Hz, 2 H), 7.79 (s, 1 H), 7.56 (d, J = 8.6 Hz, 2 H), 7.28 (d, J = 8.3 Hz, 1 H), 6.63 (dd, J = 9.3, 2.4 Hz, 1 H), 6.58 – 6.49 (m, 2 H), 5.94 (dd, J = 9.3, 2.5 Hz, 1 H), 5.77 – 5.76 (m, 1 H), 5.17 (s, 1 H), 5.07 (m, 2 H), 4.97 (s, 1 H), 4.60 – 4.47 (m, 2 H), 3.78 (s, 3 H), 3.76 (s, 3 H), 3.19 (d, J = 10.7 Hz, 1 H), 2.64 (d, J = 10.7 Hz, 1 H), 2.37 – 2.03 (m, 6 H), 2.01 (s, 3 H), 1.82 – 1.71 (m, 3 H), 0.99 (s, 3 H). $^{13}$C-NMR (75 MHz, DMSO-$d_6$): δ 174.50, 170.98, 169.47, 165.27, 161.41,

158.92, 153.66, 144.59, 136.25, 133.04, 132.53, 132.00, 129.33, 128.48, 123.28, 115.49, 107.87, 104.54, 98.42, 88.71, 83.67, 62.95, 62.59, 57.27, 55.55, 55.39, 53.62, 50.74, 50.32, 50.15, 41.43, 35.68, 34.94, 26.55, 21.79, 16.58, 14.42. HRMS for C40H42ClN4O9 $(M+H)^+$ 757.2635. Found: 757.2632.

***3α-(4-α-naphthoacetylamido-methyl-1H-1,2,3-trizol-1-yl)-13-acetoxy-ent-10β-hydroxy-20-norgibberella-1,16-diene-7,19-dioic acid-7-(2,4-dimethoxybenzyl) ester-19,10-lactone*** (**9f**) : White solid, yield 77%, m.p. 96-97 °C. $^1$H-NMR (300 MHz, $CDCl_3$): δ 7.96 – 7.93 (m, 1 H), 7.87 – 7.84 (m, 1 H), 7.81 – 7.78 (m, 1 H), 7.52 – 7.40 (m, 4 H), 7.25 – 7.21 (m, 2 H), 6.49 – 6.44 (m, 3 H), 6.37 (t, J = 5.8 Hz, 1 H), 5.70 (dd, J = 9.3, 2.5 Hz, 1 H), 5.41 (t, J = 2.5 Hz, 1 H), 5.23 (s, 1 H), 5.13 (m, 2 H), 4.99 (s, 1 H), 4.46 – 4.33 (m, 2 H), 4.02 (s, 2 H), 3.81 (s, 3 H), 3.77 (s, 3 H), 3.19 (d, J = 10.7 Hz, 1 H), 2.79 (d, J = 10.7 Hz, 1 H), 2.50 – 2.43 (m, 2 H), 2.27 – 2.13 (m, 4 H), 2.03 (s, 3 H), 1.92 – 1.74 (m, 3 H), 1.04 (s, 3 H). $^{13}$C-NMR (75 MHz, $CDCl_3$): δ 174.36, 171.04, 170.68, 169.33, 161.36, 158.77, 153.10, 144.67, 133.57, 131.71, 131.68, 130.63, 128.41, 128.00, 127.42, 126.33, 125.68, 125.33, 123.46, 121.09, 115.38, 107.97, 103.64, 98.15, 88.13, 83.67, 63.64, 62.85, 57.71, 55.05, 54.99, 53.64, 50.70, 50.57, 50.47, 41.85, 41.04, 39.85, 35.57, 34.74, 21.62, 16.61, 14.15. HRMS for C45H47N4O9 $(M+H)^+$ 787.3338. Found: 787.3334.

***3α-[4-(2-isopropyl-4-chlorobenzamido)-methyl-1H-1,2,3-trizol-1-yl]-13-acetoxy-ent-10β-hydroxy-20-norgibberella-1,16-diene-7,19-dioic acid-7-(2,4-dimethoxy-benzyl) ester-19,10-lactone*** (**9g**): White solid, yield 80%, m.p. 102-103 °C. $^1$H-NMR (300 MHz, $CDCl_3$): δ 7.31 (s, 2 H), 7.29 – 7.24 (m, 3 H), 6.56 – 6.46 (m, 3 H), 5.74 (dd, J = 9.4, 2.4 Hz, 1 H), 5.46 (t, J = 2.4 Hz, 1 H), 5.24 (s, 1 H), 5.20 – 5.10 (m, 2 H), 5.00 (s, 1 H), 4.48 (d, J = 5.5 Hz, 2 H), 3.84 (s, 3 H), 3.80 (s, 3 H), 3.22 (d, J = 10.6 Hz, 1 H), 2.87 (dd, J = 10.4, 3.8 Hz, 1 H), 2.80 (d, J = 10.7 Hz, 1 H), 2.52 – 2.15 (m, 6 H), 2.06 (s, 3 H), 1.82 – 1.80 (m, 3 H), 1.08 (s, 3 H), 1.01 (dd, J = 15.7, 6.4 Hz, 3 H), 0.71 (d, J = 6.6 Hz, 3 H). $^{13}$C-NMR (75 MHz, $CDCl_3$): δ 174.36, 172.79, 171.05, 169.33, 161.36, 158.77, 153.09, 137.58, 133.69, 132.40, 131.67, 129.33, 128.26, 128.15, 127.35, 115.38, 107.97, 103.62, 98.16, 88.19, 88.13, 83.66, 63.70, 62.87, 60.55, 57.73, 55.05, 54.98, 53.68, 53.62, 50.75, 50.58, 50.48, 35.57, 31.26, 31.01, 21.61, 21.14, 21.06, 19.95, 19.90, 14.18. HRMS for C43H48ClN4O9 $(M+H)^+$ 799.3104. Found: 799.3118.

***3α-(4-n-butylamido-methyl-1H-1,2,3-trizol-1-yl)-13-acetoxy-ent-10β-hydroxy-20-norgibberella-1,16-diene-7,19-dioic acid-7-(2,4-dimethoxy-benzyl) ester-19,10-lactone*** (**9h**): White solid, yield 75%, m.p. 92-93 °C. $^1$H-NMR (300 MHz, $CDCl_3$): δ 7.36 (s, 1 H), 7.22 (d, J = 8.5 Hz, 1 H), 6.61 (t, J = 5.4 Hz, 1 H), 6.50 (dd, J = 9.3, 2.5 Hz, 1 H), 6.46 – 6.43 (m, 2 H), 5.82 (dd, J = 9.3, 2.5 Hz, 1 H), 5.48 (t, J = 2.5 Hz, 1 H), 5.20 (s, 1 H), 5.11 (m, 2 H), 4.97 (s, 1H), 4.48 (qd, J = 15.3, 5.7 Hz, 2 H), 3.80 (s, 3 H), 3.76 (s, 3 H), 3.20 (d, J = 10.7 Hz, 1 H), 2.75 (d, J = 10.7 Hz, 1 H), 2.46 – 2.40 (m, 2 H), 2.26 – 2.11 (m, 6 H), 2.01 (s, 3 H), 1.96 – 1.70 (m, 3 H), 1.64 (q, J = 7.4 Hz, 2 H), 1.07 (s, 3 H), 0.91 (t, J = 7.4 Hz, 3 H). $^{13}$C-NMR (75 MHz, $CDCl_3$): δ 174.48, 172.80, 171.02, 169.30, 161.34, 158.74, 153.05, 133.65, 131.65, 127.47, 121.20, 115.34, 107.95, 103.62, 98.11, 88.20, 83.63, 63.69, 62.83, 57.71, 55.02, 54.96, 53.67, 50.72, 50.55, 50.45, 41.81, 39.82, 37.96, 35.52, 34.50, 21.58, 18.65, 16.56, 14.15, 13.37. HRMS for C37H45N4O9 $(M+H)^+$ 689.3181. Found: 689.3192.

***3α-[4-(2,2,3,3-tetramethylcyclopropane-1-formamido)-methyl-1H-1,2,3-trizol-1-yl]-13-acetoxy-ent-10β-hydroxy-20-norgibberella-1,16-diene-7,19-dioic acid-7-(2,4-dimethoxy-benzyl) ester-19,10-lactone*** (**9i**): White solid, yield 79%, m.p. 109-110 °C. $^1$H-NMR (300 MHz, $CDCl_3$): δ 7.34 (s, 1 H), 7.22 (d, J = 8.4 Hz, 1 H), 6.50 (dd, J = 9.3, 2.5 Hz, 1 H), 6.46 – 6.43 (m, 2 H), 6.34 (t, J = 5.6 Hz, 1 H), 5.82 (dd, J = 9.3, 2.5 Hz, 1 H), 5.48 (t, J = 2.5 Hz, 1 H), 5.20 (s, 1 H), 5.16 – 5.10 (m, 2 H), 4.97 (s, 1 H), 4.53 – 4.39 (m, 2 H), 3.80 (s, 3 H), 3.76 (s, 3 H), 3.20 (d, J = 10.8 Hz, 1 H), 2.78 (d, J = 10.8 Hz, 1 H), 2.47 – 2.11 (m, 7 H), 2.02 (s, 3 H), 1.92 – 1.69 (m, 3 H), 1.25 (s, 3 H), 1.23 (s, 3 H), 1.14 (s, 3 H), 1.13 (s, 3 H), 1.08 (s, 3 H). $^{13}$C-NMR (75 MHz, $CDCl_3$): δ 174.43, 171.43, 171.02, 169.29, 161.34, 158.74, 153.07, 133.59, 131.65, 127.53, 121.10, 115.36, 107.95, 103.62, 98.12, 88.16, 83.63, 63.71, 62.83, 57.74, 55.02, 54.96, 53.66, 50.71, 50.55, 50.45, 41.84, 39.82, 37.02, 35.53, 34.48, 27.90, 27.85, 23.28, 21.59, 16.57, 16.46, 14.16. HRMS for C41H51N4O9 $(M+H)^+$ 743.3651. Found: 743.3665.

***3α-(4-pivalamido-methyl-1H-1,2,3-trizol-1-yl)-13-acetoxy-ent-10β-hydroxy-20-norgibberella-1,16-diene-7,19-dioic acid-7-(2,4-dimethoxy-benzyl) ester-19,10-lactone*** (**9j**) : White solid, yield 81%, m.p. 89-90 °C. $^1$H-NMR (300 MHz, $CDCl_3$): δ 7.35 (s, 1 H), 7.25 (d, J = 8.0 Hz, 1 H), 6.53 (dd, J = 9.3, 2.5 Hz, 1 H), 6.49 – 6.46 (m, 2 H), 6.36 (s, 1 H), 5.85 (dd, J = 9.3, 2.5 Hz, 1 H), 5.51 (t, J = 2.5 Hz, 1 H), 5.23 (s, 1 H), 5.14 (q, J = 11.6 Hz, 2 H), 5.00 (s, 1 H), 4.51 (d, J = 5.5 Hz, 2 H), 3.83 (s, 3 H), 3.79 (s, 3 H), 3.23 (d, J = 10.8 Hz, 1 H), 2.80 (d, J = 10.8 Hz, 1 H), 2.50 – 2.43 (m, 2 H), 2.29 – 2.14 (m, 4 H), 2.04 (s, 3 H), 1.85 – 1.74 (m, 3 H), 1.22 (s, 9 H), 1.10 (s, 3 H). $^{13}$C-NMR (75 MHz, $CDCl_3$): δ 178.20, 174.44, 171.04, 169.29, 161.35, 158.76, 153.09, 144.86, 133.75, 131.67, 127.44, 121.05, 115.38, 107.95, 103.60, 98.14, 88.14, 83.64, 63.75, 62.84, 57.77, 55.04, 54.97, 53.70,

50.73, 50.58, 50.46, 41.87, 39.82, 38.27, 35.55, 34.79, 27.12, 21.60, 16.58, 14.16. HRMS for $C_{38}H_{47}N_4O_9$ $(M+H)^+$ 703.3338. Found: 703.3357.

***3α-(4-acetylamido-methyl-1H-1,2,3-trizol-1-yl)-13-acetoxy-ent-10β-hydroxy-20-norgibberella-1,16-diene-7,19-dioic acid-7-(2,4-dimethoxy-benzyl) ester-19,10-lactone*** (**9k**): White solid, yield 83%, m.p. 100-102 °C. $^1$H-NMR (300 MHz, $CDCl_3$): δ 7.37 (s, 1H), 7.22 (d, J = 8.5 Hz, 1H), 6.76 (t, J = 5.4 Hz, 1H), 6.51 (dd, J = 9.3, 2.5 Hz, 1H), 6.47 – 6.43 (m, 2 H), 5.83 (dd, J = 9.3, 2.5 Hz, 1H), 5.49 (t, J = 2.5 Hz, 1H), 5.21 (s, 1 H), 5.11 (q, J = 11.6 Hz, 2 H), 4.97 (s, 1 H), 4.47 (qd, J = 15.4, 5.6 Hz, 2 H), 3.80 (s, 3 H), 3.76 (s, 3 H), 3.21 (d, J = 10.7 Hz, 1 H), 2.77 (d, J = 10.8 Hz, 1 H), 2.47 – 2.40 (m, 2 H), 2.26 – 2.08 (m, 4 H), 2.02 (s, 3 H), 1.99 (s, 3 H), 1.92 – 1.70 (m, 3 H), 1.08 (s, 3 H). $^{13}$C-NMR (75 MHz, $CDCl_3$): δ 174.55, 171.02, 169.93, 169.33, 161.34, 158.75, 153.05, 133.69, 131.67, 127.46, 121.19, 115.34, 107.97, 103.61, 98.12, 88.25, 83.64, 63.71, 62.85, 57.72, 55.04, 54.98, 53.69, 50.70, 50.56, 50.46, 41.83, 39.83, 35.53, 34.63, 22.66, 21.60, 16.58, 14.16. HRMS for $C_{35}H_{40}N_4O_9$ $(M+H)^+$ 661.2868. Found: 661.2885.

***3α-(4-chloroacetamido-methyl-1H-1,2,3-trizol-1-yl)-13-acetoxy-ent-10β-hydroxy-20-norgibberella-1,16-diene-7,19-dioic acid-7-(2,4-dimethoxy-benzyl) ester-19,10-lactone*** (**9l**): White solid, yield 84%, m.p. 91-92 °C. $^1$H-NMR (300 MHz, $CDCl_3$): δ 7.39 – 7.35 (m, 2 H), 7.23 (d, J = 8.4 Hz, 1 H), 6.52 (dd, J = 9.3, 2.5 Hz, 1 H), 6.47 – 6.44 (m, 2 H), 5.83 (dd, J = 9.3, 2.5 Hz, 1 H), 5.52 (t, J = 2.5 Hz, 1 H), 5.21 (d, J = 1.3 Hz, 1 H), 5.12 (q, J = 11.6 Hz, 2 H), 4.98 (s, 1 H), 4.55 (qd, J = 15.3, 5.7 Hz, 2 H), 4.05 (d, J = 0.9 Hz, 2 H), 3.81 (s, 3 H), 3.77 (s, 3 H), 3.22 (d, J = 10.7 Hz, 1 H), 2.78 (d, J = 10.7 Hz, 1 H), 2.48 – 2.41 (m, 2 H), 2.31 – 2.07 (m, 4 H), 2.02 (s, 3 H), 1.95 – 1.70 (m, 3 H), 1.08 (s, 3 H). $^{13}$C-NMR (75 MHz, $CDCl_3$): δ 174.52, 171.00, 169.31, 165.80, 161.35, 158.76, 153.06, 143.74, 133.78, 131.67, 127.42, 121.26, 115.35, 107.96, 103.62, 98.13, 88.24, 83.63, 63.76, 62.85, 57.73, 55.04, 54.98, 53.68, 50.69, 50.56, 50.45, 42.12, 41.83, 39.83, 35.53, 34.92, 21.60, 16.58, 14.13. HRMS for $C_{35}H_{40}ClN_4O_9$ $(M+H)^+$ 695.2478. Found: 695.2499.

***3α-[4-(2-chloro-nicotinamido)-methyl-1H-1,2,3-trizol-1-yl]-13-acetoxy-ent-10β-hydroxy-20-norgibberella-1,16-diene-7,19-dioic acid-7-(2,4-dimethoxy-benzyl) ester-19,10-lactone*** (**9m**): White solid, yield 72 %, m.p. 99-100 °C. $^1$H-NMR (300 MHz, $CDCl_3$):δ 8.40 (dd, J = 4.8, 1.9 Hz, 1 H), 7.97 (dd, J = 7.6, 1.9 Hz, 1 H), 7.57 (t, J = 5.4 Hz, 1 H), 7.48 (s, 1 H), 7.32 - 7.28 (m, 1 H), 7.22 (d, J = 8.4 Hz, 1 H), 6.52 (dd, J = 9.3, 2.5 Hz, 1 H), 6.46 – 6.43 (m, 2 H), 5.83 (dd, J = 9.3, 2.5 Hz, 1 H), 5.49 (t, J = 2.5 Hz, 1 H), 5.20 (s, 1 H), 5.11 (m, 2 H), 4.97 (s, 1 H), 4.69 (d, J = 5.5 Hz, 2 H), 3.80 (s, 3 H), 3.76 (s, 3 H), 3.21 (d, J = 10.8 Hz, 1 H), 2.76 (d, J = 10.7 Hz, 1 H), 2.46 – 2.40 (m, 2 H), 2.25 – 2.06 (m, 4 H), 2.01 (s, 3 H), 1.92 – 1.71 (m, 3 H), 1.06 (s, 3 H). $^{13}$C-NMR (75 MHz, $CDCl_3$): δ 174.47, 171.01, 169.33, 164.73, 161.34, 158.75, 153.04, 150.40, 147.04, 138.78, 133.77, 131.66, 131.04, 127.39, 122.24, 115.34, 107.98, 103.64, 98.12, 88.23, 83.64, 63.73, 62.84, 57.69, 55.04, 54.98, 53.68, 50.69, 50.56, 50.45, 41.80, 39.84, 35.50, 35.26, 21.60, 16.58, 14.15. HRMS for $C_{39}H_{41}ClN_5O_9$ $(M+H)^+$ 758.2587. Found: 758.2604.

***3α-[4-(3,6-dichloro-picolinamido)-methyl-1H-1,2,3-trizol-1-yl]-13-acetoxy-ent-10β-hydroxy-20-norgibberella-1,16-diene-7,19-dioic acid-7-(2,4-dimethoxy-benzyl) ester-19,10-lactone*** (**9n**): White solid, yield 87%, m.p. 94-95 °C. $^1$H-NMR (300 MHz, $CDCl_3$): δ 8.22 (t, J = 5.9 Hz, 1 H), 7.76 (d, J = 8.4 Hz, 1 H), 7.44 (s, 1 H), 7.37 (d, J = 8.4 Hz, 1 H), 7.22 (d, J = 8.3 Hz, 1 H), 6.51 (dd, J = 9.3, 2.6 Hz, 1 H), 6.47 – 6.43 (m, 2 H), 5.84 (dd, J = 9.3, 2.5 Hz, 1 H), 5.52 (t, J = 2.6 Hz, 1 H), 5.21 (s, 1 H), 5.12 (q, J = 11.6 Hz, 2 H), 4.97 (s, 1 H), 4.70 (ddd, J = 32.3, 15.3, 6.0 Hz, 2 H), 3.81 (s, 3 H), 3.77 (s, 3 H), 3.21 (d, J = 10.8 Hz, 1 H), 2.78 (d, J = 10.8 Hz, 1 H), 2.47 2.40 (t, J = 10.0 Hz, 2 H), 2.27 – 2.08 (m, 4 H), 2.02 (s, 3 H), 1.82 – 1.69 (m, 3 H), 1.09 (s, 3 H). $^{13}$C-NMR (75 MHz, $CDCl_3$): δ 174.48, 171.02, 169.31, 161.82, 161.34, 158.75, 153.08, 147.37, 145.63, 142.43, 133.68, 131.66, 130.31, 127.52, 127.04, 121.36, 115.36, 107.94, 103.62, 98.12, 88.19, 83.64, 63.79, 62.84, 57.77, 55.04, 54.98, 53.67, 50.70, 50.57, 50.45, 41.86, 39.82, 35.54, 34.70, 21.60, 16.57, 14.16. HRMS for $C_{39}H_{40}Cl_2N_5O_9$ $(M+H)^+$ 792.2198. Found: 792.2212.

***3α-(4-isonicotinamido-methyl-1H-1,2,3-trizol-1-yl)-13-acetoxy-ent-10β-hydroxy-20-norgibberella-1,16-diene-7,19-dioic acid-7-(2,4-dimethoxy-benzyl) ester-19,10-lactone*** (**9o**) : White solid, yield 83%, m.p. 94-97 °C. $^1$H-NMR (300 MHz, $CDCl_3$): δ 8.73 (s, 2 H), 7.78 (t, J = 5.5 Hz, 1 H), 7.70 (d, J = 5.8 Hz, 2 H), 7.50 (s, 1 H), 7.25 (d, J = 8.0 Hz, 1 H), 6.55 (dd, J = 9.3, 2.5 Hz, 1 H), 6.49 – 6.45 (m, 2 H), 5.86 (dd, J = 9.3, 2.5 Hz, 1 H), 5.51 (t, J = 2.5 Hz, 1 H), 5.23 (s, 1 H), 5.14 (q, J = 11.6 Hz, 2 H), 4.99 (s, 1 H), 4.71 (ddd, J = 37.6, 15.3, 5.6 Hz, 2 H), 3.83 (s, 3 H), 3.79 (s, 3 H), 3.24 (d, J = 10.7 Hz, 1 H), 2.80 (d, J = 10.8 Hz, 1 H), 2.49 – 2.43 (m, 2 H), 2.29 - 2.08 (m, 4 H), 2.04 (s, 3 H), 1.97 – 1.74 (m, 3 H), 1.11 (s, 3 H). $^{13}$C-NMR (75 MHz, $CDCl_3$): δ 174.46, 171.02, 169.32, 165.20, 161.35, 158.75, 153.04, 149.94, 144.06, 140.94, 133.86, 131.67, 127.30, 121.88, 120.88,

115.34, 107.98, 103.63, 98.14, 88.25, 83.63, 63.82, 62.88, 57.69, 55.04, 54.98, 53.70, 50.72, 50.54, 50.47, 41.83, 39.83, 35.53, 34.93, 21.60, 16.58, 14.20. HRMS for $C_{39}H_{42}N_5O_9$ (M+H)$^+$ 724.2977. Found: 724.2989.

**Spectral Data of 10a~10o:**

***3α-(4-benzamido-methyl-1H-1,2,3-trizol-1-yl)-13-acetoxy-ent-10β-hydroxy-20-norgibberella-1,16-diene-7,19-dioic acid-19,10-lactone-7-carboxyl*** (**10a**): White solid, yield 83%, m.p. 174-175 °C. $^1$H-NMR (300 MHz, DMSO-$d_6$): δ 13.15 (s, 1 H), 9.10 (t, J = 5.7 Hz, 1 H), 7.91 – 7.89 (m, 2 H), 7.79 (s, 1 H), 7.54 – 7.47 (m, 3 H), 6.63 (dd, J = 9.3, 2.2 Hz, 1 H), 5.91 (dd, J = 9.3, 2.3 Hz, 1 H), 5.74 (s, 1 H), 5.12 (s, 1 H), 4.98 (s, 1 H), 4.54 (d, J = 4.7 Hz, 2 H), 3.14 (d, J = 10.6 Hz, 1 H), 2.40 – 2.06 (m, 6 H), 1.99 (s, 3 H), 1.88 – 1.73 (m, 3 H), 1.06 (s, 3 H). $^{13}$C-NMR (75 MHz, DMSO-$d_6$): δ 174.91, 169.41, 166.31, 153.91, 144.78, 134.28, 131.37, 128.37, 127.40, 123.26, 107.35, 88.91, 83.84, 79.35, 63.11, 57.73, 53.76, 50.19, 49.91, 42.00, 41.44, 35.97, 34.91, 26.56, 24.41, 21.85, 16.57, 14.56. HRMS for $C_{31}H_{33}N_4O_7$ (M+H)$^+$ 573.2344. Found: 573.2344.

***3α-(4-phenylacetamido-methyl-1H-1,2,3-trizol-1-yl)-13-acetoxy-ent-10β-hydroxy-20-norgibberella-1,16-diene-7,19-dioic acid-19,10-lactone-7-carboxyl*** (**10b**): White solid, yield 72%, m.p. 181-182 °C. $^1$H-NMR (300 MHz, DMSO-$d_6$): δ 12.84 (s, 1 H), 8.60 (t, J = 5.6 Hz, 1 H), 7.69 (s, 1 H), 7.31 – 7.22 (m, 5 H), 6.64 (dd, J = 9.3, 2.4 Hz, 1 H), 5.90 (dd, J = 9.3, 2.5 Hz, 1 H), 5.77 (t, J = 2.4 Hz, 1 H), 5.15 (s, 1 H), 5.01 (s, 1 H), 4.33 (d, J = 5.5 Hz, 2 H), 3.46 (s, 2 H), 3.16 (d, J = 10.7 Hz, 1 H), 2.59 (d, J = 10.7 Hz, 1 H), 2.37 - 2.05 (m, 6 H), 2.00 (s, 3 H), 1.90 - 1.70 (m, 3 H), 1.03 (s, 3 H). $^{13}$C-NMR (75 MHz, DMSO-$d_6$): δ 174.67, 172.54, 170.24, 169.42, 153.52, 144.54, 136.41, 132.61, 129.06, 128.50, 128.30, 126.43, 123.04, 107.62, 88.80, 83.73, 79.28, 62.97, 57.42, 53.69, 51.03, 50.10, 49.96, 42.30, 41.85, 36.06, 34.38, 21.85, 16.52, 14.51. HRMS for C32H35N4O7 (M+H)$^+$ 587.2500. Found: 587.2509.

***3α-(4-p-methylphenylacetamido-methyl-1H-1,2,3-trizol-1-yl)-13-acetoxy-ent-10β-hydroxy-20-norgibberella-1,16-diene-7,19-dioic acid-19,10-lactone-7-carboxyl*** (**10c**): White solid, yield 78%, m.p. 179-181 °C. $^1$H-NMR (300 MHz, DMSO-$d_6$): δ 12.81 (s, 1 H), 8.55 (t, J = 5.6 Hz, 1 H), 7.65 (s, 1 H), 7.13 (q, J = 8.0 Hz, 4 H), 6.63 (dd, J = 9.3, 2.4 Hz, 1 H), 5.90 (dd, J = 9.4, 2.4 Hz, 1 H), 5.76 (t, J = 2.4 Hz, 1 H), 5.15 (s, 1 H), 5.01 (s, 1 H), 4.32 (d, J = 5.5 Hz, 2 H), 3.41 (s, 2 H), 3.16 (d, J = 10.7 Hz, 1 H), 2.59 (d, J = 10.6 Hz, 1 H), 2.37 – 2.05 (m, 9 H), 2.00 (s, 3 H), 1.87 - 1.70 (m, 3 H), 1.02 (s, 3 H). $^{13}$C-NMR (75 MHz, DMSO-$d_6$): δ 174.66, 172.54, 170.45, 169.41, 153.51, 144.62, 135.40, 133.32, 132.59, 128.93, 128.88, 128.50, 122.98, 88.79, 83.72, 79.27, 62.98, 57.42, 53.69, 51.03, 50.10, 49.95, 41.93, 36.05, 34.38, 21.84, 20.76, 16.54, 14.50. HRMS for $C_{33}H_{37}N_4O_7$ (M+H)$^+$ 601.2657. Found: 601.2658.

***3α-(4-p-methoxyphenylacetamido-methyl-1H-1,2,3-trizol-1-yl)-13-acetoxy-ent-10β-hydroxy-20-norgibberella-1,16-diene-7,19-dioic acid-19,10-lactone-7-carboxyl*** (**10d**) : White solid, yield 81%, m.p. 132-135 °C. $^1$H-NMR (300 MHz, DMSO-$d_6$): δ 12.87 (s, 1 H), 8.53 (t, J = 5.5 Hz, 1 H), 7.66 (s, 1 H), 7.18 (d, J = 8.6 Hz, 2 H), 6.86 (d, J = 8.6 Hz, 2 H), 6.64 (dd, J = 9.3, 2.2 Hz, 1 H), 5.90 (dd, J = 9.4, 2.3 Hz, 1 H), 5.76 (s, 1 H), 5.14 (s, 1 H), 5.01 (s, 1 H), 4.31 (d, J = 5.5 Hz, 2 H), 3.73 (s, 3 H), 3.15 (d, J = 10.6 Hz, 1 H), 2.58 (d, J = 10.7 Hz, 1 H), 2.32 - 2.05 (m, 6 H), 2.00 (s, 3 H), 1.84 - 1.73 (m, 3 H), 1.01 (s, 3 H). $^{13}$C-NMR (75 MHz, DMSO-$d_6$): δ 174.68, 170.59, 169.42, 158.02, 153.54, 144.60, 132.58, 130.04, 128.51, 128.33, 123.01, 113.78, 88.81, 83.73, 79.28, 62.96, 57.43, 55.13, 53.68, 50.09, 49.95, 41.86, 41.42, 36.06, 35.90, 34.36, 21.85, 16.52, 14.51. HRMS for $C_{33}H_{37}N_4O_8$ (M+H)$^+$ 617.2606. Found: 617.2608.

***3α-(4-p-chlorobenzamido-methyl-1H-1,2,3-trizol-1-yl)-13-acetoxy-ent-10β-hydroxy-20-norgibberella-1,16-diene-7,19-dioic acid-19,10-lactone-7-carboxyl*** (**10e**) : White solid, yield 80%, m.p. 198-201 °C. $^1$H-NMR (300 MHz, DMSO-$d_6$): δ 12.55 (s, 1 H), 9.12 (t, J = 5.7 Hz, 1 H), 7.90 (d, J = 8.6 Hz, 2 H), 7.80 (s, 1 H), 7.54 (d, J = 8.6 Hz, 2 H), 6.62 (dd, J = 9.3, 2.4 Hz, 1 H), 5.92 (dd, J = 9.3, 2.4 Hz, 1 H), 5.75 (t, J = 2.3 Hz, 1 H), 5.12 (s, 1 H), 4.99 (s, 1 H), 4.53 (d, J = 4.0 Hz, 2 H), 3.14 (d, J = 10.7 Hz, 1 H), 2.56 (d, J = 10.7 Hz, 1 H), 2.33 - 2.03 (m, 6 H), 1.98 (s, 3 H), 1.87 - 1.66 (m, 3 H), 1.02 (s, 3 H). $^{13}$C-NMR (75 MHz, DMSO-$d_6$): δ 174.70, 169.42, 165.30, 153.51, 148.59, 144.55, 136.27, 132.99, 132.57, 129.32, 128.48, 123.37, 107.60, 88.82, 83.71, 62.99, 57.43, 53.70, 51.07, 50.08, 49.92, 48.73, 41.83, 36.03, 34.94, 21.84, 21.15, 16.51, 14.51. HRMS for $C_{31}H_{32}ClN_4O_7$ (M+H)$^+$ 607.1954. Found: 607.1958.

***3α-(4-α-naphthoacetylamido-methyl-1H-1,2,3-trizol-1-yl)-13-acetoxy-ent-10β-hydroxy-20-norgibberella-1,16-diene-7,19-dioic acid-19,10-lactone-7-carboxyl*** (**10f**): White solid, yield 76%, m.p. 179-180 °C. $^1$H-NMR (300 MHz, DMSO-$d_6$): δ 12.68 (s, 1 H), 8.73 (t, J = 5.6 Hz, 1 H), 8.10 – 8.07 (m, 1 H), 7.93 (dd, J = 6.4, 3.1 Hz, 1 H), 7.83 (dd, J = 6.6, 2.9 Hz, 1 H), 7.67 (s, 1 H), 7.59 – 7.41 (m, 4 H), 6.63 (dd, J =

9.3, 2.3 Hz, 1 H), 5.87 (dd, J = 9.3, 2.5 Hz, 1 H), 5.77 (t, J = 2.3 Hz, 1 H), 5.15 (s, 1 H), 5.02 (s, 1 H), 4.37 (d, J = 5.5 Hz, 2 H), 3.97 (s, 2 H), 3.17 (d, J = 10.6 Hz, 1 H), 2.61 (d, J = 10.6 Hz, 1 H), 2.21 (ddd, J = 52.8, 34.5, 10.1 Hz, 6 H), 2.01 (s, 3 H), 1.90 – 1.64 (m, 3 H), 1.03 (s, 3 H). $^{13}$C-NMR (75 MHz, DMSO-$d_6$): δ 174.70, 172.56, 170.23, 169.42, 153.53, 144.63, 133.46, 132.77, 132.66, 132.10, 128.47, 127.84, 127.18, 126.11, 125.71, 125.61, 124.31, 122.96, 107.64, 88.80, 83.73, 79.28, 62.97, 57.42, 53.70, 51.03, 50.10, 49.96, 41.86, 36.07, 34.49, 21.85, 21.15, 16.53, 14.53. HRMS for $C_{36}H_{37}N_4O_7$ (M+H)$^+$ 637.2657. Found: 637.2657.

***3α-[4-(2-isopropyl-4-chlorobenzamido)-methyl-1H-1,2,3-trizol-1-yl]-13-acetoxy-ent-10β-hydroxy-20-norgibberella-1,16-diene-7,19-dioic acid-19,10-lactone-7-carboxyl*** (**10g**) : White solid, yield 89%, m.p. 201-202 °C. $^{1}$H-NMR (300 MHz, DMSO-$d_6$): δ 12.82 (s, 1 H), 8.65 - 8.61 (m, 1 H), 7.51 (d, J = 3.3 Hz, 1 H), 7.34 (s, 3 H), 6.62 (dd, J = 9.3, 2.3 Hz, 1 H), 5.83 (d, J = 9.6 Hz, 1 H), 5.75 (dd, J = 4.4, 2.4 Hz, 1 H), 5.13 (s, 1 H), 5.00 (s, 1 H), 4.41 - 4.17 (m, 2 H), 3.13 (d, J = 10.7 Hz, 1 H), 3.08 (dd, J = 10.6, 2.4 Hz, 1H), 2.56 (d, J = 10.7 Hz, 1 H), 2.33 – 2.13 (m, 6 H), 1.99 (s, 3 H), 1.83 – 1.67 (m, 3 H), 0.98 - 0.90 (m, 6 H), 0.61 (d, J = 6.7 Hz, 3 H). $^{13}$C-NMR (75 MHz, DMSO-$d_6$): δ 174.57, 172.48, 169.42, 153.50, 144.61, 144.58, 139.10, 132.54, 131.40, 130.02, 128.42, 128.18, 107.62, 88.75, 83.71, 62.90, 58.90, 57.37, 53.63, 53.61, 51.00, 50.07, 49.93, 48.73, 41.83, 36.04, 34.23, 30.91, 21.84, 21.24, 21.17, 20.26, 16.52, 14.39. HRMS for $C_{34}H_{38}ClN_4O_7$ (M+H)$^+$ 649.2424. Found: 649.2455.

***3α-(4-n-butylamido-methyl-1H-1,2,3-trizol-1-yl)-13-acetoxy-ent-10β-hydroxy-20-norgibberella-1,16-diene-7,19-dioic acid-19,10-lactone-7-carboxyl*** (**10h**): White solid, yield 71%, m.p. 199-200 °C. $^{1}$H-NMR (300 MHz, DMSO-$d_6$): δ 12.85 (s, 1 H), 8.32 (t, J = 5.0 Hz, 1 H), 7.71 (s, 1 H), 6.64 (dd, J = 9.3, 2.4 Hz, 1 H), 5.92 (dd, J = 9.2, 2.5 Hz, 1 H), 5.77 (s, 1 H), 5.14 (s, 1 H), 5.01 (s, 1 H), 4.30 (d, J = 5.6 Hz, 2 H), 3.14 (d, J = 10.7 Hz, 1 H), 2.57 (d, J = 10.8 Hz, 1 H), 2.35 – 2.05 (m, 8 H), 2.00 (s, 3 H), 1.83 – 1.72 (m, 3 H), 1.53 (dd, J = 14.7, 7.3 Hz, 2 H), 1.01 (s, 3 H), 0.85 (t, J = 7.4 Hz, 3 H). $^{13}$C-NMR (75 MHz, DMSO) δ 174.68, 172.16, 169.43, 153.56, 148.97, 144.78, 132.57, 128.53, 123.06, 107.59, 88.80, 83.72, 62.95, 57.45, 53.68, 51.09, 50.08, 49.92, 48.72, 41.84, 37.26, 36.04, 34.15, 21.84, 18.73, 16.51, 14.48, 13.70. HRMS for $C_{28}H_{35}N_4O_7$ (M+H)$^+$ 539.2500. Found: 539.2512.

***3α-[4-(2,2,3,3-tetramethylcyclopropane-1-formamido)-methyl-1H-1,2,3-trizol-1-yl]-13-acetoxy-ent-10β-hydroxy-20-norgibberella-1,16-diene-7,19-dioic acid-19,10-lactone-7-carboxyl*** (**10i**) : White solid, yield 80%, m.p. 169-170 °C. $^{1}$H-NMR (300 MHz, DMSO-$d_6$): δ 12.66 (s, 1 H), 8.25 (t, J = 5.6 Hz, 1 H), 7.70 (s, 1 H), 6.64 (dd, J = 9.3, 2.4 Hz, 1 H), 5.92 (dd, J = 9.3, 2.4 Hz, 1 H), 5.78 (t, J = 2.3 Hz, 1 H), 5.13 (s, 1 H), 5.00 (s, 1 H), 4.27 (d, J = 5.4 Hz, 2 H), 3.15 (d, J = 10.7 Hz, 1 H), 2.57 (d, J = 10.7 Hz, 1 H), 2.35 - 2.04 (m, 6 H), 1.99 (s, 3 H), 1.88 – 1.68 (m, 3 H), 1.19 (d, J = 3.0 Hz, 6 H), 1.11 (s, 7 H), 1.02 (s, 3 H). $^{13}$C-NMR (75 MHz, DMSO-$d_6$): δ 174.67, 170.96, 169.41, 153.56, 148.99, 144.97, 138.16, 132.53, 128.57, 124.85, 123.15, 107.59, 88.78, 83.72, 79.27, 62.92, 57.45, 53.68, 51.07, 50.09, 49.93, 41.85, 36.02, 34.22, 27.10, 23.64, 21.85, 16.72, 14.47. HRMS for $C_{32}H_{41}N_4O_7$ (M+H)$^+$ 593.2970. Found: 593.2984.

***3α-(4-pivalamido-methyl-1H-1,2,3-trizol-1-yl)-13-acetoxy-ent-10β-hydroxy-20-norgibberella-1,16-diene-7,19-dioic acid-19,10-lactone-7-carboxyl*** (**10j**): White solid, yield 79%, m.p. 172-174 °C. $^{1}$H-NMR (300 MHz, DMSO-$d_6$): δ 12.82 (s, 1 H), 8.05 (t, J = 5.8 Hz, 1 H), 7.61 (s, 1 H), 6.63 (dd, J = 9.3, 2.4 Hz, 1 H), 5.92 (dd, J = 9.3, 2.4 Hz, 1 H), 5.77 (d, J = 2.4 Hz, 1 H), 5.13 (s, 1 H), 5.00 (s, 1 H), 4.30 (d, J = 5.7 Hz, 2 H), 3.14 (d, J = 10.6 Hz, 1 H), 2.57 (d, J = 10.7 Hz, 1 H), 2.33 - 2.04 (m, 6 H), 1.99 (s, 3 H), 1.85 - 1.72 (m, 3 H), 1.10 (s, 9 H), 1.00 (s, 3 H). $^{13}$C-NMR (75 MHz, DMSO-$d_6$): δ 177.61, 174.63, 172.53, 169.40, 153.53, 145.46, 132.56, 128.55, 122.86, 107.60, 88.76, 83.71, 79.26, 62.92, 57.41, 53.68, 51.00, 50.09, 49.93, 41.84, 38.08, 36.05, 34.65, 27.43, 21.84, 16.52, 14.43. HRMS for $C_{29}H_{37}N_4O_7$ (M+H)$^+$ 553.2657. Found: 553.2673.

***3α-(4-acetylamido-methyl-1H-1,2,3-trizol-1-yl)-13-acetoxy-ent-10β-hydroxy-20-norgibberella-1,16-diene-7,19-dioic acid-19,10-lactone-7-carboxyl*** (**10k**) : White solid, yield 85%, m.p. 202-205 °C. $^{1}$H-NMR (300 MHz, DMSO-$d_6$): δ 12.77 (s, 1 H), 8.36 (t, J = 5.6 Hz, 1 H), 7.73 (s, 1 H), 6.64 (dd, J = 9.3, 2.4 Hz, 1 H), 5.93 (dd, J = 9.3, 2.5 Hz, 1 H), 5.76 (t, J = 2.4 Hz, 1 H), 5.14 (s, 1 H), 5.01 (s, 1 H), 4.29 (d, J = 4.4 Hz, 2 H), 3.15 (d, J = 10.6 Hz, 1 H), 2.58 (d, J = 10.6 Hz, 1 H), 2.33 – 2.05 (m, 6 H), 2.00 (s, 3 H), 1.85 - 1.69 (m, 6 H), 1.03 (s, 3 H). $^{13}$C-NMR (75 MHz, DMSO) δ 174.69, 172.54, 169.42, 169.26, 153.54, 144.58, 132.57, 128.55, 123.09, 107.60, 88.81, 83.72, 79.28, 62.96, 57.42, 53.69, 51.05, 50.09, 49.94, 41.84, 36.05, 34.22, 22.56, 16.51, 14.52. HRMS for $C_{26}H_{31}N_4O_7$ (M+H)$^+$ 511.2187. Found: 511.2201.

***3α-(4-chloroacetamido-methyl-1H-1,2,3-trizol-1-yl)-13-acetoxy-ent-10β-hydroxy-20-norgibberella-1,16-diene-7,19-dioic acid-19,10-lactone-7-carboxyl*** (**10l**) : White solid, yield 73%, m.p. 179-180 °C. $^{1}$H-NMR (300 MHz, DMSO-$d_6$): δ 12.67 (s, 1 H), 8.74 (t, J = 5.6 Hz, 1 H), 7.78 (s, 1 H), 6.64 (dd, J = 9.3, 2.4 Hz,

1 H), 5.93 (dd, J = 9.3, 2.5 Hz, 1 H), 5.78 (t, J = 2.5 Hz, 1 H), 5.13 (s, 1 H), 5.00 (s, 1 H), 4.37 (d, J = 5.5 Hz, 2 H), 4.10 (s, 2 H), 3.16 (d, J = 10.7 Hz, 1 H), 2.58 (d, J = 10.7 Hz, 1 H), 2.35 – 2.02 (m, 6 H), 2.00 (s, 3 H), 1.85 - 1.72 (m, 3 H), 1.03 (s, 3 H). $^{13}$C-NMR (75 MHz, DMSO-$d_6$): δ 174.68, 172.60, 169.45, 166.04, 153.55, 143.78, 132.58, 128.50, 123.38, 107.66, 88.80, 83.68, 79.27, 62.91, 57.34, 53.67, 50.97, 50.06, 49.91, 42.62, 41.80, 36.05, 34.62, 21.86, 16.51, 14.52. HRMS for $C_{26}H_{30}ClN_4O_7$ $(M+H)^+$ 545.1818. Found: 545.1798.

***3α-[4-(2-chloro-nicotinamido)-methyl-1H-1,2,3-trizol-1-yl]-13-acetoxy-ent-10β-hydroxy-20-norgibberella-1,16-diene-7,19-dioic acid-19,10-lactone-7-carboxyl*** (**10m**) : White solid, yield 89%, m.p. 146-148 °C. $^1$H-NMR (300 MHz, DMSO-$d_6$): δ 12.80 (s, 1 H), 9.17 (t, J = 5.7 Hz, 1 H), 8.49 (dd, J = 4.8, 1.9 Hz, 1 H), 7.91 (dd, J = 7.5, 1.9 Hz, 1 H), 7.84 (s, 1 H), 7.51 (dd, J = 7.5, 4.8 Hz, 1 H), 6.66 (dd, J = 9.3, 2.4 Hz, 1 H), 5.95 (dd, J = 9.3, 2.5 Hz, 1 H), 5.82 (t, J = 2.4 Hz, 1 H), 5.14 (s, 1 H), 5.01 (s, 1 H), 4.53 (d, J = 5.6 Hz, 2 H), 3.17 (d, J = 10.7 Hz, 1 H), 2.60 (d, J = 10.7 Hz, 1 H), 2.36 - 2.06 (m, 6 H), 1.90 – 1.69 (m, 3 H), 1.05 (s, 3 H). $^{13}$C-NMR (75 MHz, DMSO-$d_6$): δ 174.67, 172.53, 169.42, 165.22, 153.53, 150.36, 146.70, 144.04, 138.10, 133.00, 132.63, 128.52, 123.25, 123.09, 107.62, 88.81, 83.72, 79.27, 63.00, 57.42, 53.72, 51.01, 50.10, 49.95, 41.86, 36.06, 34.88, 21.84, 16.52, 14.48. HRMS for $C_{30}H_{31}ClN_5O_7$ $(M+H)^+$ 608.1907. Found: 608.1920.

***3α-[4-(3,6-dichloro-picolinamido)-methyl-1H-1,2,3-trizol-1-yl]-13-acetoxy-ent-10β-hydroxy-20-norgibberella-1,16-diene-7,19-dioic acid-19,10-lactone-7-carboxyl*** (**10n**) : White solid, yield 90%, m.p. 186-187 °C. $^1$H-NMR (300 MHz, DMSO-$d_6$): δ 12.65 (s, 1 H), 9.25 (t, J = 5.8 Hz, 1 H), 8.11 (d, J = 8.5 Hz, 1 H), 7.84 (s, 1 H), 7.68 (d, J = 8.5 Hz, 1 H), 6.64 (dd, J = 9.3, 2.4 Hz, 1 H), 5.95 (dd, J = 9.3, 2.4 Hz, 1 H), 5.82 (t, J = 2.3 Hz, 1 H), 5.13 (s, 1 H), 5.00 (s, 1 H), 4.53 (d, J = 5.7 Hz, 2 H), 3.16 (d, J = 10.7 Hz, 1 H), 2.58 (d, J = 10.6 Hz, 1 H), 2.35 - 2.02 (m, 6 H), 1.99 (s, 3 H), 1.88 – 1.68 (m, 3 H), 1.04 (s, 3 H). $^{13}$C-NMR (75 MHz, DMSO-$d_6$): δ 174.65, 172.57, 169.42, 163.46, 153.51, 151.33, 147.65, 143.74, 141.90, 132.57, 128.55, 127.82, 126.82, 123.48, 107.61, 88.80, 83.72, 79.25, 62.97, 57.43, 53.69, 51.04, 50.09, 49.94, 41.84, 36.04, 34.61, 21.85, 16.51, 14.49. HRMS for $C_{30}H_{30}Cl_2N_5O_7$ $(M+H)^+$ 642.1517. Found: 642.1527.

***3α-(4-isonicotinamido-methyl-1H-1,2,3-trizol-1-yl)-13-acetoxy-ent-10β-hydroxy-20-norgibberella-1,16-diene-7,19-dioic acid-19,10-lactone-7-carboxyl*** (**10o**) : White solid, yield 75%, m.p. 228-230 °C. $^1$H-NMR (300 MHz, DMSO-$d_6$): δ 12.83 (s, 1 H), 9.38 (t, J = 5.7 Hz, 1 H), 8.75 (d, J = 5.3 Hz, 2 H), 7.84 - 7.82 (m, 3 H), 6.63 (dd, J = 9.3, 2.3 Hz, 1 H), 5.93 (dd, J = 9.3, 2.4 Hz, 1 H), 5.77 (t, J = 2.4 Hz, 1 H), 5.13 (s, 1 H), 5.00 (s, 1 H ), 4.56 (d, J = 3.7 Hz, 2 H), 3.15 (d, J = 10.7 Hz, 1 H), 2.57 (d, J = 10.7 Hz, 1 H), 2.34 – 2.04 (m, 6 H), 1.99 (s, 3 H), 1.88 – 1.71 (m, 3 H), 1.03 (s, 3 H). $^{13}$C-NMR (75 MHz, DMSO-$d_6$): δ 174.69, 172.57, 169.42, 164.65, 153.52, 150.14, 144.15, 141.69, 132.59, 128.52, 123.43, 121.69, 107.61, 88.82, 83.71, 79.26, 63.01, 57.42, 53.70, 51.04, 50.08, 49.93, 41.83, 36.04, 34.97, 21.85, 16.51, 14.52. HRMS for $C_{30}H_{32}N_5O_7$ $(M+H)^+$ 574.2296. Found: 574.2308.

## Notes and References in Supporting Information


1. Serebryakov, E.P.; Agnistikova, V.N.; Suslova, L.M. Structure-activity study of gibberellins. Part 1. Growth-promoting activity of some selectively modified gibberellins. *Phytochemistry* **1984**, *23*, 1847-1854.
2. Das, S.; Li, Y.; Lu, L.-Q.; Junge, K.; Beller, M. A general and selective rhodium-catalyzed reduction of amides, n-acyl amino esters, and dipeptides using phenylsilane. *Chem. - Eur. J.* **2016**, *22*, 7050-7053.
3. Li, J.; Lear, M.J.; Hayashi, Y. Sterically demanding oxidative amidation of $\alpha$-substituted malononitriles with amines using o2. *Angew. Chem., Int. Ed.* **2016**, *55*, 9060-9064.
4. Jia, W.Z.; Cheng, F.; Zhang, Y.J.; Ge, J.Y.; Yao, S.Q.; Zhu, Q. Rapid synthesis of flavone-based monoamine oxidase (mao) inhibitors targeting two active sites using click chemistry. *Chem. Biol. Drug Des.* **2017**, *89*, 141-151.
5. Kogler, M.; Vanderhoydonck, B.; De Jonghe, S.; Rozenski, J.; Van Belle, K.; Herman, J.; Louat, T.; Parchina, A.; Sibley, C.; Lescrinier, E., *et al.* Synthesis and evaluation of 5-substituted 2'-deoxyuridine monophosphate analogs as inhibitors of flavin-dependent thymidylate synthase in mycobacterium tuberculosis. *J. Med. Chem.* **2011**, *54*, 4847-4862.
6. Herner, A.; Nikic, I.; Kallay, M.; Lemke, E.A.; Kele, P. A new family of bioorthogonally applicable fluorogenic labels. *Org. Biomol. Chem.* **2013**, *11*, 3297-3306.
7. Rajagopal, B.; Chen, Y.-Y.; Chen, C.-C.; Liu, X.-Y.; Wang, H.-R.; Lin, P.-C. Cu(i)-catalyzed synthesis of dihydropyrimidin-4-ones toward the preparation of β- and β3-amino acid analogues. *J. Org. Chem.* **2014**, *79*, 1254-1264.
8. Kamal, A.; Reddy, N.V.S.; Nayak, V.L.; Bolla, N.R.; Subba Rao, A.V.; Prasad, B. Synthesis and evaluation of n-((1-benzyl-1h-1,2,3-triazol-4-yl)methyl)nicotinamides as potential anticancer agents that inhibit tubulin polymerization. *Bioorg. Med. Chem.* **2014**, *22*, 3465-3477.
9. Djernes, K.E.; Moshe, O.; Mettry, M.; Richards, D.D.; Hooley, R.J. Metal-coordinated water-soluble cavitands act as c-h oxidation catalysts. *Org. Lett.* **2012**, *14*, 788-791.

$^{1}$H-NMR spectrum of compound **2.**

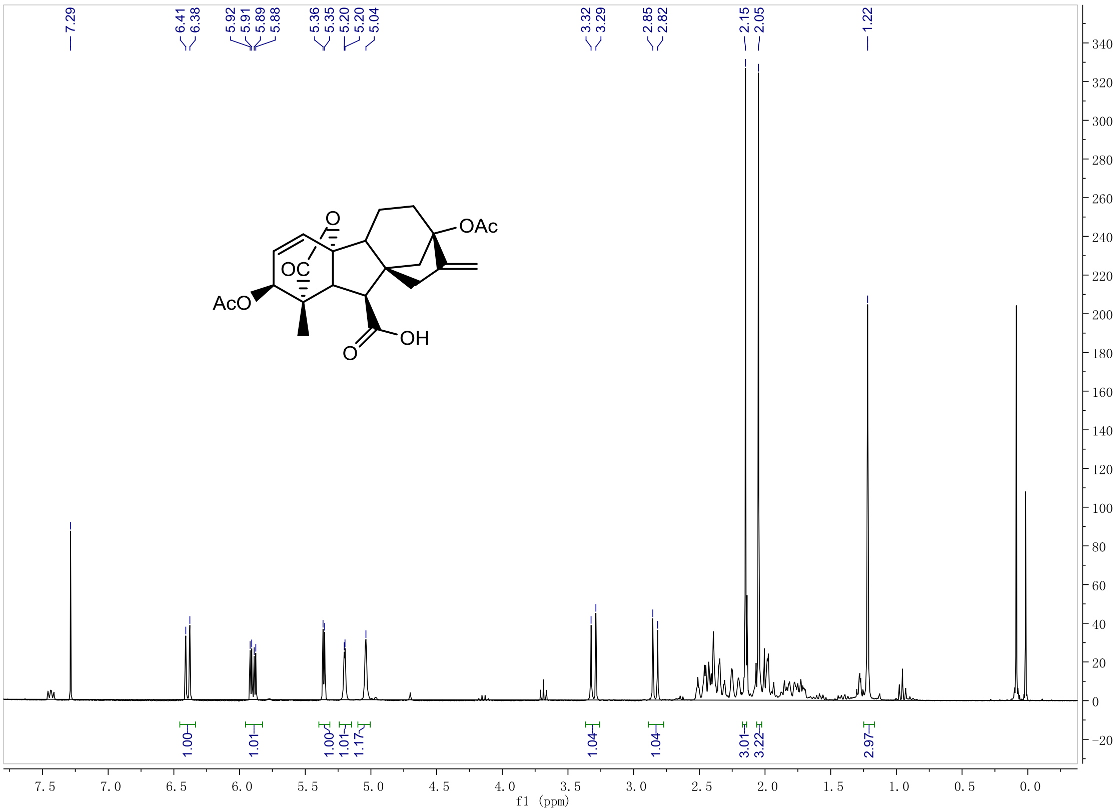


$^{1}$H-NMR spectrum of compound **3.**

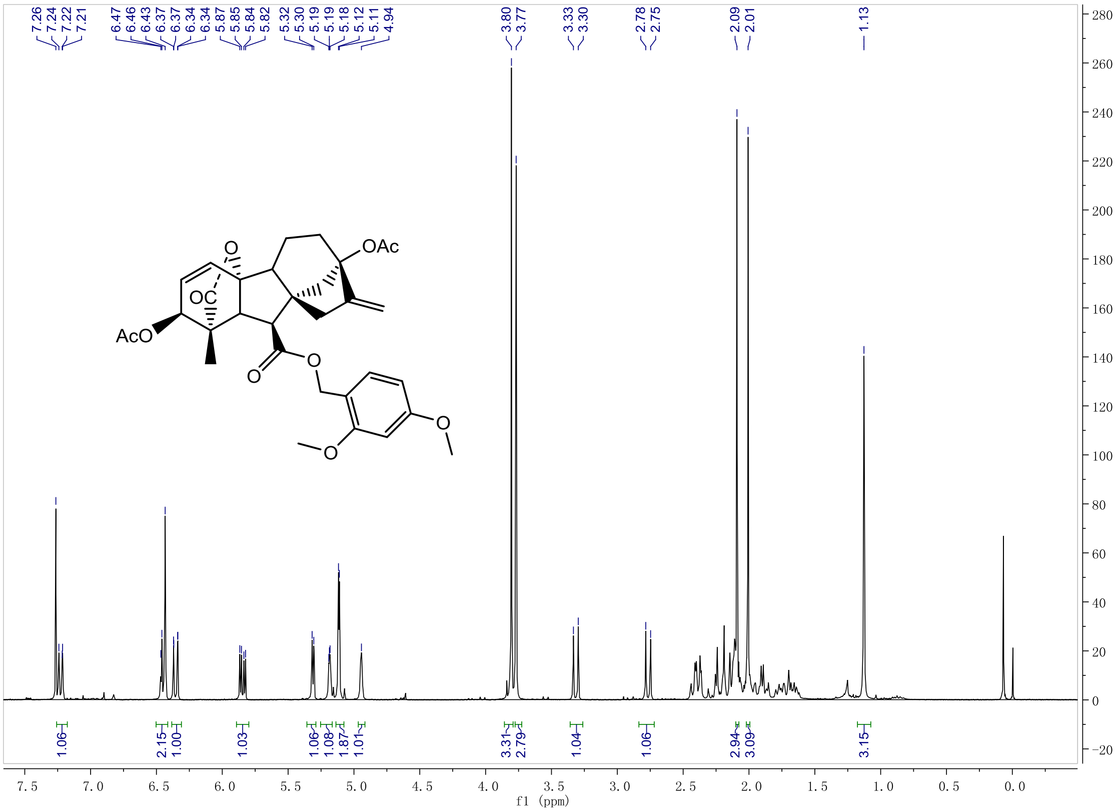

$^{13}$C-NMR spectrum of compound **3.**

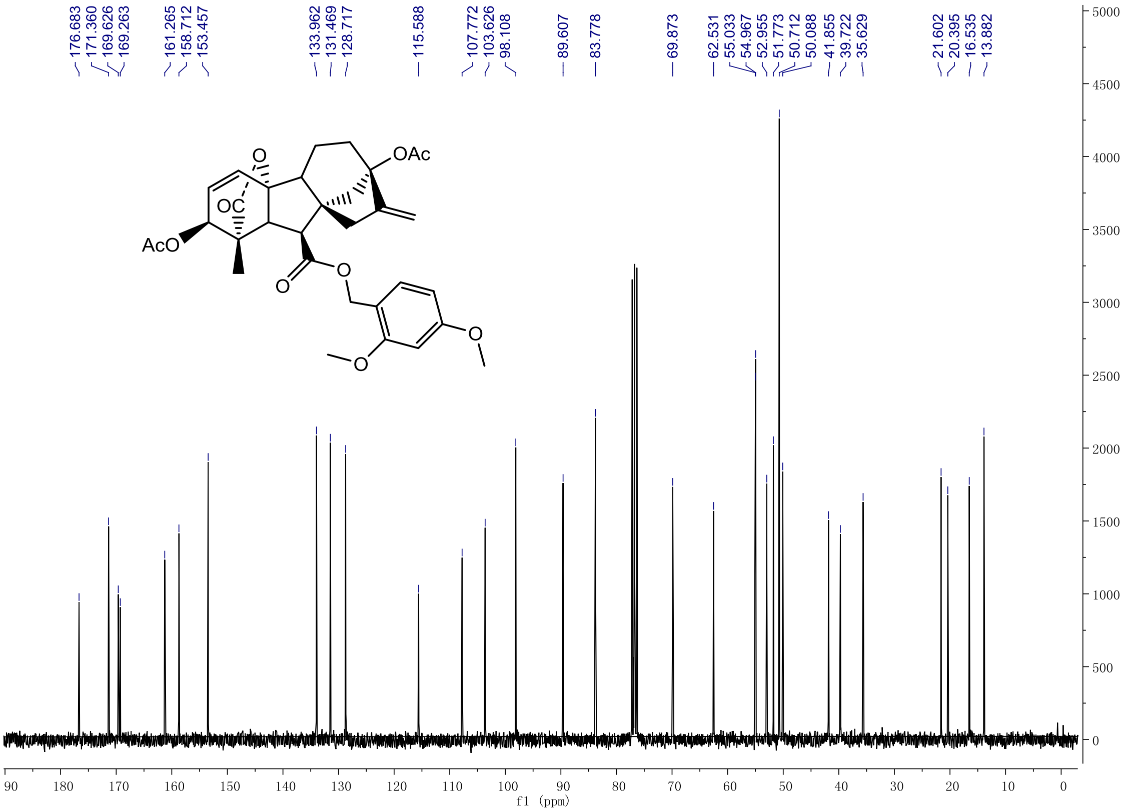


HRMS of compound **3.**

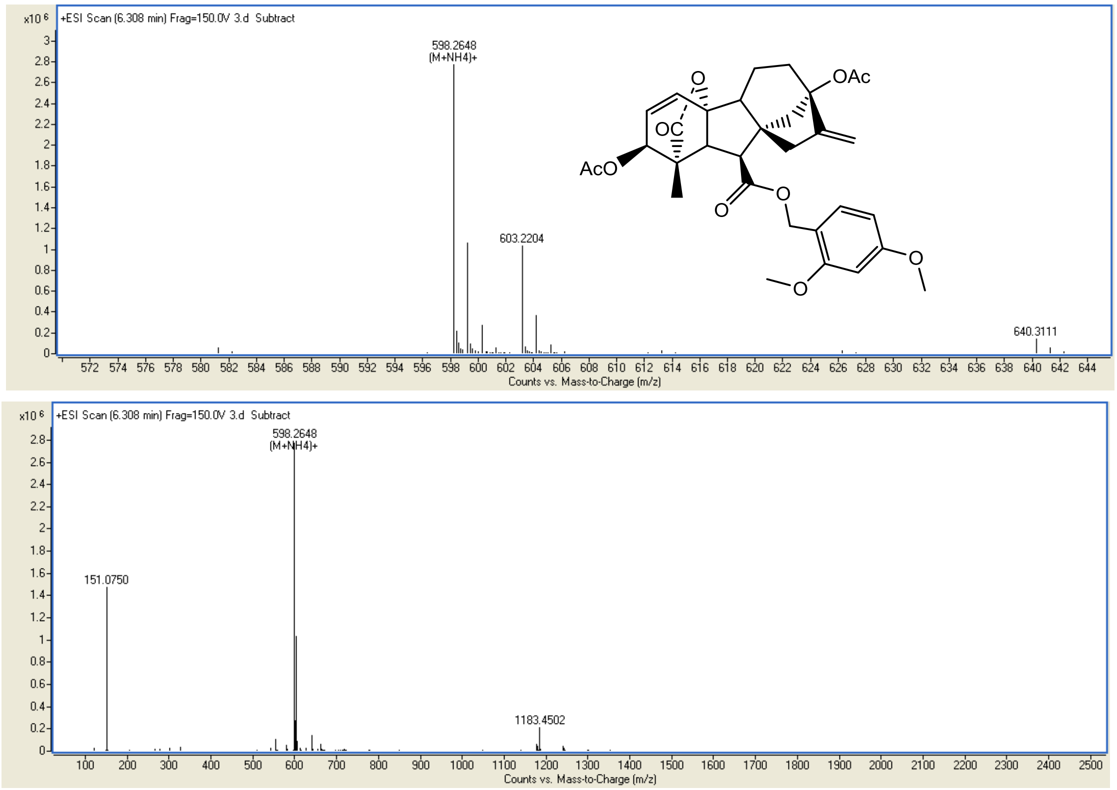

$^{1}$H-NMR spectrum of compound **4.**

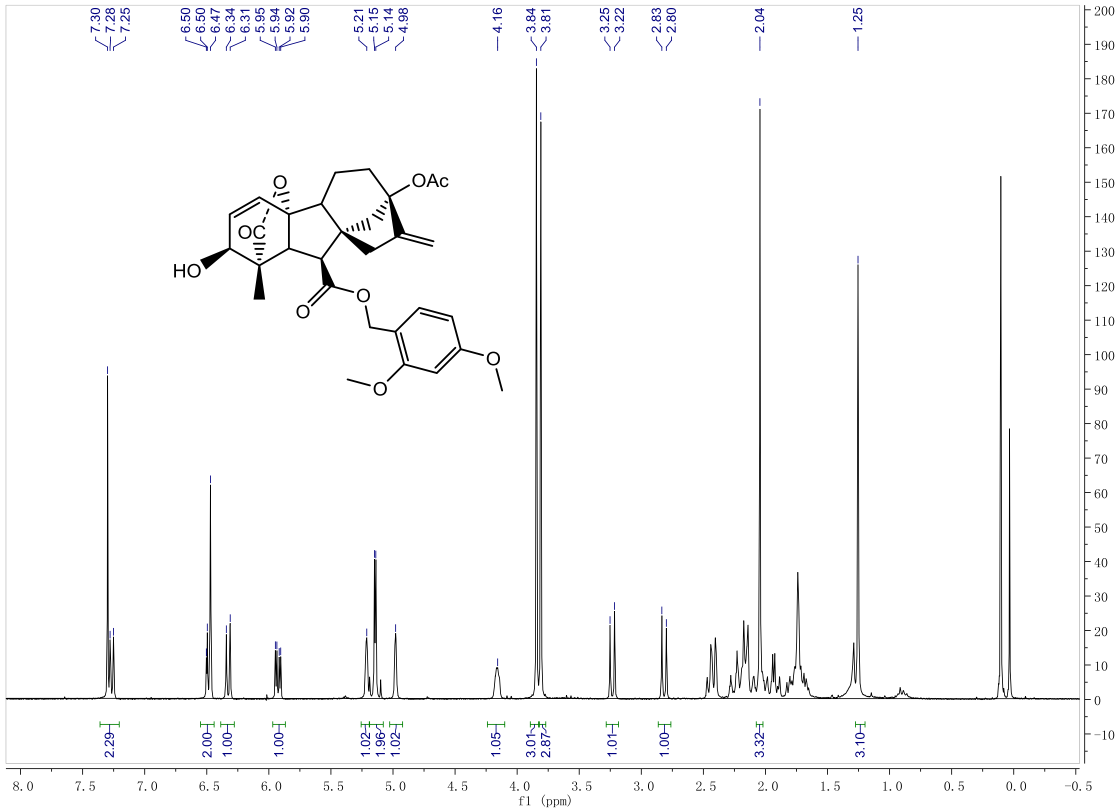


$^{13}$C-NMR spectrum of compound **4.**

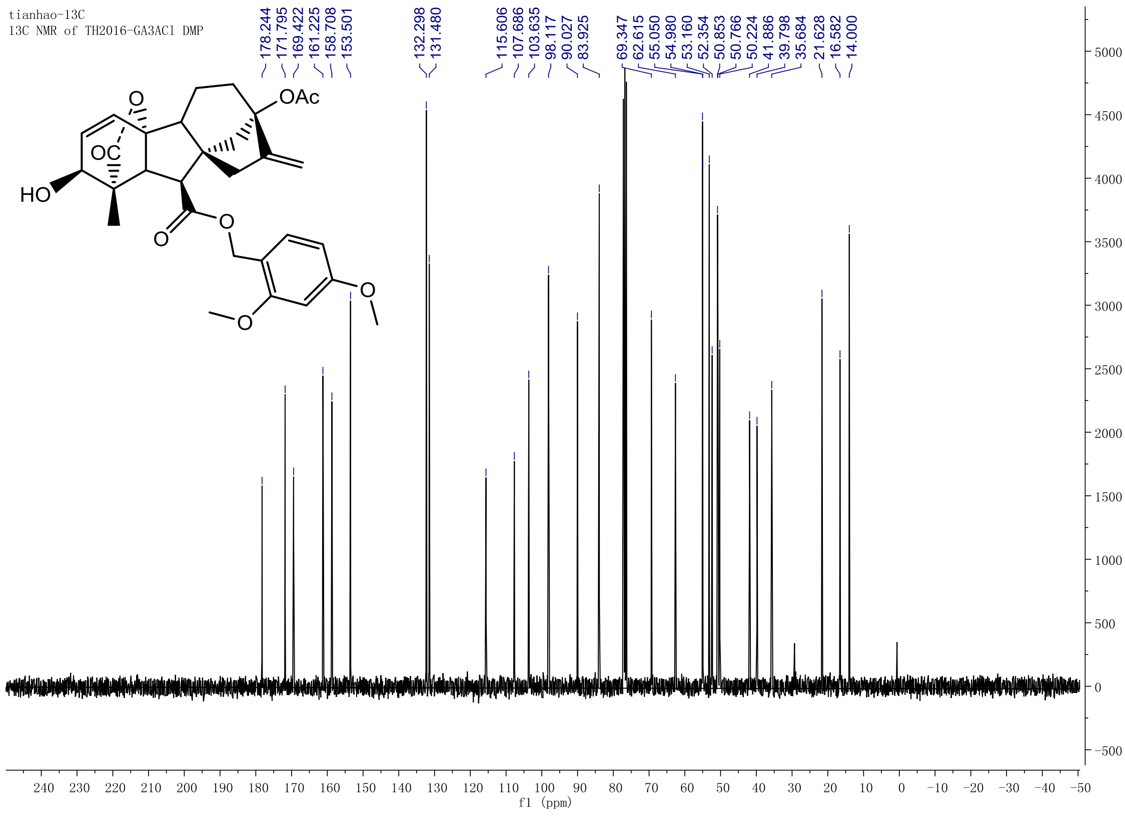

HRMS of compound **4.**

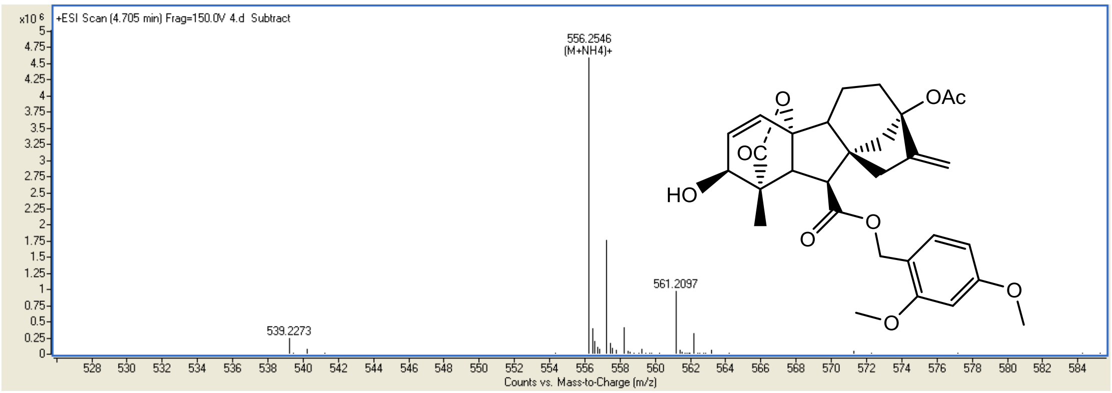


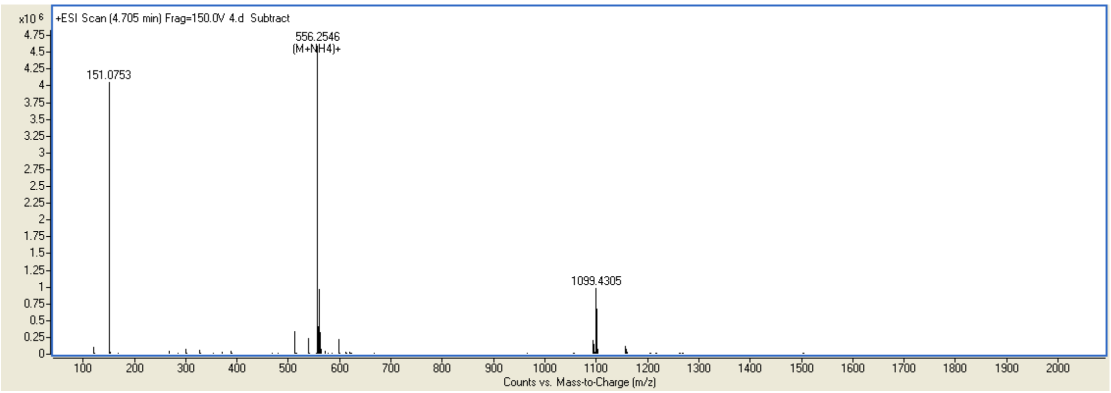


$^{1}$H-NMR spectrum of compound **5.**

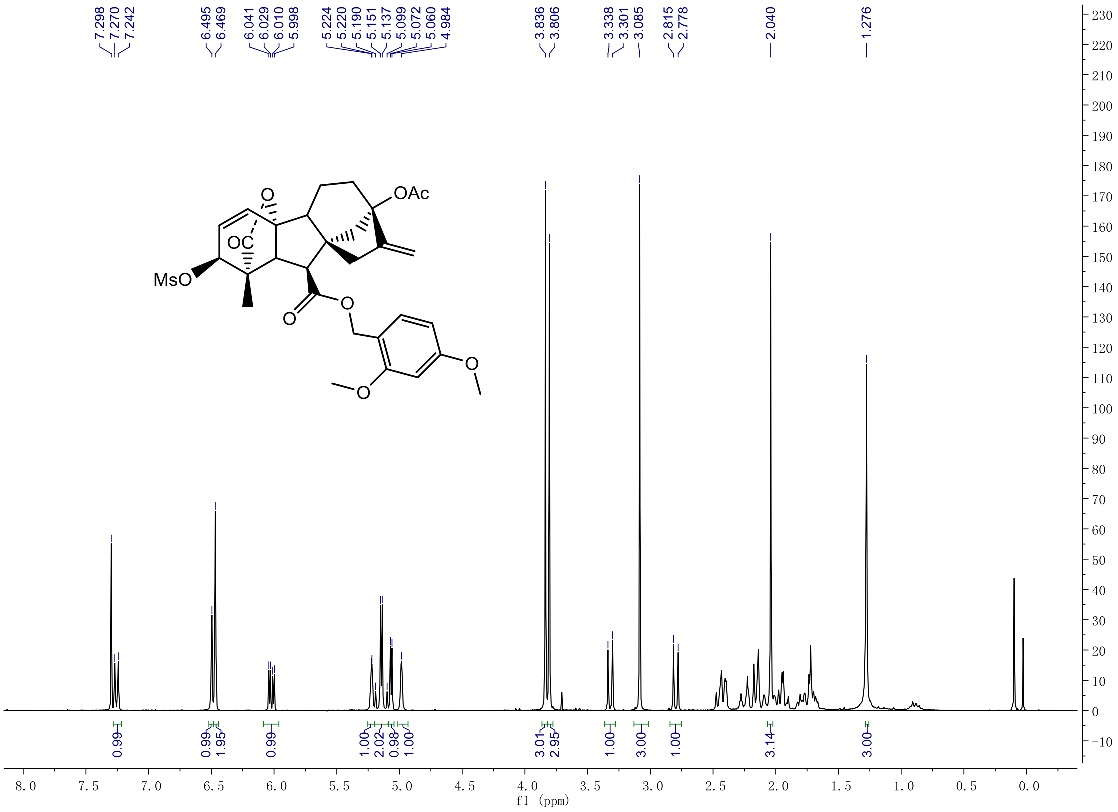

$^{13}$C-NMR spectrum of compound **5.**

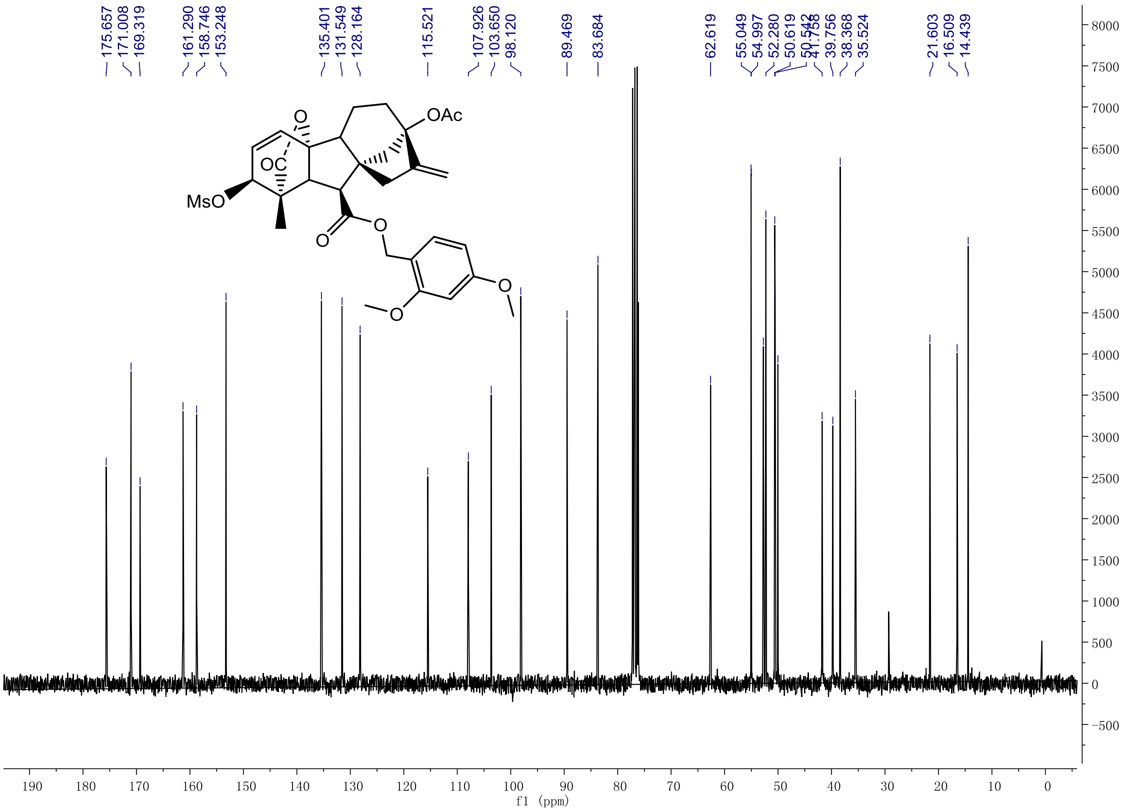


HRMS of compound **5.**

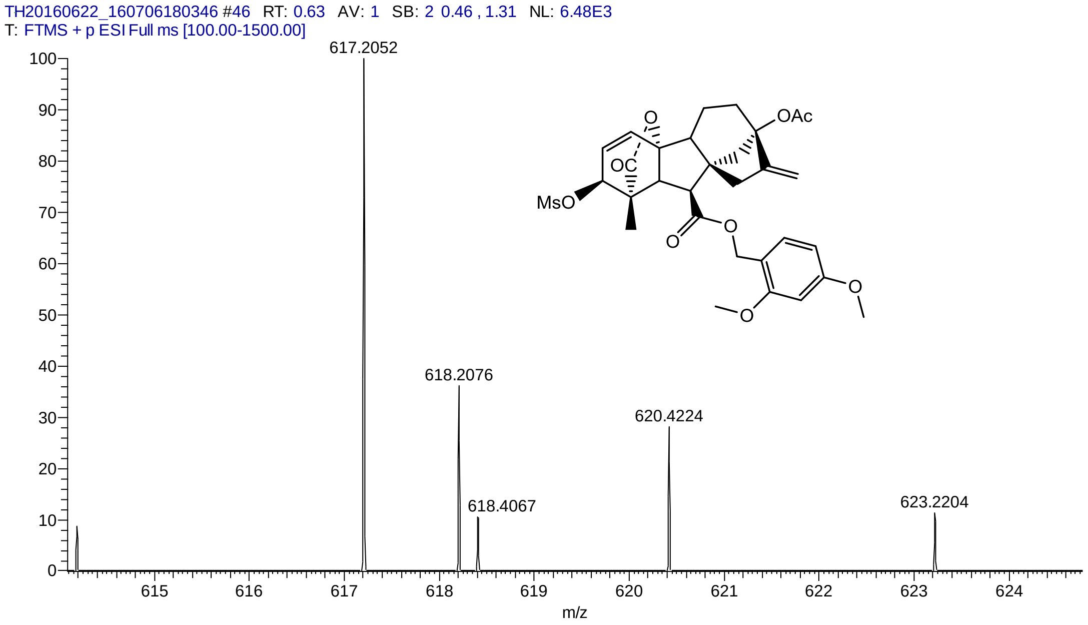

$^{1}$H-NMR spectrum of compound **6.**

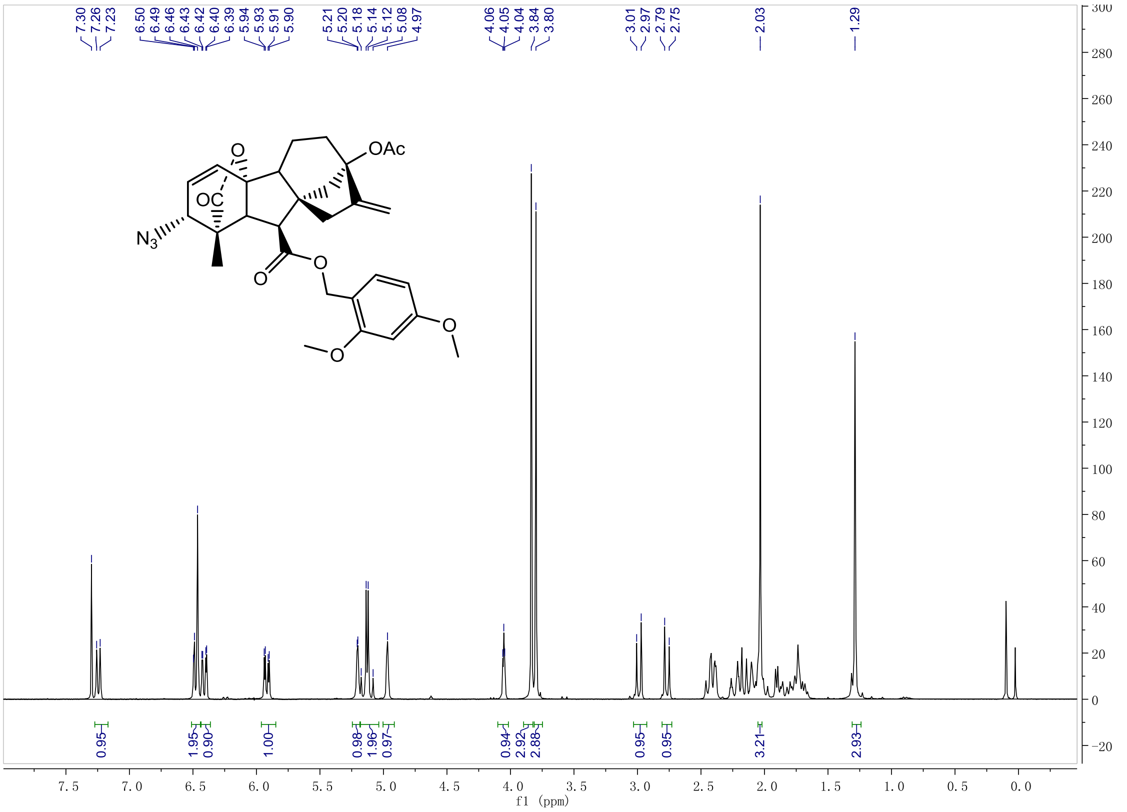


$^{13}$C-NMR spectrum of compound **6.**

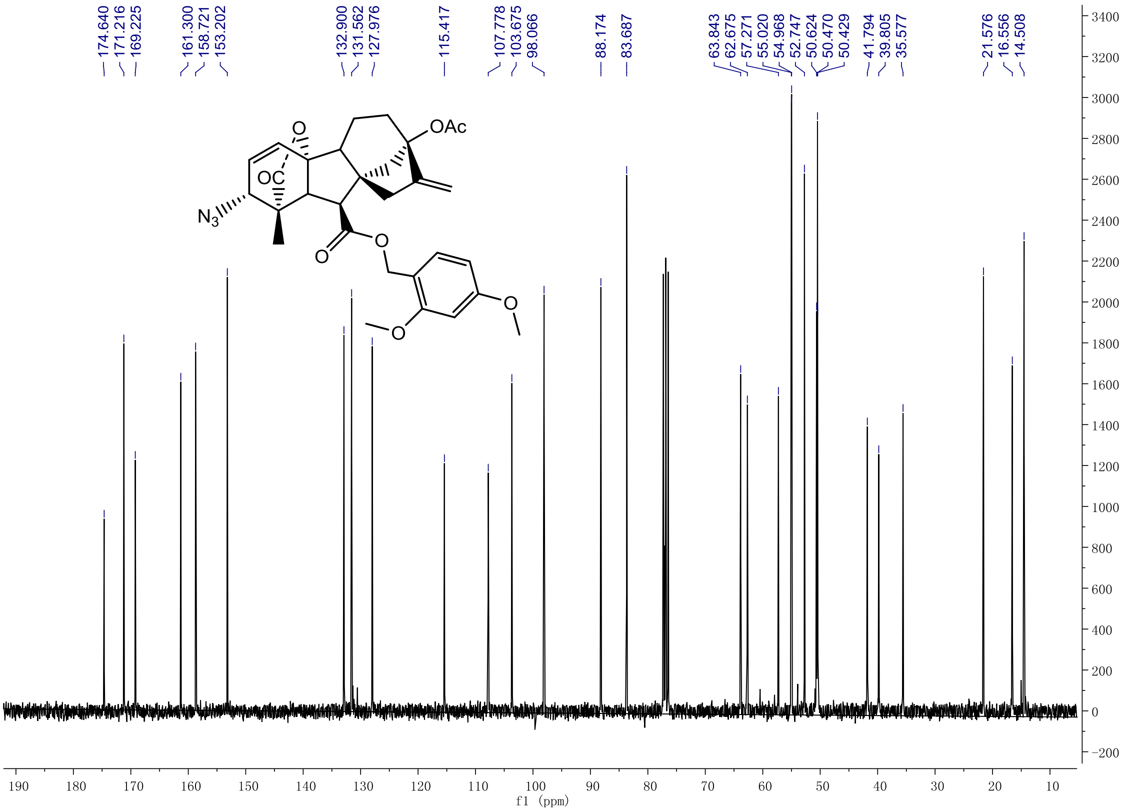

HRMS of compound **6.**

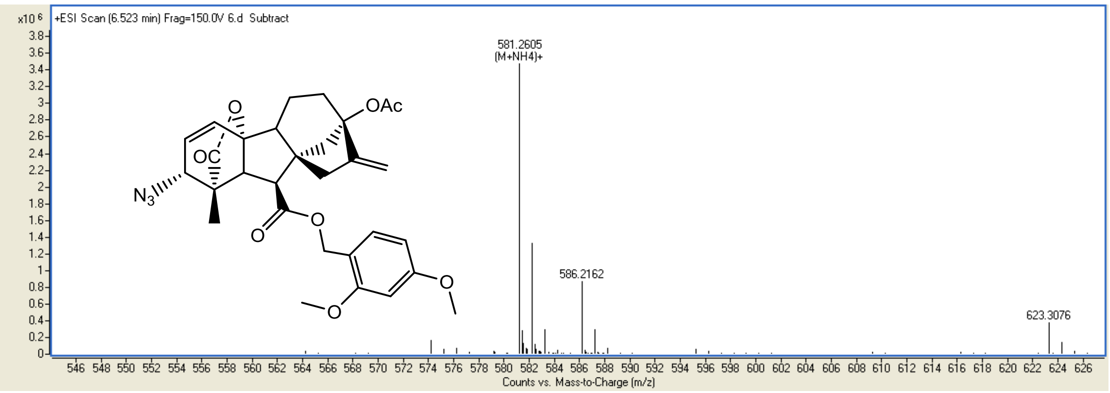


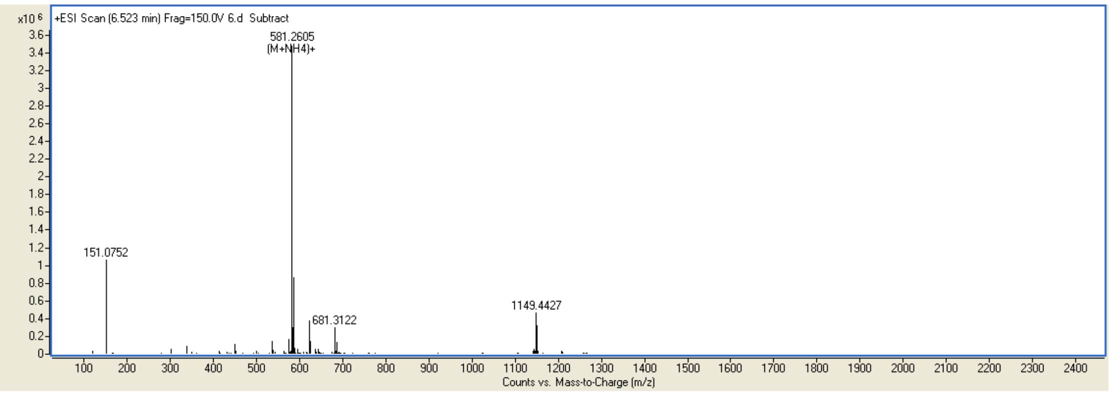


$^{1}$H-NMR spectrum of compound **8c**.

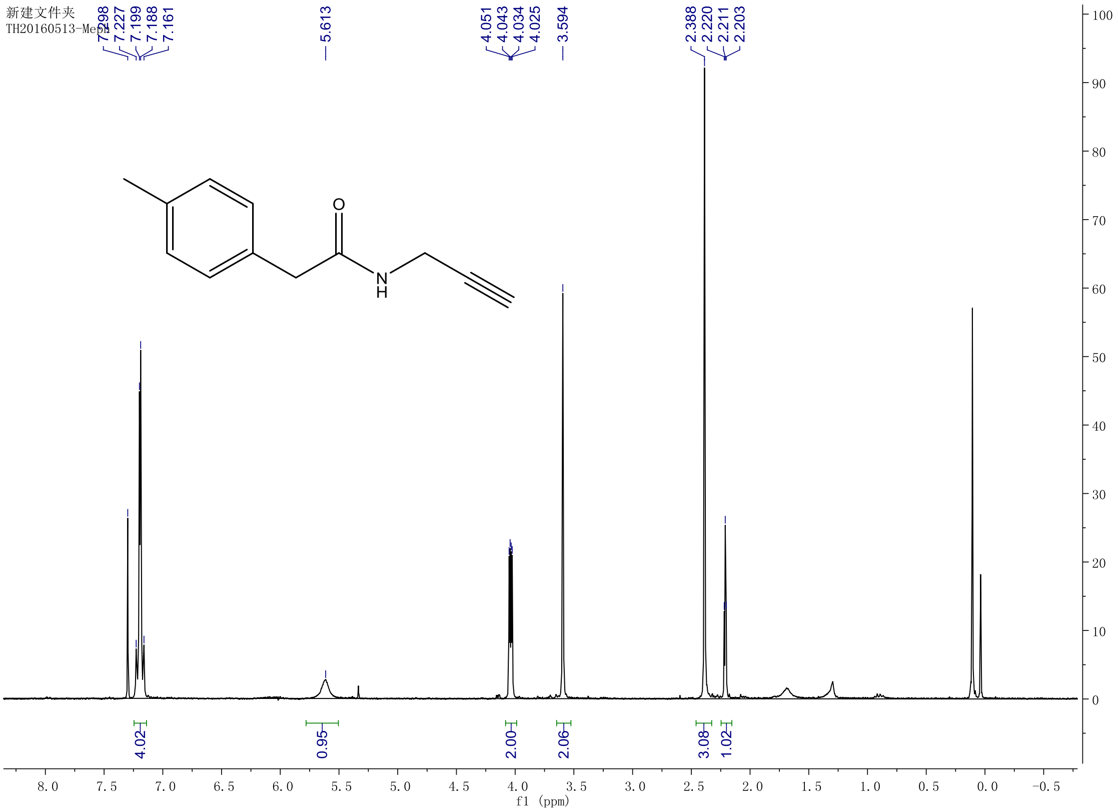

$^{13}$C-NMR spectrum of compound **8c**.

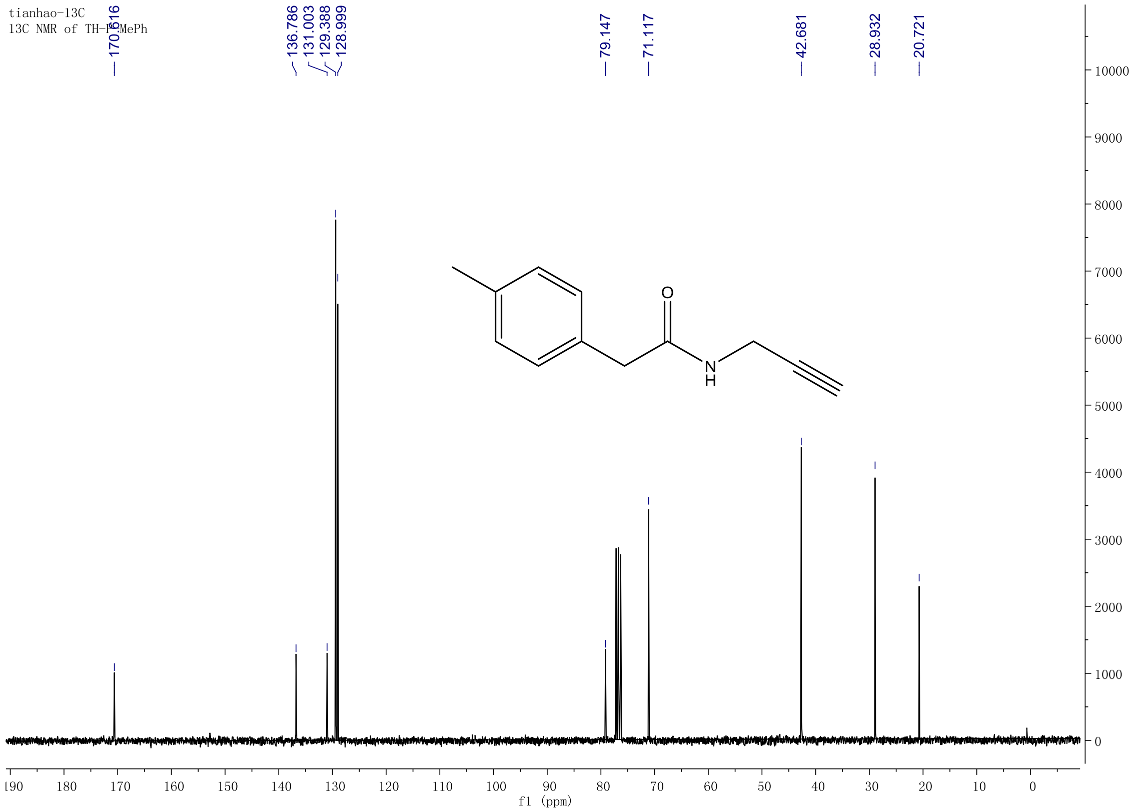


HRMS of compound **8c**.

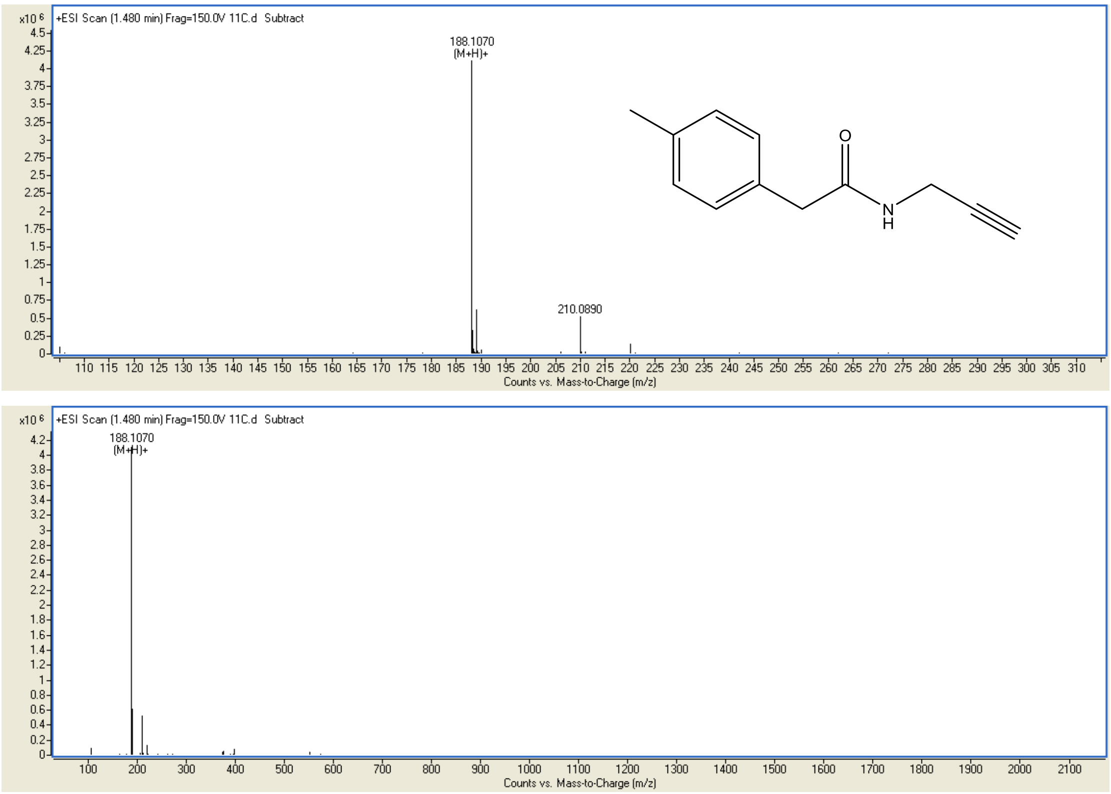

$^{1}$H-NMR spectrum of compound **8d**.

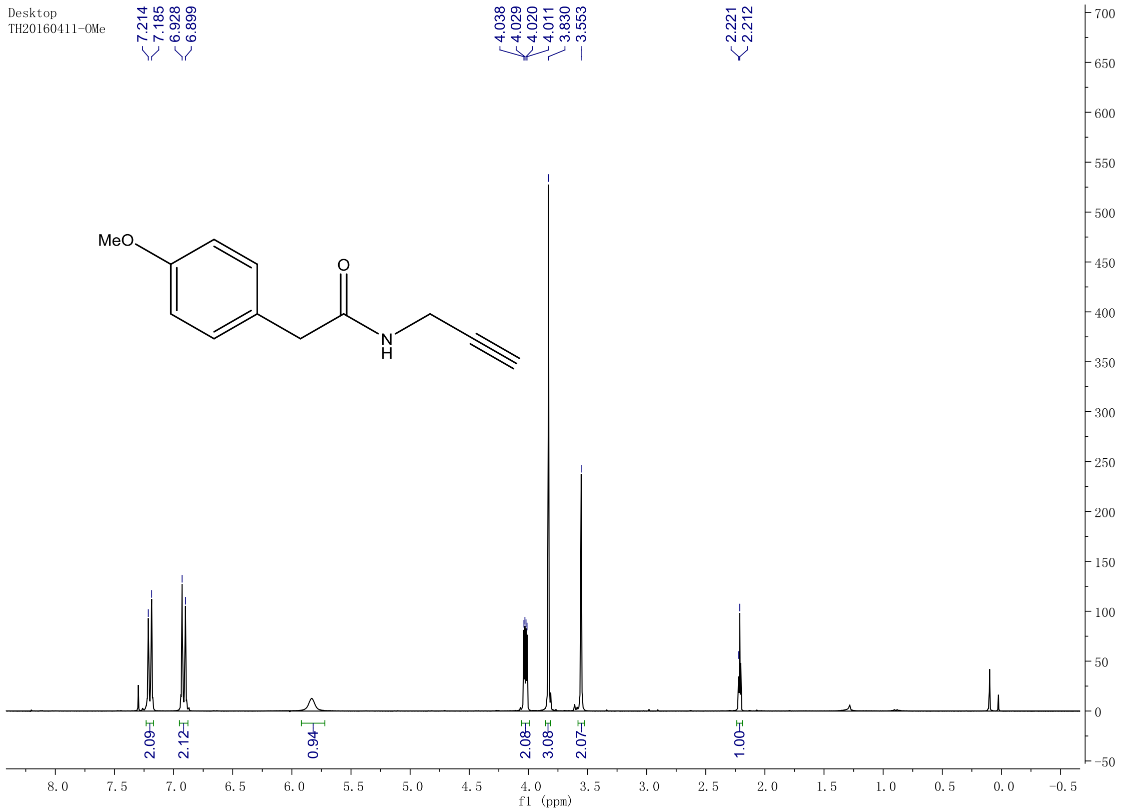


$^{13}$C-NMR spectrum of compound **8d**.

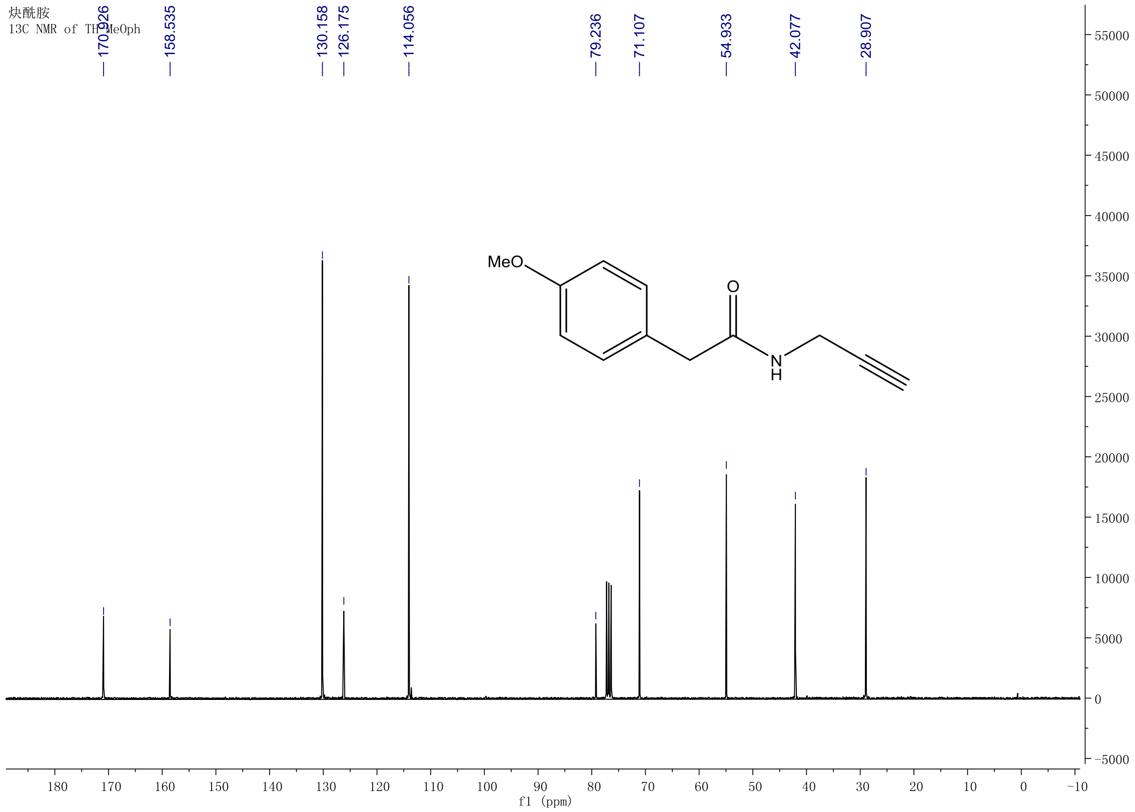

HRMS of compound **8d**.

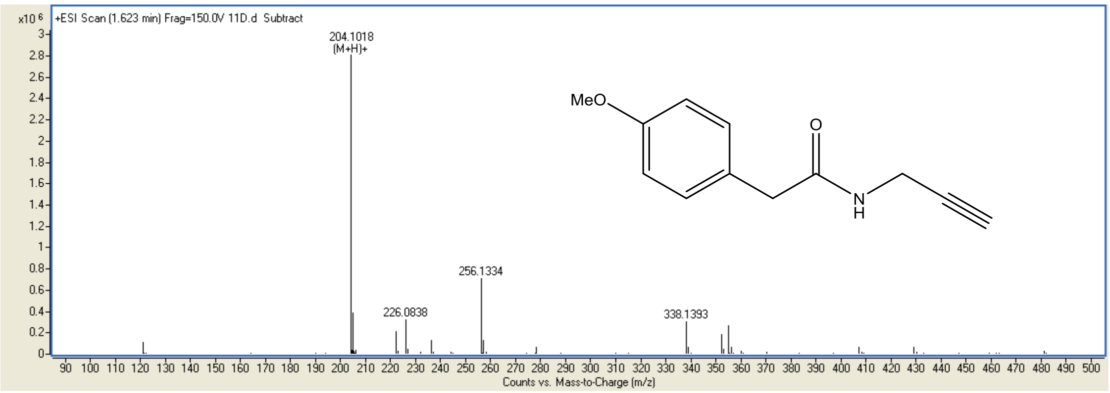


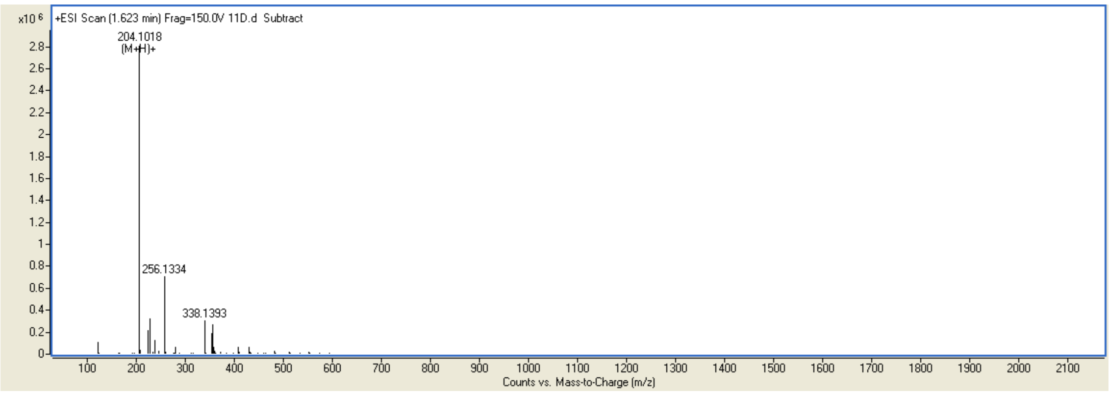


$^{1}$H-NMR spectrum of compound **8f**.

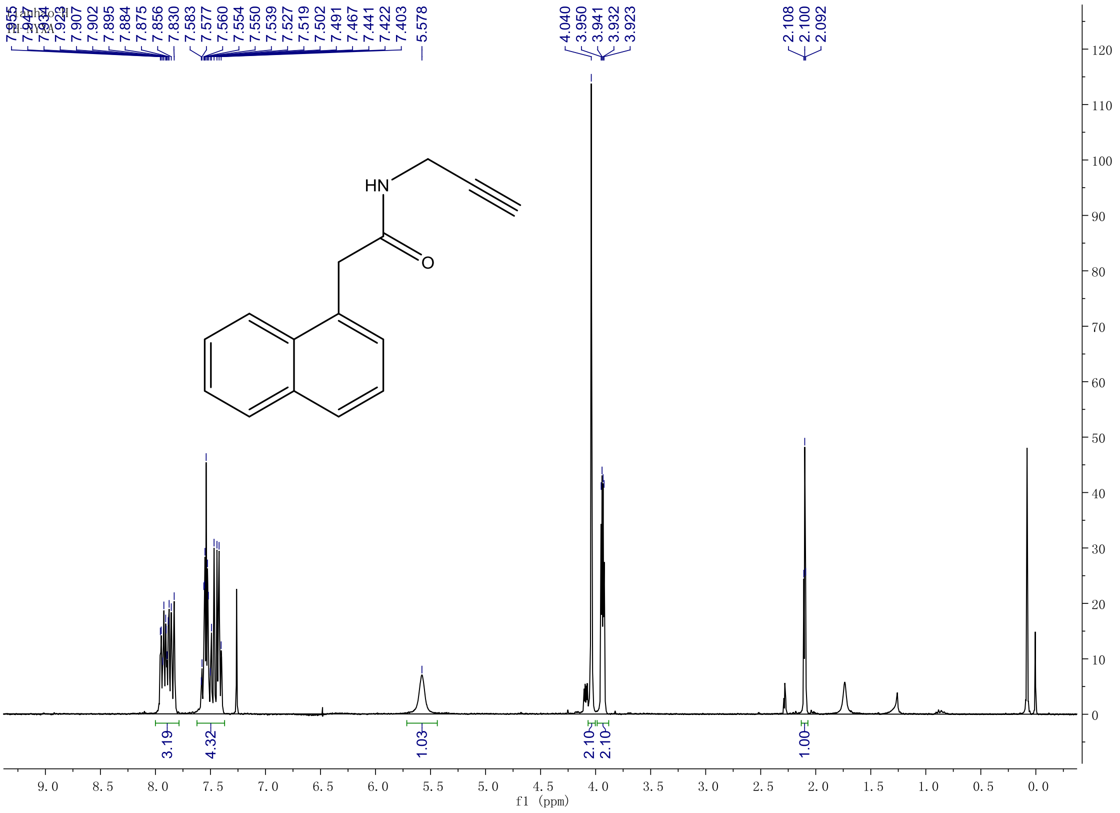

$^{13}$C-NMR spectrum of compound **8f**.

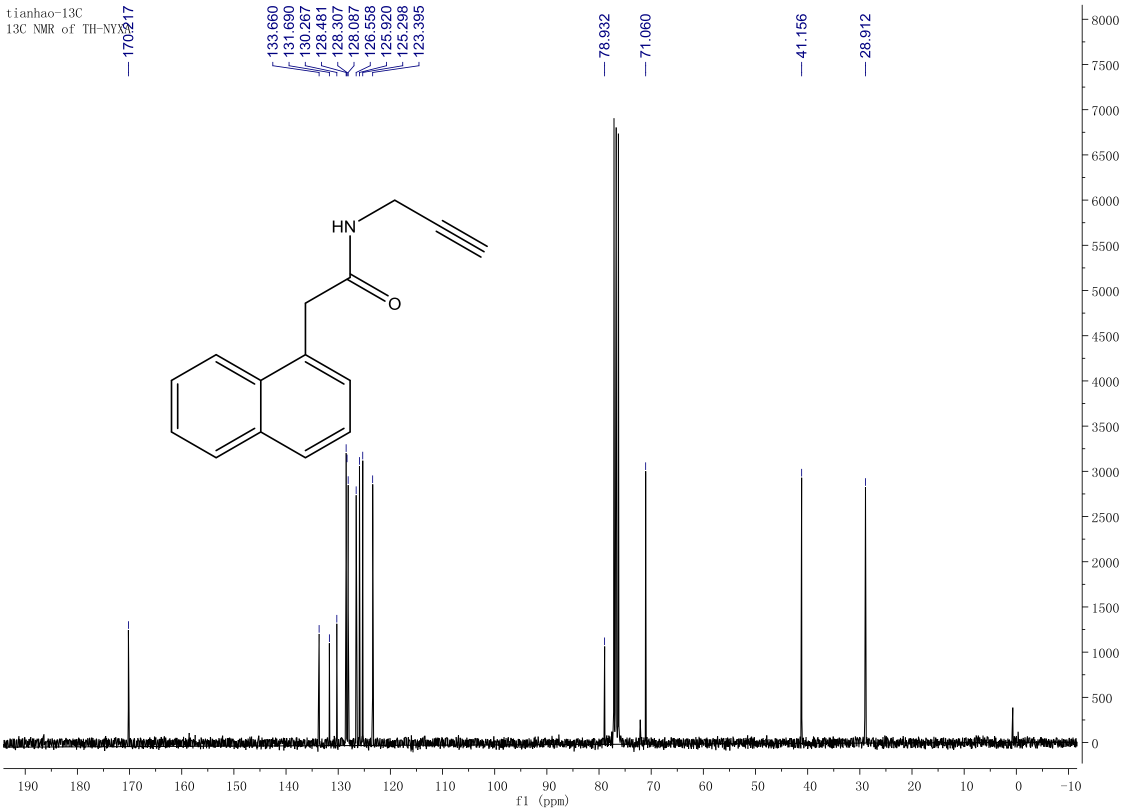


HRMS of compound **8f**.

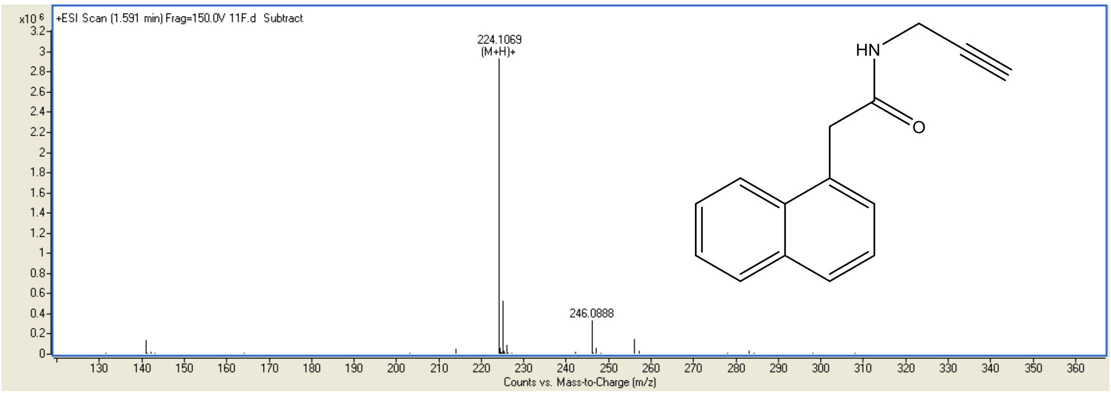


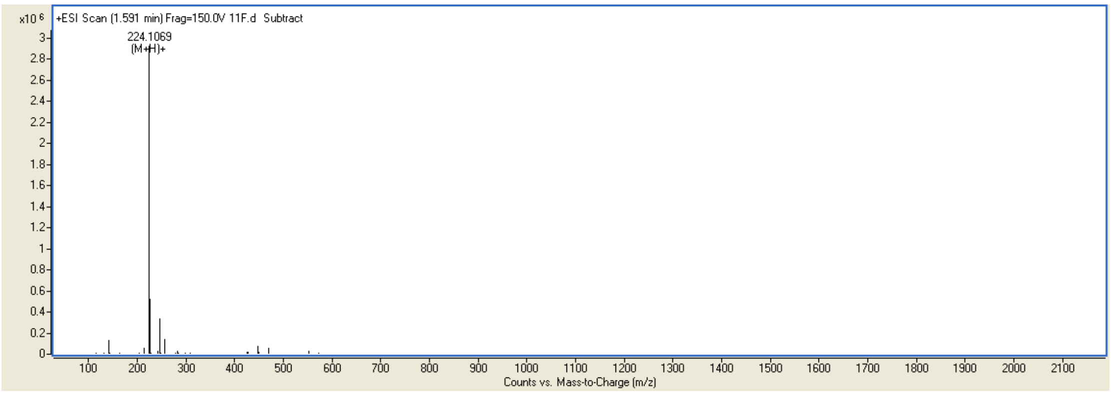

$^{1}$H-NMR spectrum of compound **8g**.

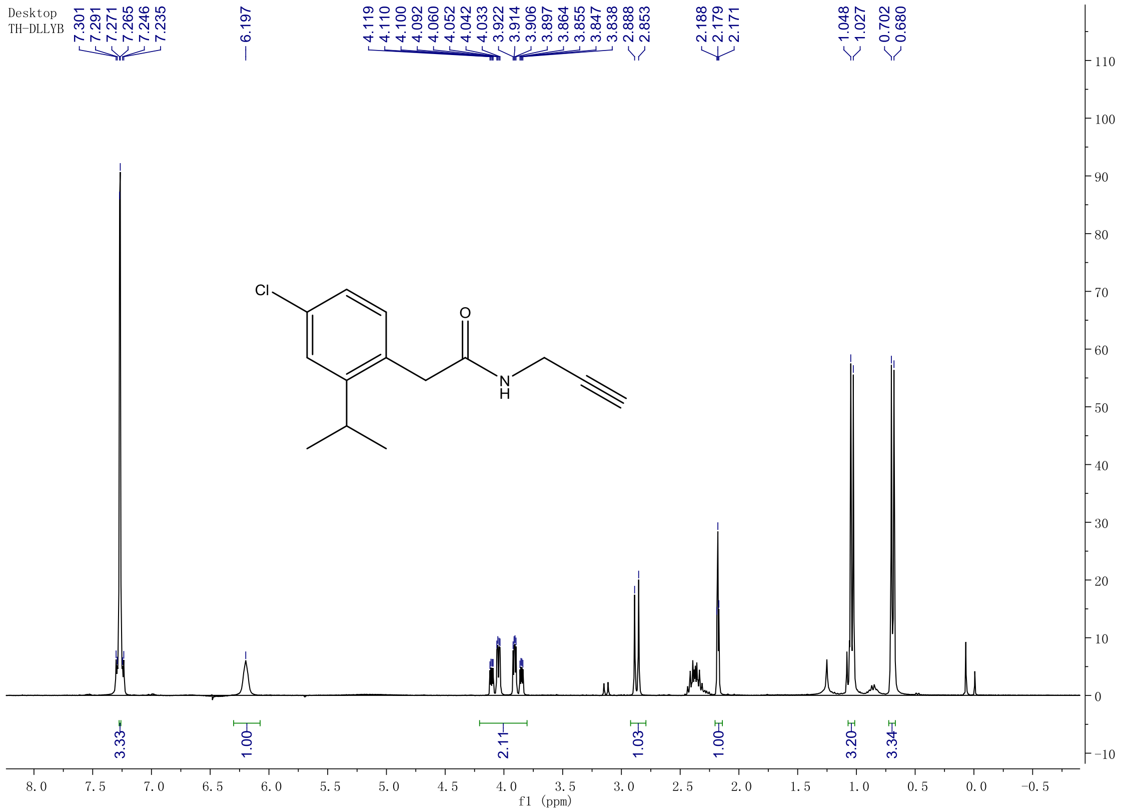


$^{13}$C-NMR spectrum of compound **8g**.

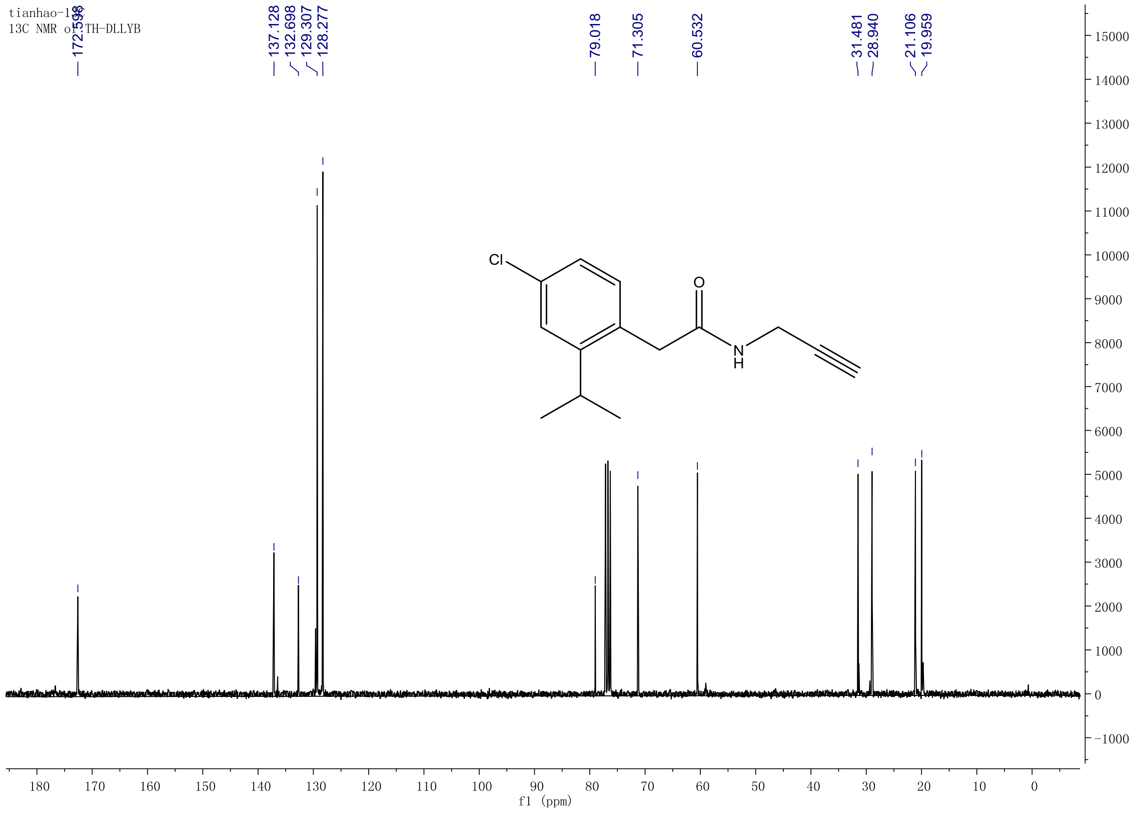

HRMS of compound **8g**.

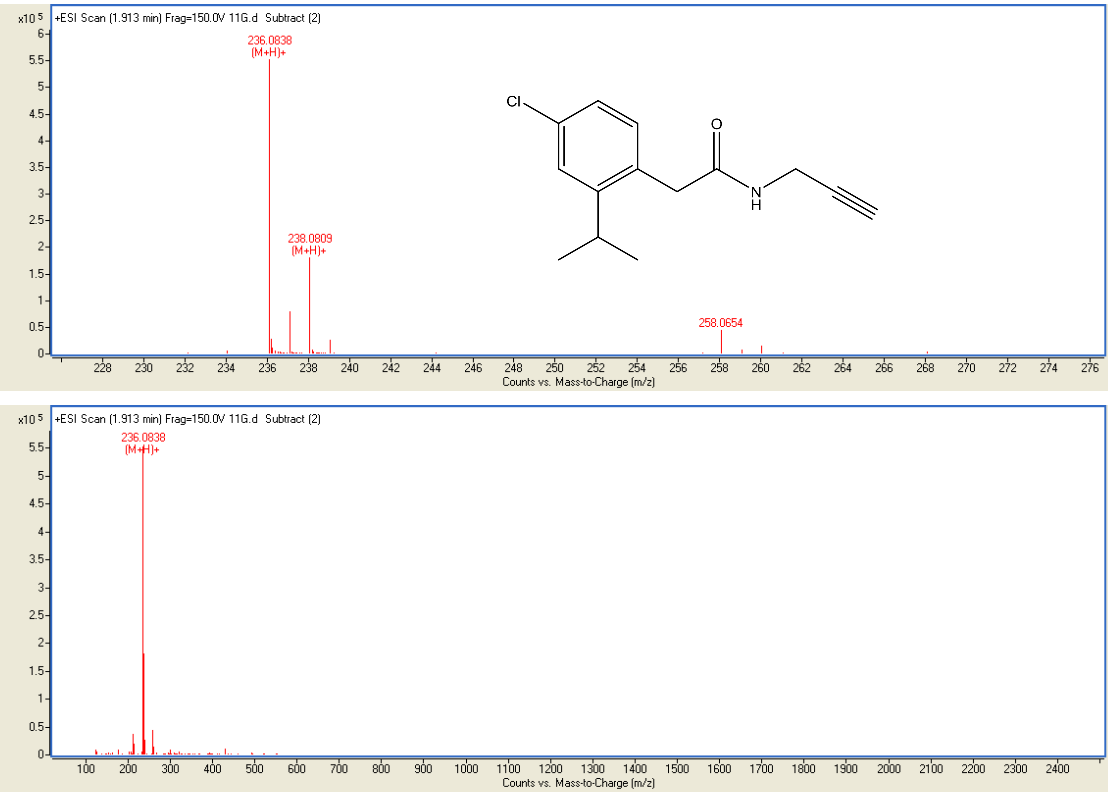


$^{1}$H-NMR spectrum of compound **8i**.

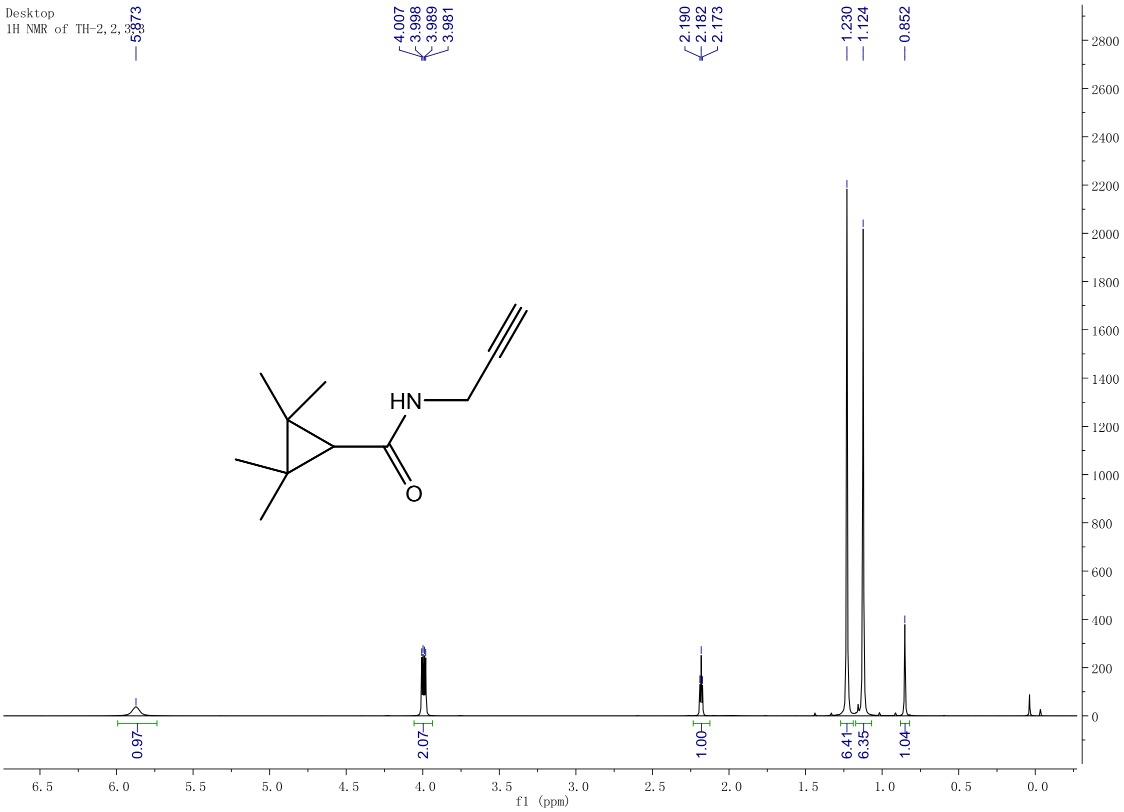

$^{13}$C-NMR spectrum of compound **8i**.

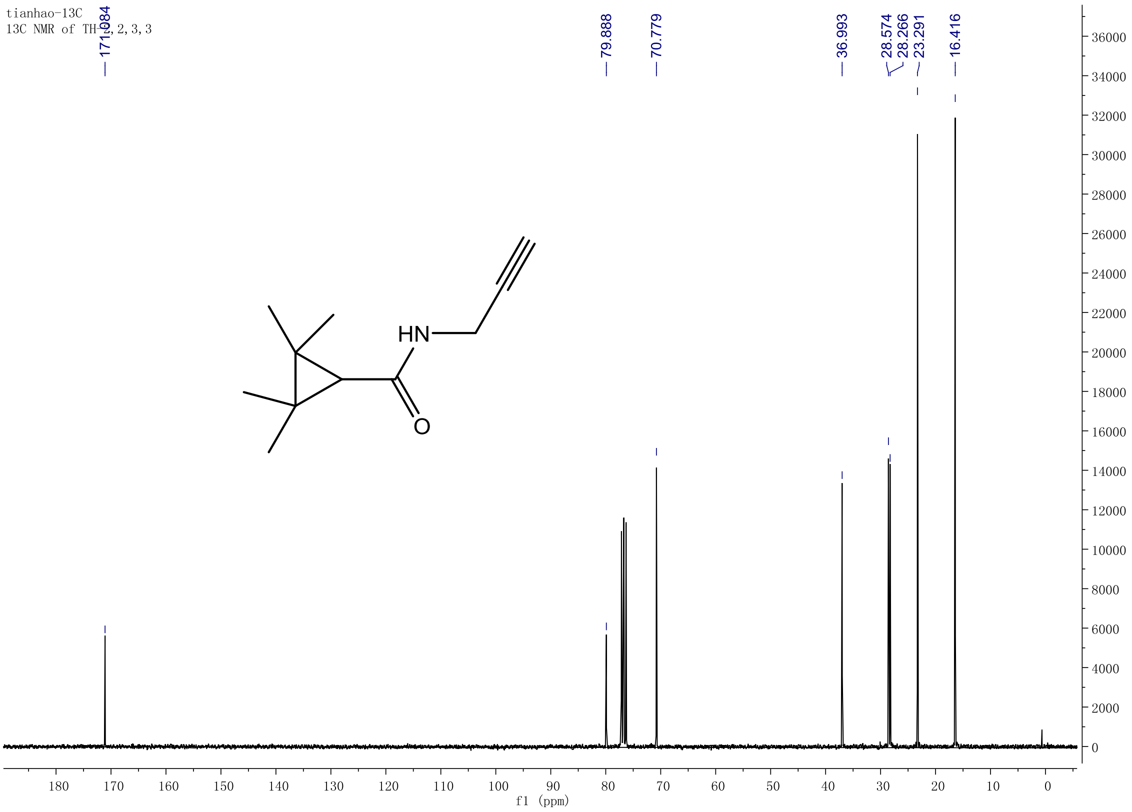


HRMS of compound **8i**.

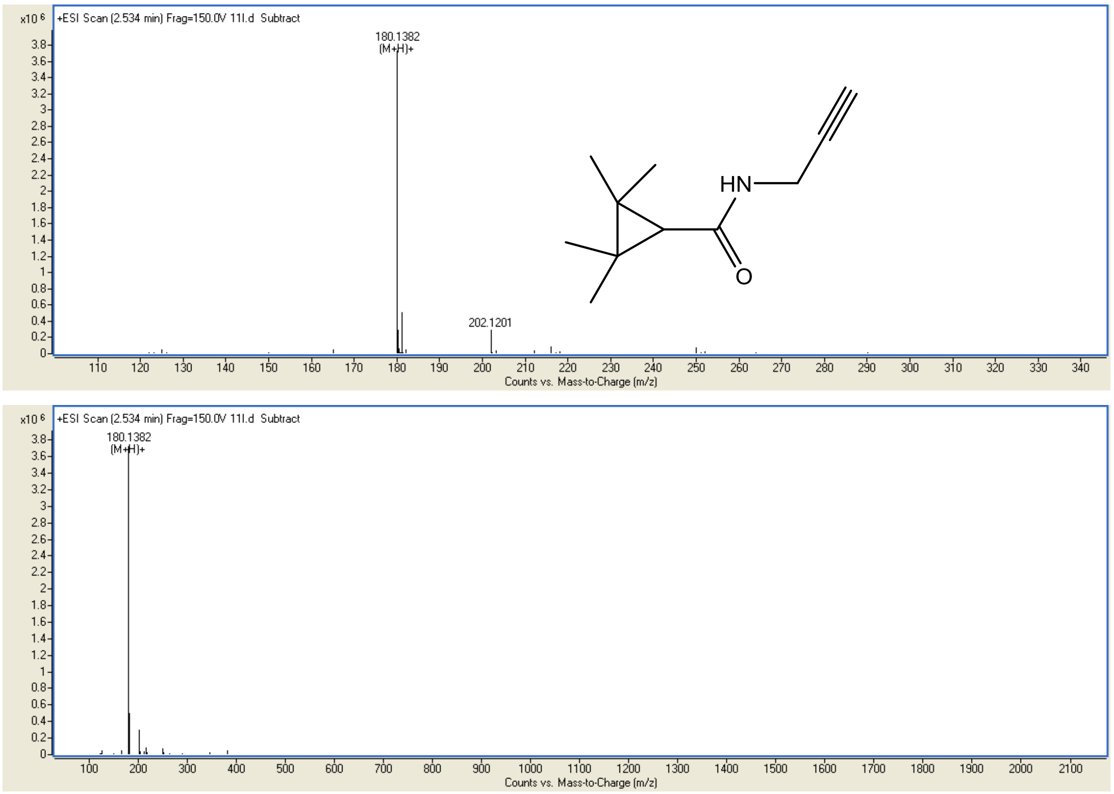

$^{1}$H-NMR spectrum of compound **8n**.

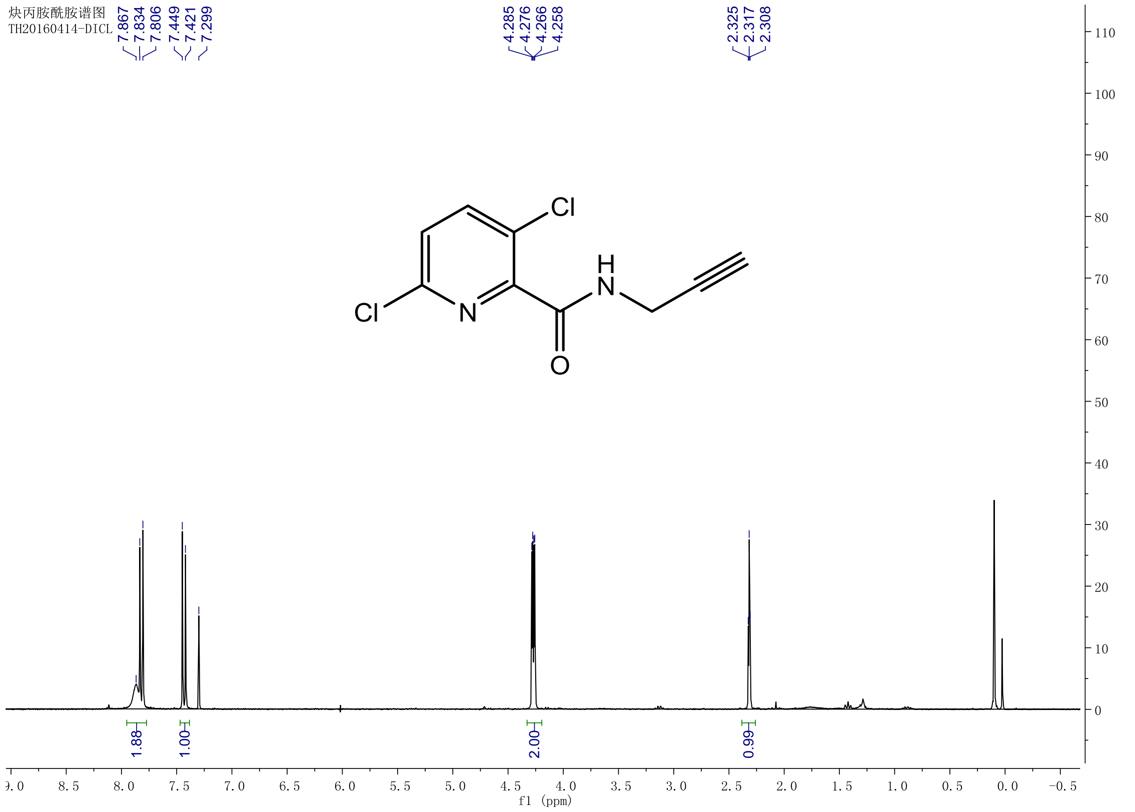


$^{13}$C-NMR spectrum of compound **8n**.

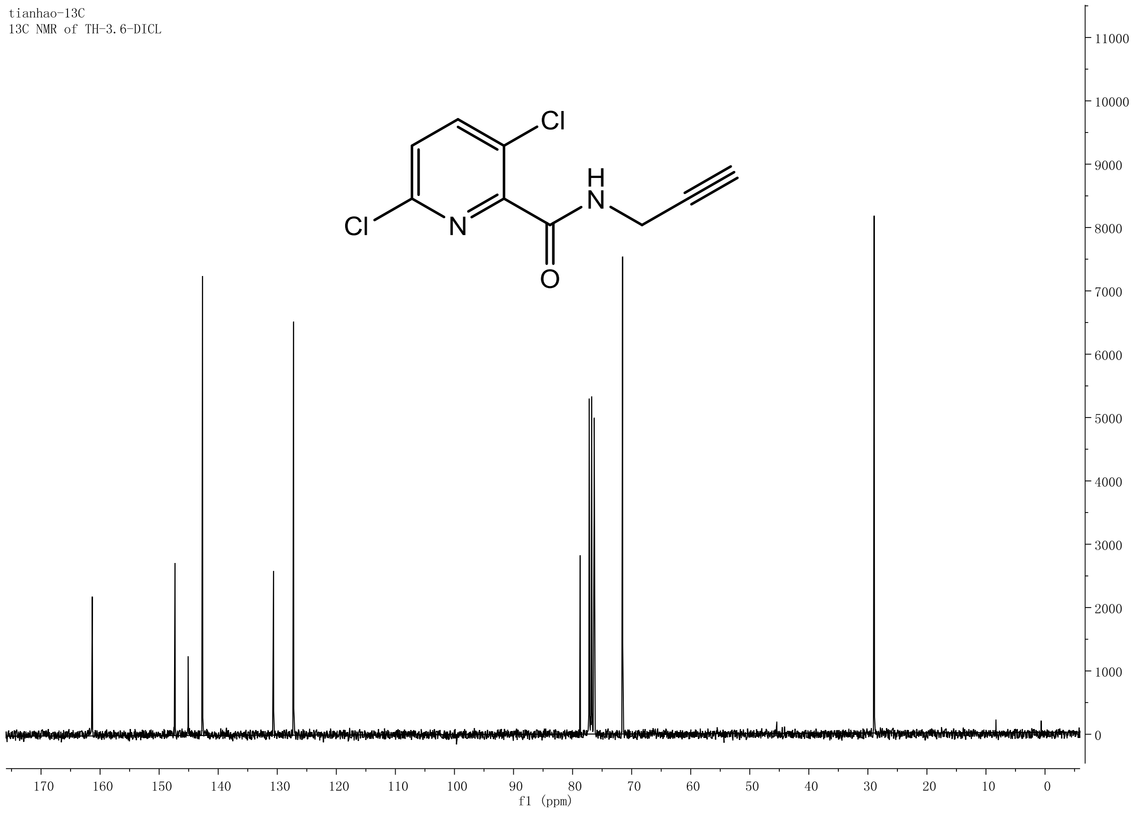

HRMS of compound **8n**.

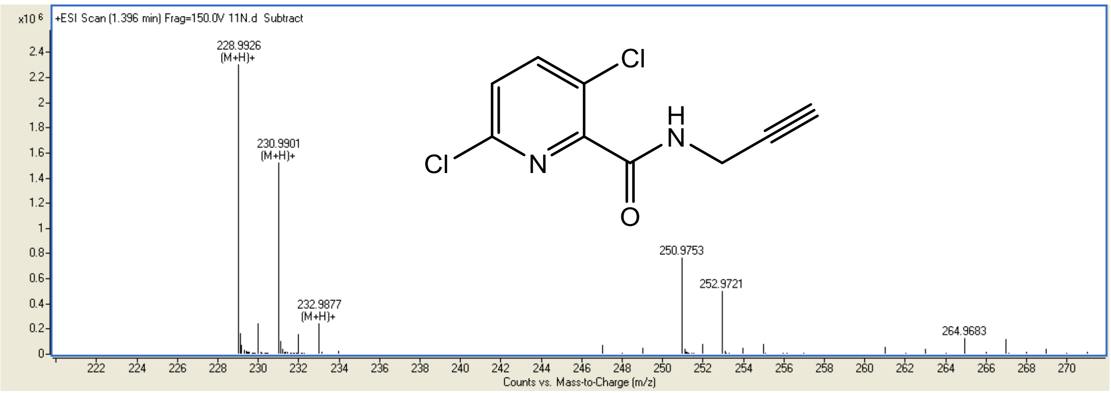


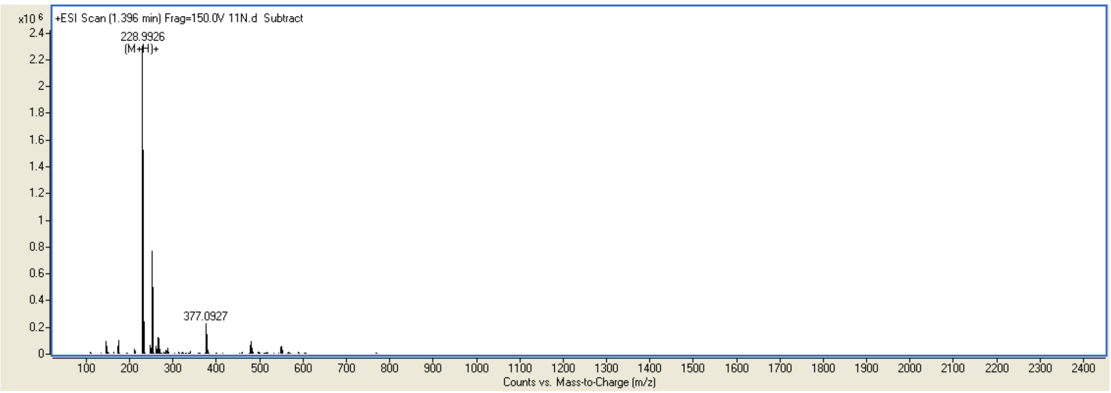


$^{1}$H-NMR spectrum of compound **9a.**

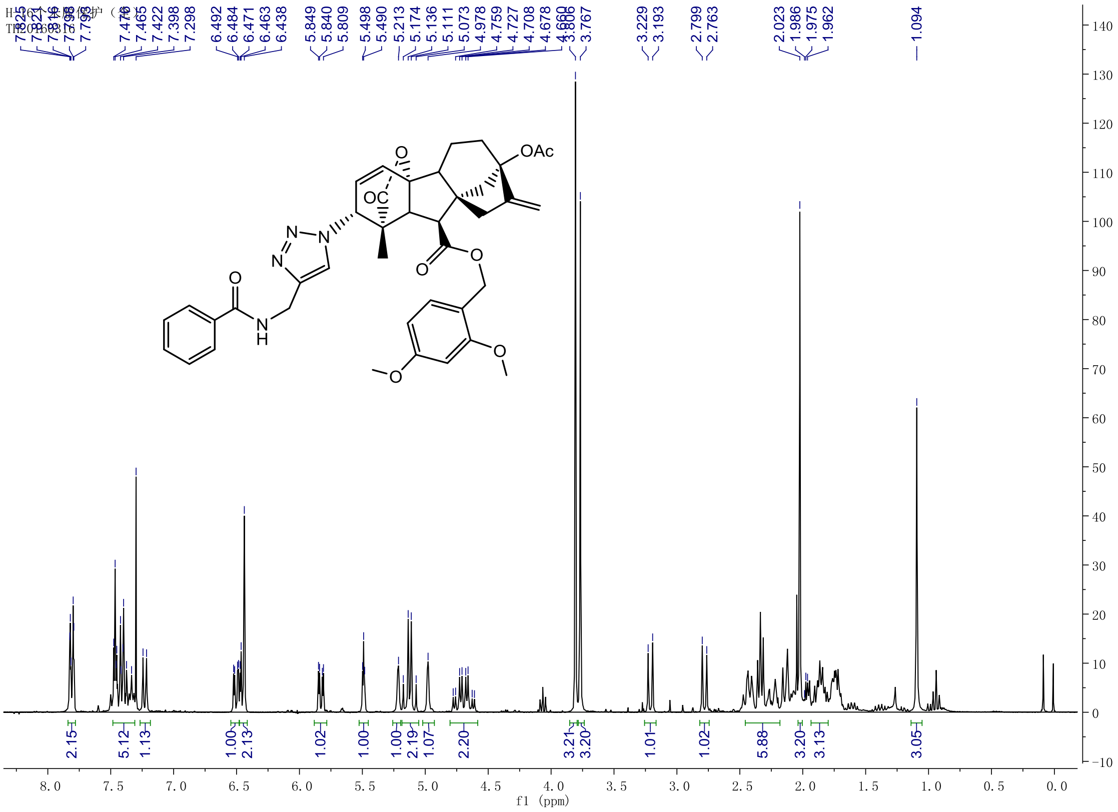

$^{13}$C-NMR spectrum of compound **9a.**

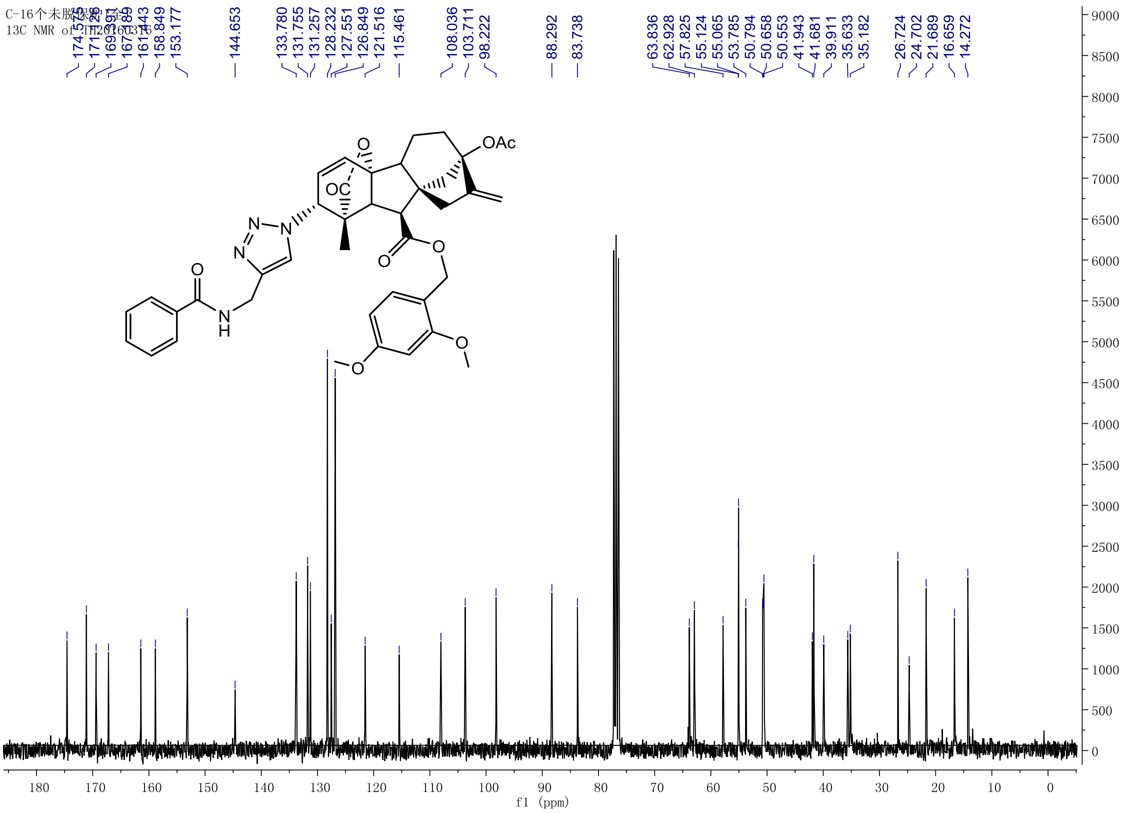


HRMS of compound **9a.**

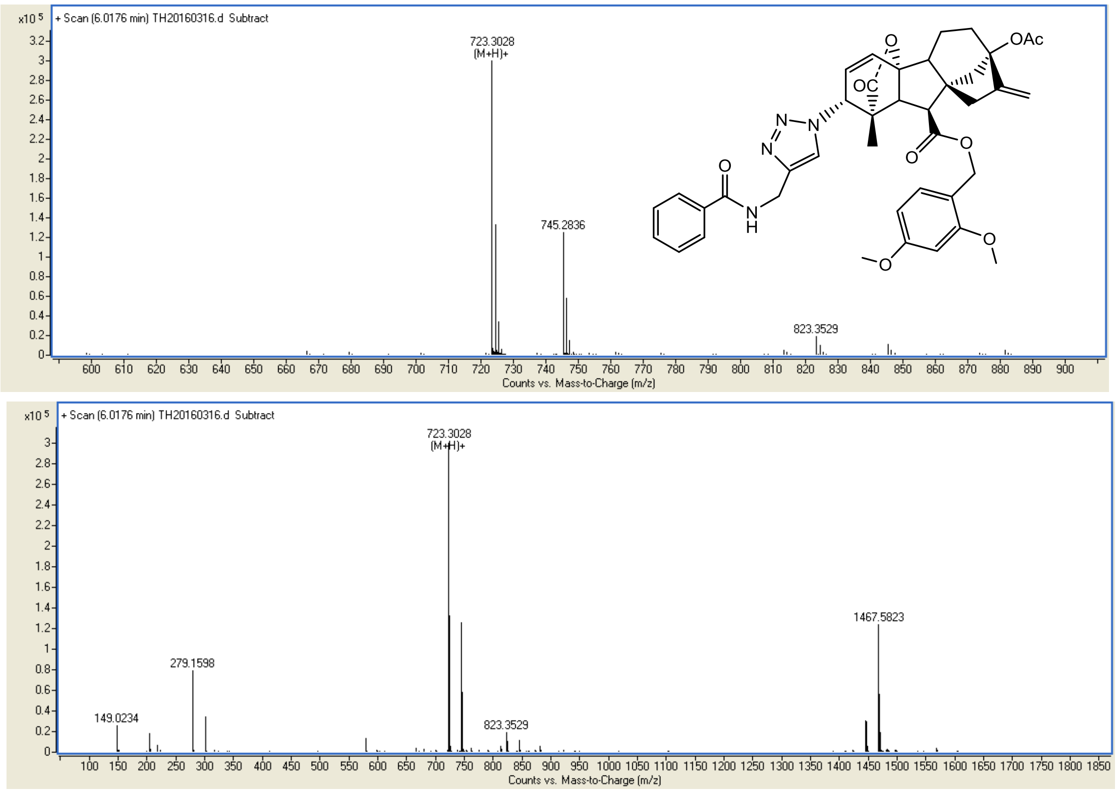

$^{1}$H-NMR spectrum of compound **9b.**

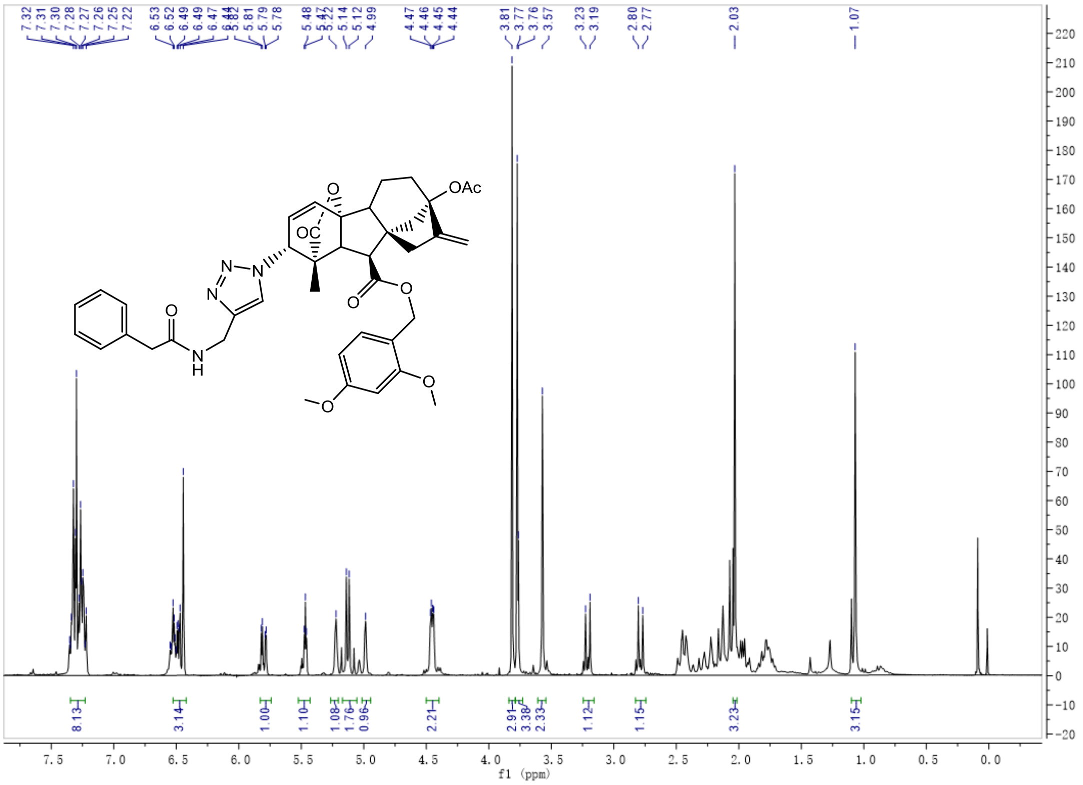


$^{13}$C-NMR spectrum of compound **9b.**

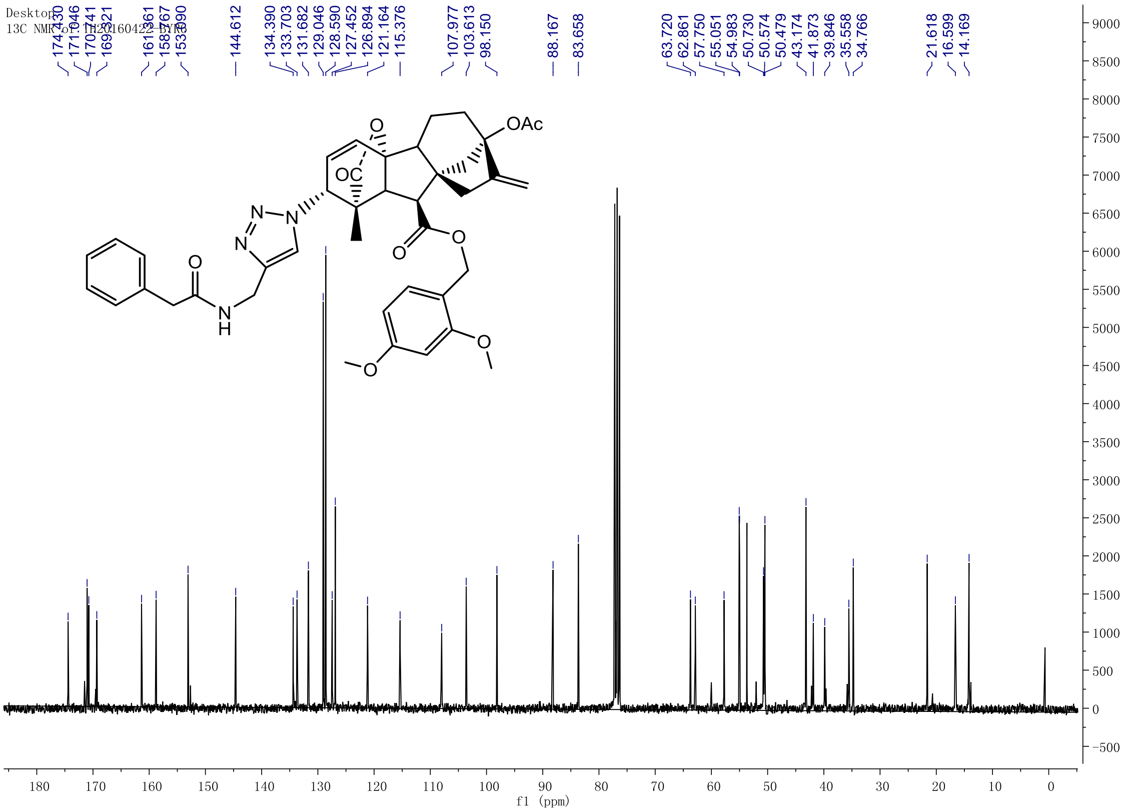

HRMS of compound **9b.**

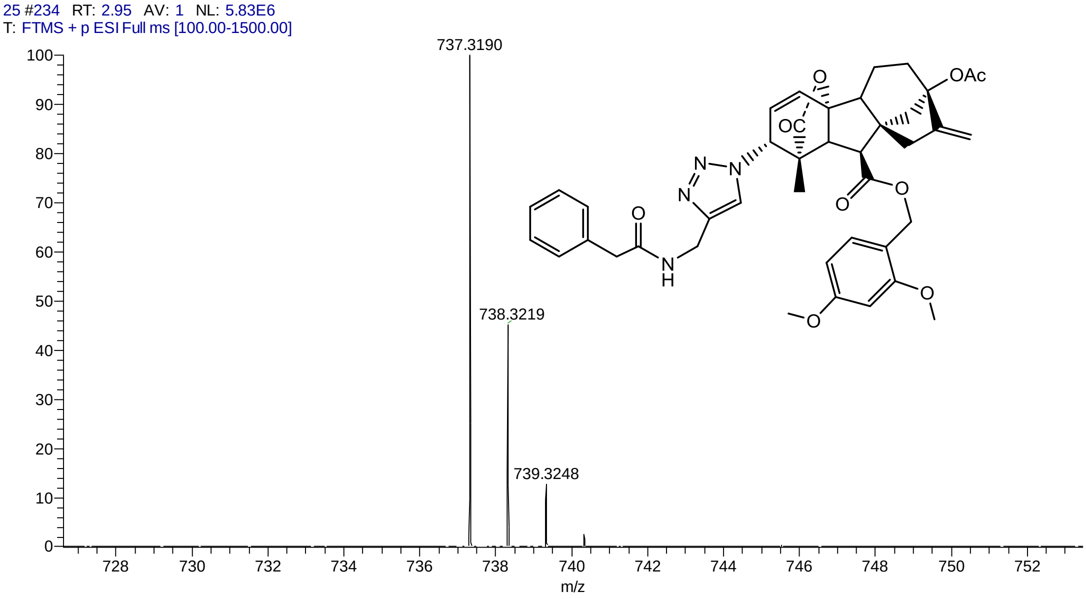


$^1$H-NMR spectrum of compound **9c.**

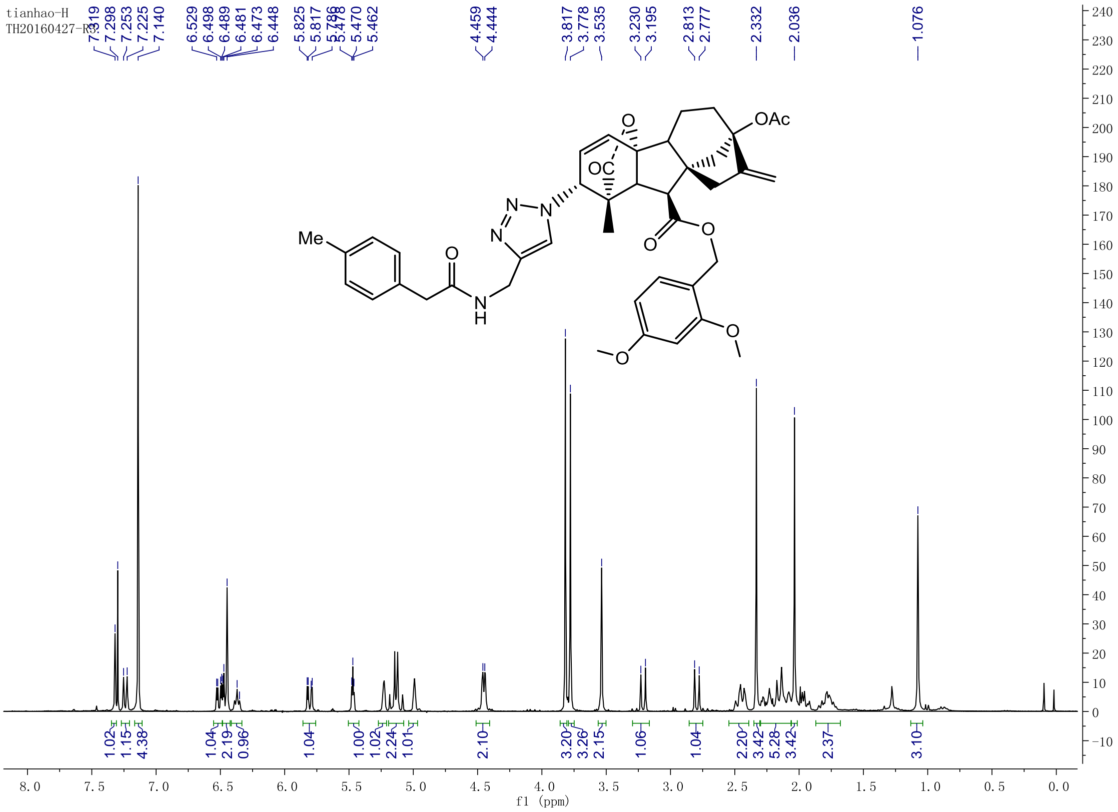

$^{13}$C-NMR spectrum of compound **9c.**

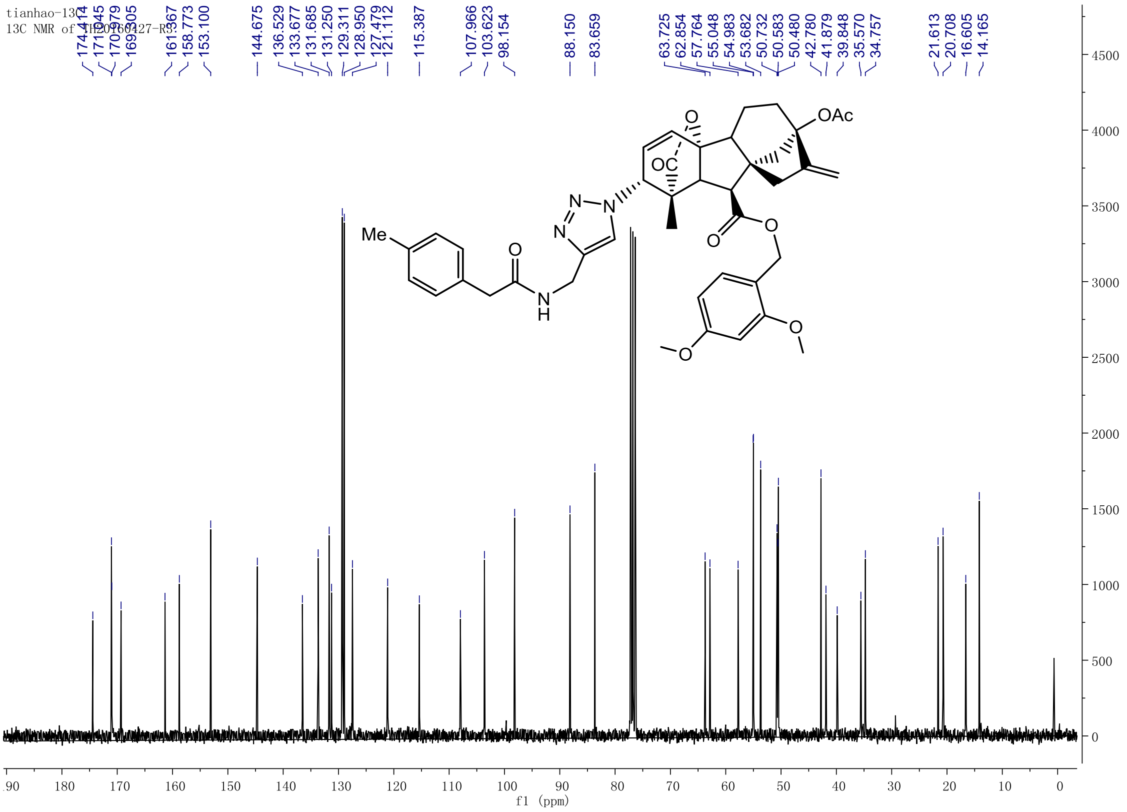


HRMS of compound **9c.**

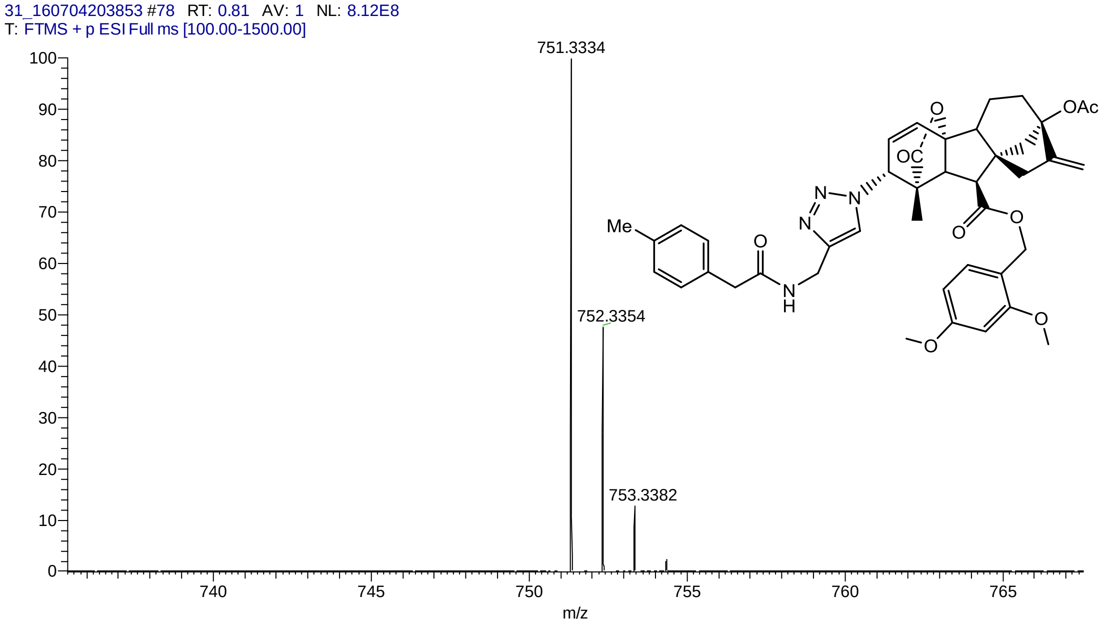

$^{1}$H-NMR spectrum of compound **9d.**

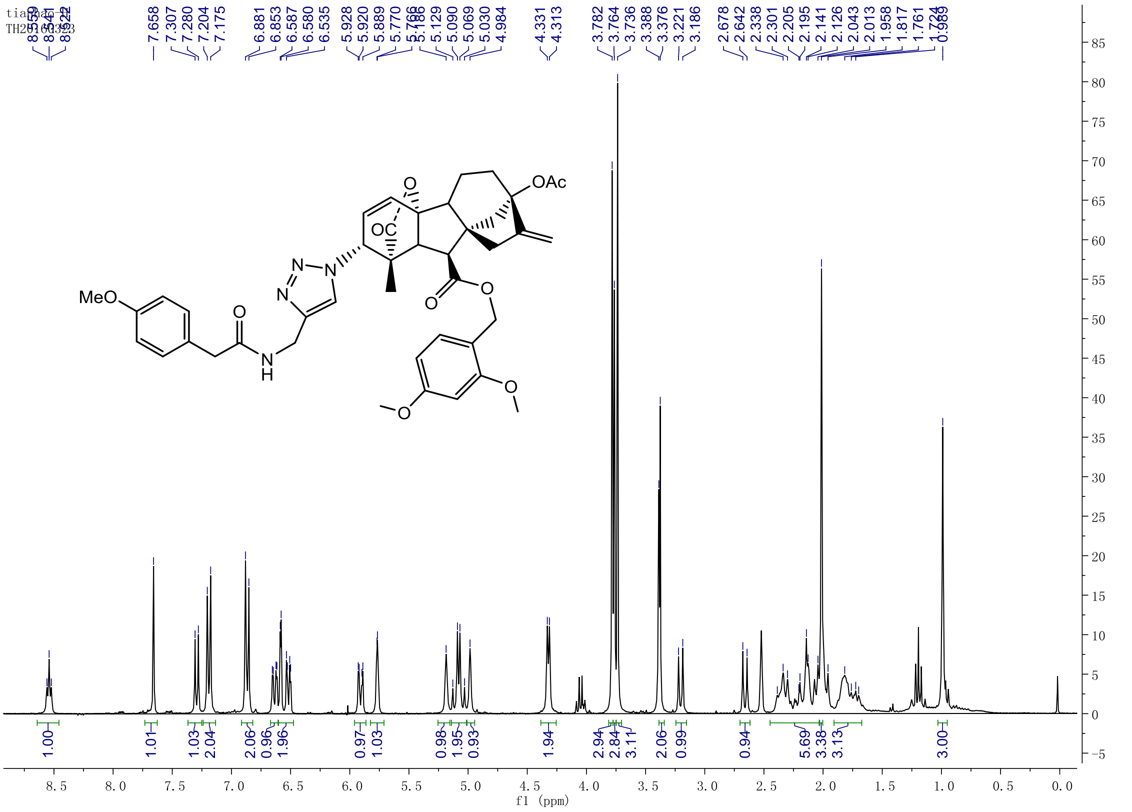


$^{13}$C-NMR spectrum of compound **9d.**

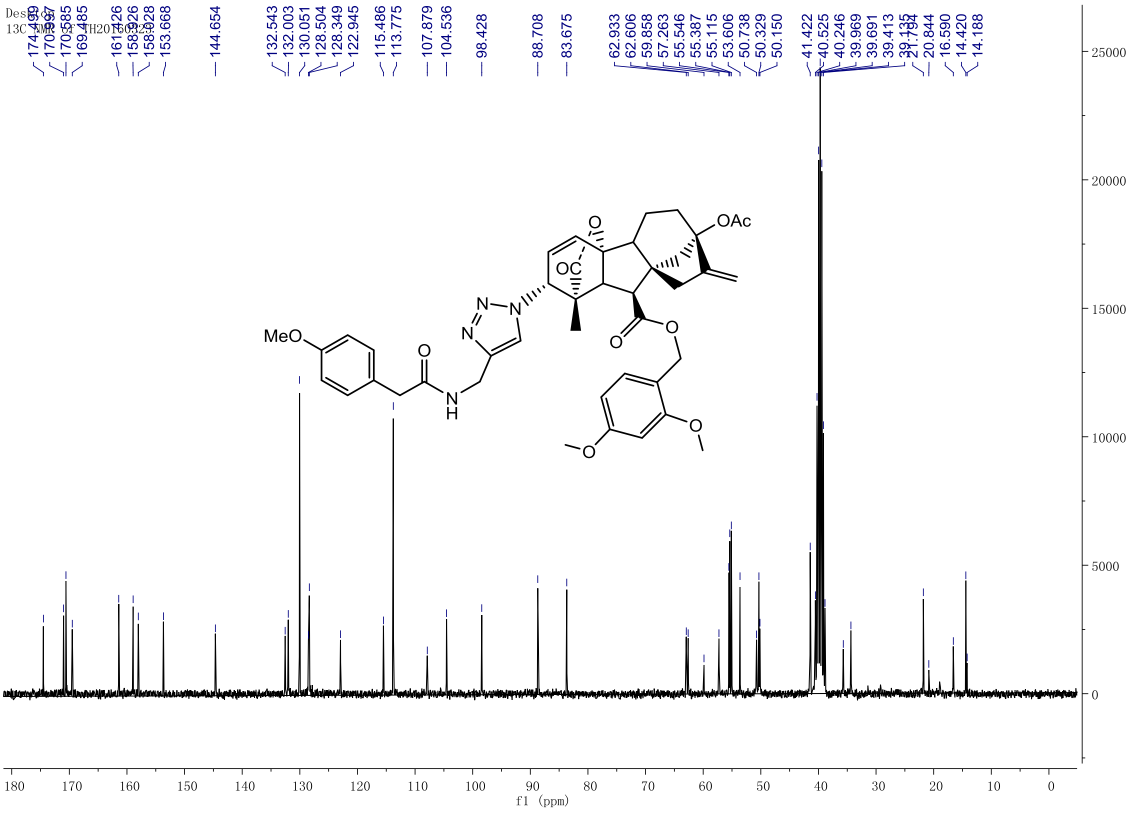

HRMS of compound **9d.**

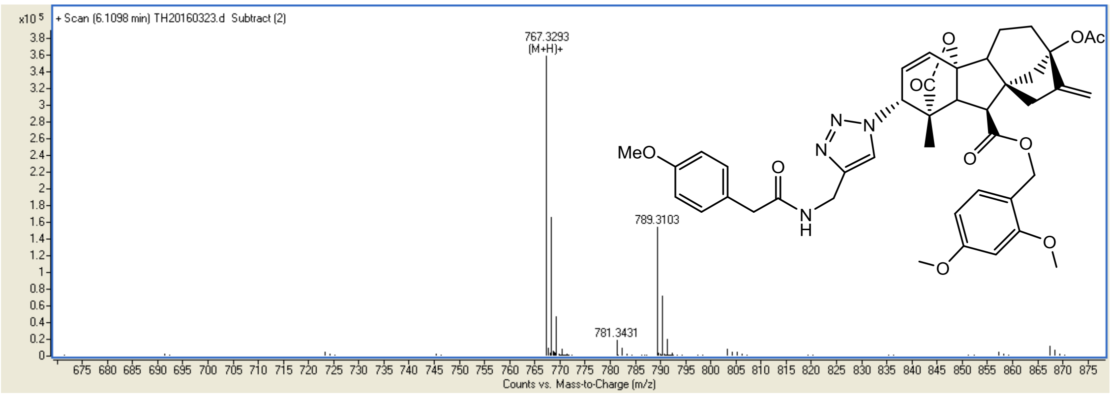


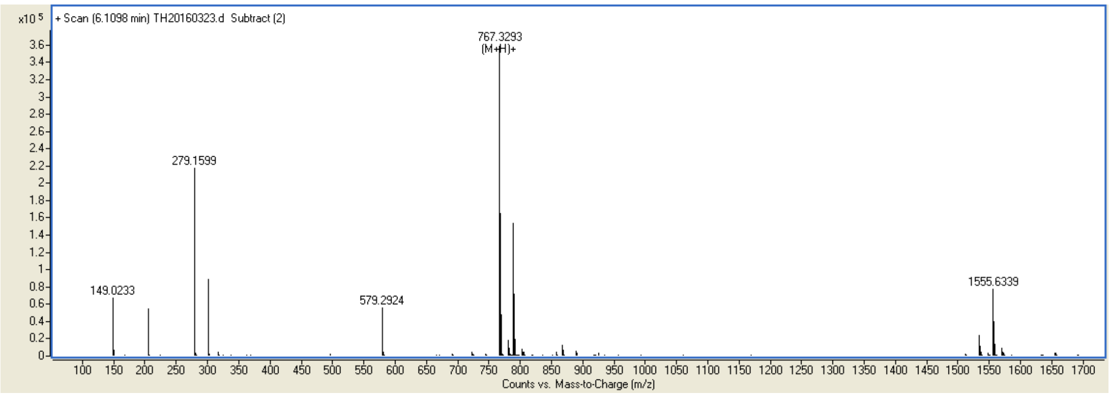


$^{1}$H-NMR spectrum of compound **9e.**

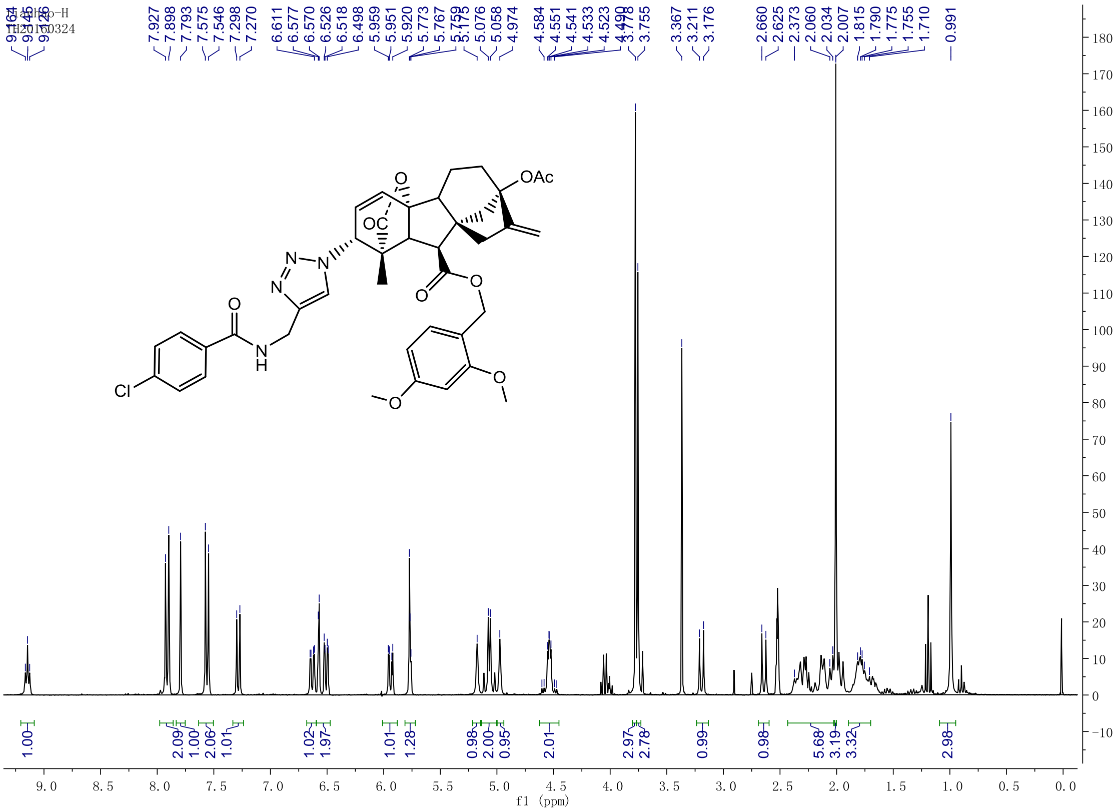

$^{13}$C-NMR spectrum of compound **9e.**

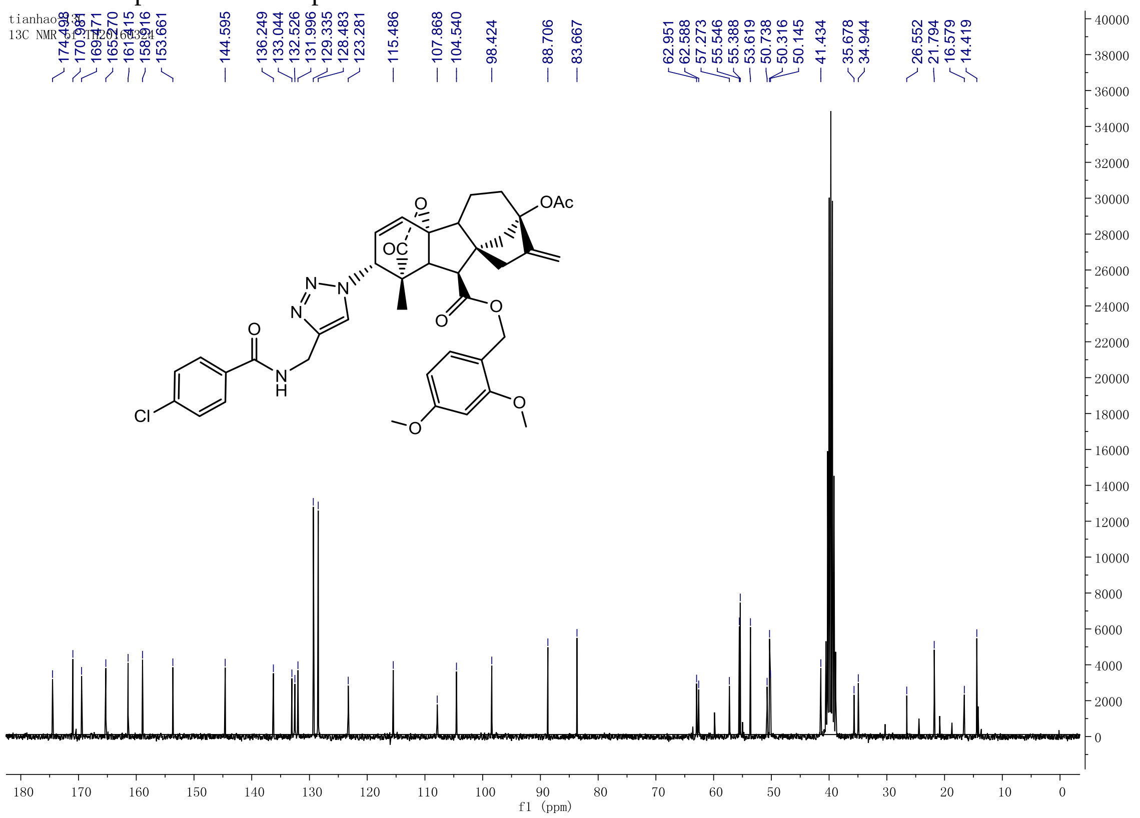


HRMS of compound **9e.**

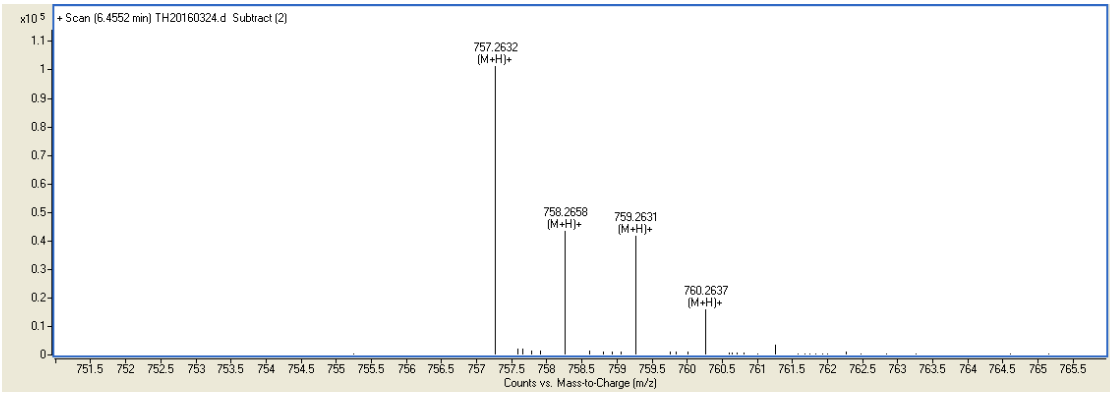


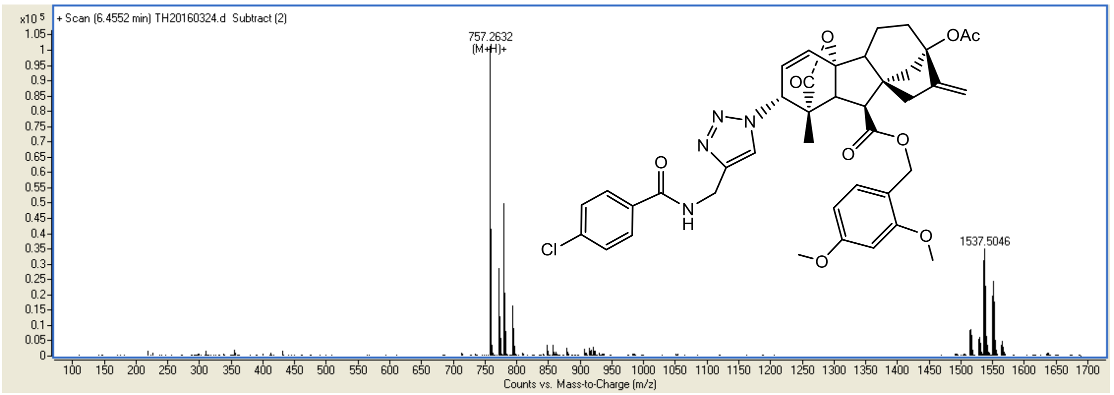

599

600 $^{1}$H-NMR spectrum of compound **9f.**

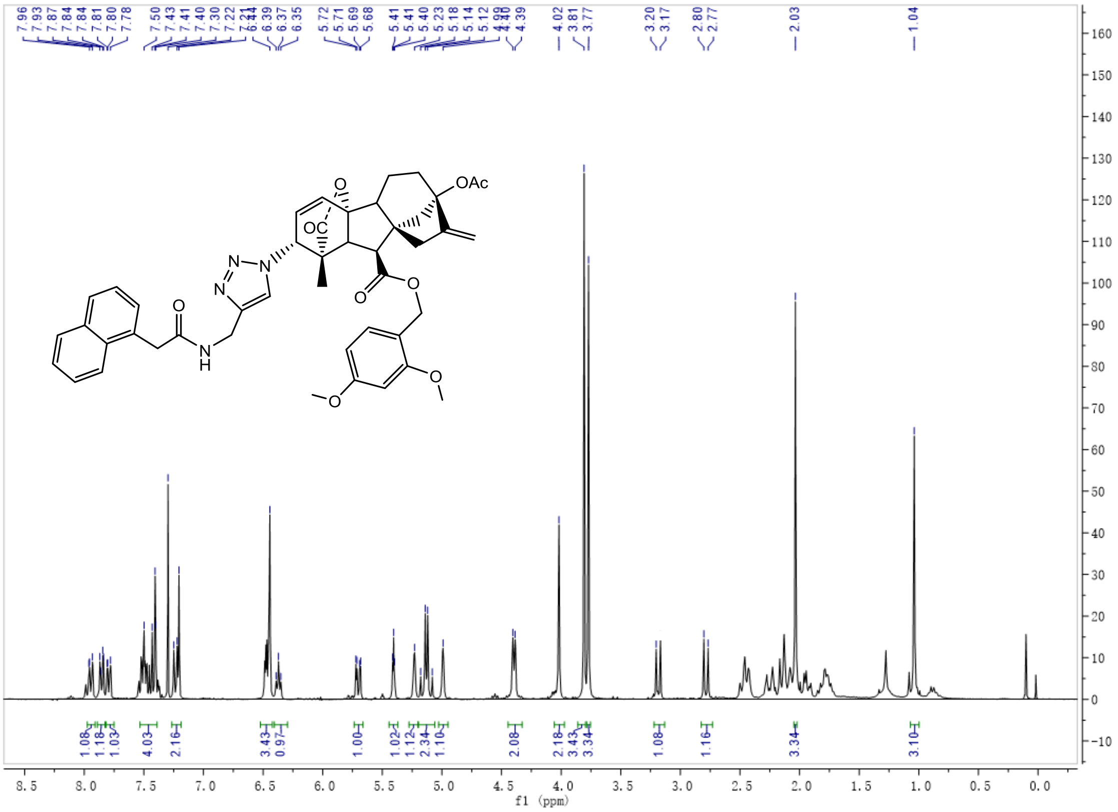

601

602 $^{13}$C-NMR spectrum of compound **9f.**

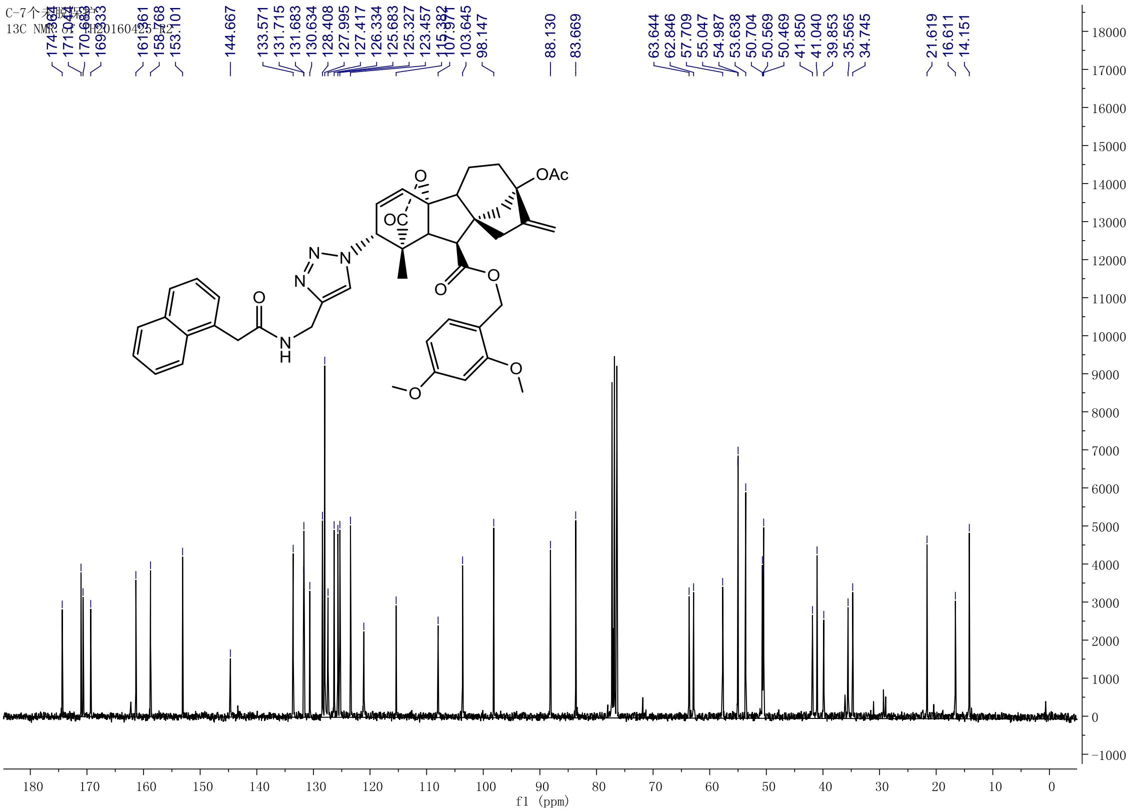

603

HRMS of compound **9f.**

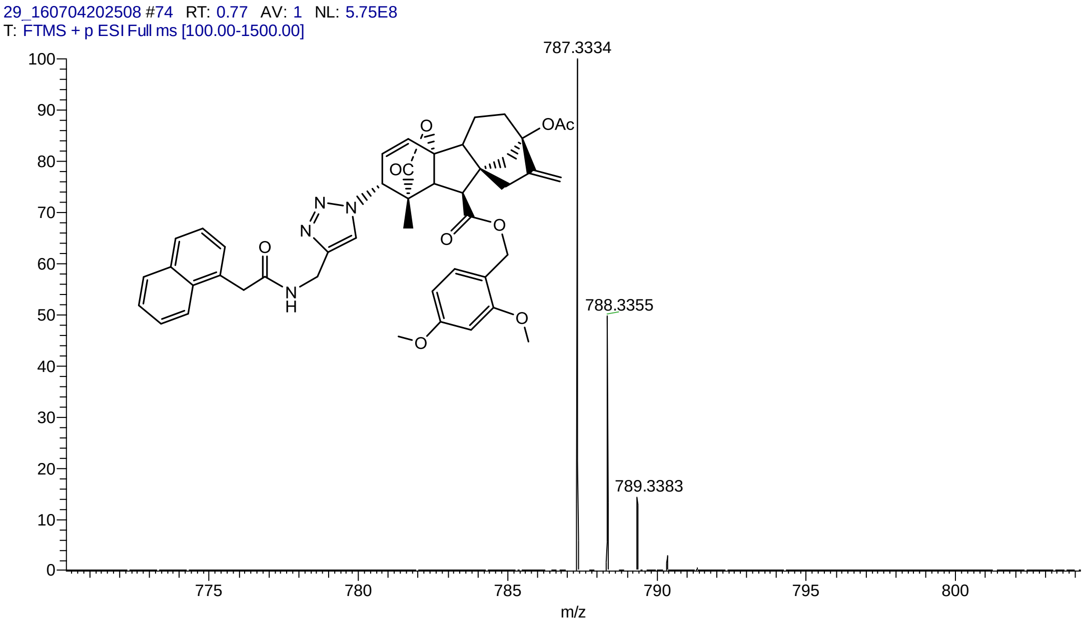


$^{1}$H-NMR spectrum of compound **9g.**

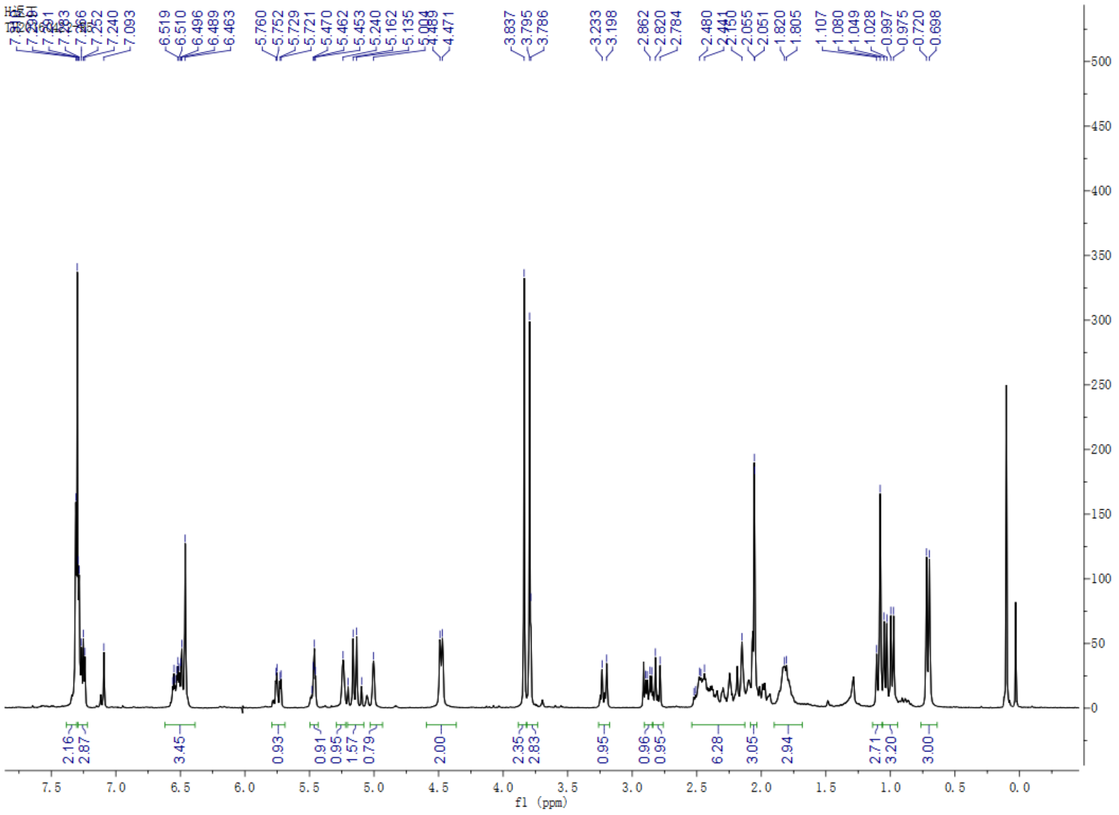

$^{13}$C-NMR spectrum of compound **9g.**

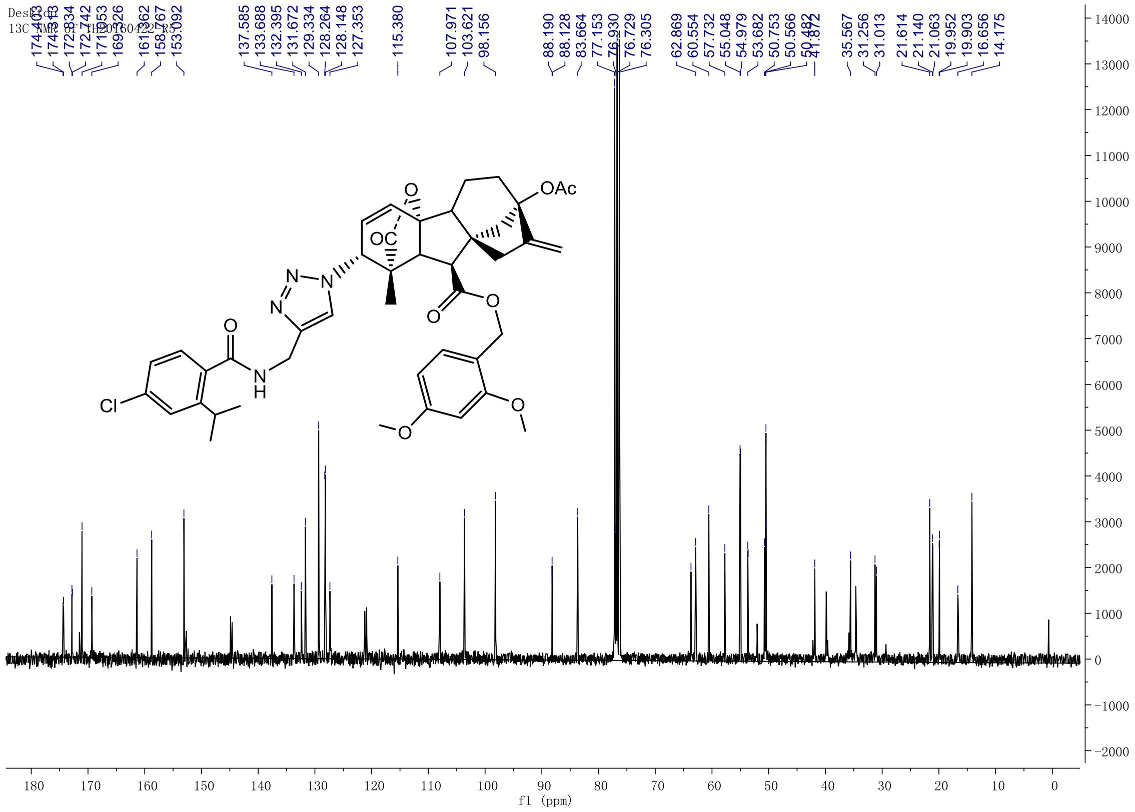


HRMS of compound **9g.**

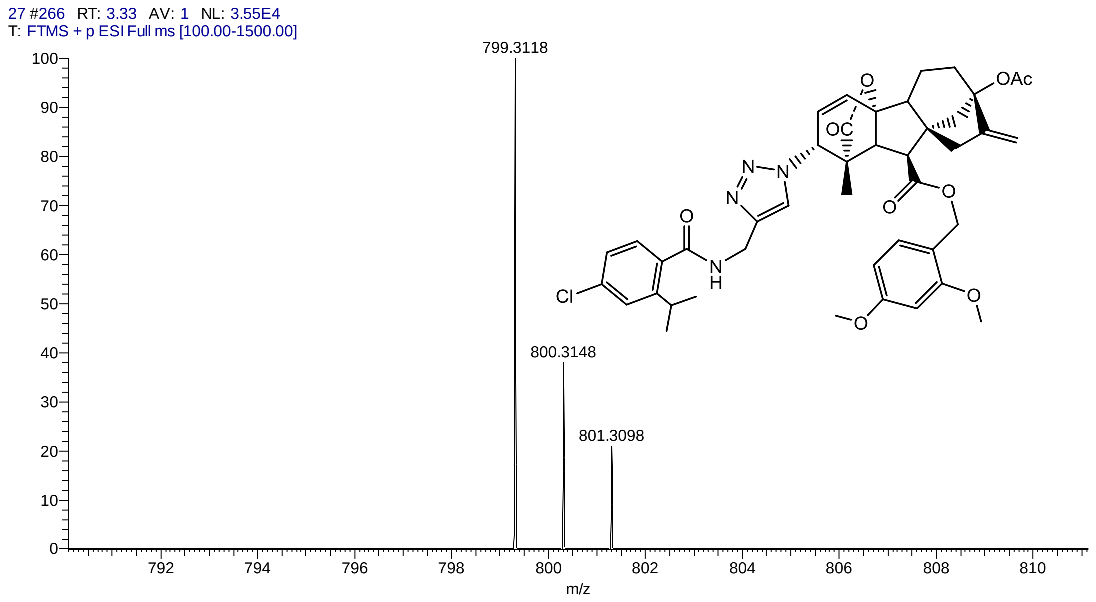

$^{1}$H-NMR spectrum of compound **9h.**

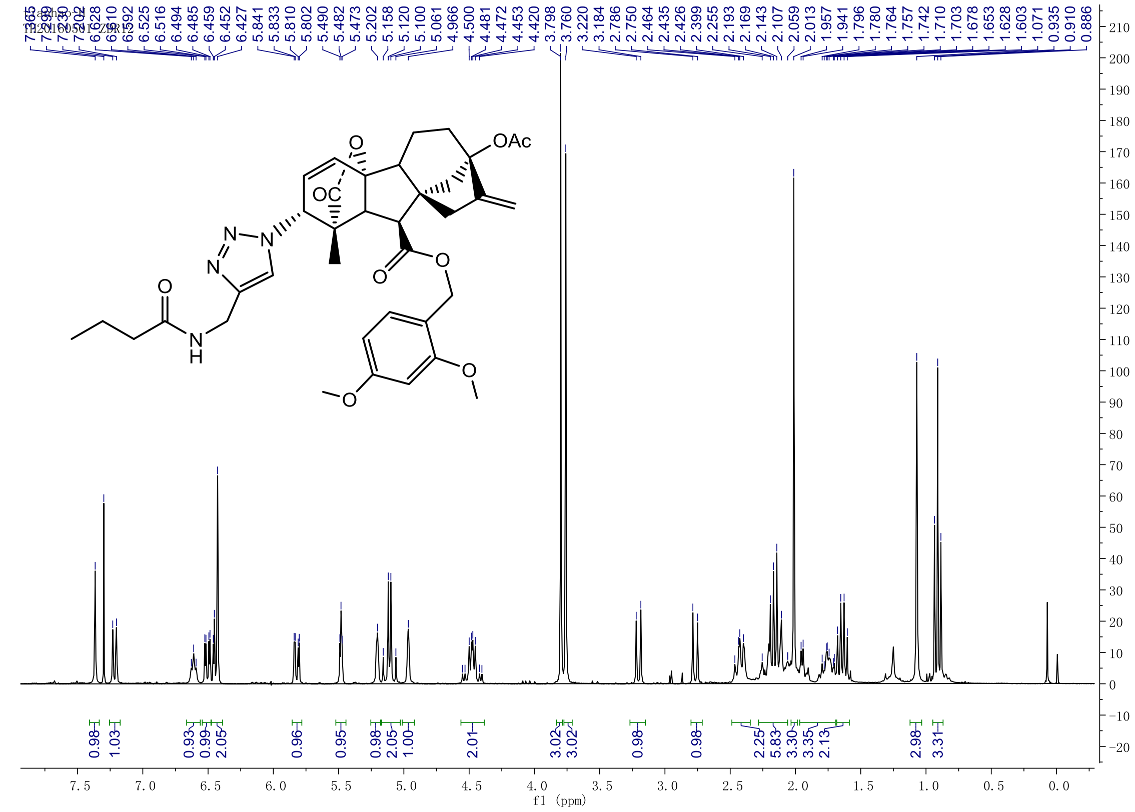


$^{13}$C-NMR spectrum of compound **9h.**

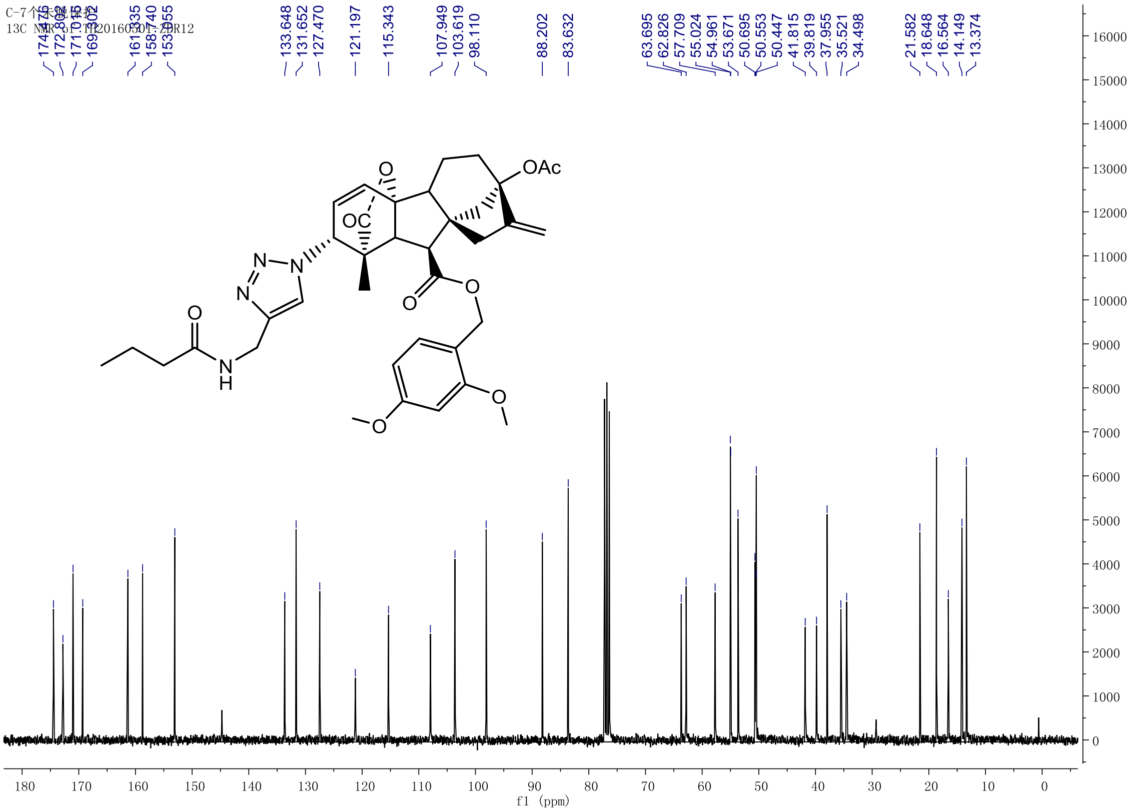

HRMS of compound **9h.**

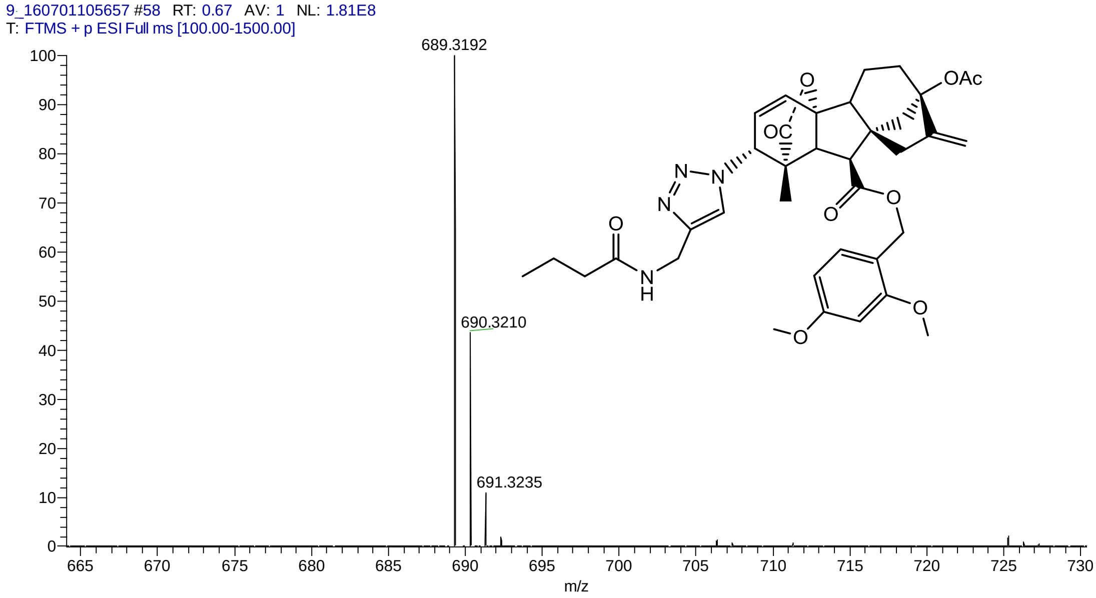


$^{1}$H-NMR spectrum of compound **9i.**

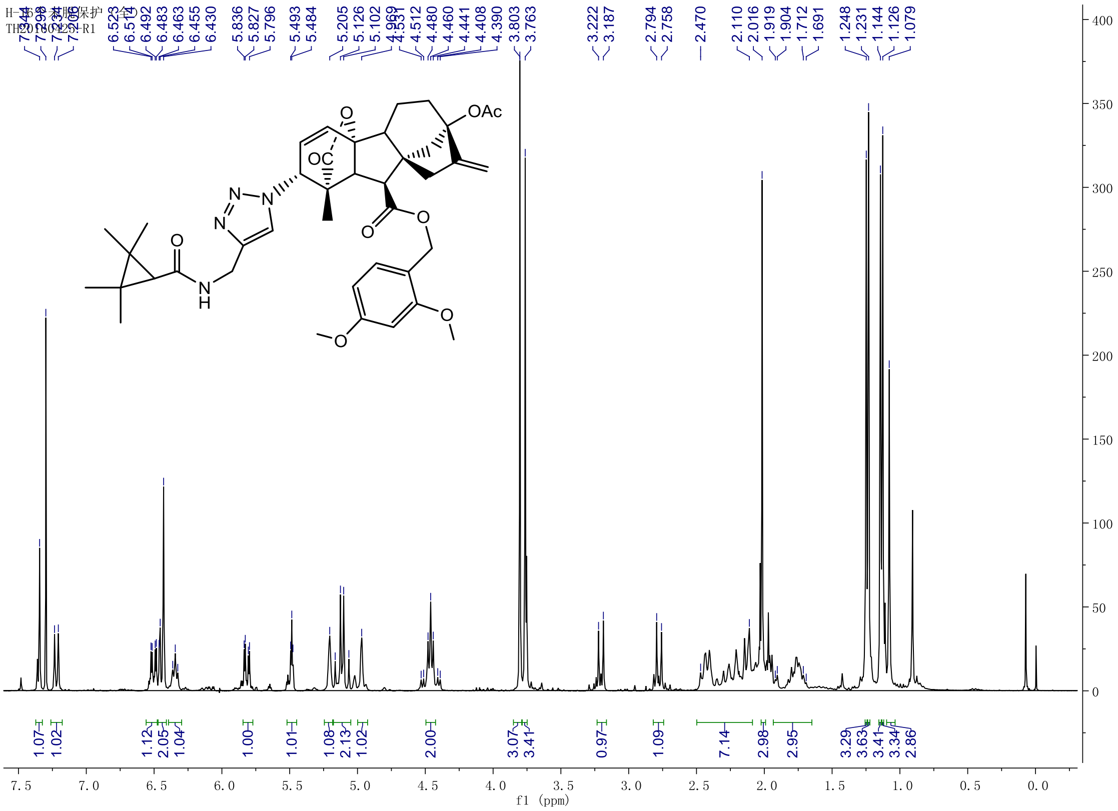

$^{13}$C-NMR spectrum of compound **9i.**

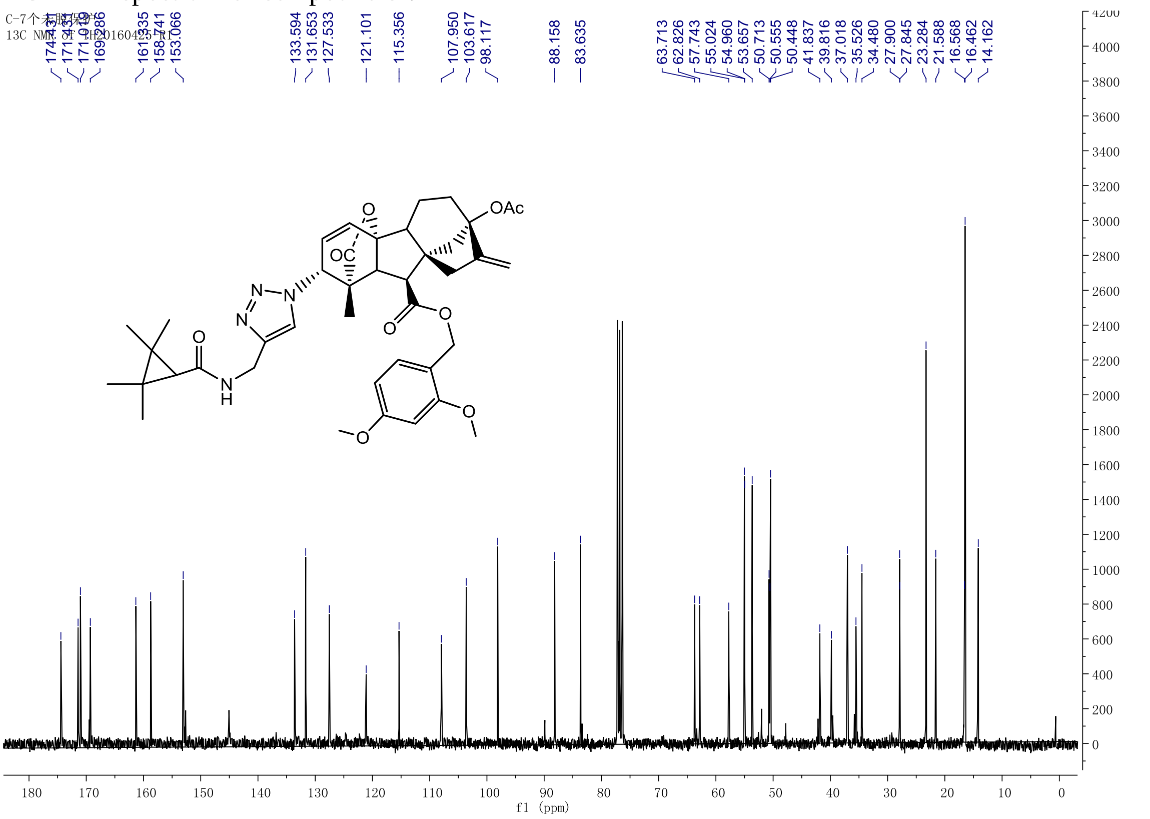


HRMS of compound **9i.**

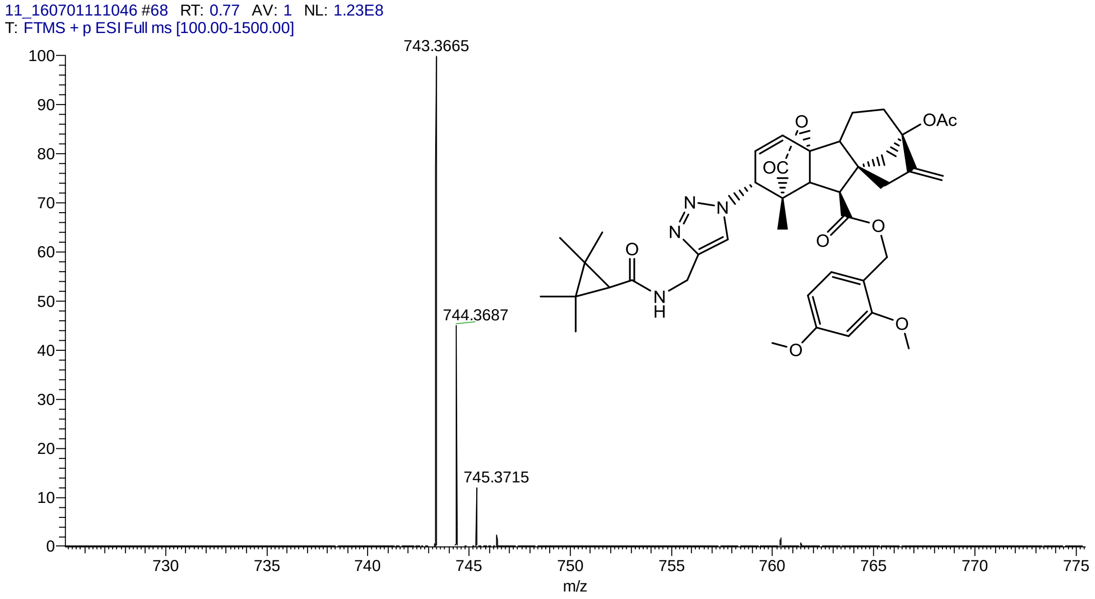

$^{1}$H-NMR spectrum of compound **9j.**

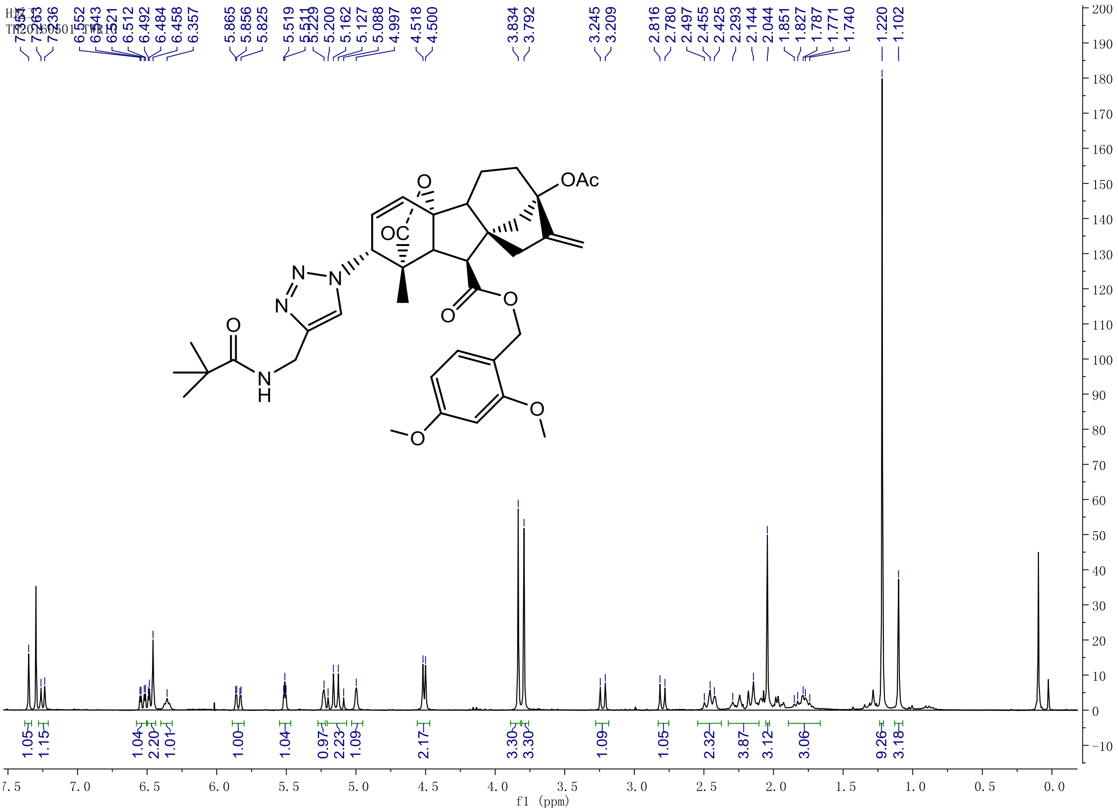


$^{13}$C-NMR spectrum of compound **9j.**

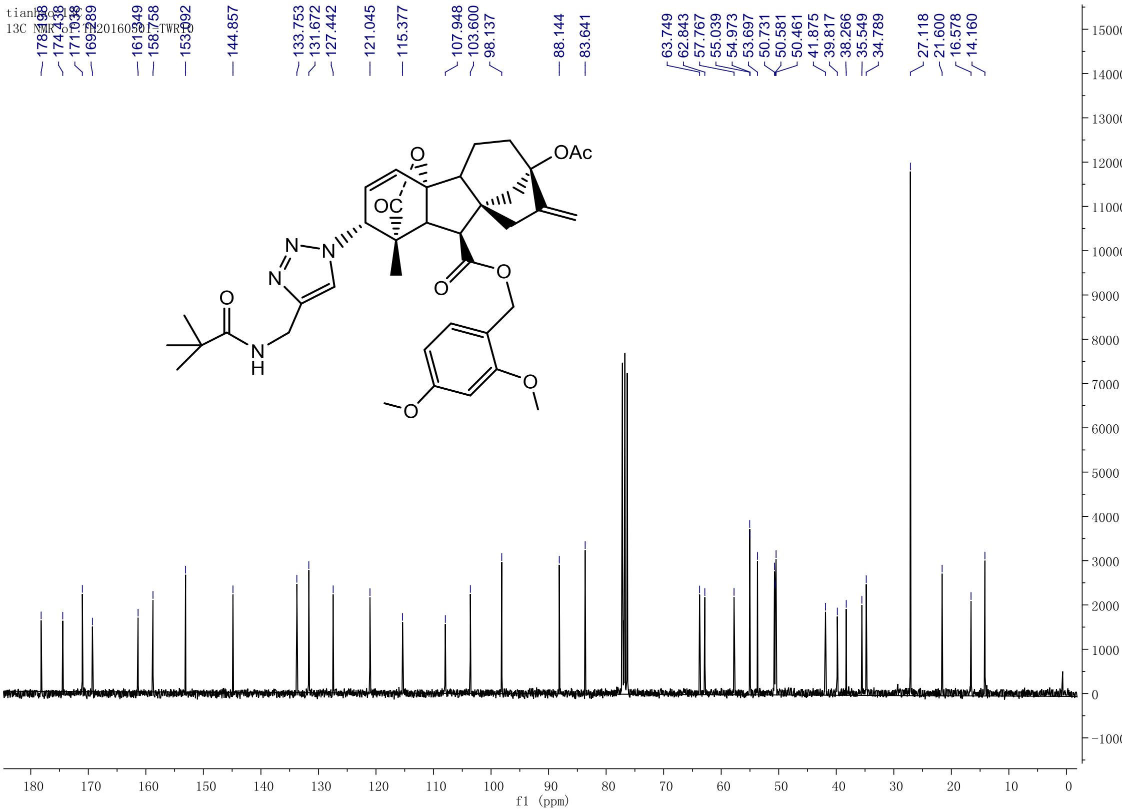

HRMS of compound **9j.**

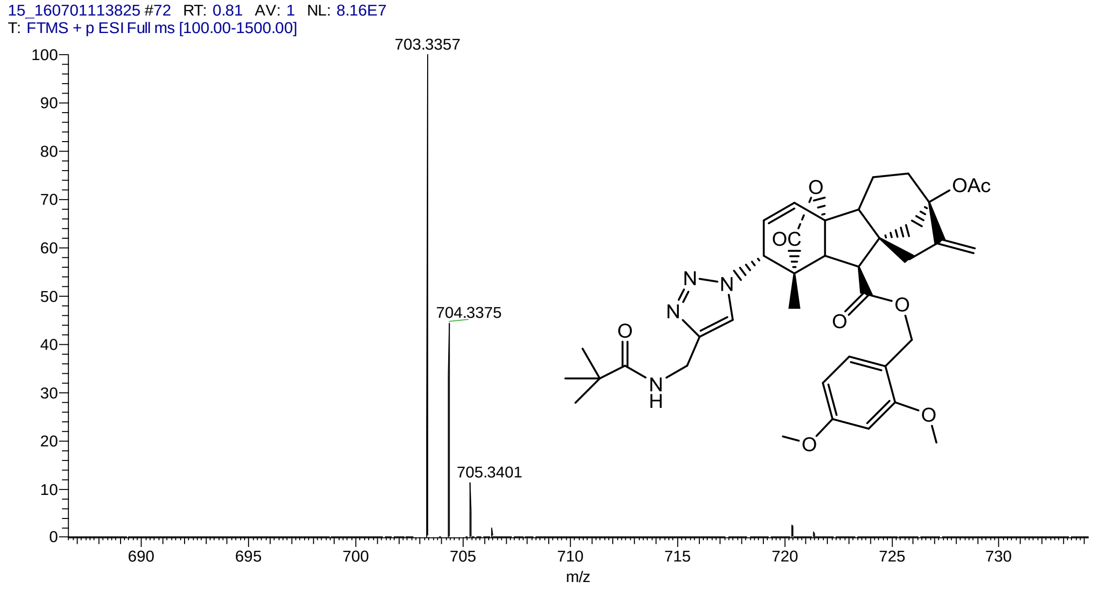


$^{1}$H-NMR spectrum of compound **9k.**

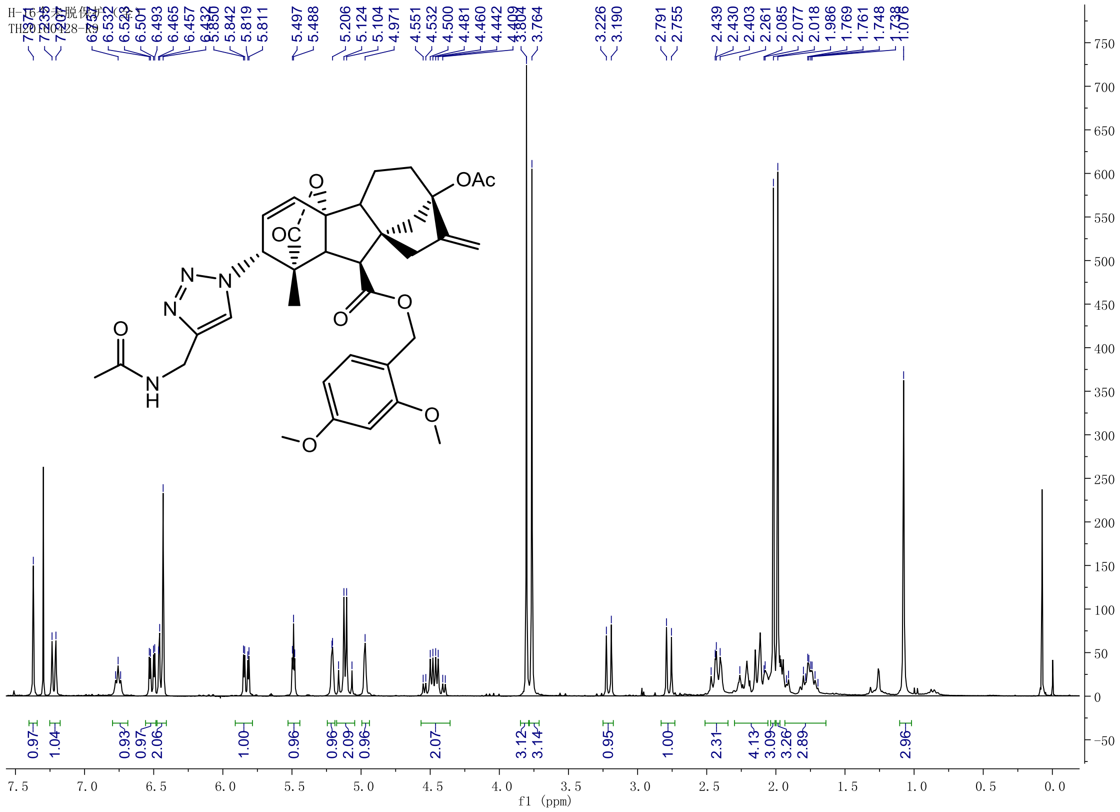

$^{13}$C-NMR spectrum of compound **9k.**

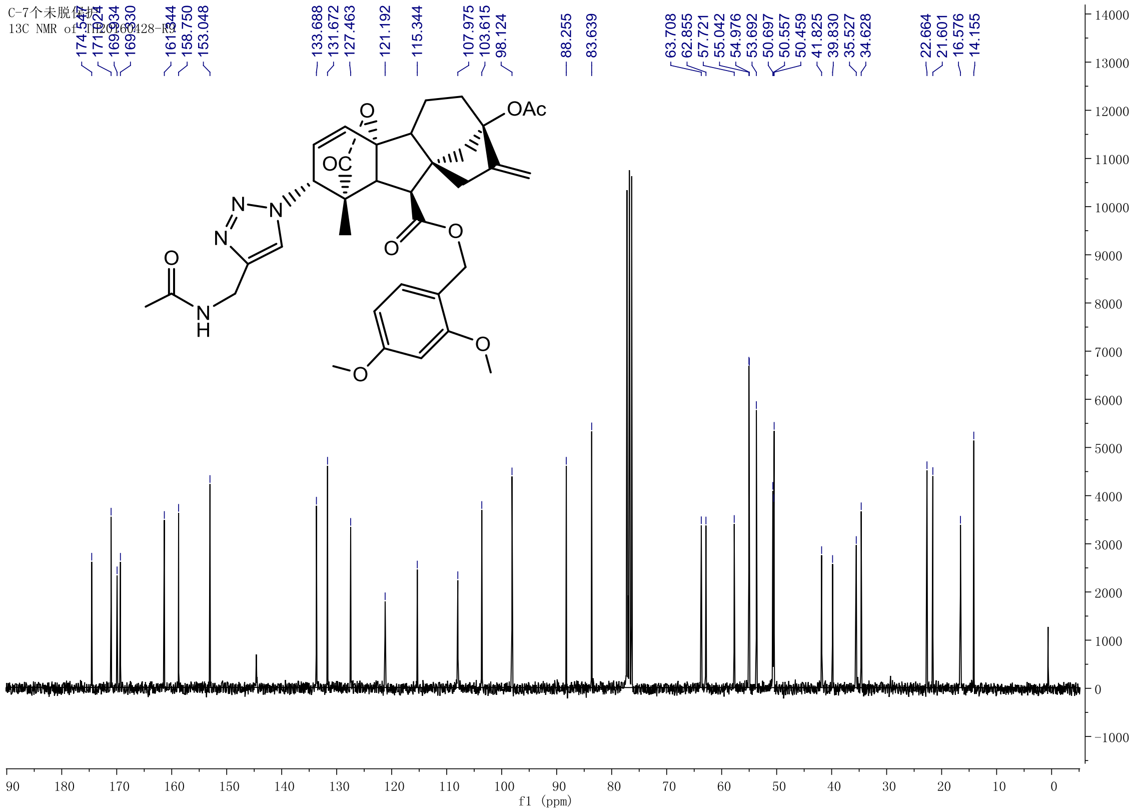


HRMS of compound **9k.**

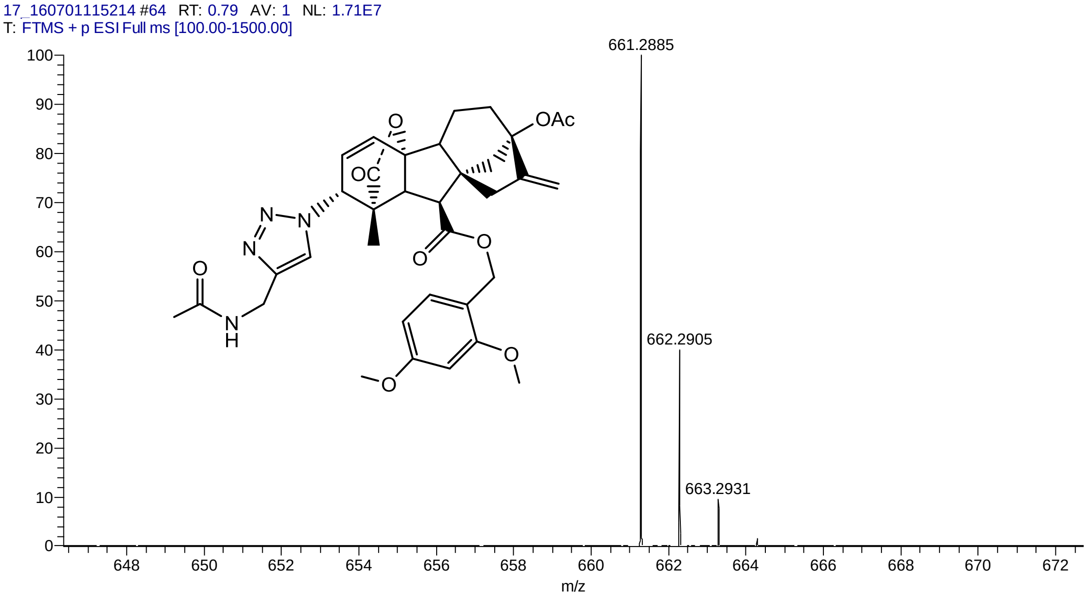

$^{1}$H-NMR spectrum of compound **9l.**

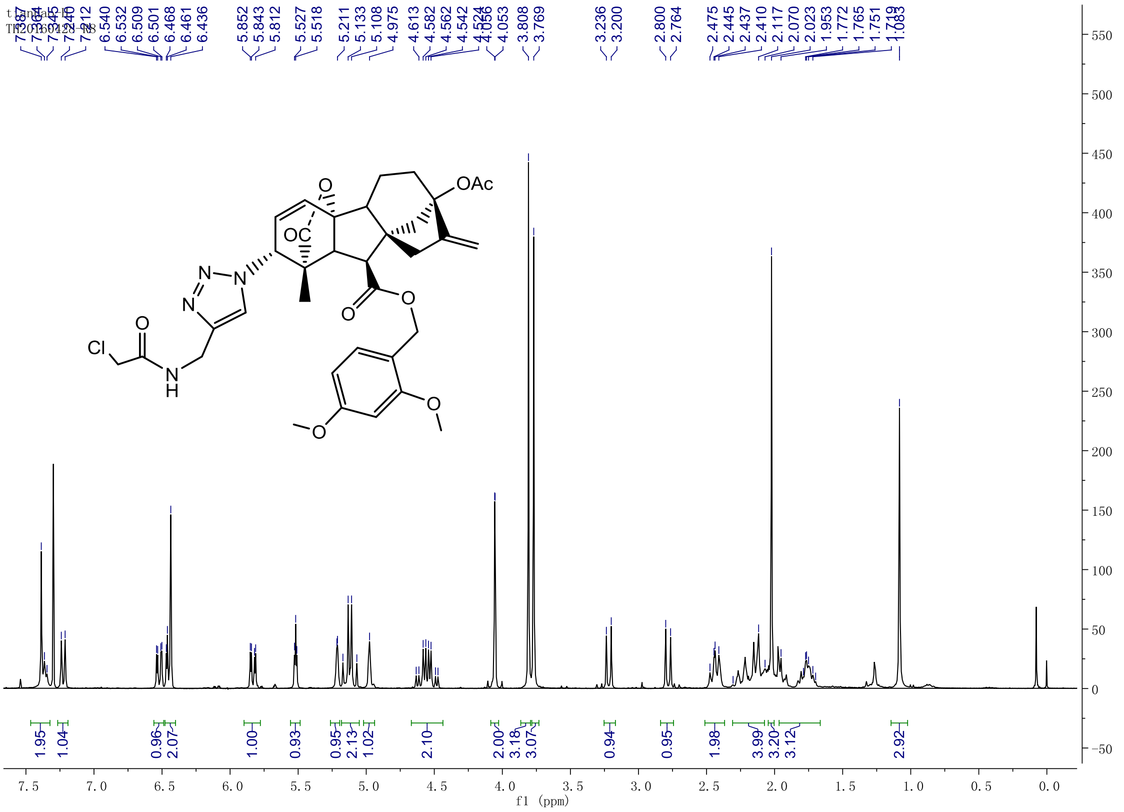


$^{13}$C-NMR spectrum of compound **9l.**

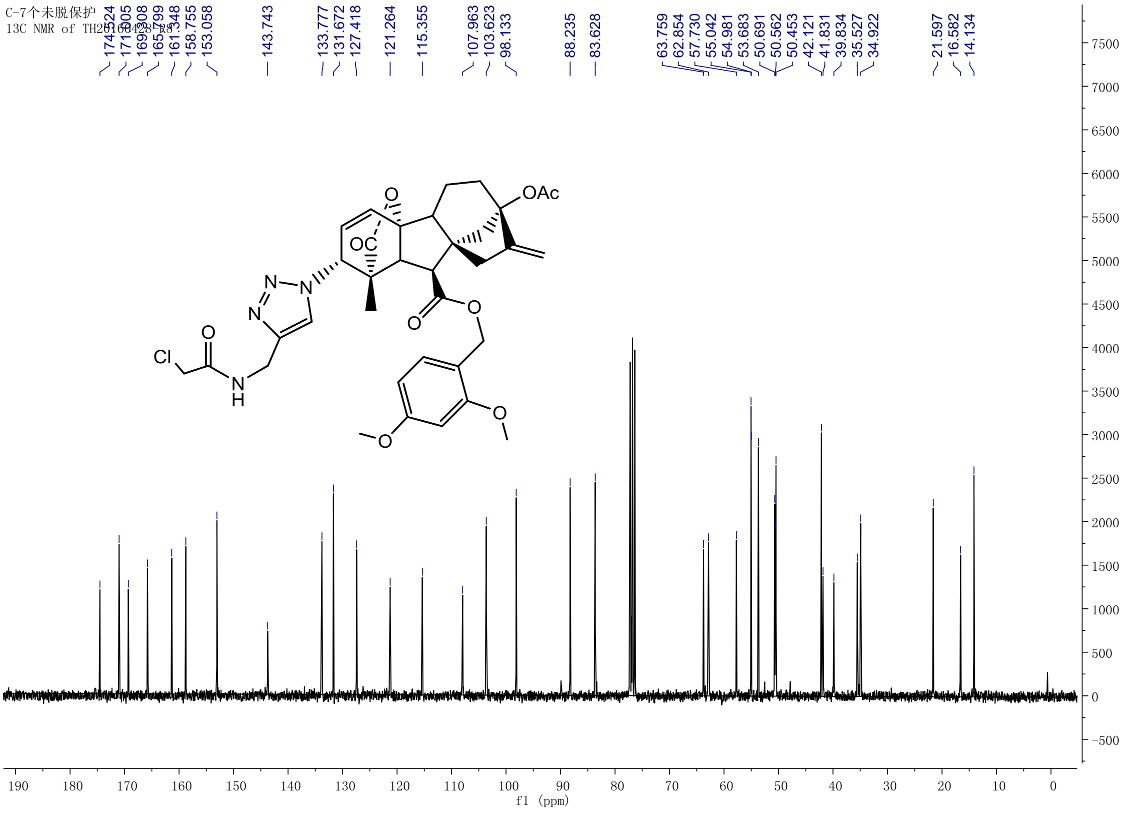

HRMS of compound **9l.**

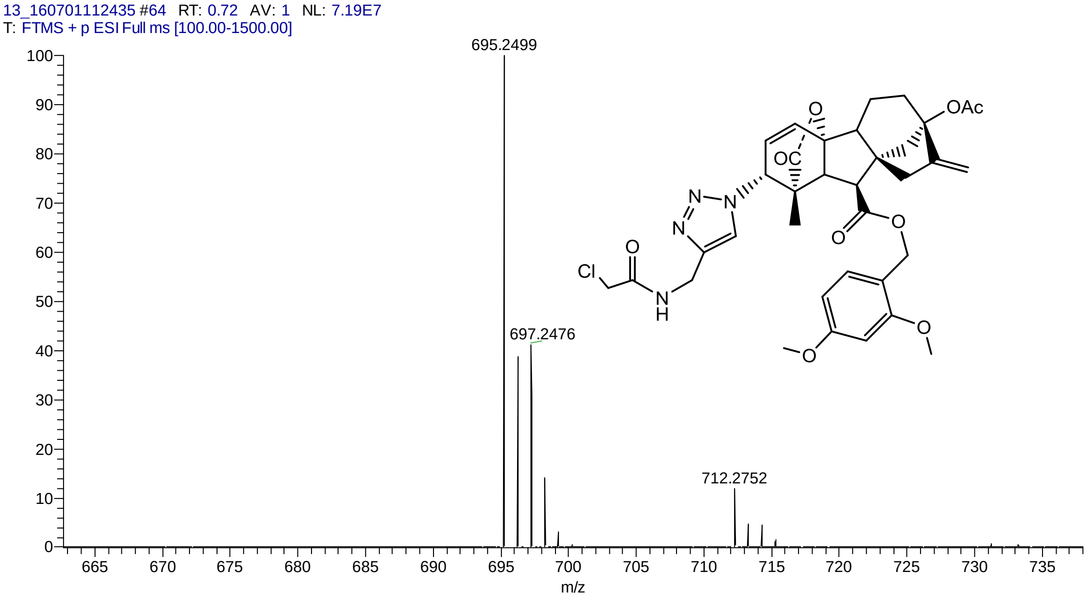


$^{1}$H-NMR spectrum of compound **9m.**

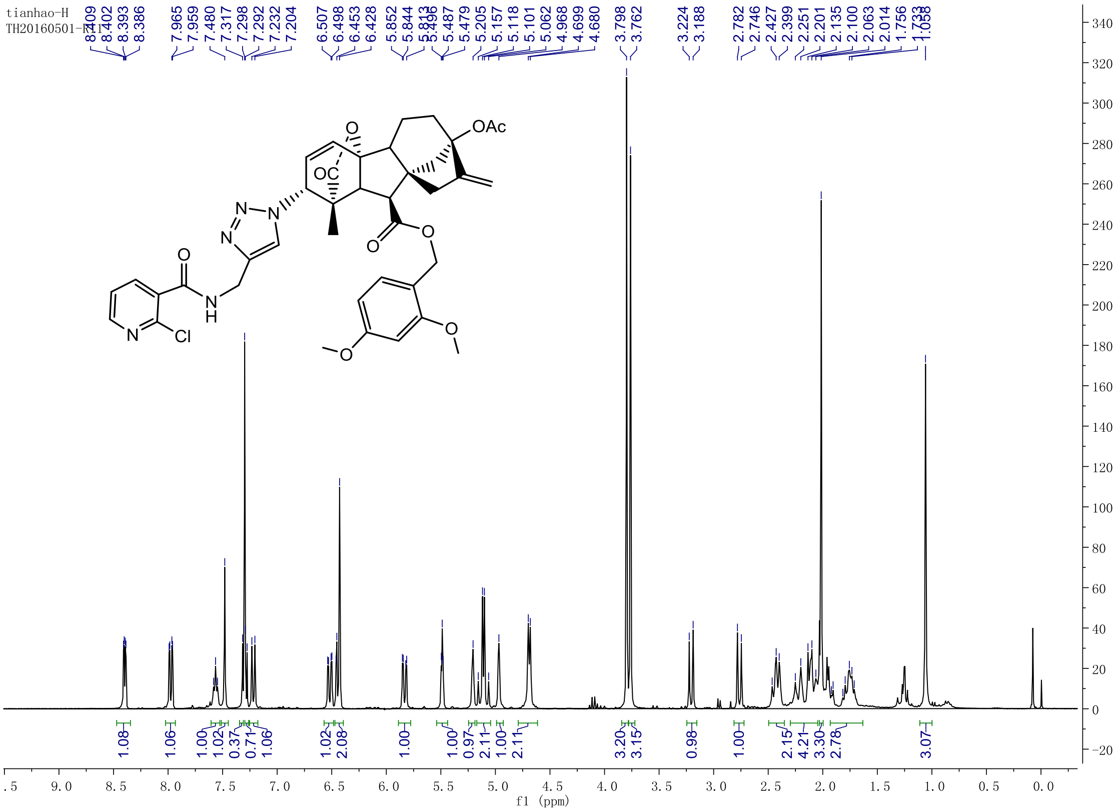

$^{13}$C-NMR spectrum of compound **9m.**

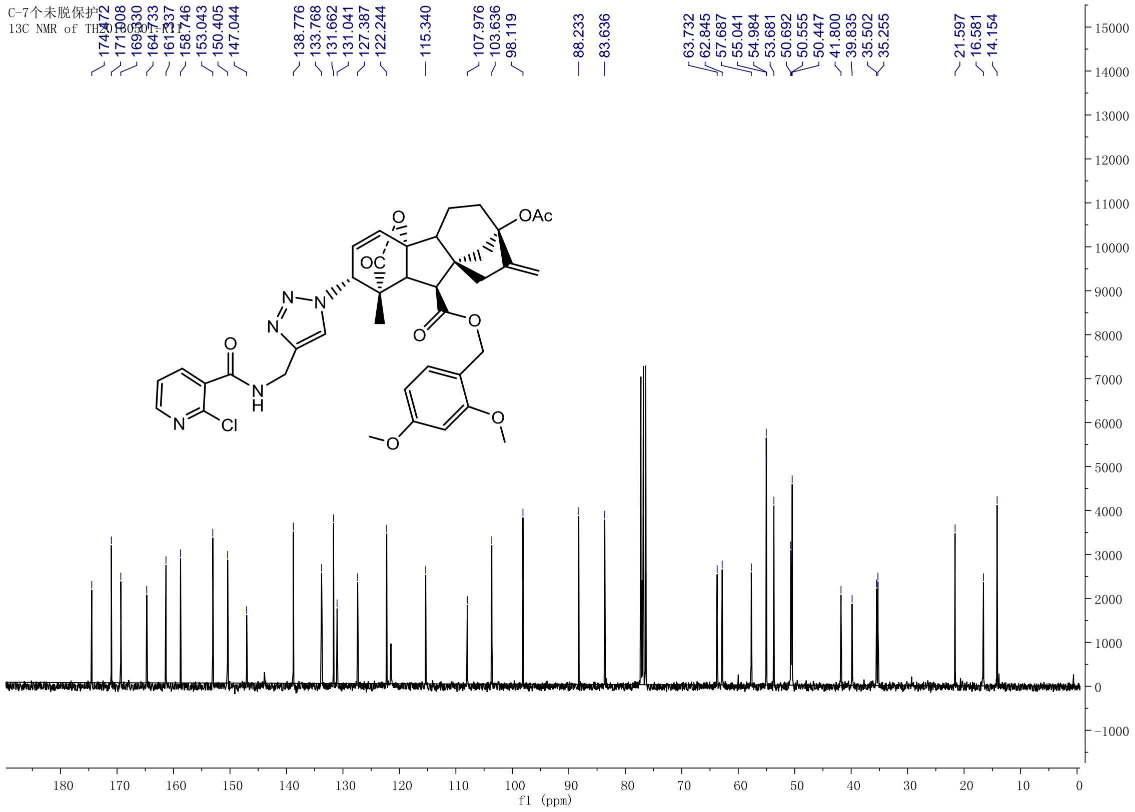


HRMS of compound **9m.**

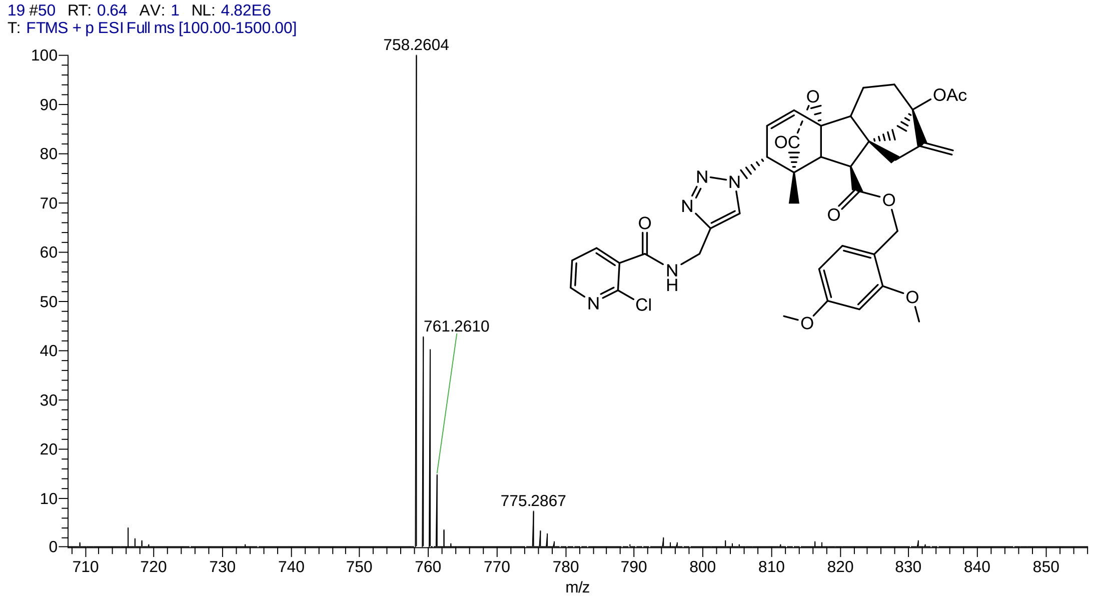

$^{1}$H-NMR spectrum of compound **9n.**

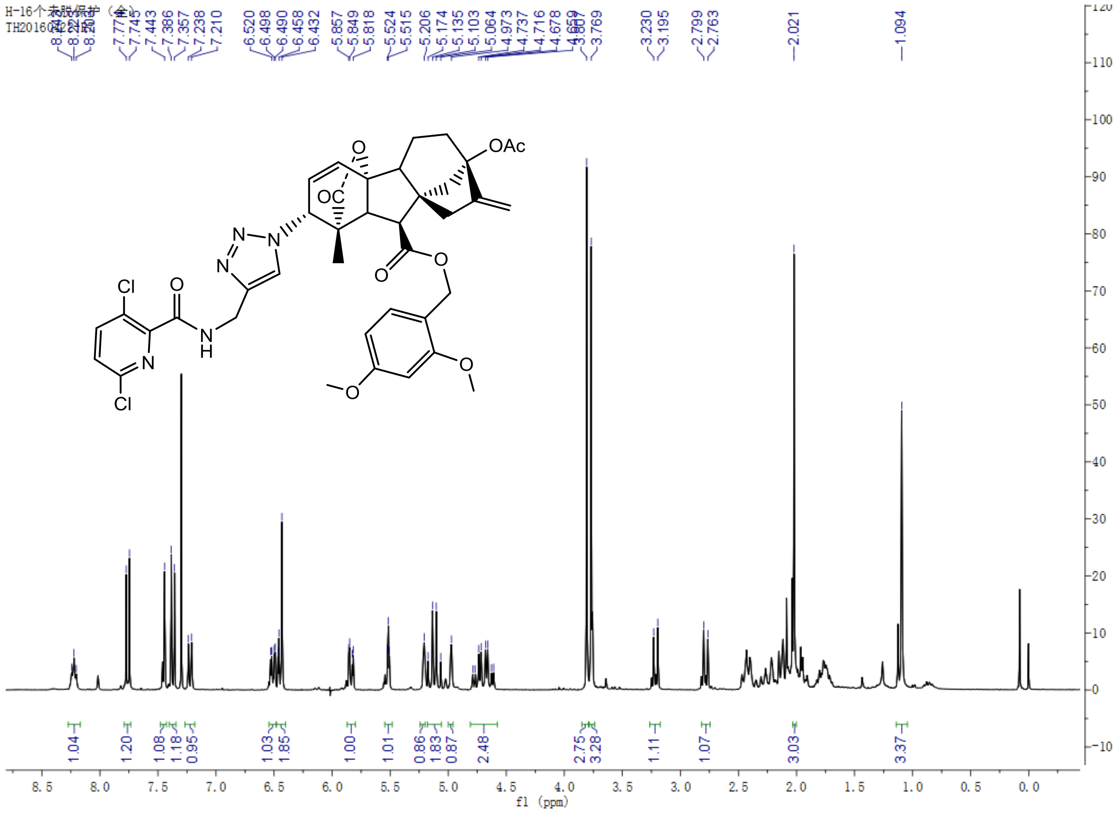


$^{13}$C-NMR spectrum of compound **9n.**

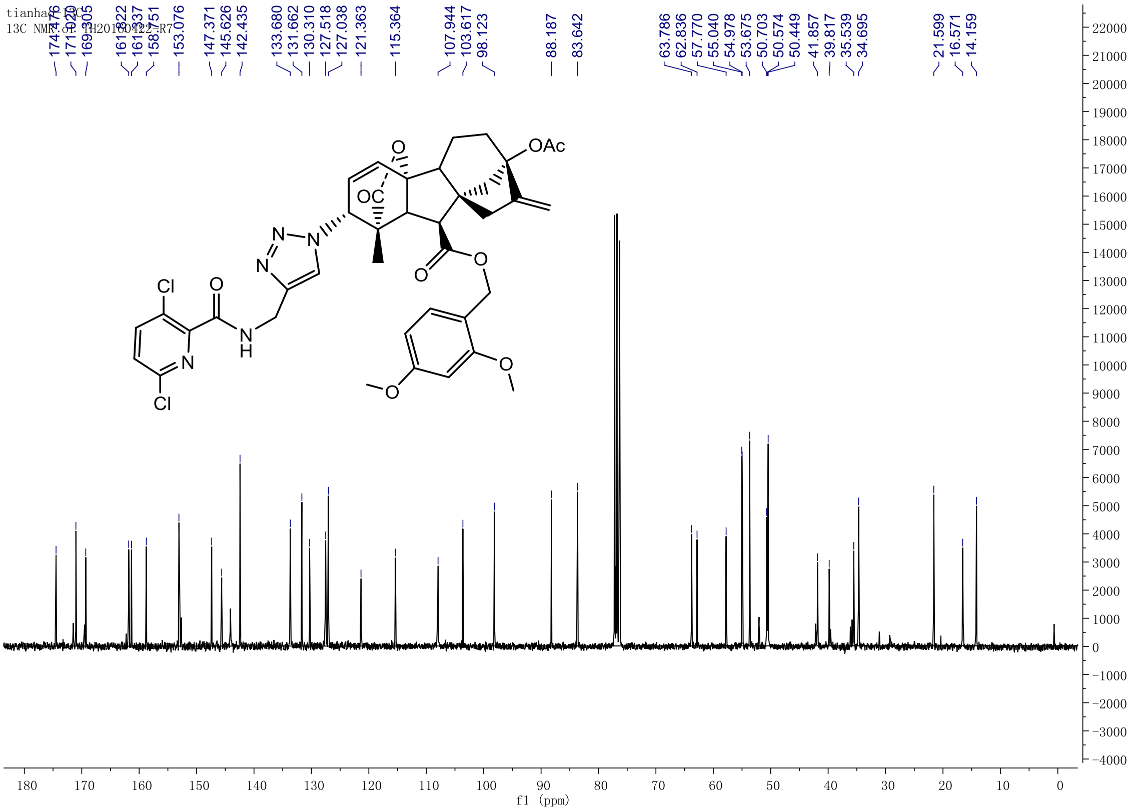

HRMS of compound **9n.**

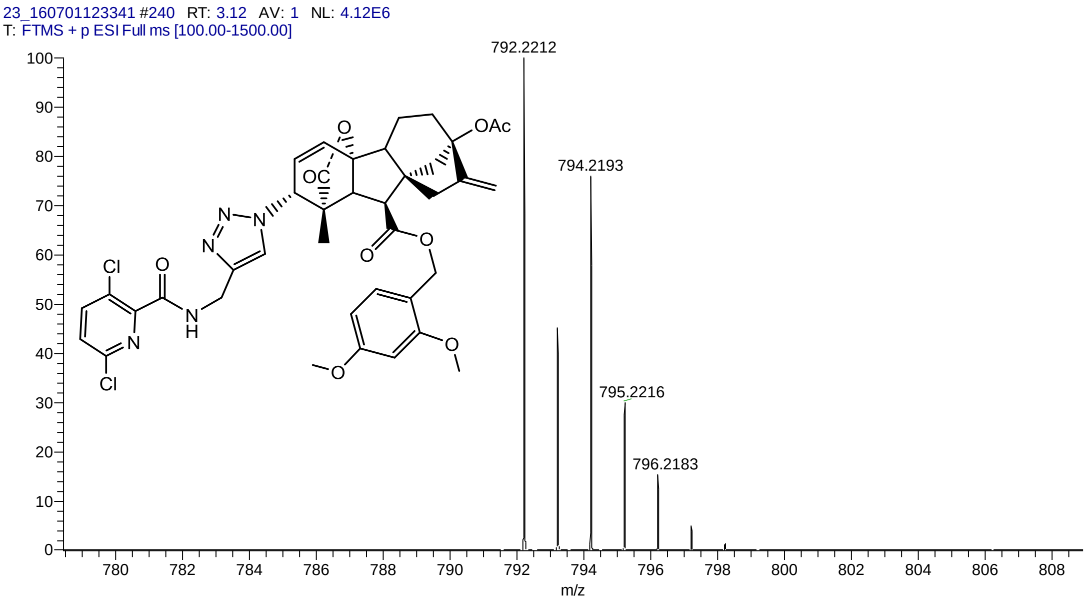


$^{1}$H-NMR spectrum of compound **9o.**

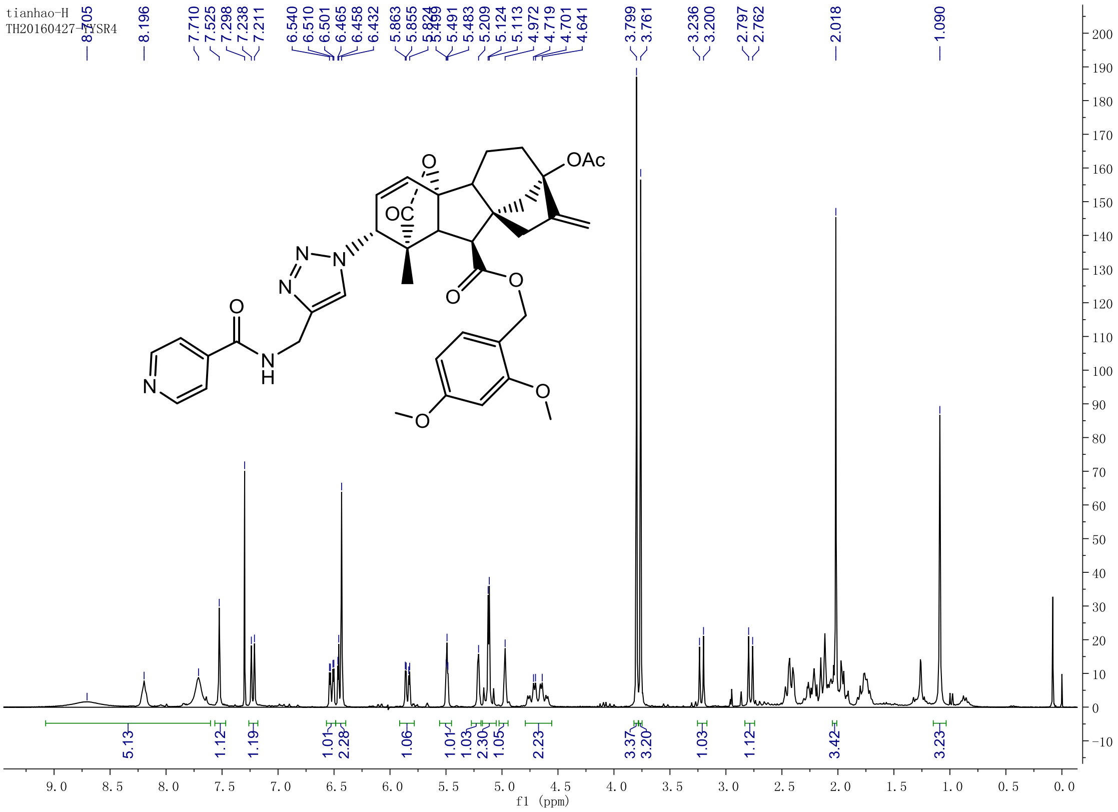

$^{13}$C-NMR spectrum of compound **9o.**

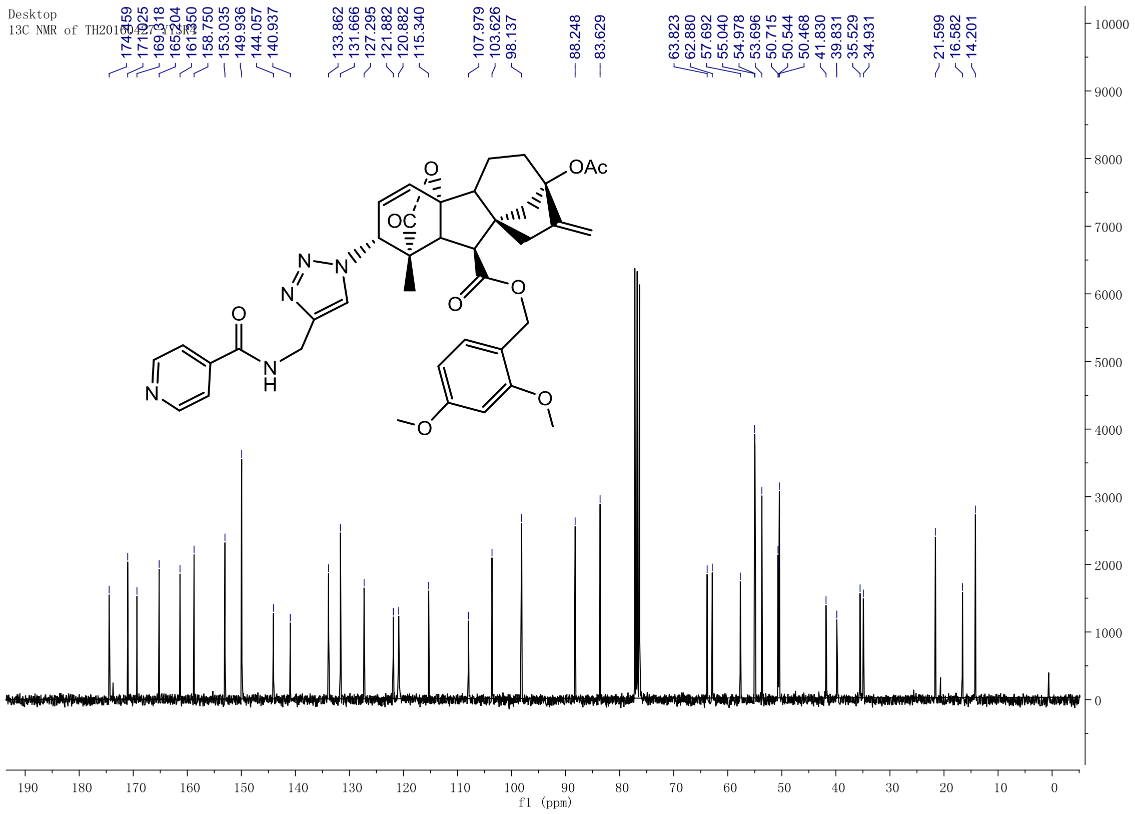


HRMS of compound **9o.**

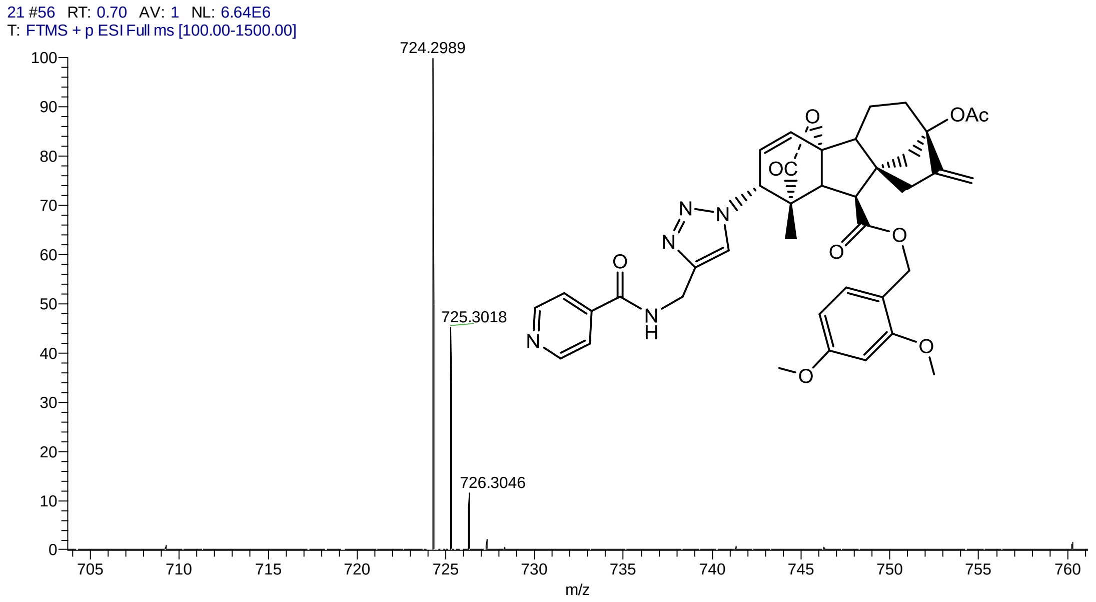

$^{1}$H-NMR spectrum of compound **10a.**

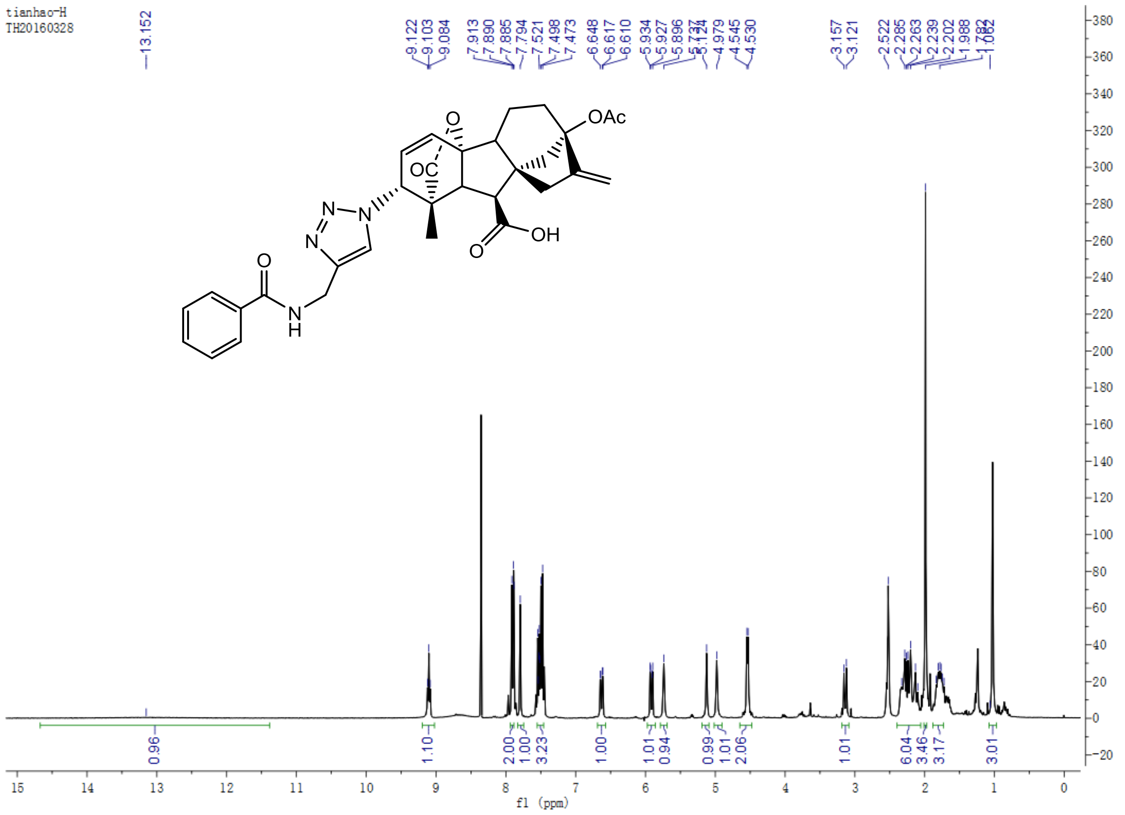


$^{13}$C-NMR spectrum of compound **10a.**

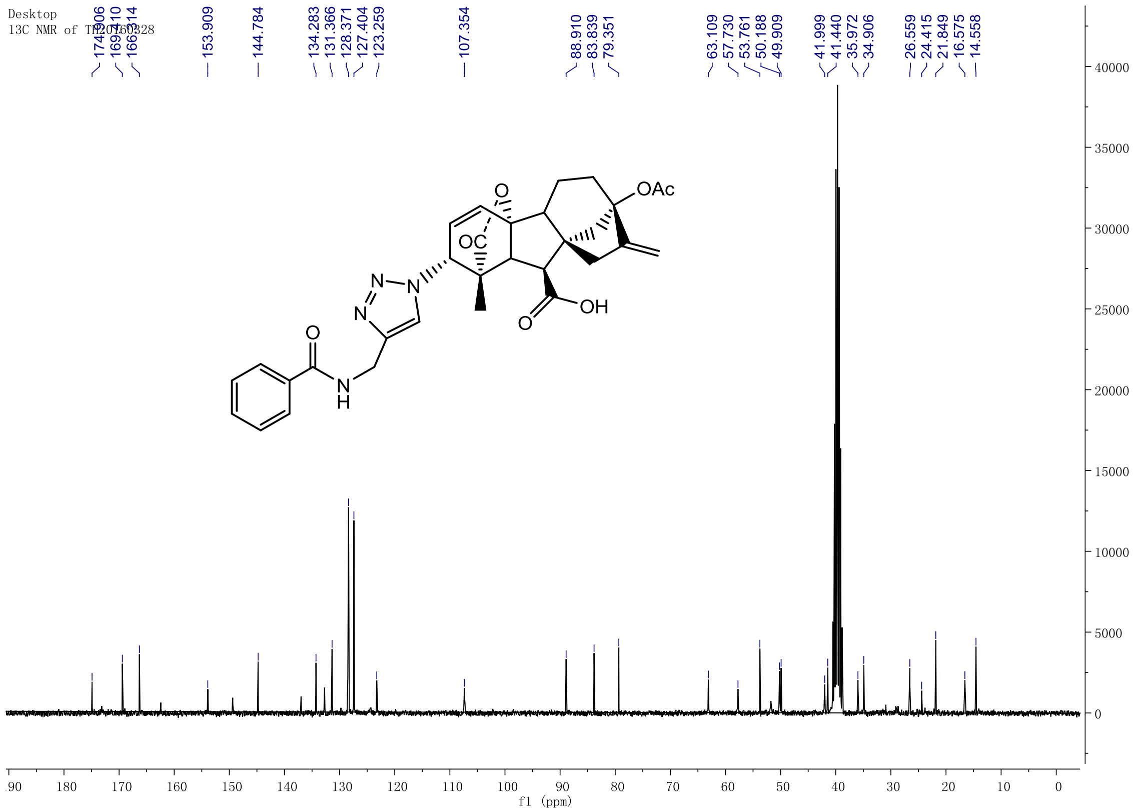

HRMS of compound **10a.**

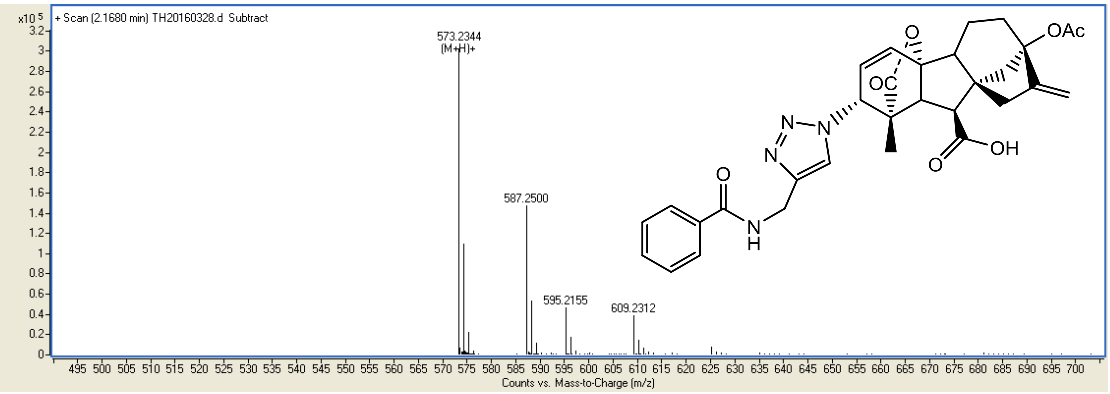


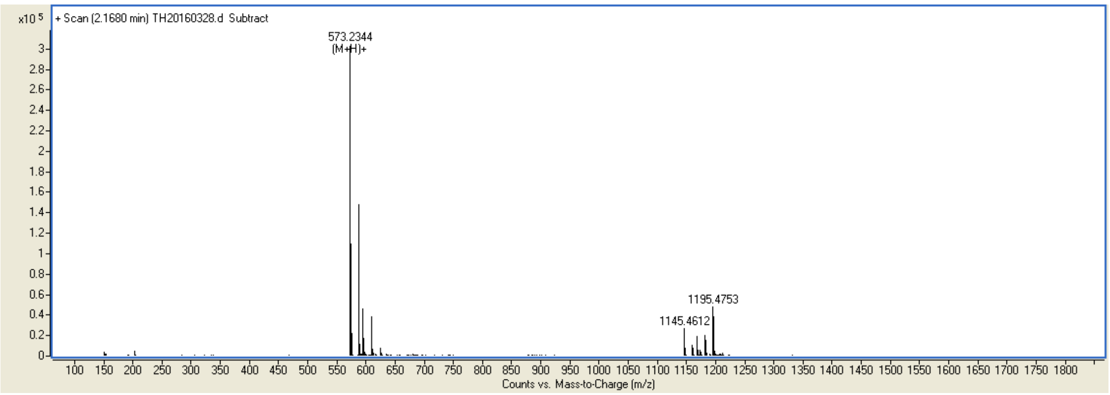


$^{1}$H-NMR spectrum of compound **10b.**

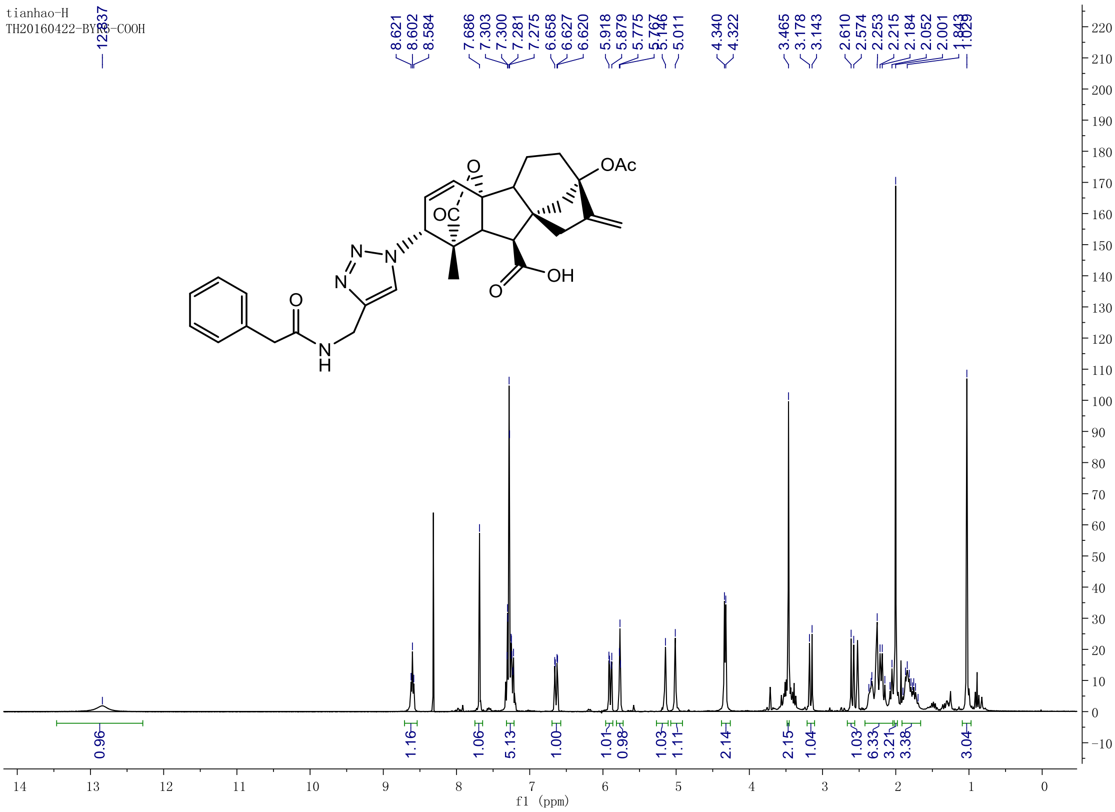

$^{13}$C-NMR spectrum of compound **10b.**

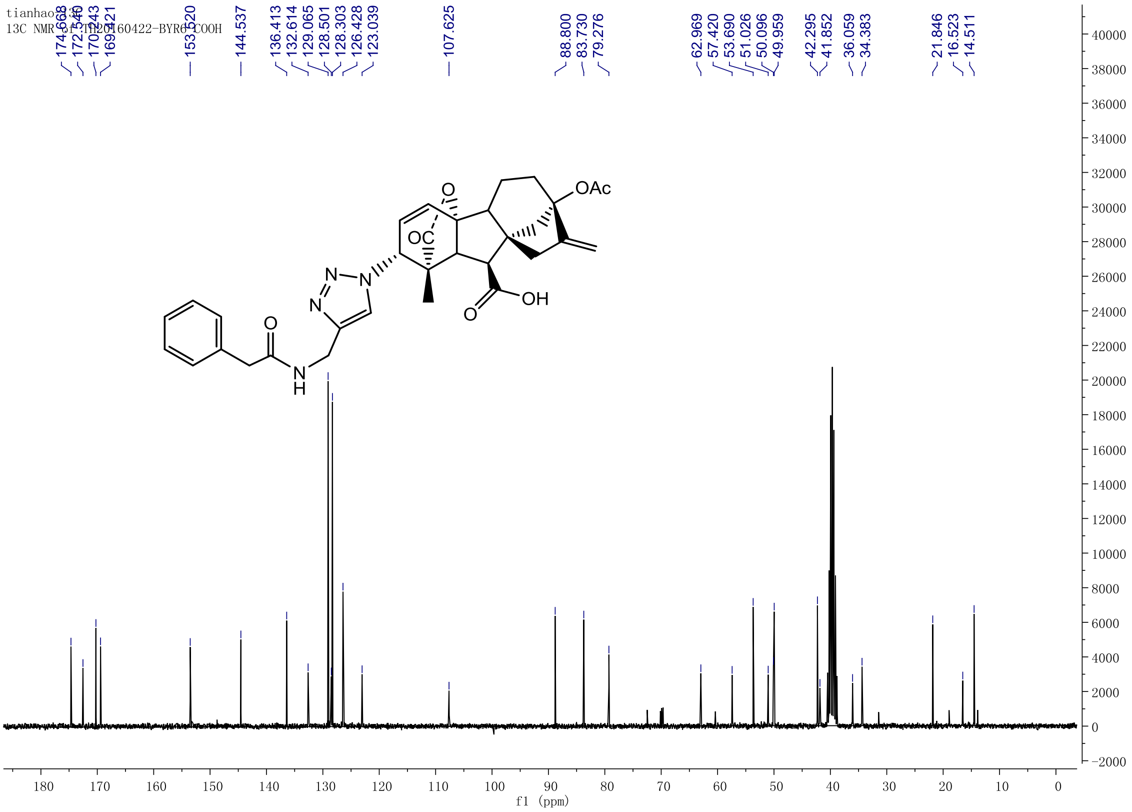


HRMS of compound **10b.**

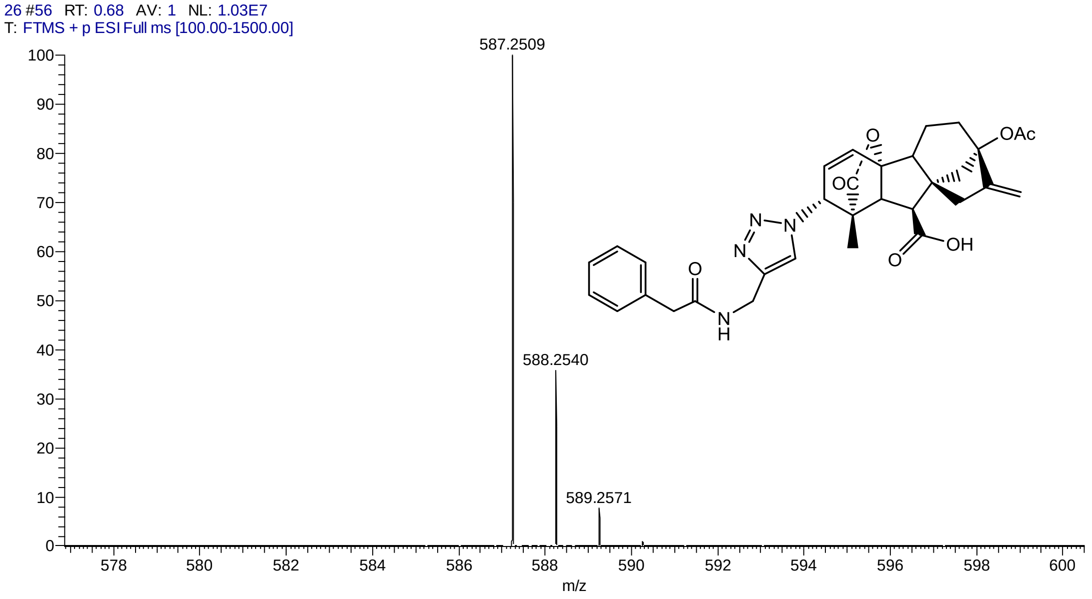

$^{1}$H-NMR spectrum of compound **10c**.

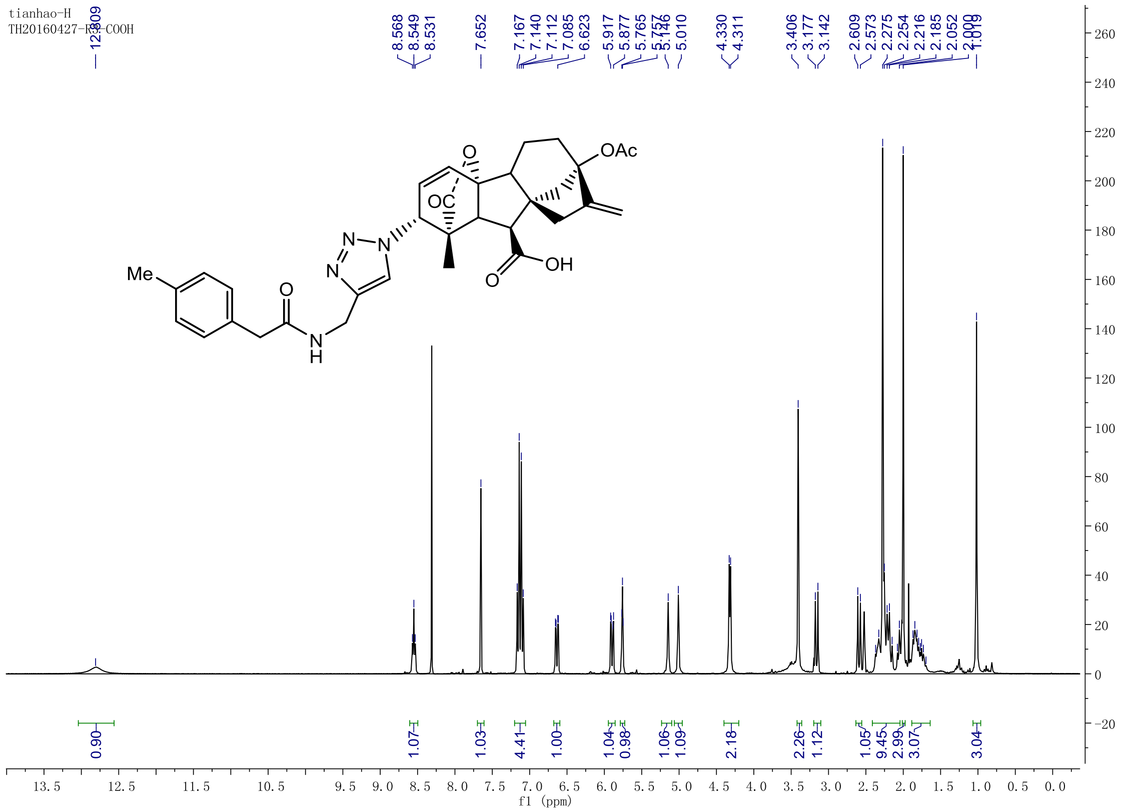


$^{13}$C-NMR spectrum of compound **10c**.

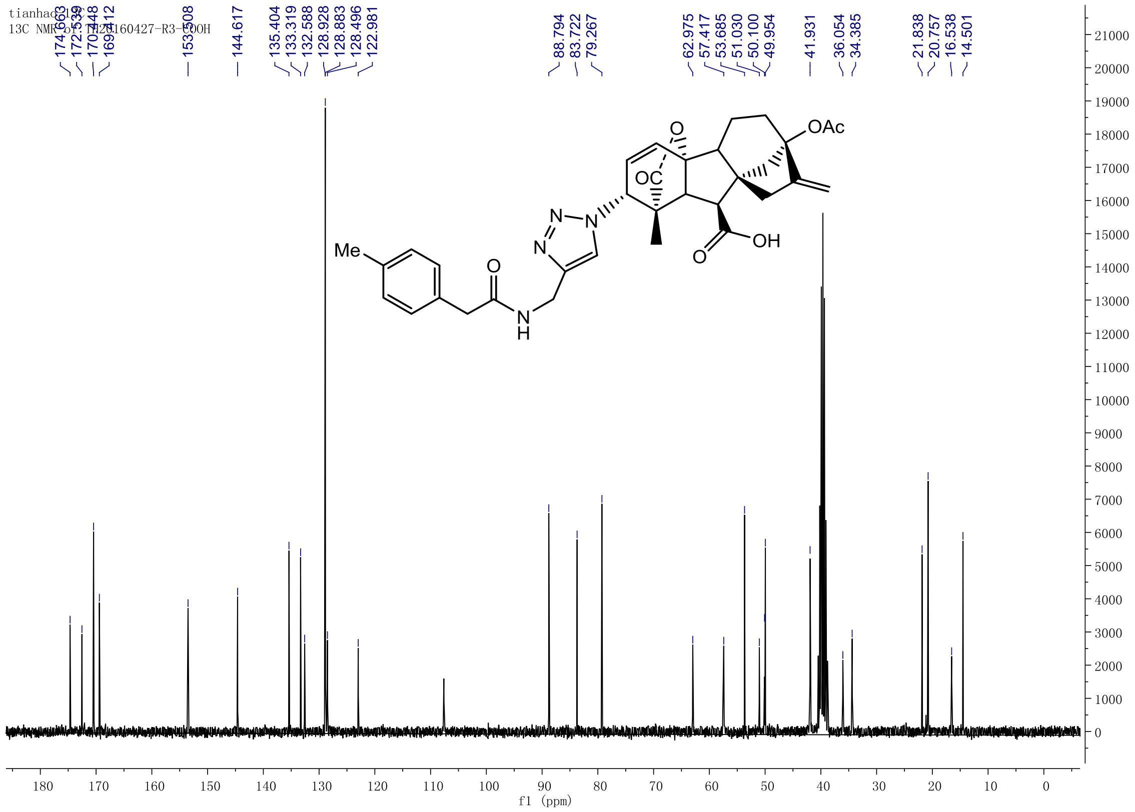

HRMS of compound **10c**.

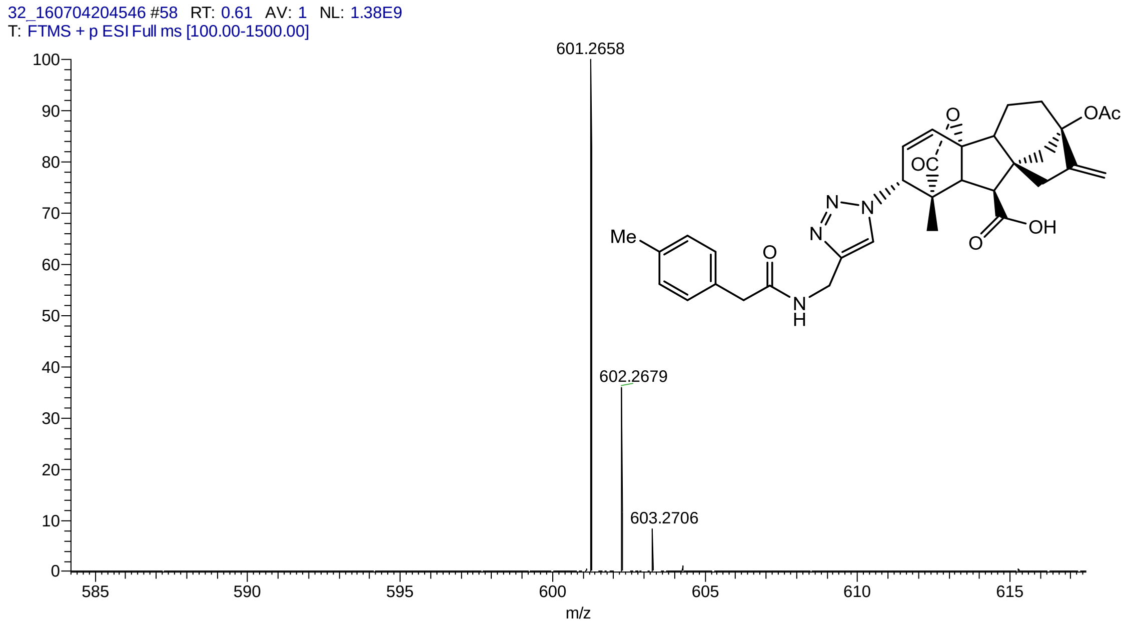


$^{1}$H-NMR spectrum of compound **10d**.

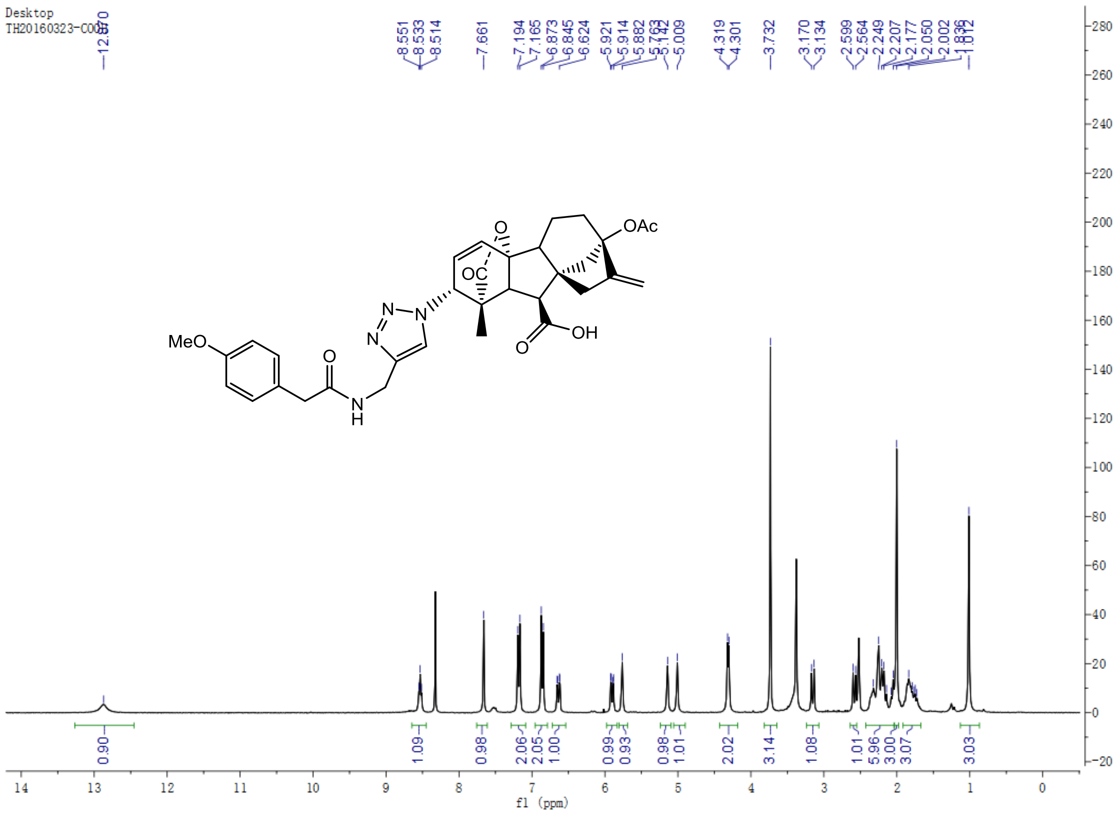

$^{13}$C-NMR spectrum of compound **10d**.

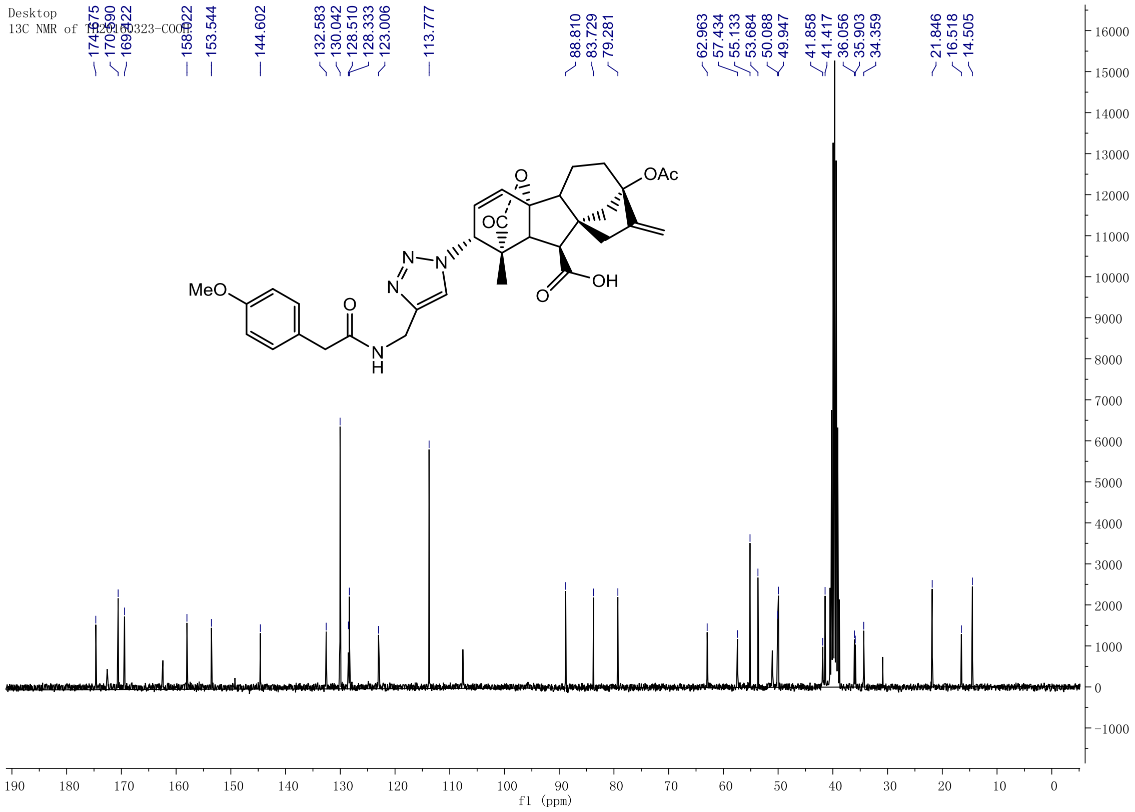


HRMS of compound **10d**.

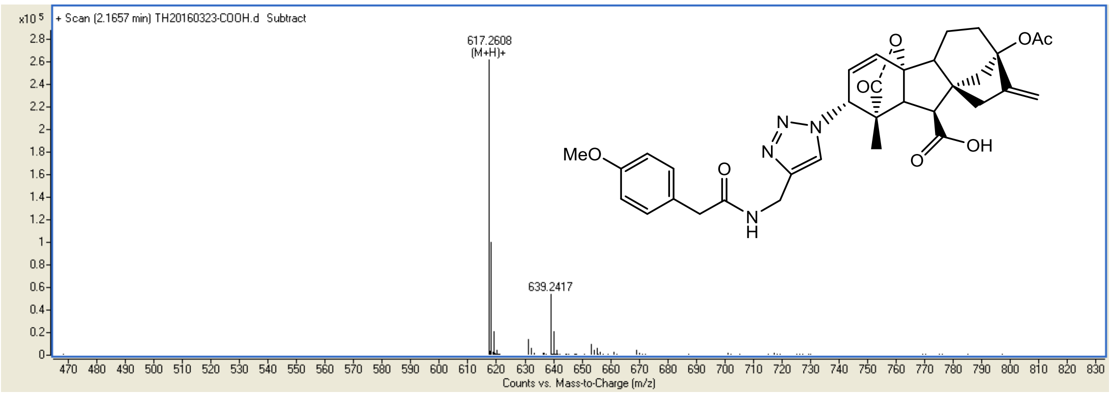


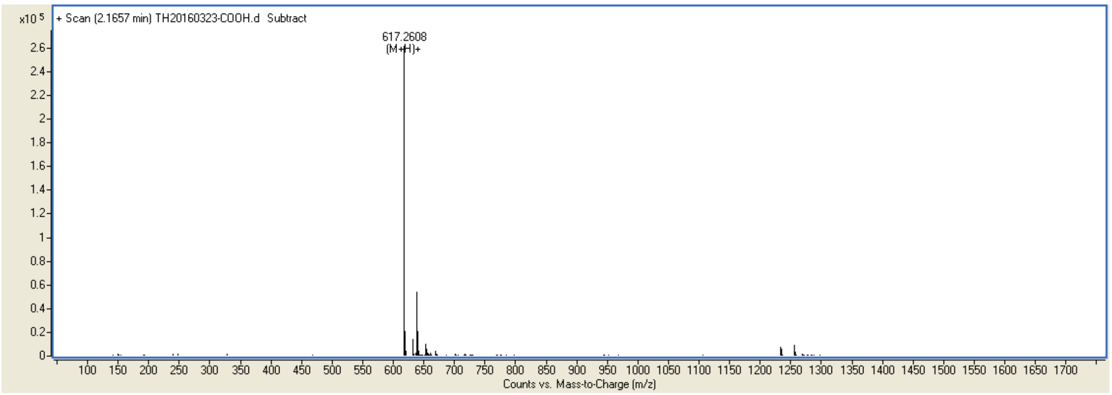

$^{1}$H-NMR spectrum of compound **10e**.

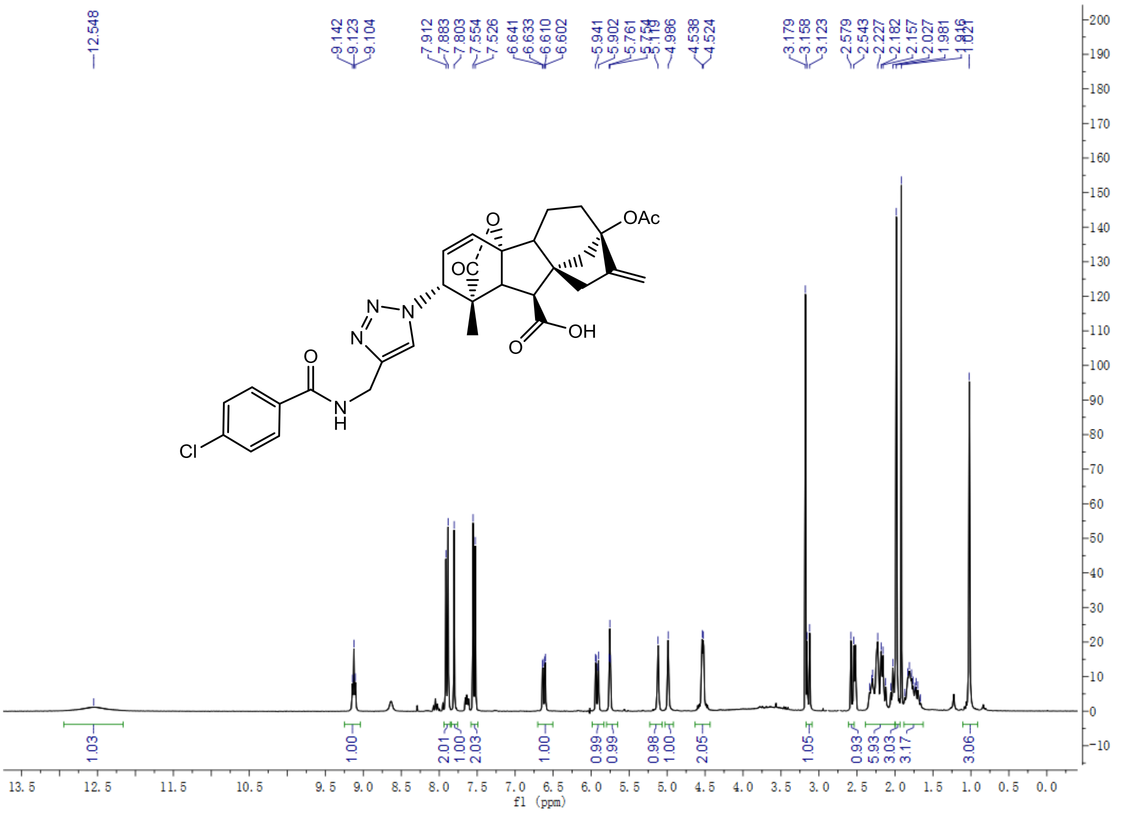


$^{13}$C-NMR spectrum of compound **10e**.

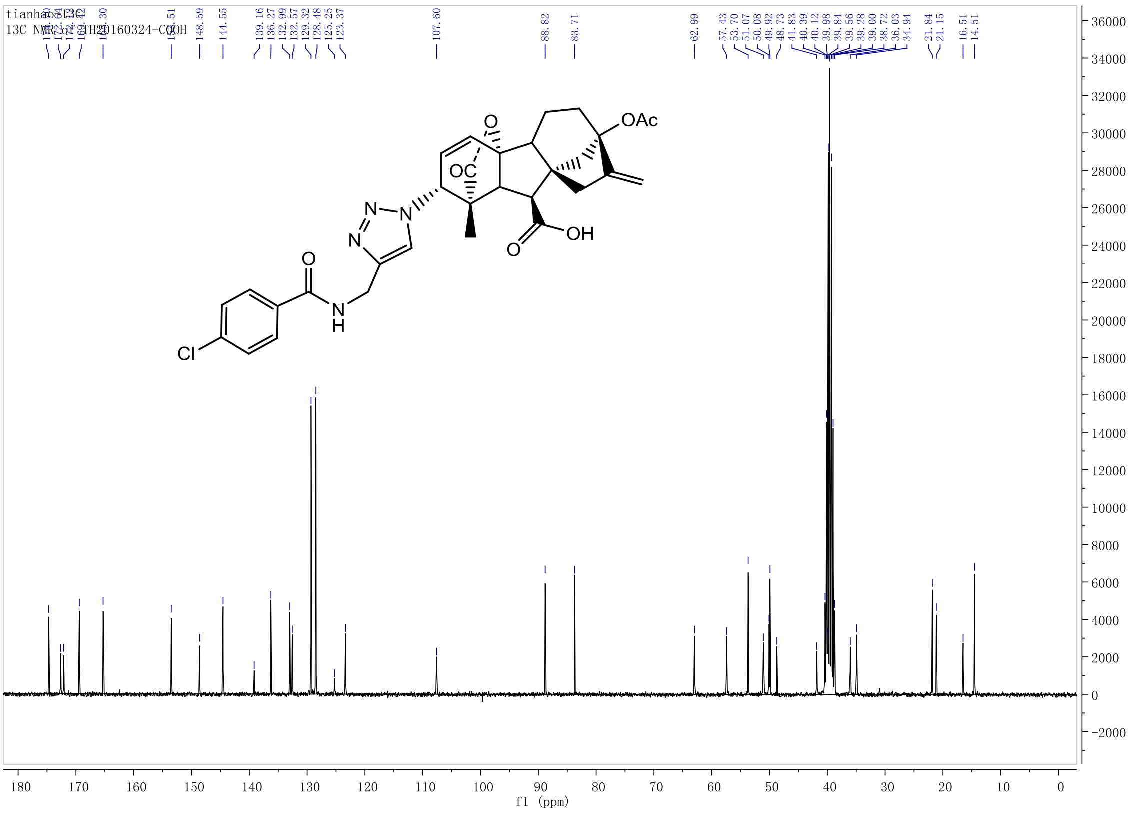

HRMS of compound **10e**.

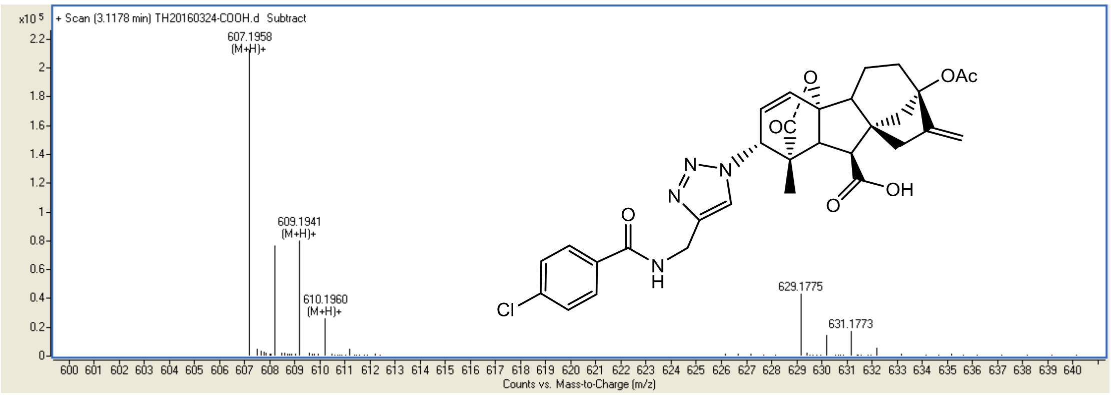


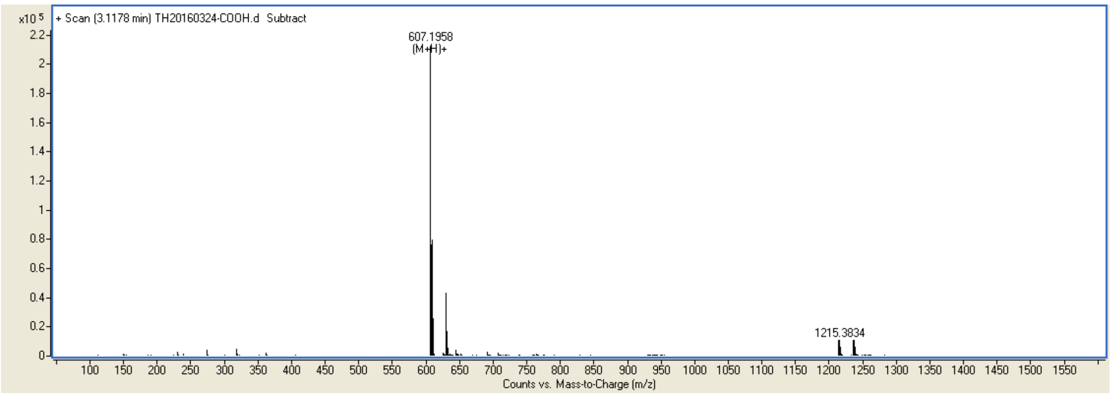


$^{1}$H-NMR spectrum of compound **10f**.

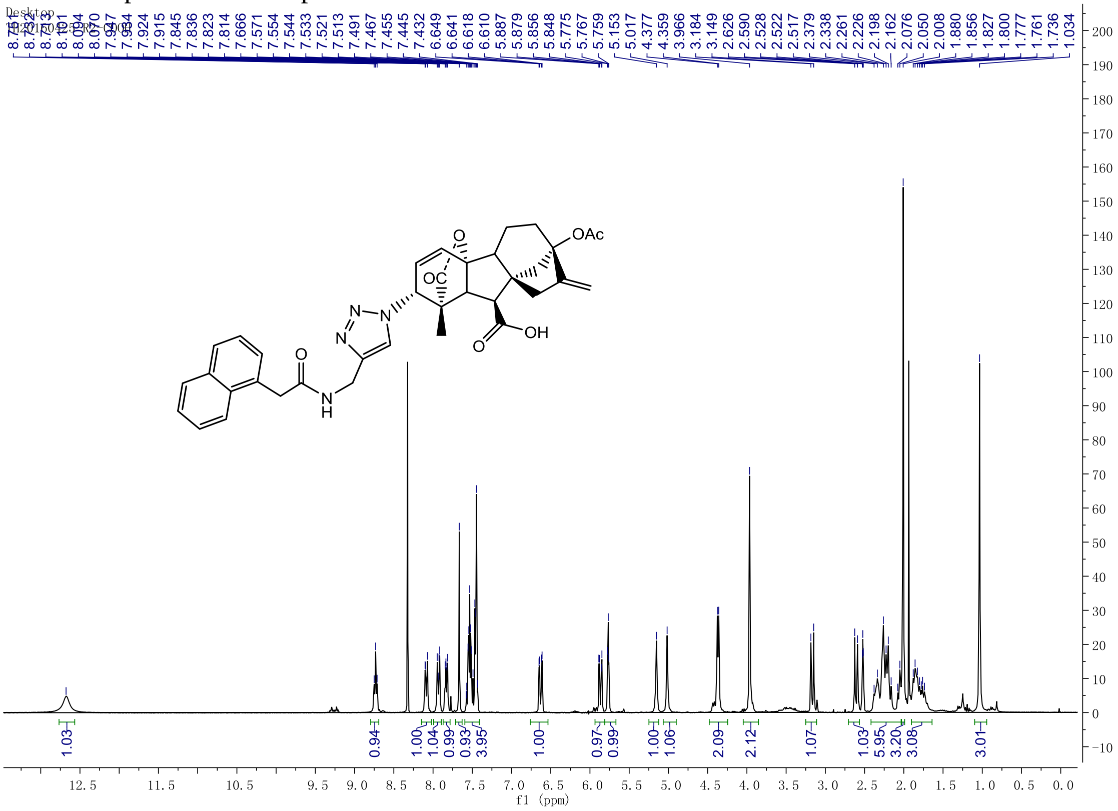

$^{13}$C-NMR spectrum of compound **10f**.

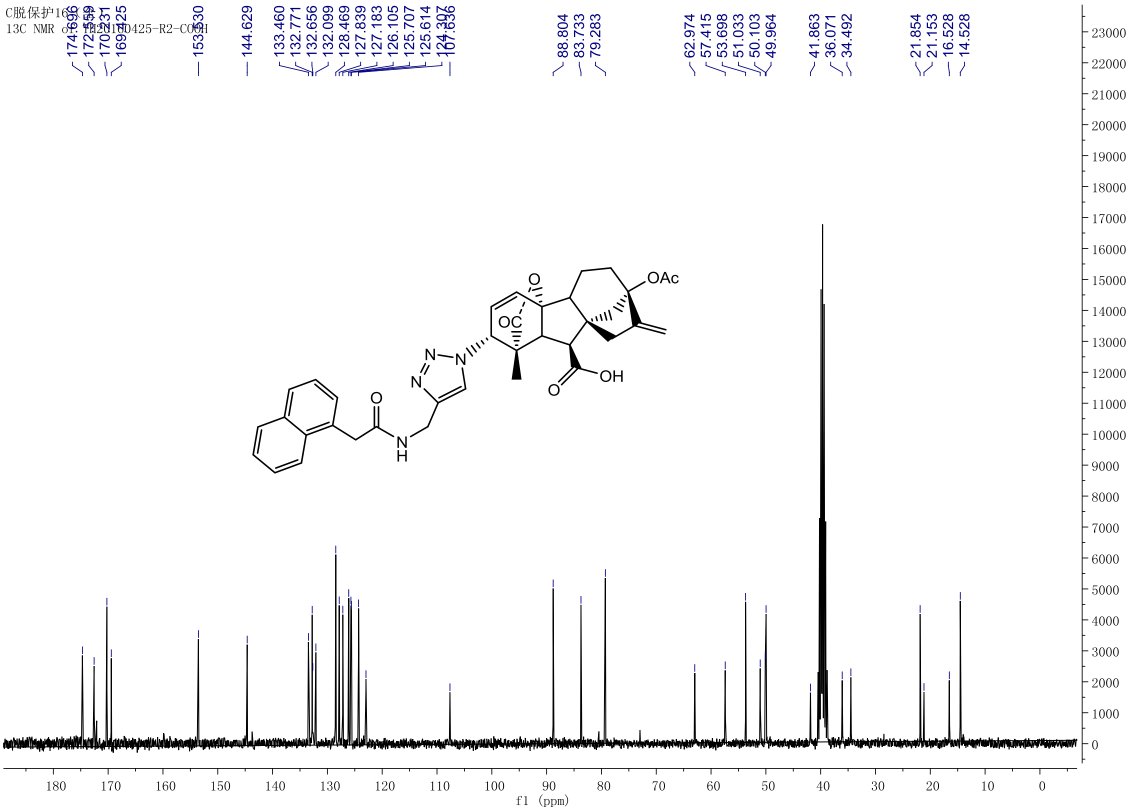


HRMS of compound **10f**.

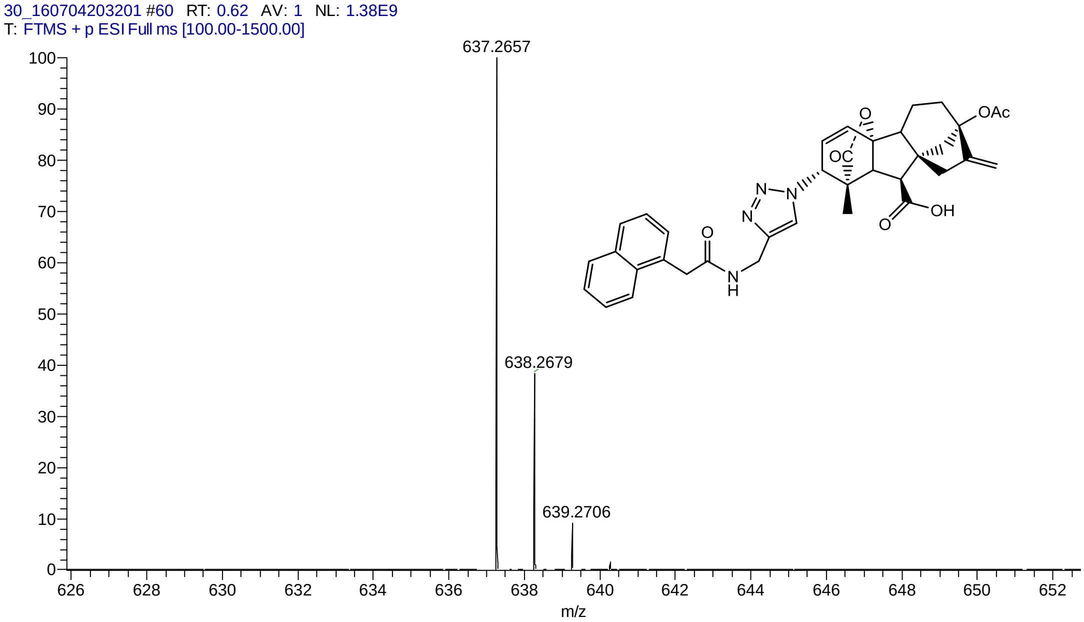

$^{1}$H-NMR spectrum of compound **10g**.

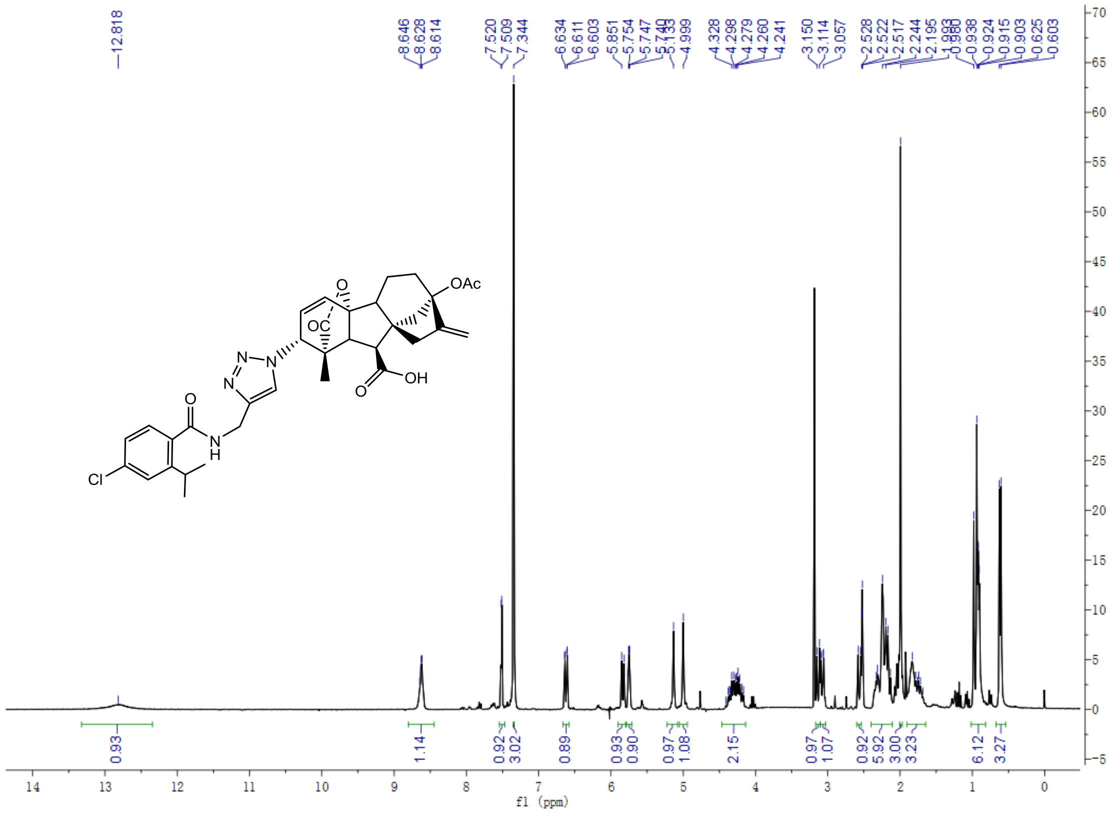


$^{13}$C-NMR spectrum of compound **10g**.

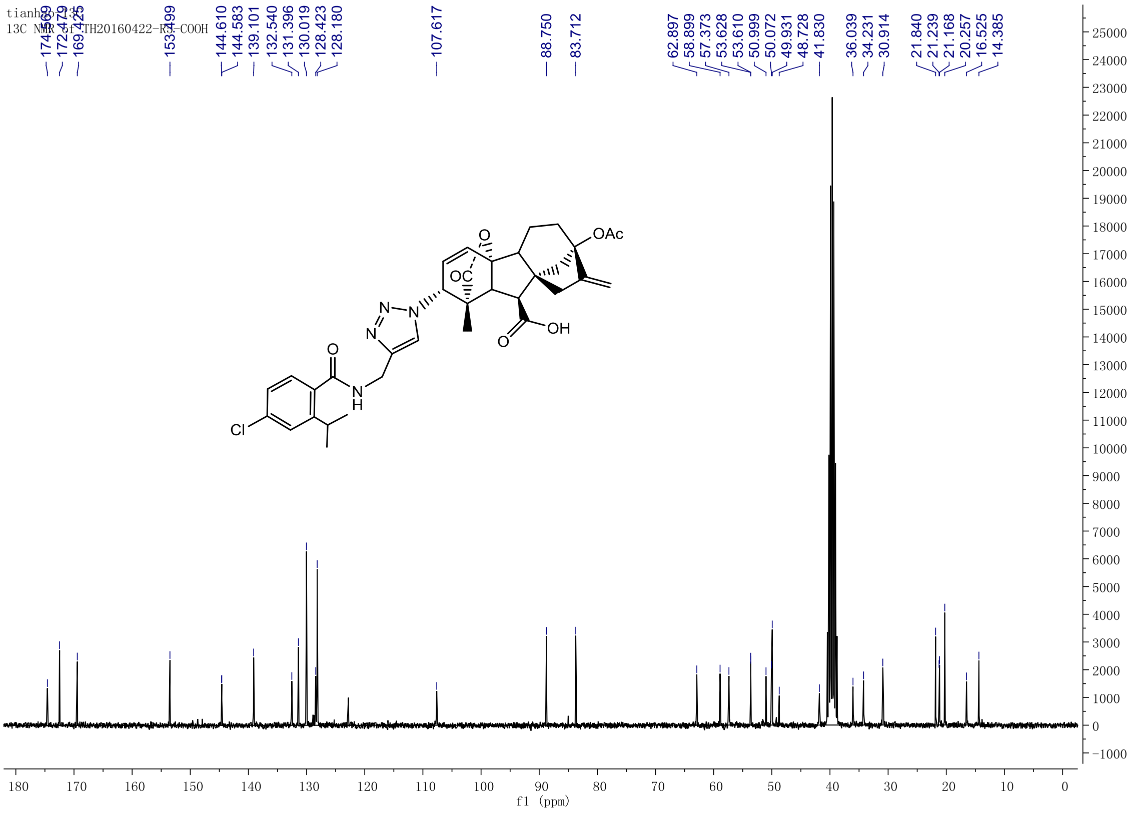

HRMS of compound **10g**.

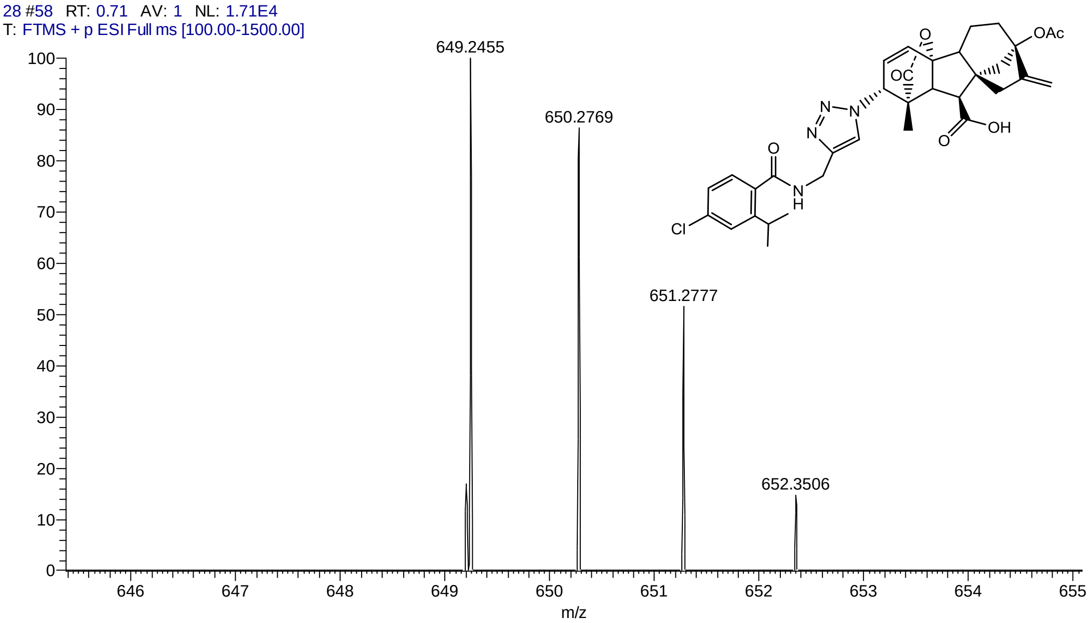


$^{1}$H-NMR spectrum of compound **10h**.

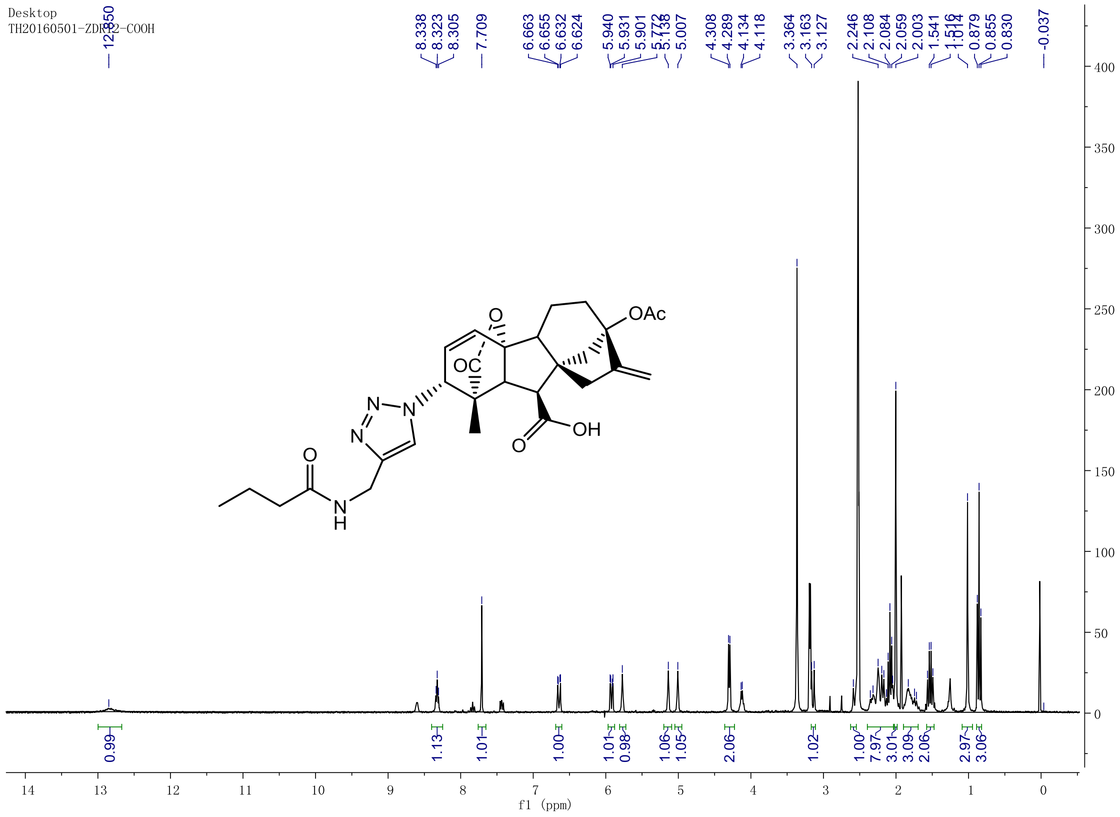

$^{13}$C-NMR spectrum of compound **10h**.

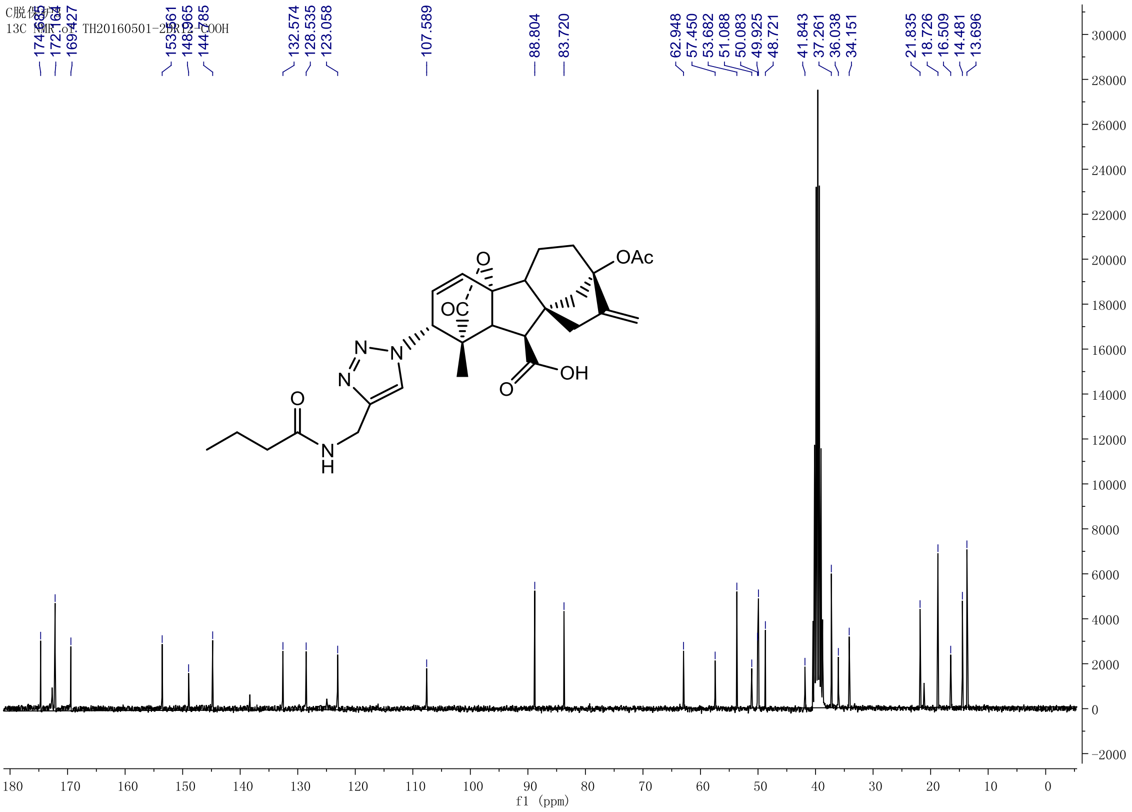


HRMS of compound **10h**.

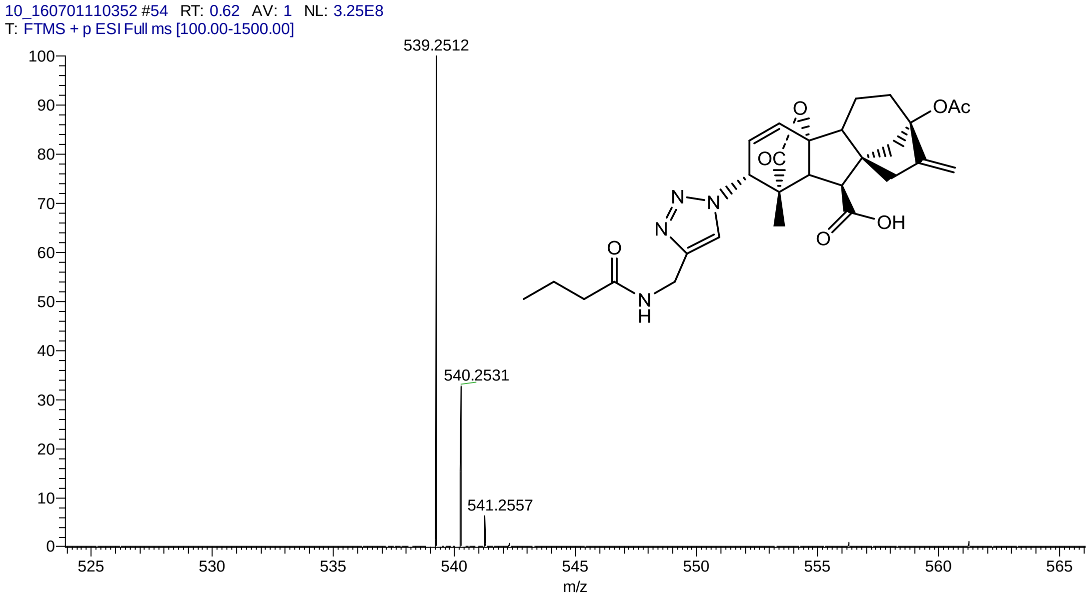

$^{1}$H-NMR spectrum of compound **10i**.

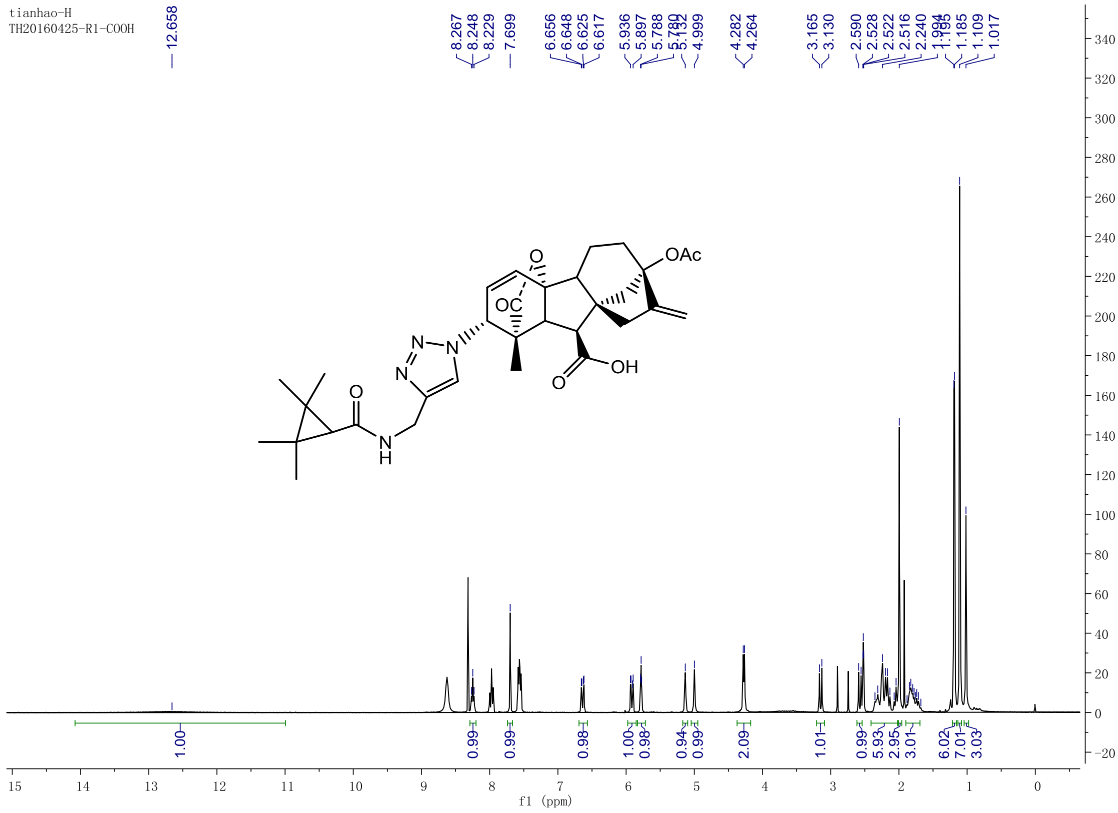


$^{13}$C-NMR spectrum of compound **10i**.

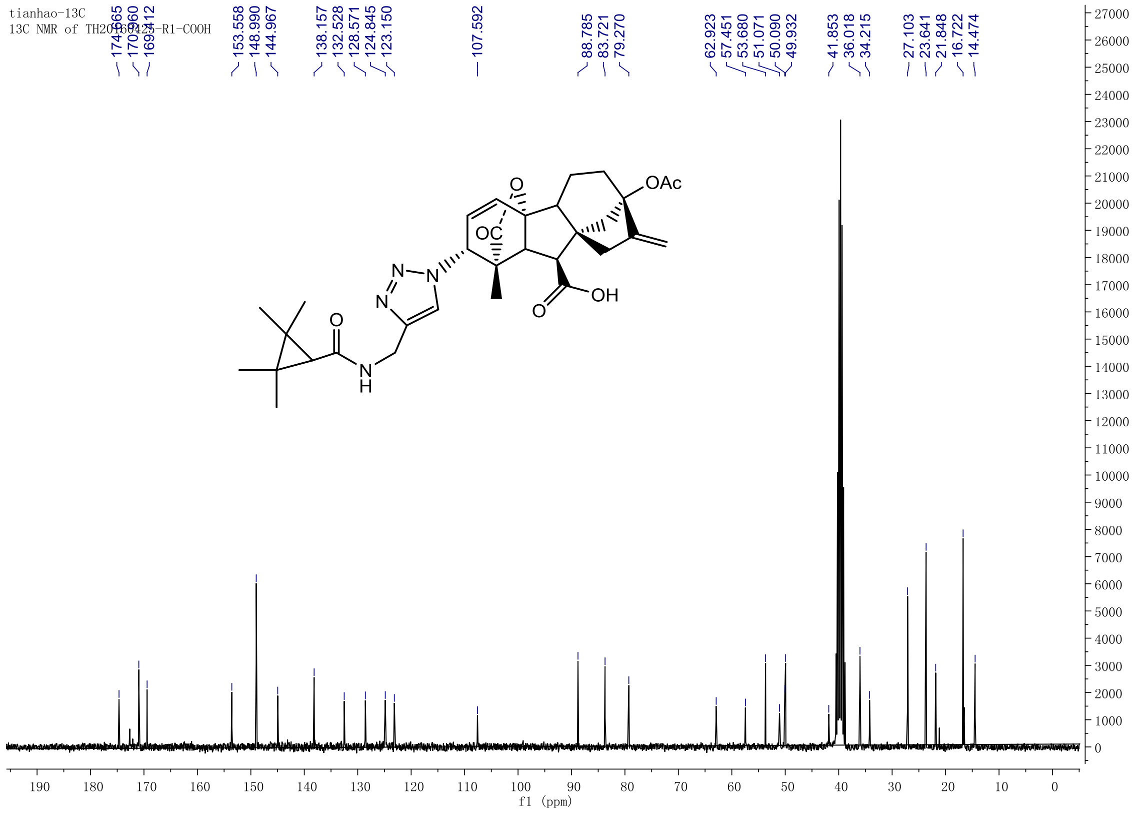

HRMS of compound **10i**.

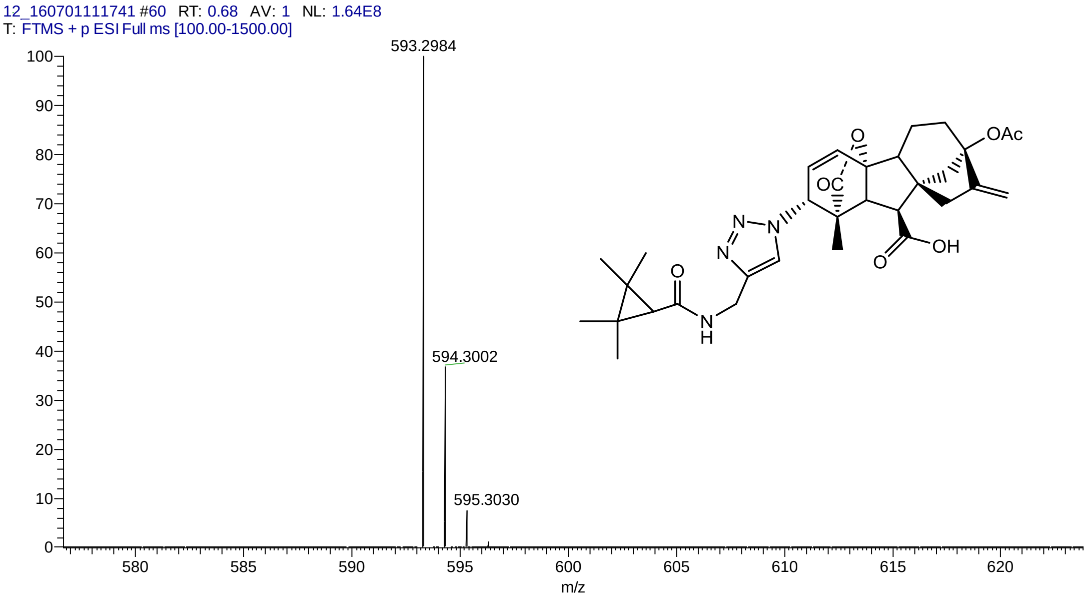


<sup></sup>

$^{1}$H-NMR spectrum of compound **10j**.

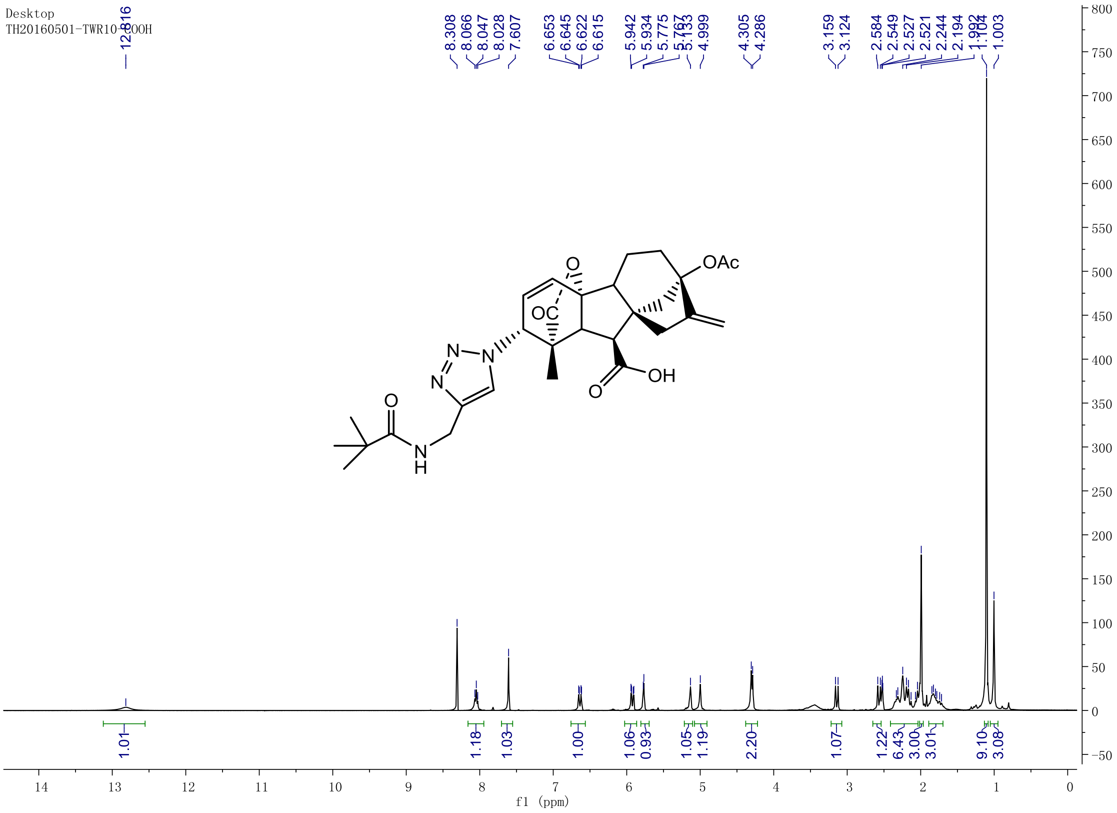

$^{13}$C-NMR spectrum of compound **10j**.

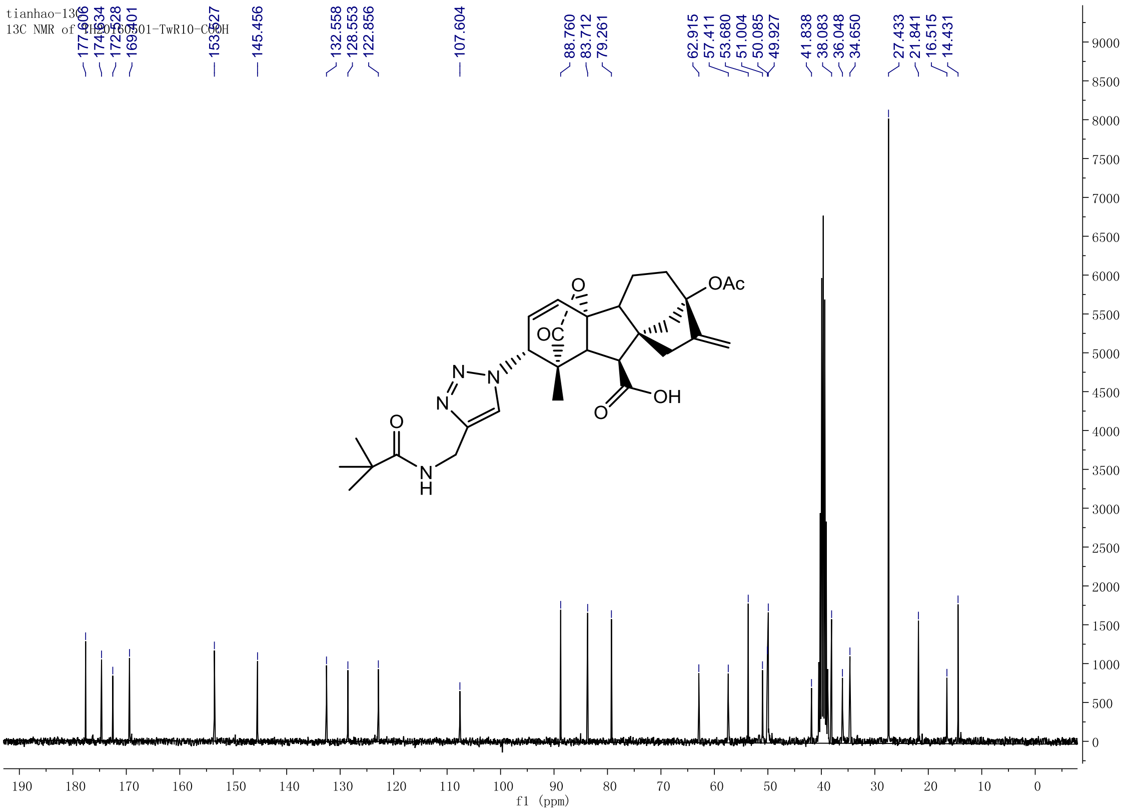


HRMS of compound **10j**.

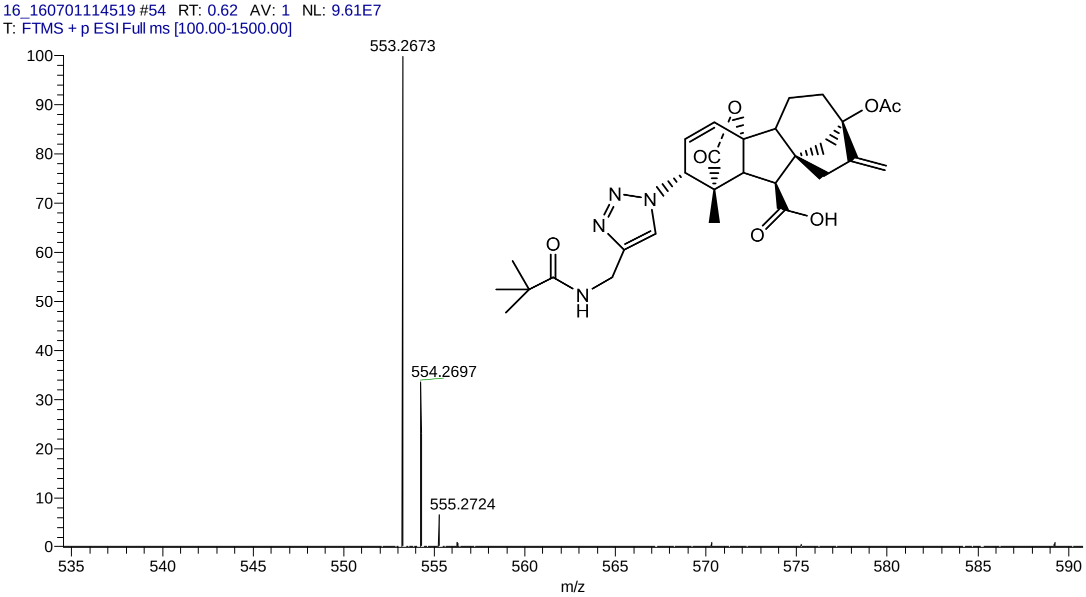

$^{1}$H-NMR spectrum of compound **10k**.

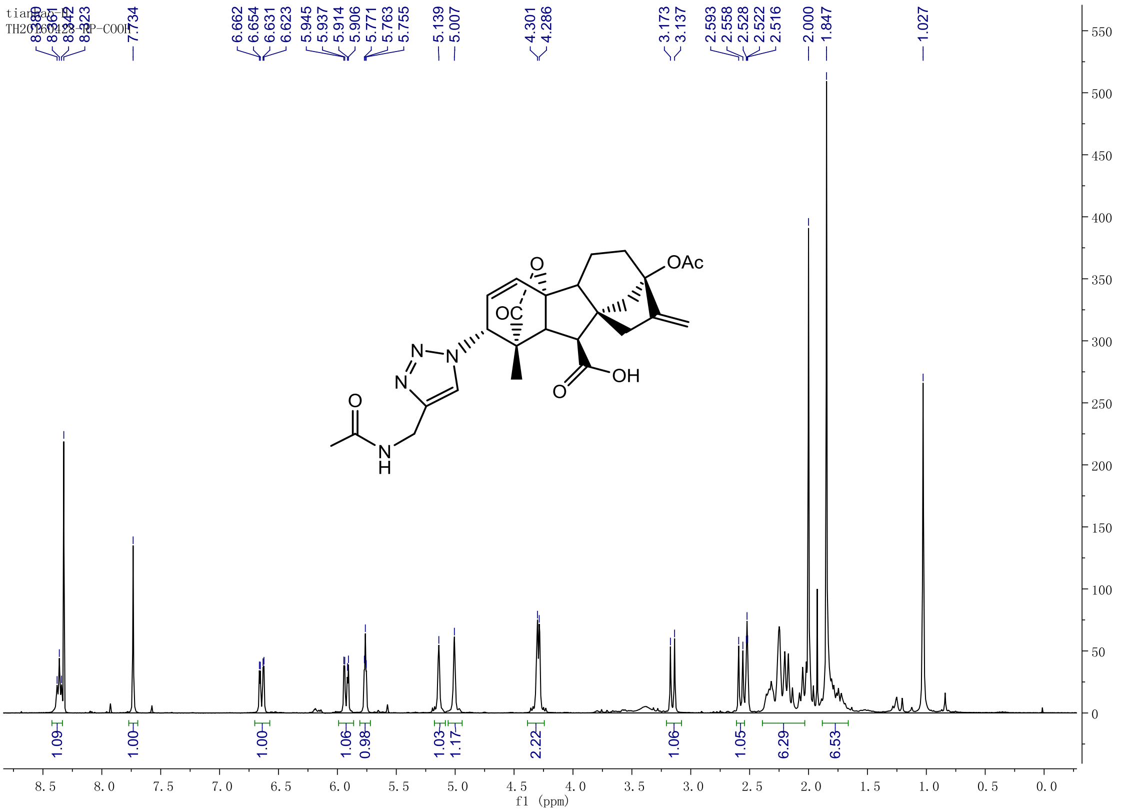


$^{13}$C-NMR spectrum of compound **10k**.

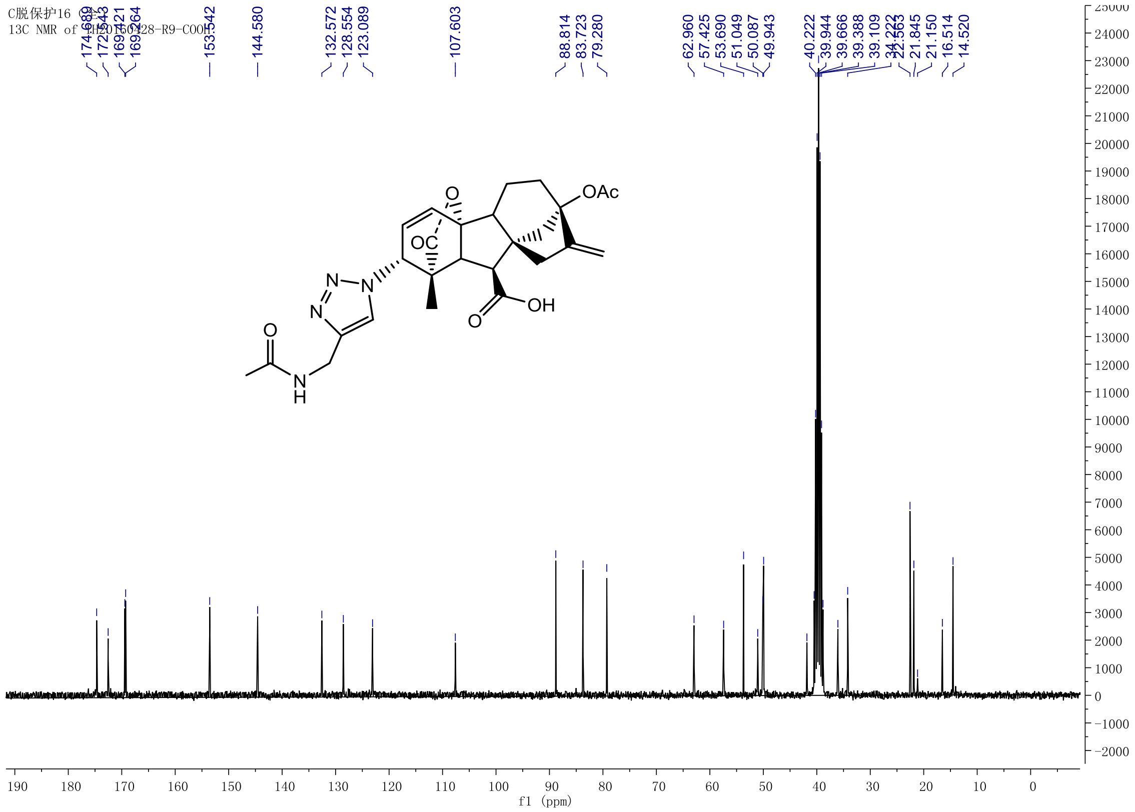

HRMS of compound **10k**.

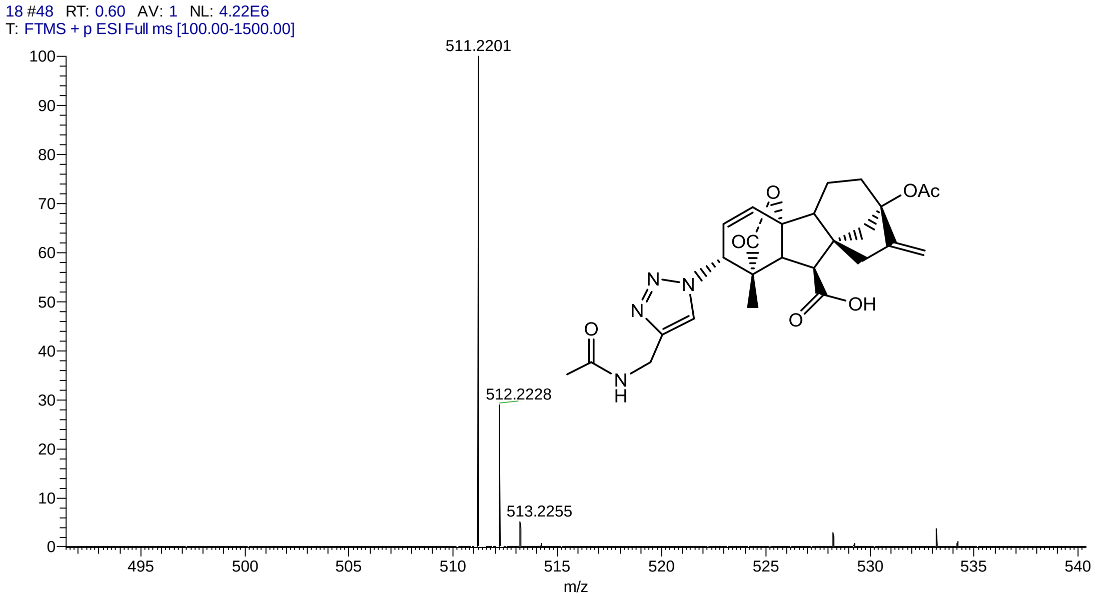


$^{1}$H-NMR spectrum of compound **10l**.

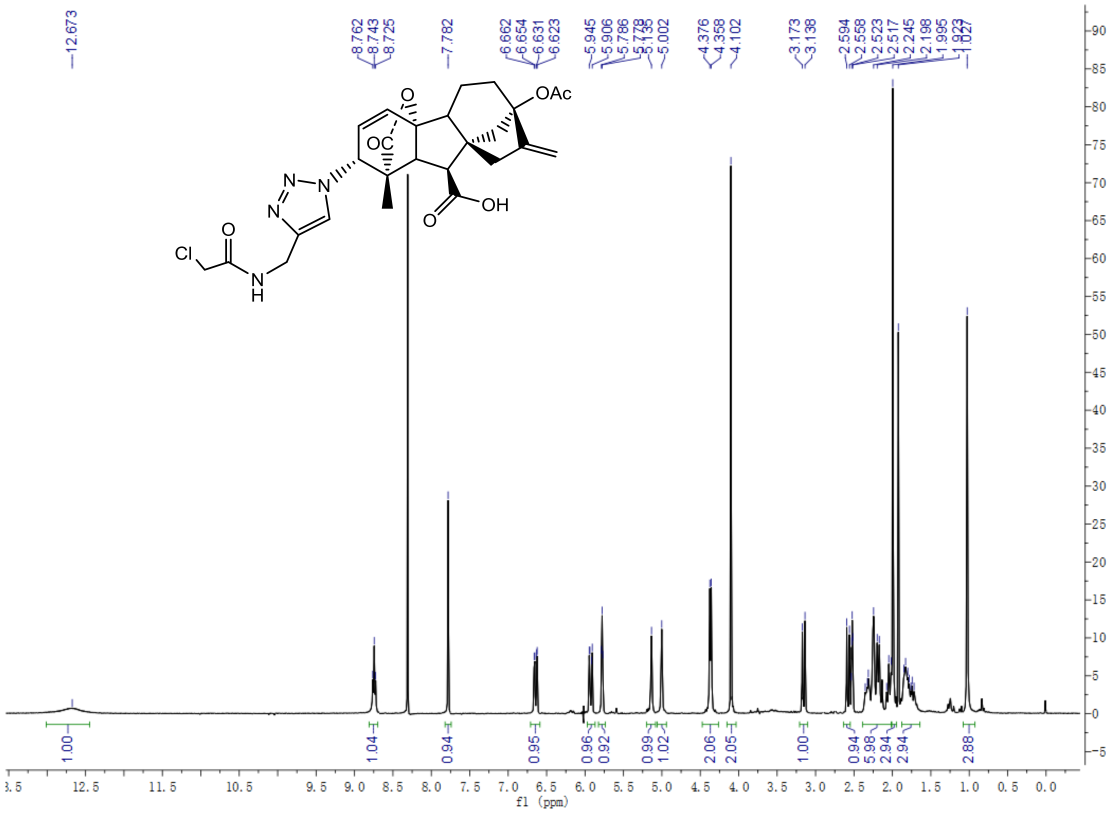

$^{13}$C-NMR spectrum of compound **10l**.

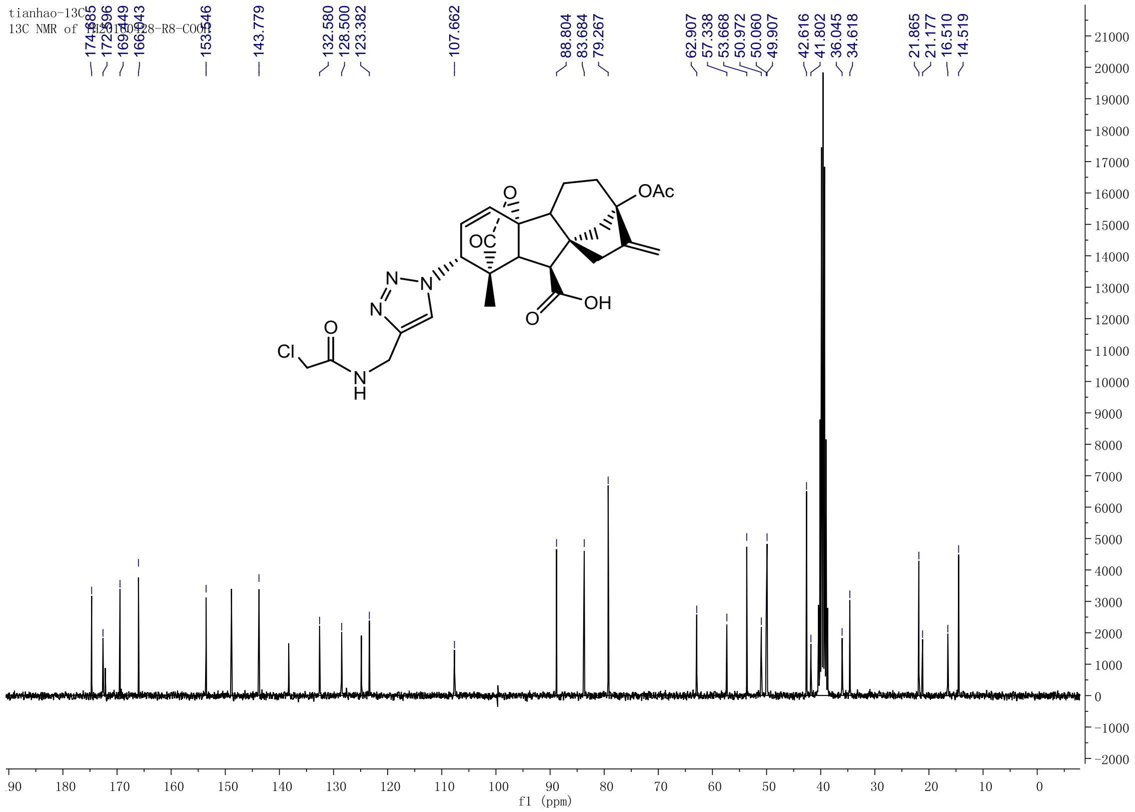


HRMS of compound **10l**.

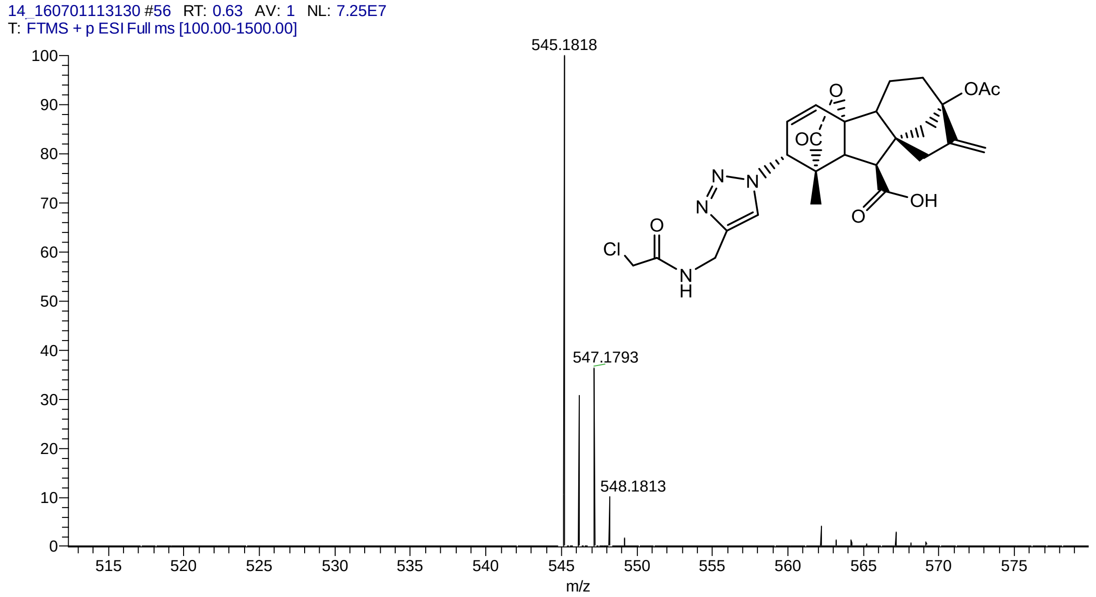

$^{1}$H-NMR spectrum of compound **10m**.

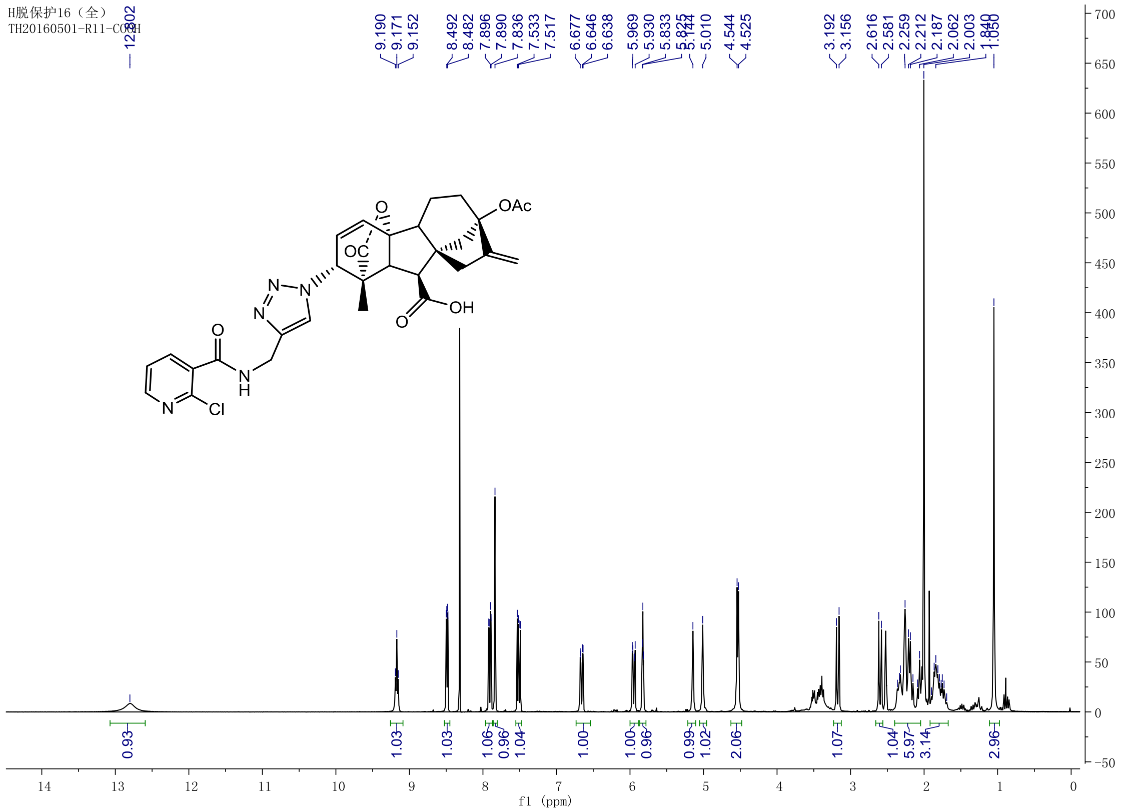


$^{13}$C-NMR spectrum of compound **10m**.

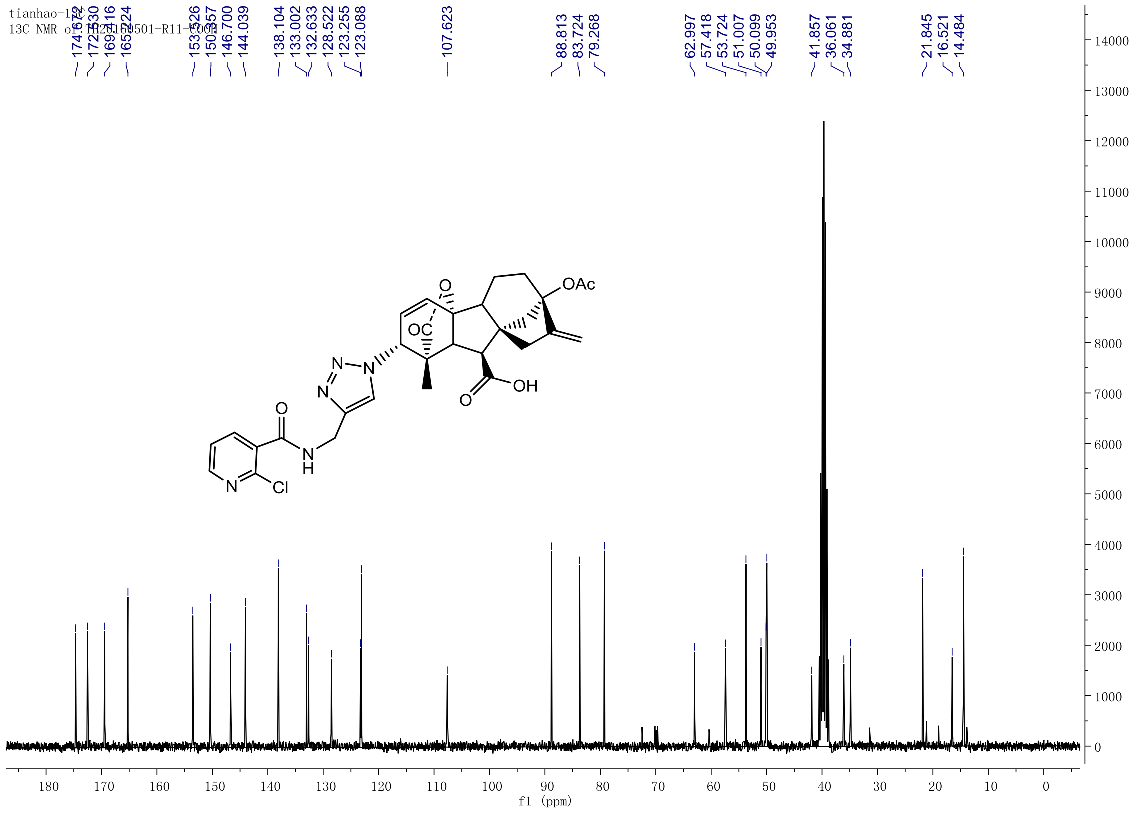

HRMS of compound **10m**.

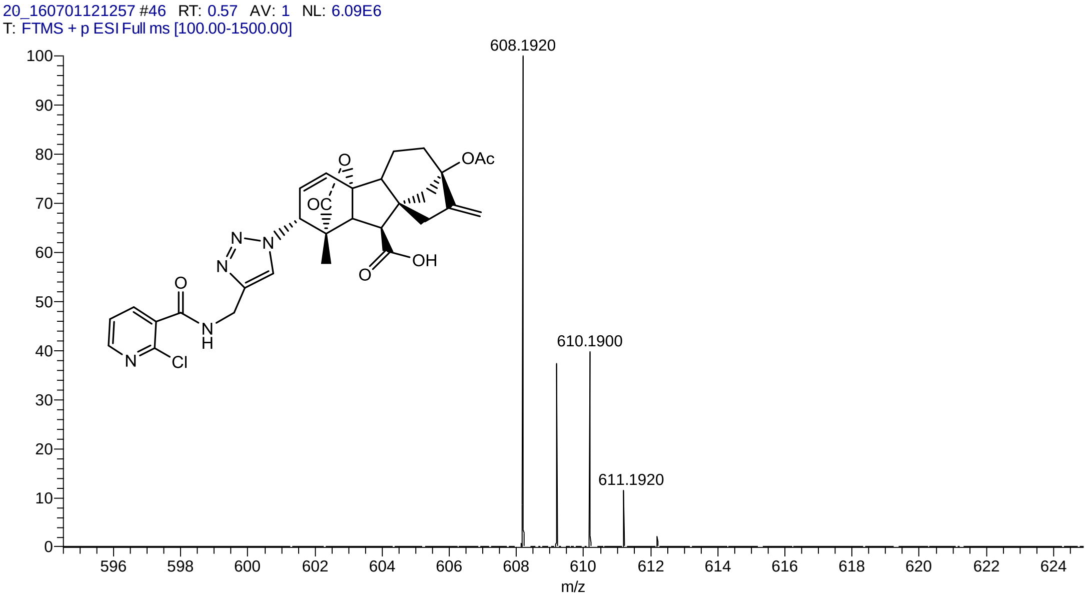


$^{1}$H-NMR spectrum of compound **10n**.

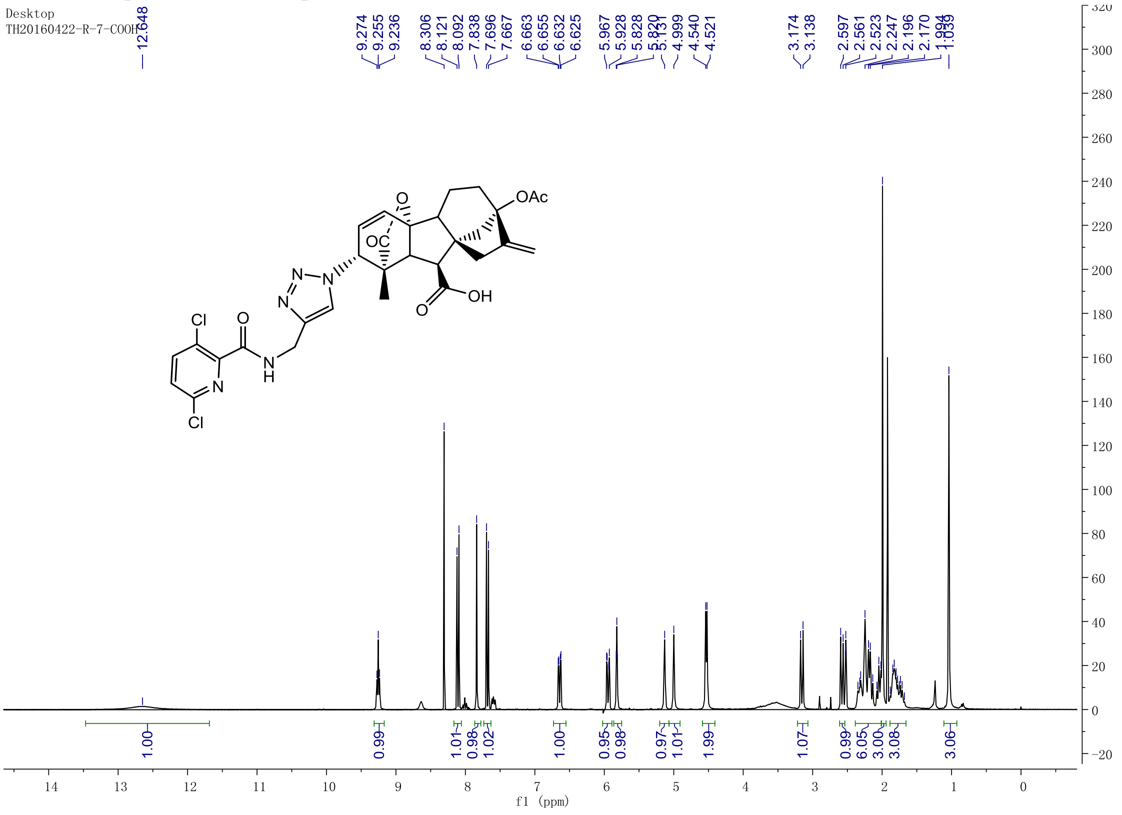

$^{13}$C-NMR spectrum of compound **10n**.

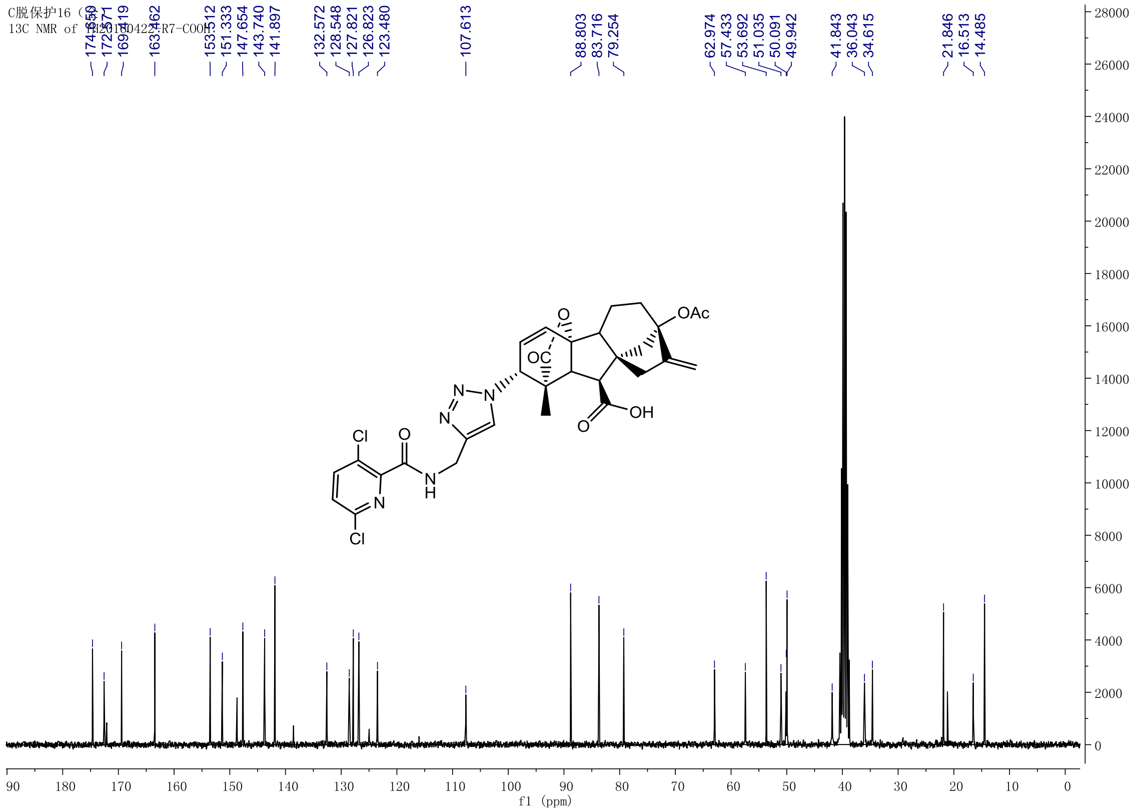


HRMS of compound **10n**.

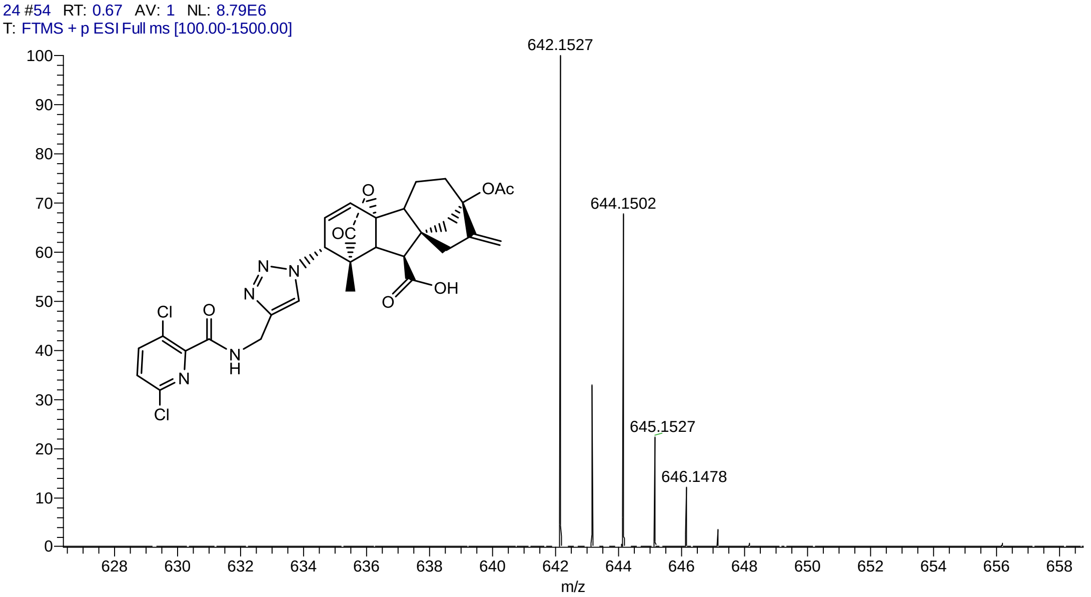

$^{1}$H-NMR spectrum of compound **10o**.

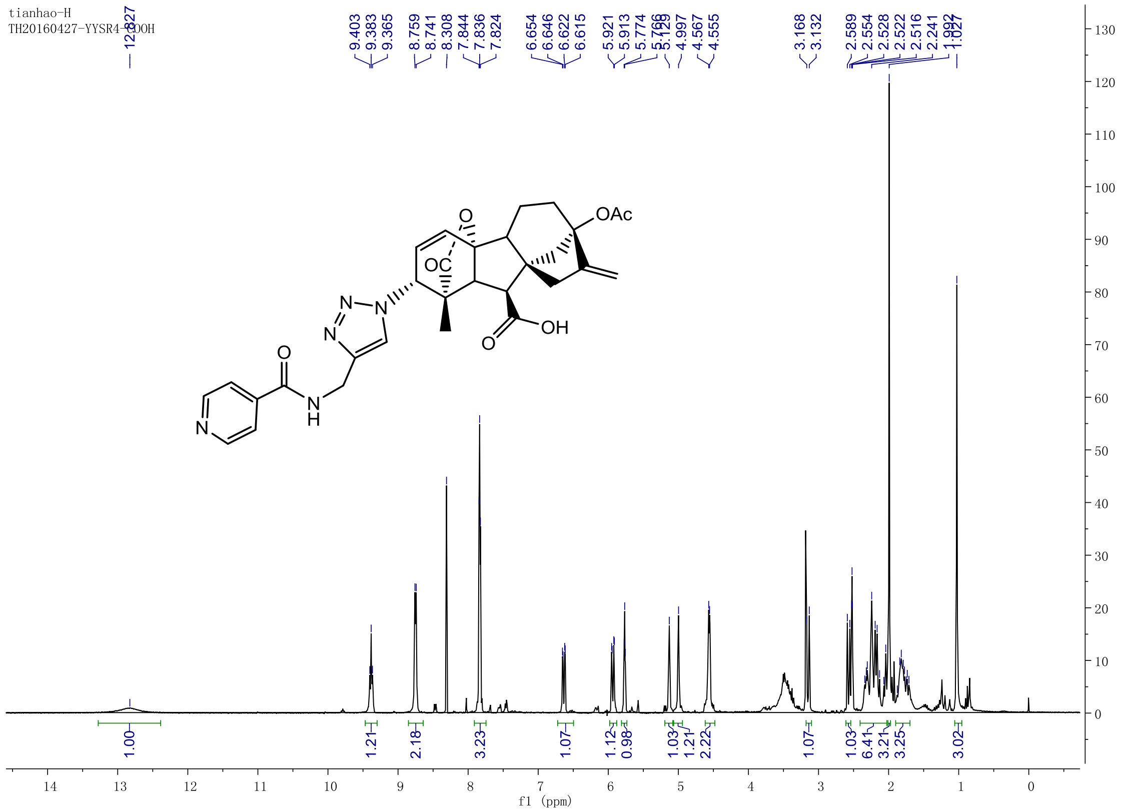


$^{13}$C-NMR spectrum of compound **10o**.

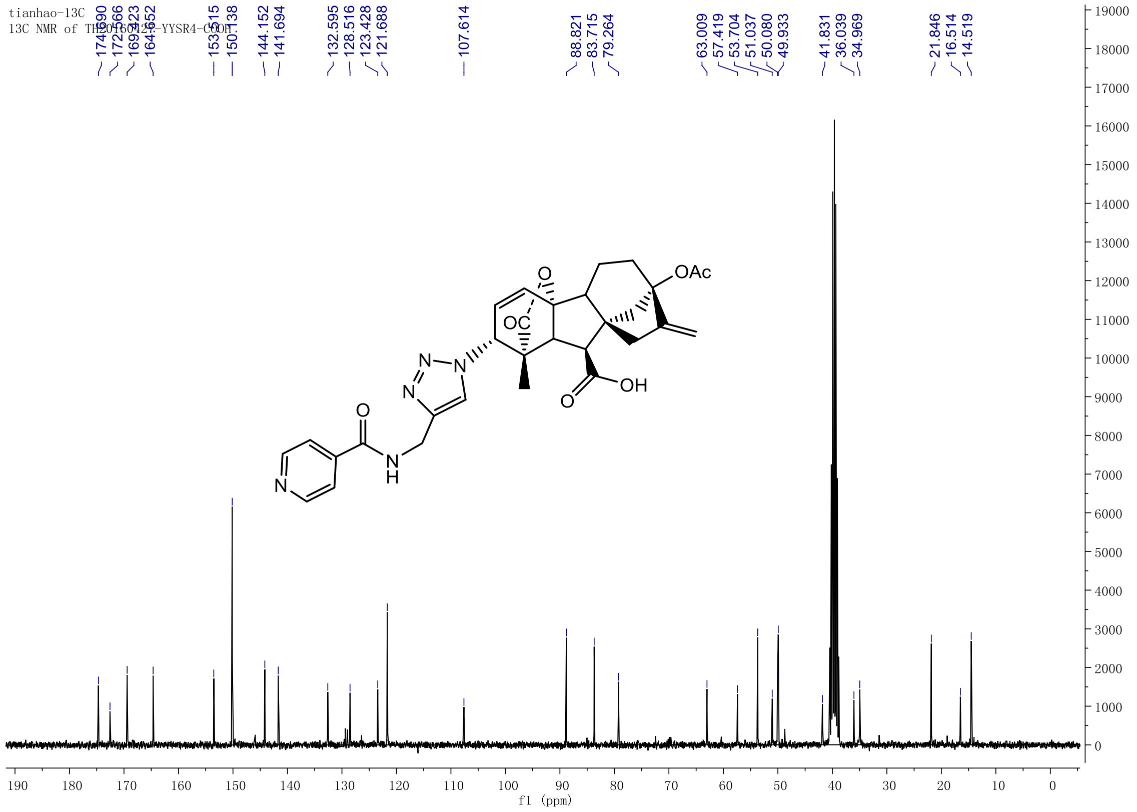

HRMS of compound **10o**.

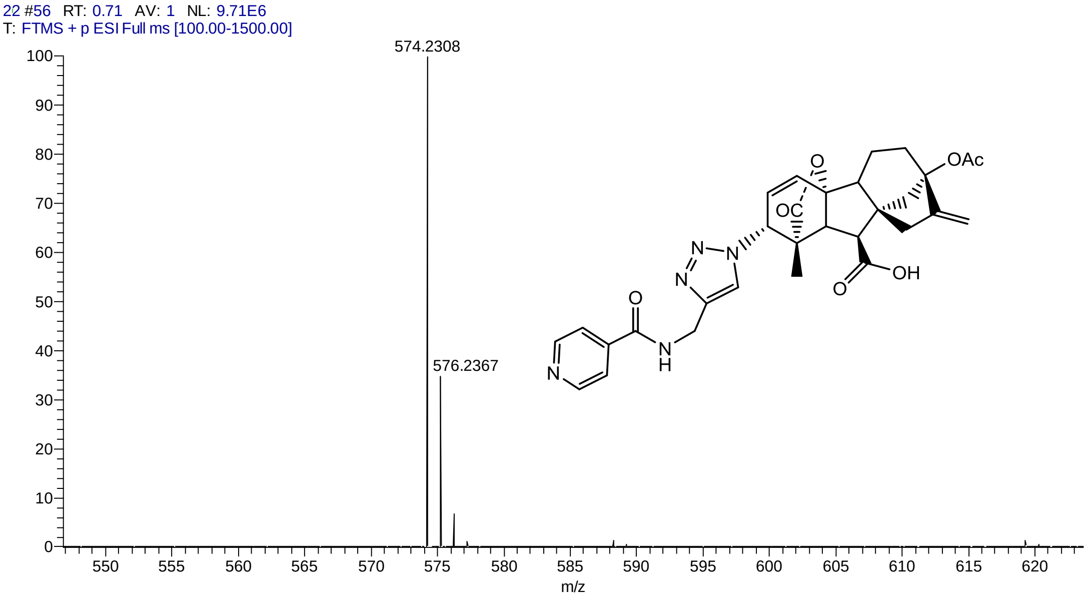

Supplement: Supplementary file 1 [file molecules-22-00694-s001.pdf]
